# Changes in the Distribution Preference of Soil Microbial Communities During Secondary Succession in a Temperate Mountain Forest

**DOI:** 10.3389/fmicb.2022.923346

**Published:** 2022-06-17

**Authors:** Peikun Li, Jian Zhang, Senlin Wang, Panpan Zhang, Wenju Chen, Shengyan Ding, Jingjing Xi

**Affiliations:** ^1^Key Laboratory of Geospatial Technology for the Middle and Lower Yellow River Regions, Henan University, Ministry of Education, Kaifeng, China; ^2^College of Geography and Environmental Science, Henan University, Kaifeng, China; ^3^College of Life Sciences, Henan Agricultural University, Zhengzhou, China; ^4^College of Resources and Environment Sciences, Henan Agricultural University, Zhengzhou, China; ^5^State Key Laboratory of Biocontrol, School of Ecology, Sun Yat-sen University, Shenzhen, China

**Keywords:** forest dynamics monitoring plot, niche theory, distribution mechanisms, specialization, species diversity

## Abstract

Soil microbes play a crucial role in a forest ecosystem. However, whether the distribution of bacteria and fungi in different forest succession stages is random or following ecological specialization remains to be further studied. In the present study, we characterized soil bacterial and fungal communities to determine their distribution preference, with different succession communities in a temperate mountain forest. The Kruskal–Wallis method was used to analyze structural differences between bacterial and fungal communities in different succession processes. The specificity of soil microbial distribution in a secondary forest was studied by network analysis. The torus-translation test was used to analyze the species distribution preference of soil microbes in different succession stages. Results showed that the species composition of soil bacteria and fungi differed significantly in different succession processes. The modularity index of fungi (0.227) was higher than that of bacteria (0.080). Fungi (54.47%) had specific preferences than bacteria (49.95%) with regard to forests in different succession stages. Our work suggests that the distribution pattern of most soil microbes in a temperate mountain forest was not random but specialized in temperate mountain forests. Different microbes showed different distribution preferences. Fungi were more sensitive than bacteria during secondary succession in a temperate mountain forest. In addition, microbe–environment relations varied during secondary succession. Our results provided new insight into the mechanism through which complex soil microbial communities responded to changes in forest community succession.

## Introduction

During the renewal and reconstruction of plant communities, succession plays an important role in species composition (Zhang and Shangguan, 2006). Thus, studying the effects of succession on species diversity is important for the renewal and development of forest communities (Fang et al., 2019; Xi et al., 2021). Soil microbes not only play a role in the circulation of soil materials in the soil ecosystem (Bardgett and van der Putten, 2014; De Vries and Wallenstein, 2017), but also produce strong positive feedback to aboveground parts and promote their regeneration and succession (Lucas-Borja and Delgado-Baquerizo, 2019). Many studies have shown that microbes are closely related to plant diversity (Liu et al., 2020). Plants can affect the survival of microbes by changing the input of litter and root exudates (Reynolds et al., 2003; Wardle et al., 2004; Maestre et al., 2009). Microbes indirectly affect the species composition and distribution of plant communities by regulating the turnover of soil nutrients and elements (Zhang Z. F. et al., 2021). However, given the changes in the secondary succession in a temperate mountain forest, the distribution mechanism of microbes in a forest ecosystem remains unclear.

Ecological specialization is the process by which a species adapts to its living environment and persists in that environment (Devictor et al., 2010; Poisot et al., 2011; Xi et al., 2021). In a forest community, most species have good environmental adaptability and stable growth and reproduction (Bevill and Louda, 1999; Xi et al., 2021). The habitat division hypothesis provides a conceptual framework to explain the maintenance of soil microbial diversity (Silvertown, 2004; Chen et al., 2018b). Its core principle is to assume that environmental conditions are spatially structured and that this structure is reflected in the distribution of species through their association with different habitats (Svenning, 1999; Gao et al., 2017; Chen et al., 2018b,^2020^). However, most of these studies have focused on woody plant communities (Harms et al., 2001; Comita et al., 2007; Lai et al., 2009; Bin et al., 2016; Guo et al., 2017). Few studies have investigated the distribution preference of forest soil microbial community. Whether the distribution of soil microbes is random or is based on ecological specialization during secondary succession in a temperate mountain forest remains to be elucidated.

The interaction between aboveground and belowground biotic communities drives community dynamics and ecosystem functioning (Hooper et al., 2000; Wardle et al., 2004; Van Dam and Heil, 2011). As an important component of belowground microbes, bacteria and fungi can influence plant growth and fitness, plant community dynamics, and biogeochemical cycling (Landeweert et al., 2001; Van der Heijden et al., 2008; Liang et al., 2016; Almario et al., 2017; Wu et al., 2018). Plant significantly forms soil microbe communities through host specificity (Ishida et al., 2007; Tedersoo et al., 2012; Martinez-Garcia et al., 2015; Ciccolini et al., 2016; Linde et al., 2018; Sepp et al., 2019), generating diverse substrates and changing microhabitats (Wardle, 2006; Dickie, 2007; Aponte et al., 2010). However, the role of forest community succession in the maintenance of soil microbial diversity remains poorly known.

Fungi and bacteria differ substantially in their dispersal capability (typically thought to be greater for bacteria and more variable across taxa for fungi because of the differences in propagule size and number) and growth habit (generally unicellular versus filamentous growth) (Powell et al., 2015). Compared with fungi, bacteria are more resilient to disturbance because of their relatively high intrinsic growth rates and unicellular nature (Wardle, 2002). However, whether bacteria and fungi have different distribution mechanisms of forest community succession in temperate mountain forest remains unclear.

In this study, four 1 hm^2^ (100 m × 100 m) forest dynamic monitoring plots during secondary succession in a temperate mountain forest were randomly selected in a temperate deciduous broad-leaved forest in China. We characterized soil bacterial and fungal communities to determine their distribution mechanism during secondary succession. This study aimed to (1) identify whether the species distribution of soil microbes is random or followed ecological specialization during secondary succession in a temperate mountain forest; and to (2) assess if the differences in the distribution mechanism between bacteria and fungi in the face of forest community succession. The results can improve understanding of the distribution mechanism of fungi and bacteria in response to changes during secondary succession in a temperate mountain forest in forest ecosystems.

## Materials and Methods

### Study Site

This study was conducted in Baiyun Mountain (33°38′-33°34′N, 111°48′-111°52′ E, 1,500 m above sea level), which is located in Hennan Province in east China. It belongs to temperate deciduous broad-leaved forest, with a forest coverage rate of 98.5%. The mean annual precipitation at the study site was 1,200 mm, with the wet season occurring from July to September. In addition, the mean annual temperature was 18°C (Lin, 1999; Xu et al., 2014; Chen et al., 2017).

Baiyun Mountain is rich in plant resources. Based on the investigation, a total of 1,991 species of plants were identified (Li et al., 2017). The Baiyun mountain comprised a large area of temperate deciduous broad-leaved forest dominated by *Quercus aliena* var. *acutiserrata*, *Betula platyphylla*, *Carpinus turczaninowii*, and *Toxicodendron vernicifluum* (Li et al., 2017).

### Sampling Site Setting and Soil Sampling

Four forest succession, namely, plantation forest, twice-cut forest, once-cut forest, and old-growth forest, were selected on the basis of the different degree of human disturbance in the forest succession to explore the difference in soil microbial diversity during secondary succession in a temperate mountain forest (Supplementary Figure 1).

(I) Plantation forest (A): In the plantation forest, a *Larix kaempferi* forest, which was planted after logging and clearing and was about 20 years old (high disturbance), was considered (Xi et al., 2021). There were 42 species of woody plants in the plot, and the total number of plants was 1,165. In the field, *Larix gmelinii* and *Quercus aliena* var. *acutiserrata* were the dominant species (Supplementary Table 1).

(II) Twice-cut forest (B): In this forest, natural regeneration occurred after once-cutting. Twice-cutting and breeding were conducted when the natural recovery was about 30 years old, followed by natural recovery, with a stand age of about 50 years (moderate disturbance) (Xi et al., 2021). There were 46 species of woody plants in the plot, and the total number of plants was 3,065. In the sample community, the main species include *Pinus armandii*, *Quercus aliena* var. *acutiserrata*, and *Corylus heterophylla* (Supplementary Table 1).

(III) Once-cut forest (C): This forest was restored after comprehensive once-cutting, with a stand age of about 50 years (slight disturbance) (Xi et al., 2021). There were 57 species of woody plants in the plot, and the total number of plants was 4,302. The main species found in the site include *P. armandii*, *Quercus aliena* var. *acutiserrata*, and *Forsythia suspensa* (Supplementary Table 1).

(IV) Old-growth forest (D): In this forest, the individual density, mean diameter at breast height (DBH), and aboveground biomass of the woody plants were higher than the three aforementioned plant community types (Burrascano et al., 2013). The forest has been a natural forest for more than 100 years without human disturbance (undisturbance) (Xi et al., 2021). There were 52 species of woody plants in the plot, and the total number of plants was 2,490. The sample land was dominated by *Quercus aliena* var. *acutiserrata*, *Litsea tsinlingensis*, and *Sorbus hupehensis* species (Supplementary Table 1).

Based on the technical specification for plot construction and monitoring of the Tropical Forestry Research Center of the Smithsonian Institution (Condit, 1995; Richard, 1998), 1 hm^2^ sample plots(100 m × 100 m) were set in four kinds of sample forests, respectively. Each 100 m × 100 m plot was further divided into twenty-five 20 m × 20 m quadrats. All woody plants with DBH ≥ 1 cm in each 20 m × 20 m quadrat were tagged, identified, measured, and recorded (Supplementary Figure 1).

Soil samples (0–10 cm depth) were collected in August 2020. Three soil cores were randomly collected after litter removal within each 20 m × 20 m quadrat and mixed as a sample quadrat for soil physicochemical analyses, for a total of 100 soil samples in four forest succession. The soil samples were sieved using a 2 mm sieve to remove impurities (stones, plant roots, and litter). Each sample was divided into two parts: one part was stored at −80°C for DNA extraction, and the other part was stored at 4°C to measure soil physicochemical properties.

### Molecular Analyses for Soil Microbes

Total DNA was extracted from 0.5 g of soil samples using the FastDNA^®^ Spin Kit for Soil following the manufacturer’s instructions. Barcoded primer sets 515F/806R (Jing et al., 2015; Liu et al., 2021) targeting the V4 region of prokaryotic (bacteria) 16S rRNA genes and ITS1F/ITS2R (McGuire et al., 2013) targeting fungal ITS1 genes were used (Meyer and Kircher, 2010; Liu et al., 2021). The PCR mixtures contained 4 μL of 5 × TransStart FastPfu buffer, 0.8 μL of forward primer (5 μM), 2 μL of 2.5 mM dNTPs, 0.4 μL of TransStart FastPfu DNA polymerase, 0.8 μL of reverse primer (5 μM), 10 ng of template DNA, and 20 μL of ddH_2_O. The PCR reactions included initial denaturation at 95°C for 3 min, with 27 cycles of 95°C for 30 s, 55°C for 30 s, and 72°C for 30 s, and a final extension at 72°C for 10 min and then at 4°C. Each sample was amplified in triplicate. The PCR products from triplicate reactions per sample were pooled and gel-purified. Samples were then evaluated for quantity and quality *via* electrophoresis using 2% agarose gel (Lu et al., 2017). Purified amplicons were pooled in equimolar and paired-end sequences (2 × 300) on an Illumina MiSeq platform (Illumina, San Diego, United States) in accordance with the standard protocols by Majorbio Bio-Pharm Technology Co., Ltd. (Shanghai, China) (Liu et al., 2018; Sun et al., 2018; Zhang et al., 2018). The raw reads were deposited onto the NCBI Sequence Read Archive (SRA) database.

### Bioinformatic Analysis

The raw gene sequencing reads were quality filtered, demultiplexed by Trimmomatic, and merged by Fast Length Adjustment of Short reads (FLASH, v1.2.11) in accordance with the following criteria (Bao, 2000). Operational taxonomic units (OTUs) with 97% similarity cutoff (Kõljalg et al., 2013) were clustered using UPARSE (version 7.1)^1^, and chimeric sequences were identified and removed. We assessed the taxonomy of each representative OTU sequence using RDP Classifier^2^ against the 16S rRNA database and ITS database (e.g., Silva SSU128) with a confidence threshold of 0.7 (Magoc and Salzbera, 2011; Yu et al., 2019).

### Environmental Factor Analysis

We measured a range of environmental factors that influence the microbes. Topographical characteristics (slope [°], mean elevation [m], convex and concave [°], and aspect) were assessed during plot establishment (Harms et al., 2001; Valencia et al., 2004). The abundance and richness of woody plants (WA and WR) were assessed on the basis of the 20 m × 20 m quadrat of the four plots. Five edaphic variables were measured: pH, soil water content (SWC), soil organic matter (SOM), soil available phosphorus (P), and nitrogen (N). Soil pH was measured with a water: soil ratio of 2.5:1 (w/v) (Lu et al., 2019). SWC was measured by oven drying at 105°C to a constant mass (Zhang J. Q. et al., 2021). SOM content was measured using the K_2_CrO_7_ volumetric method and external heating (Nelson and Sommers, 1982; Yang et al., 2019). P was extracted with NaHCO_3_ and determined using the molybdenum antimony colorimetric method (Olsen et al., 1982; Sui et al., 2019). The content of N was measured by alkali solution diffusion (Bao, 2015).

### Statistical Analyses

Three microbial groups were constructed to understand species information across different succession: all species, core species, and dominant species. Core species accounted for 10% of all species (Jiao et al., 2020). Dominant species accounted for 0.5% of all species (Xiong et al., 2020). The core species and dominant species had an important ecological role in microbiome assembly and ecosystem functions (Banerjee et al., 2018; Delgado-Baquerizo et al., 2018).

A heat map was generated using R (version 4.0.3) to show the richness of bacterial and fungal communities at the OTU level. Venn diagrams showed the number of OTUs that were unique and shared among different succession. In addition, OTUs were visualized using the VennDiagram package in R (Chen and Boutros, 2011). In addition, the Kruskal–Wallis method was used to compare the richness of bacteria and fungi in four forest succession. Species accumulation curves of bacterial and fungal communities were drawn using the “specaccum” function in the vegan package of R (Li et al., 2018).

Network analysis was used to detect the specificity of microbes to different forests at the community level. The architecture of the plant community–microbe network was visualized on the basis of the ForceAtlas 2 node-layout algorithm using Gephi (Bastian et al., 2009). We evaluated the structure of the plant community–microbe network using the modularity index (Olesen et al., 2007). The torus-translation test was used to detect the distribution of microbes to different forests at the OTU level. The torus-translation test is the commonly used method to test the correlation between microbe and environment. It can exclude spatial autocorrelation to a certain extent, which makes the test more sensitive (Harms et al., 2001; Debski et al., 2002; DeWalt et al., 2006; Gunatilleke et al., 2006; Yamada et al., 2006; Comita et al., 2007). Further details on this method are provided by Harms et al. (2001). In the present study, four 1 hm^2^ plots during the secondary forest succession were selected as four microhabitats. We removed OTUs with a relative abundance of < 0.01% to reduce rare OTUs in the community (Jiao et al., 2020). A total of 14,224 bacterial OTUs and 5,410 fungal OTUs were used for torus-translation analysis. We analyzed the correlation among species, core species, and dominant species in four forest succession (positive or negative correlations, *P* ≤ 0.05).

Redundancy analysis (RDA) was used to map the effects of environmental factors (pH, SWC, P, SOM, N, WA, WR, aspect, slope, mean elevation, and convex) among the four forest succession on the measured soil bacterial and fungal communities using the “vegan” package (Gao et al., 2021). Hellinger transformation was performed before RDA linear ordering of response variables. In addition, the Monte Carlo permutation test was performed on the basis of 999 permutations to analyze whether the model reached a significant level (*P* < 0.05) (Chen et al., 2020). The “rdacca.hp” package was used to quantify the relative importance of each environmental factor independently explaining variations in soil bacterial and fungal communities and the contribution of a single explanatory variable (Lai et al., 2022). All methods were used for analysis of all species, core species, and dominant species. All statistical analyses were performed using R software 4.0.3 unless otherwise indicated.

## Results

### Species Diversity During Secondary Succession in a Temperate Mountain Forest

**FIGURE 1:**
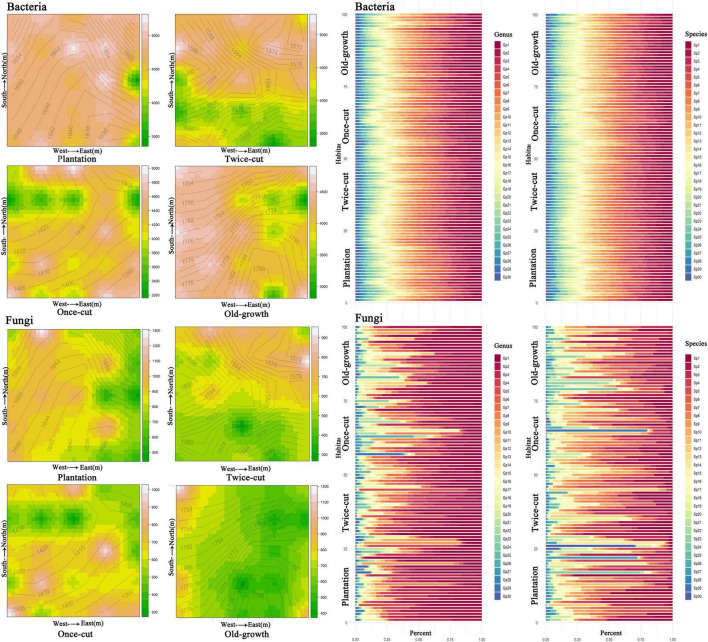
Spatial distribution and composition of bacterial and fungal species diversity in the four forest succession in the sample plot. The species accumulation diagrams show the composition of bacteria and fungi at the genus and species levels, respectively. The top 30 genera and species were selected for abundance. The abscissa is the proportion of species in the sample, and the ordinate is the plot. Different colored columns represent different species, and the length of the columns represents the proportion of the size of the species. The abbreviations of species are shown in Supplementary Table 4.

Combined with the topographic map of the sample site, the spatial distribution of bacterial and fungal OTU diversity in the four forest succession was not uniform, and evident spatial heterogeneity was observed (Figure 1 and Supplementary Figure 2). The percentage stacking diagrams showed that there were differences in the abundance of dominant species of fungi at the genus and species levels. On the contrary, the abundance of dominant species of bacteria was similar at the genus and species level (Figure 1).

**FIGURE 2:**
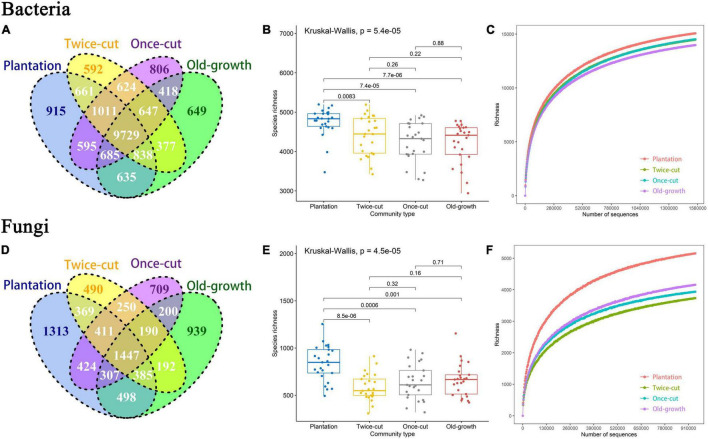
Distribution differences of bacterial and fungal OTU diversity in different forest succession. **(A,D)** Are the number of OTU of bacteria and fungi in different forest succession. **(B,E)** Are the OTU richness of bacteria and fungi in the four forest succession. The black lines obtained by the Kruskal-Wallis method indicate significant differences. **(C,F)** Are the rarefaction curve of bacteria and fungi in the four forest succession at the OTU level. Different colored curves indicated different types of succession (*P* ≤ 0.05 was the significance level).

The results of Venn diagram showed that the OTU composition of bacteria and fungi was different during secondary succession in a temperate mountain forest (Figures 2A,D and Supplementary Figures 3A,D,G,J). A maximum number of OTUs were found in the plantation forest. Four forest succession had 2,962 unique bacterial OTUs and 9,729 shared OTUs, as well as 3,451 unique fungal OTUs and 1,447 shared OTUs (Figures 2A,D).

The Kruskal–Wallis test results showed significant differences in the OTU richness of bacteria and fungi in the four forest succession (Figures 2B,E and Supplementary Figures 3B,E,H,K). The rarefaction curve tended to flatten as the number of measured sequences increased. This result indicated that the sample data obtained by the experiment reflected the composition of bacterial and fungal communities in the studied soils (Figures 2C,F and Supplementary Figures 3C,F,I,L).

### Spatial Distribution of Fungi and Bacteria Among the Four Forest Succession

**FIGURE 3:**
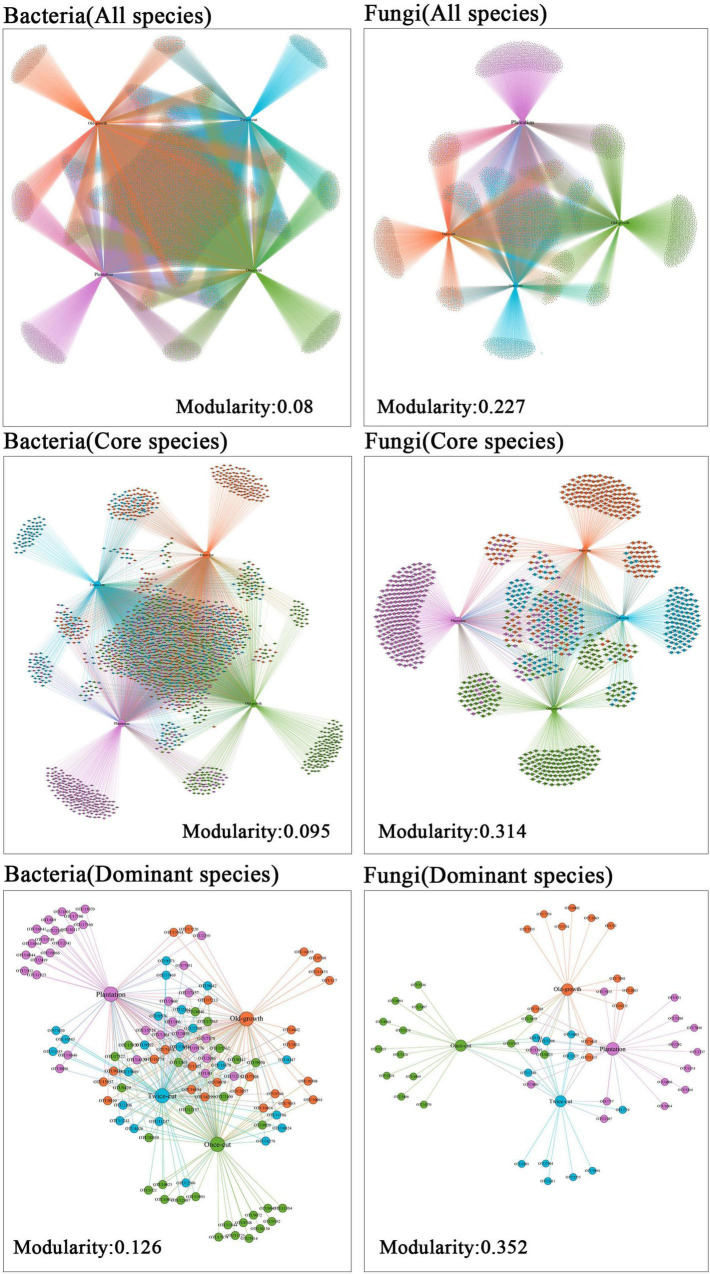
Network analysis of dominant OTU of bacteria and fungi in the four forest succession. The size of the node indicates the richness of the species. The colors of node indicate the distribution of OTUs in different succession.

The network of associations between microbe and plant communities was highly asymmetric in species abundance (Figure 3). For all species of bacteria, the modularity index was 0.080 (core: 0.095; dominant: 0.126). For all species of fungi, the modularity index was 0.227 (core: 0.314; dominant: 0.352; Figure 3). The results of network analysis showed that the modular index of fungi was higher than that of bacteria. The results showed that fungi had higher specificity than bacteria during secondary succession in a temperate mountain forest.

**FIGURE 4:**
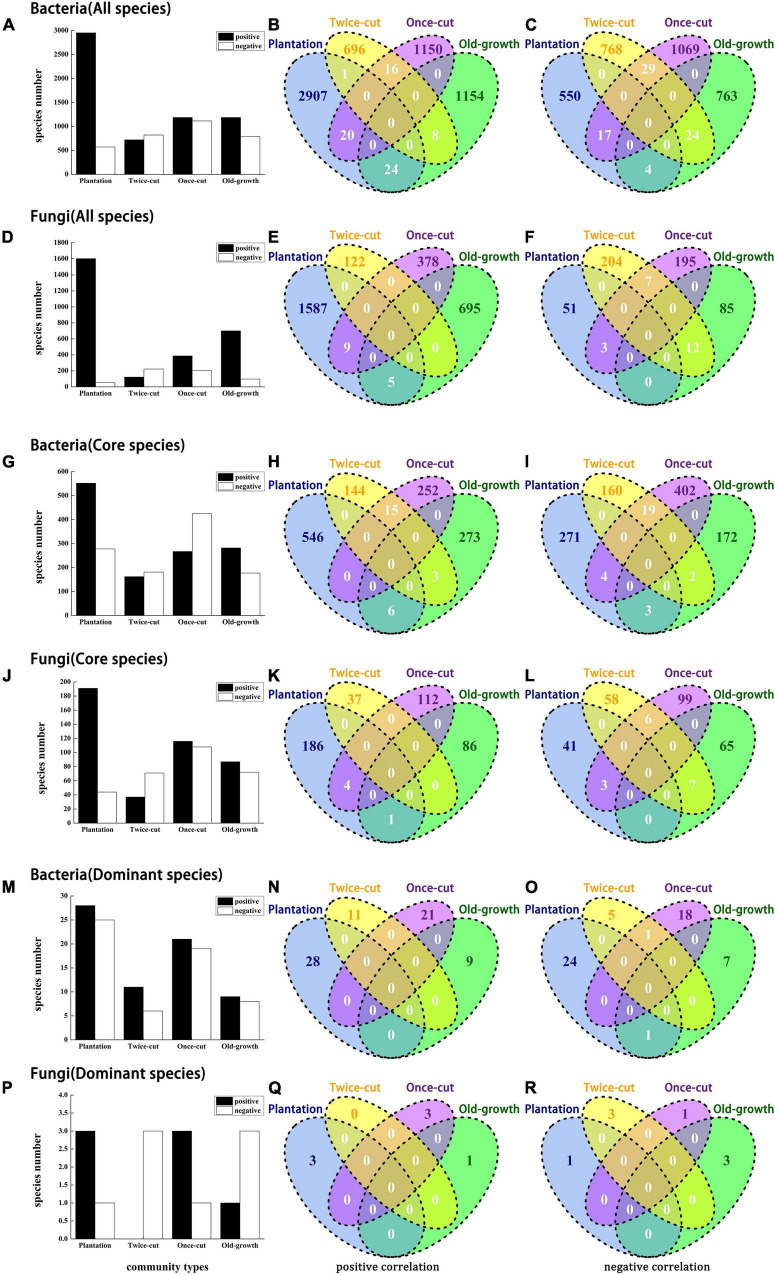
Bars and Venn diagrams of bacterial and fungal distribution at the OTU level. The bar diagrams shows the number of bacteria and fungi associated with the four forest succession. Venn diagrams show the number of species with significant positive and negative correlations between bacteria and fungi in the four forest succession. The association between microbes and plant community was tested by torus-translation random test (Torus-translation test, *P* ≤ 0.05 significance level).

A total of 14,224 bacteria and 5,410 fungi were associated with four forest succession. At *P* < 0.05, 49.95% (7105/14224) of bacteria and 54.47% (2947/5410) of fungi were associated with at least one plant community. Most OTUs were positively correlated with plantation forest, with 41.55% (2952/7105) bacteria and 54.33% (1601/2947) fungi (Figures 4A–F). The distribution of significantly correlated OTUs varied greatly among plant communities (Figures 4A,D,G,J,M,P). Torus translation showed that 23.18% (4553/19634) of the OTUs were distributed in plantation forest, whereas only 4.29% (843/19634), 8.01% (1573/19634), and 9.61% (1886/19634) of the OTUs were distributed in twice-cut forest, once-cut forest, and old-growth forest, respectively. No OTUs were positively or negatively correlated with the four forest succession (Figures 4B,C,E,F,H,I,K,L,N,O,Q,R). Supplementary Tables 2, 3 show the detailed associations between microbe and succession.

### Relationship Between Microbe and Environment Among the Four Forest Succession

**FIGURE 5:**
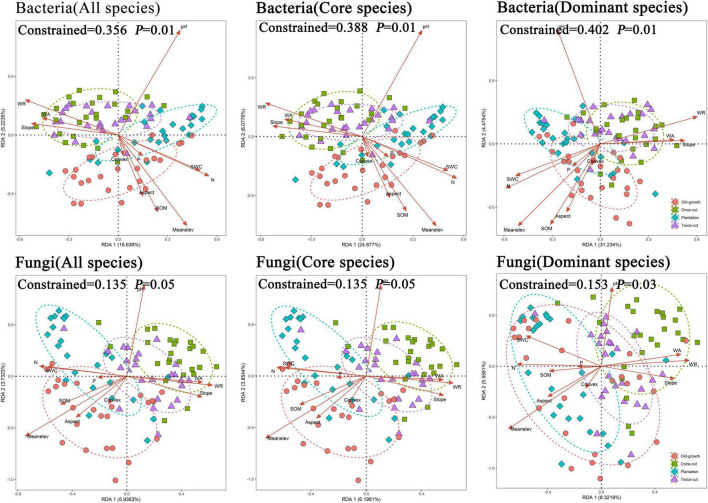
Redundancy analysis (RDA) plot showing the relationship between soil microbes and environmental factors in the experiment. Different colored dots indicate different types of succession. The ellipse has a 95% confidence interval. Environmental factors are indicated by an open-headed red arrow pointing in the direction of increasing values. pH: soil pH, SWC: soil water content, P: soil available phosphorus, SOM: soil organic matter, N: soil alkali-hydrolyzed nitrogen, Meanelev: mean elevation, Convex: convex concave, WA: woody plant abundance, WR: woody plant abundance.

Based on RDA, environmental factors (topographical, plants, and soil) significantly affected the composition of soil bacterial and fungal communities (Table 1). The composition of the soil microbial community differed significantly in different succession processes. RDA showed that these variables explained a total of 35.6 and 13.5% of the variance in the composition of bacterial and fungal communities among different succession processes, respectively. Among the eleven environmental factors, mean elevation and pH had the greatest effect on soil bacteria and fungi. Core and dominant species also showed similar trends to all species (Figure 5).

**TABLE 1:** Redundancy analysis (RDA) of effects of different environmental factors on soil bacteria and fungi.

	Bacteria			Fungi		
		
	All species	Core species	Dominant species	All species	Core species	Dominant species
pH	0.01**	0.01**	0.01**	0.01**	0.01**	0.01**
SWC	0.01**	0.01**	0.01**	0.01**	0.01**	0.03*
P	0.27	0.23	0.20	0.12	0.22	0.62
SOM	0.01**	0.01**	0.01**	0.01**	0.01**	0.34
N	0.01**	0.01**	0.01**	0.01**	0.01**	0.68
Aspect	0.01**	0.01**	0.01**	0.01**	0.01**	0.37
Slope	0.01**	0.01**	0.01**	0.01**	0.01**	0.48
Meanelev	0.01**	0.01**	0.01**	0.01**	0.01**	0.03*
Convex	0.34	0.34	0.43	0.14	0.09	0.27
WA	0.01**	0.01**	0.01**	0.01**	0.01**	0.68
WR	0.01**	0.01**	0.01**	0.01**	0.01**	0.72

“**”means more significant difference P < 0.01; “*”means difference P < 0.05.

## Discussion

### Spatial Distribution of Soil Microbes at the Community Level

Habitat differentiation is an important driving force for maintaining species diversity in forest ecosystems (Ye, 2000). Along with forest succession, soil structure coevolved with plant community change (Liu et al., 2021). In this study, the characteristics of bacteria and fungi assemblages differed during secondary succession in a temperate mountain forest. This result may indicate that ecological specialization plays an important role in the distribution of soil microbes during secondary succession in a temperate mountain forest.

In this study, the organization of the links in the microbe–succession network showed more specialization and unevenness. The characteristic network structure of the soil microbe–succession network in the forest ecosystem may be determined by the biological environment (e.g., woody plant composition) (Ferrer and Gilbert, 2003) and abiotic environment (e.g., soil physicochemical properties and light availability under canopy) (Chang and Miles, 2004; Barbier et al., 2008; Nakamura et al., 2017). Plant communities can affect microbial distribution through direct host–microbial interactions and rhizosphere effects (Martinez-Garcia et al., 2015) and indirect mediation of soil physicochemical properties (Zak et al., 2003). Plant communities primarily affect the structure and composition of underground soil microbial communities through litters and root secretion (Sasse et al., 2018; Shao et al., 2019). In this study, the composition and structure of plant species varied greatly as forest succession progressed. Great differences in light availability and soil physical and chemical properties under the canopy were also observed with the progress of forest succession. These factors may indicate the modular distribution of soil microbes in this study. Hence, the distribution of soil microbes during secondary succession in a temperate mountain forest is not random, but rather it is specialized.

### Spatial Distribution of Soil Microbes at the Operational Taxonomic Units Level

Our results showed that different soil microbes had different plant community preferences with long-term temperate forest recovery. In addition, the composition of soil bacterial and fungal communities and the relative abundance of dominant species changed significantly during the secondary succession in the studied temperate forest (Figure 1). These findings are consistent with previous studies across different forest ecosystems (Fichtner et al., 2014; Gao et al., 2015; Lee et al., 2017; Bonner et al., 2020). The results of torus translation analyses (Figure 4) provided further evidence that the different bacterial (49.95%) and fungal (54.47%) OTUs were associated with a specific forest community in long-term temperate forest recovery.

Our analyses revealed that soil microbes preferred to be distributed in plantation forest. Despite the potential influence of unmeasured environmental variables (Anderson, 2011), the results showed that heterogeneous environmental selection influenced the distribution of microbial communities in the early forest succession (Ferrenberg et al., 2013). In particular, environmental variables such as plant composition and inorganic nitrogen in forests might serve as strong filters, causing different microbial communities to be activated by different microbial seed banks that may exist at the sampling site (Lennon and Jones, 2011). Moreover, relatively few microbes were distributed in the twice-cut and once-cut forests. Different components of litters in different forest types could affect their decomposition rate, resulting in differences in soil nutrients and properties, thereby affecting the distribution of microbes (Sun, 2019). Our study demonstrates the importance of forest partitioning during secondary succession in maintaining local diversity in soil microbial communities.

### Relationship Between Soil Microbes and Environment

The changes in the structure of soil microbial community in a forest were significantly correlated with numerous environment variables, including soil pH and content of soil N and C (Banning et al., 2011; Qu et al., 2020). Different from previous studies, this study considered the effects of soil physicochemical factors, topography, and woody plants as environmental factors on soil microbial community structure, which can explain the joint effect of multiple factors on soil microbial distribution. Our study found that differences in succession processes could affect the relationship between soil microbes and the environment. This finding may be related to changes in plant attributes. Our results in temperate mountain forests (Figure 5) indicated that the influence of soil microbial distribution might vary with plant richness and abundance. Plant richness and abundance could reflect environmental heterogeneity across successional stages through diversification of available resources for soil microbes and feedbacks between plants and microbes (Reynolds et al., 2003; Wardle et al., 2004).

At different successional stages, the role of soil physicochemical properties can explain the change in the composition of soil microbial communities (Sterkenburg et al., 2015; Yeoh et al., 2017; Chai et al., 2019; Qiang et al., 2021). In this study, soil pH is a significant factor associated with variations in soil bacterial and fungal communities (Figure 5), which is consistent with many studies on succession (Banning et al., 2011; Montagna et al., 2018; Qiang et al., 2021). Soil pH might affect microbial distribution through selective pressure on soil microbial fitness and survival in acidic soil conditions of the studied forests (Tripathi et al., 2018). Similarly, the importance of inorganic nitrogen to microbial distribution may be related to microbial fitness at different concentrations of inorganic nitrogen (Verhamme et al., 2011; Fuchsman et al., 2019). With regard to the effects of plant communities, the variation in plant composition across successional stages reflects not only the changes in the relative abundance of plant species but also environmental heterogeneity. High plant richness promotes environmental heterogeneity through diversity of resources available to soil microbes and feedback between plants and microorganisms (Reynolds et al., 2003; Wardle et al., 2004; Liu et al., 2021). Plant communities at different stages of succession are affected by litter and root exudates (Reynolds et al., 2003; Wardle et al., 2004; Shao et al., 2019) and plant–microbial interactions (Martinez-Garcia et al., 2015), and understory microclimate affects the distribution of soil microbes (Maestre et al., 2009). Topography is an important environmental factor that reflects the soil environment, humidity, and temperature to a certain extent (Wangda and Ohsawa, 2006; Lan et al., 2011; Lei, 2019). Topography affects the spatial distribution of soil physicochemical properties through the redistribution of light, heat, and water resources (Garcia-Pausas et al., 2007), resulting in spatial differences in soil physicochemical properties (Wang et al., 2013; Yang et al., 2015) and differences in soil water and nutrient conditions under different topographic conditions (Pennock, 2005; Wu, 2015). Thus, environmental factors reflect changes in selective pressure that operates on soil microbial communities.

### Distribution Differences of Soil Fungi and Bacteria

Above-ground and below-ground connections and interactions are important to the structure and function of ecosystems, and they may be the main drivers of soil microbial communities (Bardgett, 2018). Plants can also lead to distinct shifts in fungal and bacterial communities in response to forest community succession (Chai et al., 2019). In our study, the contribution of vegetation community to microbial community change is as important as that of soil physicochemical properties and topography (Figure 5 and Table 1). Plants can attract specific rhizosphere microbes through species-specific root exudates in soil (Huang et al., 2014). In addition, soil bacterial and fungal communities change with plant traits, reflecting plant productivity (Sayer et al., 2017). In the mutually beneficial feedback circle, soil microbes can regulate the plant soil environment by regulating nitrogen fixation and nutrient conversion (Araya et al., 2017).

Compared with that of soil bacteria, the distribution of soil fungi in a temperate mountain forest showed higher specialization. In addition, compared with bacteria, more fungi exhibited distinct specific preferences in forest ecosystems in our study. This finding could be due to community assembly differences between bacteria and fungi. Previous studies considered light as the major driver for fungi and soil physicochemical factors as the major drivers for bacteria (Chen et al., 2018a; Deng J. et al., 2018). Compared with the soil microhabitat, forest gaps have changed understory light availability (Song et al., 2011). Moreover, forest canopy can be an influencing factor of the distribution of fungi (Nakamura et al., 2017). Light availability differences were distinct in the temperate deciduous broad-leaved forest (Hou and Hou, 1983). Fungi are more sensitive to changes in light than bacteria (Chang and Miles, 2004). They are also more closely related to plants than bacteria (Liu et al., 2021). Abundant ectomycorrhizal trees (e.g., *L. gmelinii* and *P. armandii*) could develop strong biotic interactions with ectomycorrhizal fungi (Su et al., 1992). Therefore, more fungi exhibited distinct distribution preferences than bacteria.

We analyzed the response of the soil microbial community to forest succession. From the coniferous forest at the early stage of succession, to the mixed coniferous and broadleaved forest at the middle stage, to the broadleaf forest at the last stage, the microbial population in the coniferous forest at the early stage of succession was significantly higher than that in other habitats. Studies have shown that litters in coniferous forests are more acidic than those in broadleaf forests (Augusto et al., 2002). Soil bacteria are more sensitive to soil pH than fungi (Lauber et al., 2009; Rousk et al., 2010; Zhalnina et al., 2015). Low pH inhibits enzyme and metabolic activities of bacteria, which is not conducive to bacterial growth (Beales, 2004). High pH can promote bacterial diversity by releasing dissolved organic matter (Curtin et al., 2016). The variation trend of bacteria and fungi species in temperate deciduous broad-leaved forests was basically the same in different succession processes. The number of OTUs in the plantation forest was the highest, and the number of OTUs in the twice-cut forest was the lowest, indicating rapid changes in bacterial and fungal communities after the succession was initiated. Previous studies have shown that soil bacterial communities respond more quickly to disturbances than soil fungi (Deng J. J. et al., 2018; Ren et al., 2018). It may be that disturbances increase the similarity among biotic communities (Petsch, 2016). In addition, Wang et al. (2020) found that the presence of litter during middle and late succession resulted in similar microbial community structures among different tree species. The reasons behind these results clarify further investigation.

## Conclusion and Implications

In this study, the distribution pattern of most soil microbes in a temperate mountain forest was not random, but it was specialized. Different microbes showed different distribution preferences. Fungal species showed higher specialization than bacterial species in forest community succession. Microbe–environment relations varied during secondary succession in a temperate mountain forest. These findings indicated that ecological specialization was important for microbial diversity during secondary succession in a temperate mountain forest.

Our findings provided comprehensive understanding of how complex soil microbial communities respond to changes in forest community succession. The distribution preferences of soil microbes were important for maintaining soil bacterial and fungal diversity. Based on the results of this study, the growth preferences of soil microbes should be fully considered in the subsequent forest ecosystem protection based on different environmental conditions. Fungi were more sensitive than bacteria during secondary succession in a temperate mountain forest. Changes in forest community succession could have important effects on soil fungal communities by potentially influencing the stability and health of forest ecosystems.

## Data Availability Statement

The datasets presented in this study can be found in online repositories. The names of the repository/repositories and accession number(s) can be found below: https://www.ncbi.nlm.nih.gov/ and http://www.ncbi.nlm.nih.gov/bioproject/806647.

## Author Contributions

PL and JX designed the study and analyzed the data. JZ, SW, PZ, and SD performed the field experiments and conducted the fieldwork. JX, SW, and WC conducted the laboratory work. JX wrote the manuscript. All authors read and approved the final manuscript.

## Conflict of Interest

The authors declare that the research was conducted in the absence of any commercial or financial relationships that could be construed as a potential conflict of interest.

## Publisher’s Note

All claims expressed in this article are solely those of the authors and do not necessarily represent those of their affiliated organizations, or those of the publisher, the editors and the reviewers. Any product that may be evaluated in this article, or claim that may be made by its manufacturer, is not guaranteed or endorsed by the publisher.

## Footnotes

1
http://drive5.com/uparse/

2
http://rdp.cme.msu.edu/
