# Supplementary material for: Changes in the Distribution Preference of Soil Microbial Communities During Secondary Succession in a Temperate Mountain Forest
Source: Front Microbiol. 2022 Jun 17;13:923346. doi: 10.3389/fmicb.2022.923346 (PMC9247583; doi:10.3389/fmicb.2022.923346)
Supplement: Supplementary file 1 [file Data_Sheet_1.docx]

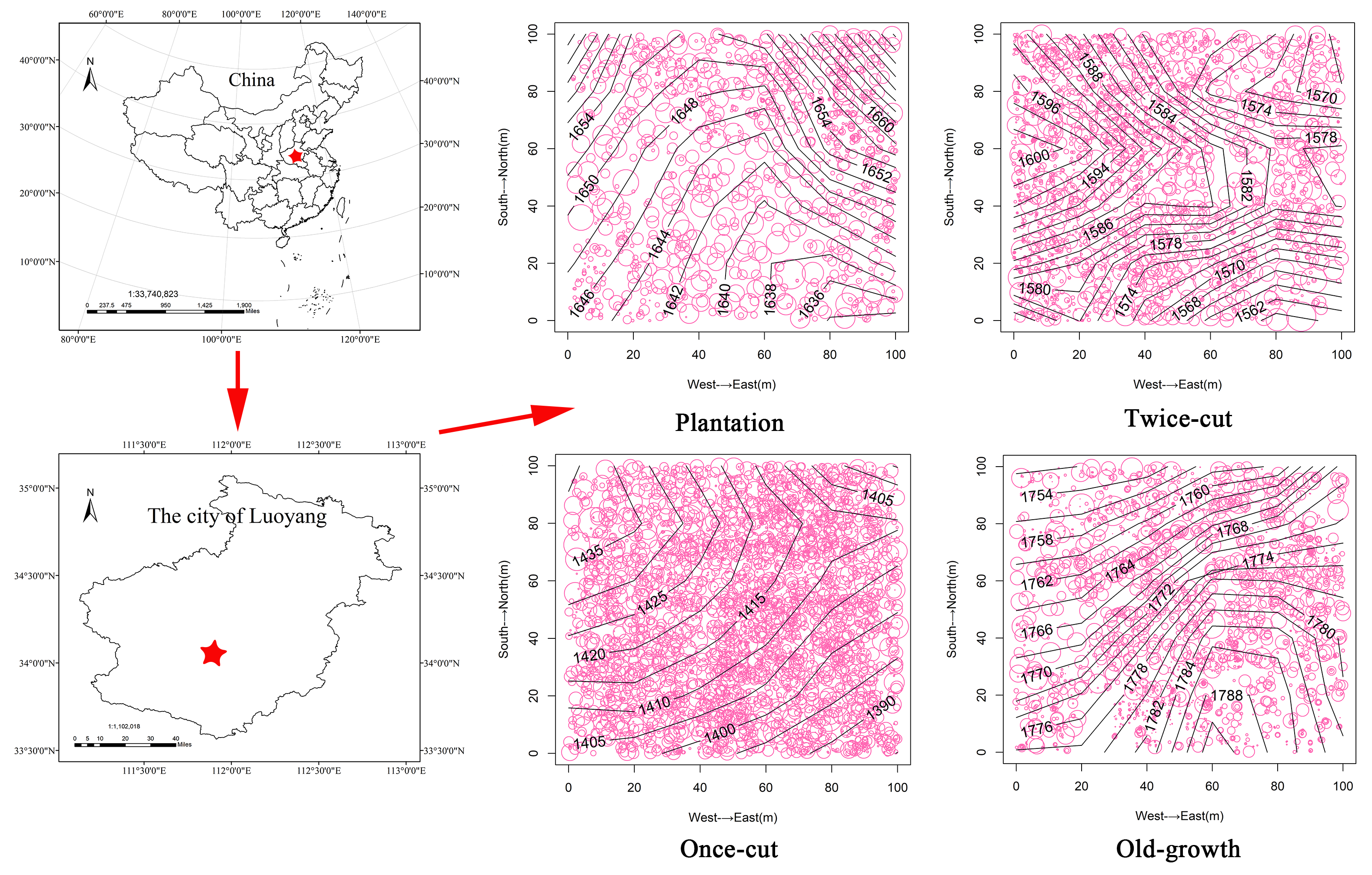


**Supplementary Figure 1.** Map of sampling plots and spatial distribution of species in the four forest succession.The red star indicates the location of the sample plots.The magenta circles represent woody plants.The solid black line is the contour map of the sample plots.


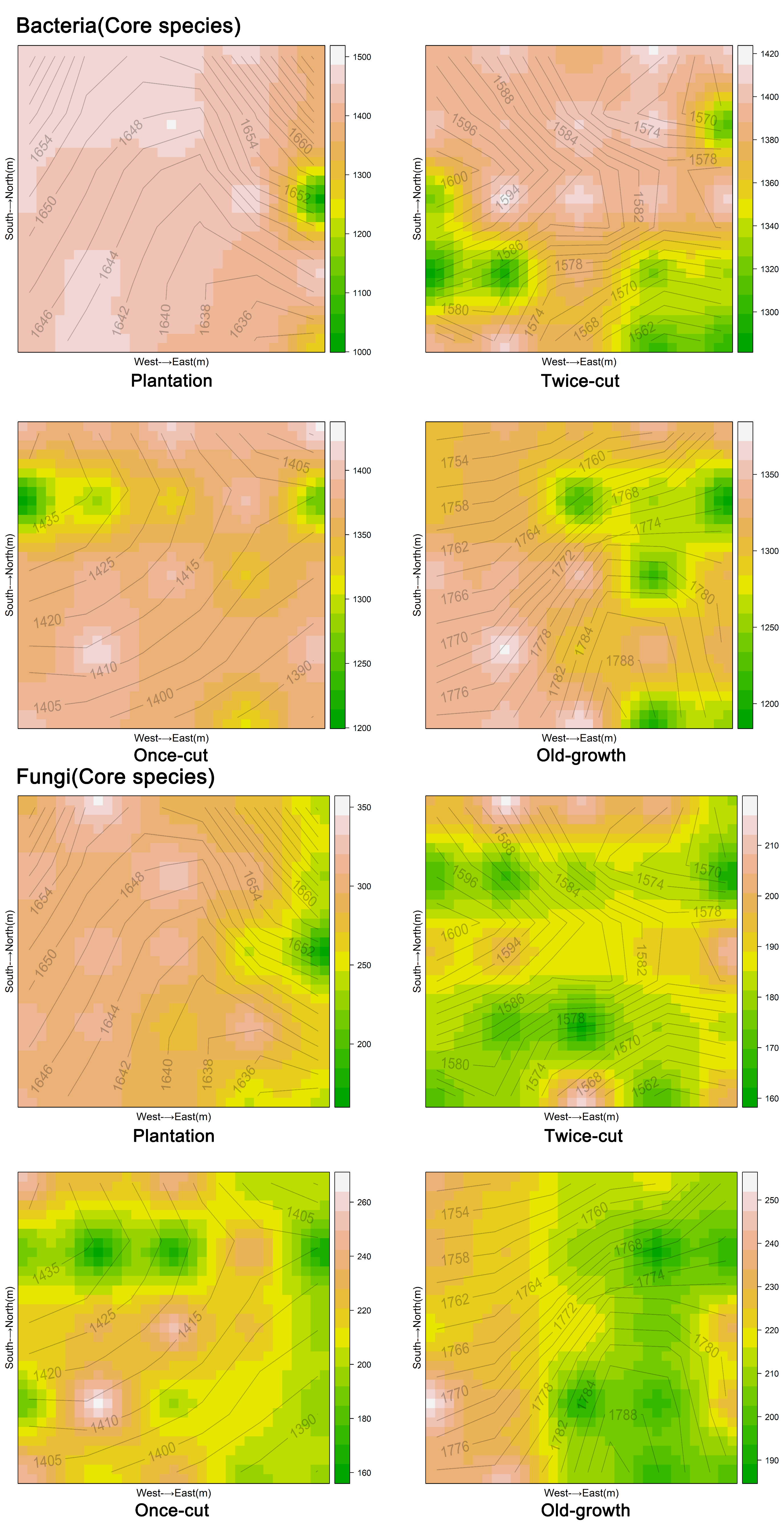


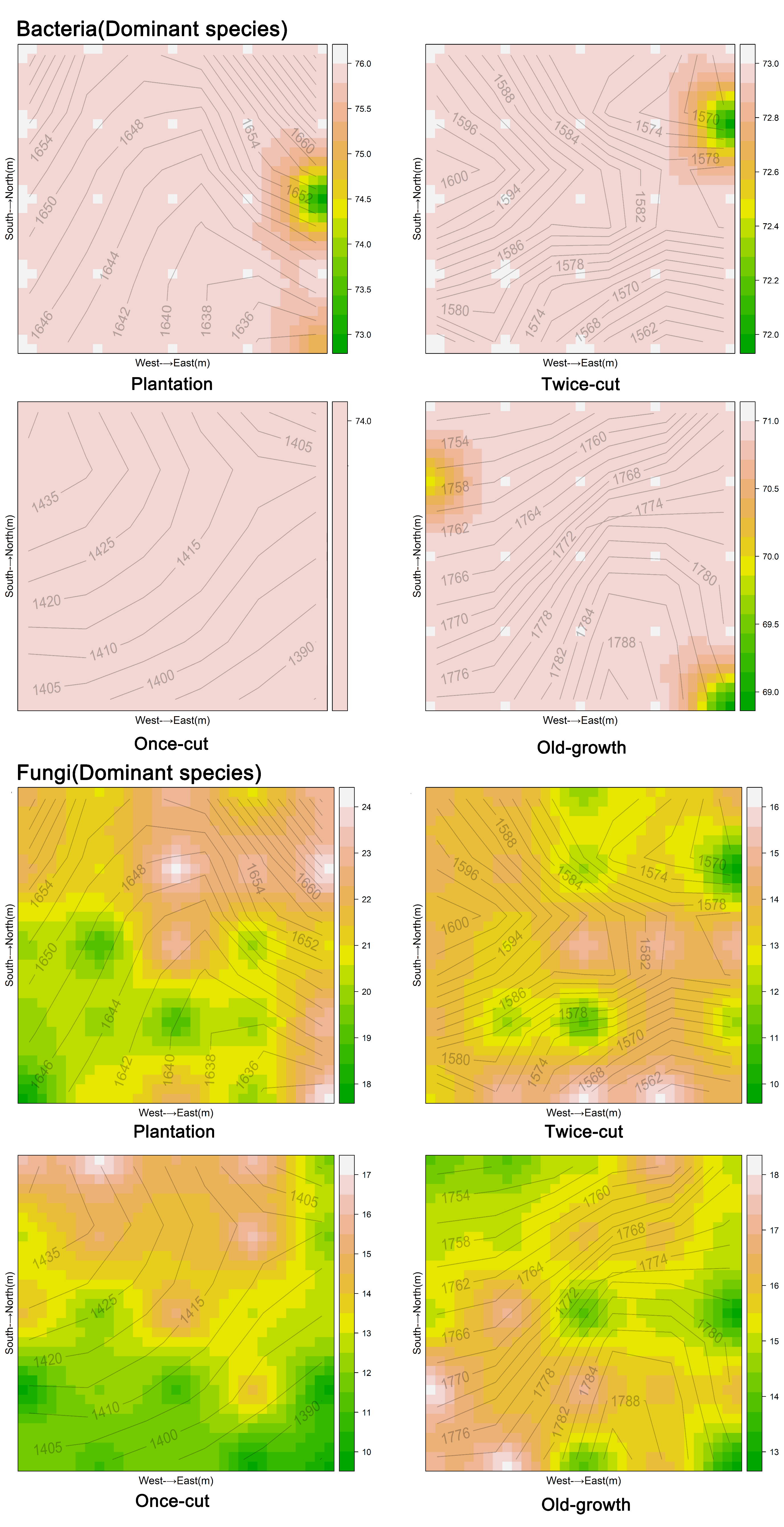


**Supplementary Figure 2.** Spatial distribution of core and dominant species diversity of bacteria and fungi in the four forest succession in the sample plots.





**Supplementary Figure 3.** Distribution differences of core and dominant species diversity of bacteria and fungi in different succession. (A), (D), (G) and (J) are the number of OTU of core and dominant species in different succession. (B) , (E), (H) and (K) are the OTU richness of core and dominant species in the four forest succession. The black lines obtained by the Kruskal-Wallis method indicate significant differences. (C), (F), (I) and (L) are the rarefaction curve of core and dominant species in the four forest succession at the OTU level. Different colored curves indicated different types of plant community (*P* ≤0.05 was the significance level).

**Supplementary Table 1.** Woody plant species in four forest succession.

| Species | Community | Species | Community |
| --- | --- | --- | --- |
| *Acer davidii* | Plantation | *Carpinus viminea* | Once-cut |
| *Acer davidii* subsp. *grosseri* | Plantation | *Celastrus orbiculatus* | Once-cut |
| *Acer pictum* subsp. *mono* | Plantation | *Celtis koraiensis* | Once-cut |
| *Acer truncatum* | Plantation | *Cerasus clarofolia* | Once-cut |
| *Betula chinensis* | Plantation | *Cerasus serrulata* | Once-cut |
| *Betula platyphylla* | Plantation | *Cornus controversa* | Once-cut |
| *Carpinus cordata* | Plantation | *Cornus hemsleyi* | Once-cut |
| *Carpinus turczaninowii* | Plantation | *Cornus kousa* subsp*. chinensis* | Once-cut |
| *Celastrus orbiculatus* | Plantation | *Cornus macrophylla* | Once-cut |
| *Cerasus clarofolia* | Plantation | *Cornus walteri* | Once-cut |
| *Cerasus serrulata* | Plantation | *Corylus chinensis* | Once-cut |
| *Cornus kousa* subsp. *chinensis* | Plantation | *Corylus heterophylla* | Once-cut |
| *Cornus macrophylla* | Plantation | *Cotoneaster acutifolius* | Once-cut |
| *Cornus walteri* | Plantation | *Crataegus wilsonii* | Once-cut |
| *Corylus heterophylla* | Plantation | *Euonymus phellomanus* | Once-cut |
| *Crataegus hupehensis* | Plantation | *Euptelea pleiosperma* | Once-cut |
| *Crataegus wilsonii* | Plantation | *Forsythia suspensa* | Once-cut |
| *Juglans cathayensis* | Plantation | *Fraxinus bungeana* | Once-cut |
| *Larix gmelinii* | Plantation | *Fraxinus chinensis* | Once-cut |
| *Lindera obtusiloba Blume* | Plantation | *Fraxinus paxiana* | Once-cut |
| *Litsea tsinlingensis* | Plantation | *Juglans cathayensis* | Once-cut |
| *Lonicera tatarinowii* | Plantation | *Larix gmelinii* | Once-cut |
| *Malus honanensis* | Plantation | *Lindera obtusiloba Blume* | Once-cut |
| *Malus hupehensis* | Plantation | *Litsea tsinlingensis* | Once-cut |
| *Meliosma flexuosa* | Plantation | *Lonicera microphylla* | Once-cut |
| *Meliosma veitchiorum* | Plantation | *Maackia hwashanensis* | Once-cut |
| *Padus buergeriana* | Plantation | *Malus honanensis* | Once-cut |
| *Padus obtusata* | Plantation | *Malus hupehensis* | Once-cut |
| *Pinus armandii Franch* | Plantation | *Meliosma flexuosa* | Once-cut |
| *Pinus tabuliformis Carrière* | Plantation | *Meliosma veitchiorum* | Once-cut |
| *Quercus aliena* var. *acutiserrata* | Plantation | *Ostrya japonica* | Once-cut |
| *Rosa bella* | Plantation | *Philadelphus incanus* | Once-cut |
| *Salix tangii* | Plantation | *Pinus armandii Franch* | Once-cut |
| *Sorbaria sorbifolia* | Plantation | *Pinus tabuliformis Carrière* | Once-cut |
| *Sorbus alnifolia* | Plantation | *Quercus aliena* var. *acutiserrata* | Once-cut |
| *Styrax obassis* | Plantation | *Quercus serrata* var. *brevipetiolata* | Once-cut |
| *Symplocos paniculata* | Plantation | *Quercus variabilis* | Once-cut |
| *Tilia japonica* | Plantation | *Rhododendron micranthum* | Once-cut |
| *Tilia paucicostata* | Plantation | *Sorbus alnifolia* | Once-cut |
| *Toxicodendron vernicifluum* | Plantation | *Styrax obassis* | Once-cut |
| *Viburnum lobophyllum* | Plantation | *Symplocos paniculata* | Once-cut |
| *Yulania denudata* | Plantation | *Tilia japonica* | Once-cut |
| *Abelia uniflora* | Twice-cut | *Tilia paucicostata* | Once-cut |
| *Acer davidii* | Twice-cut | *Toxicodendron vernicifluum* | Once-cut |
| *Acer truncatum* | Twice-cut | *Ulmus davidiana* | Once-cut |
| *Berberis circumserrata* | Twice-cut | *Viburnum betulifolium* | Once-cut |
| *Betula chinensis* | Twice-cut | *Yulania denudata* | Once-cut |
| *Betula luminifera* | Twice-cut | *Abelia biflora* | Old-growth |
| *Betula platyphylla* | Twice-cut | *Acer davidii* | Old-growth |
| *Carpinus cordata* | Twice-cut | *Acer pictum* subsp. *mono* | Old-growth |
| *Carpinus turczaninowii* | Twice-cut | *Acer truncatum* | Old-growth |
| *Celastrus orbiculatus* | Twice-cut | *Ailanthus altissima* | Old-growth |
| *Cerasus clarofolia* | Twice-cut | *Alangium platanifolium* | Old-growth |
| *Cornus controversa* | Twice-cut | *Berberis circumserrata* | Old-growth |
| *Cornus kousa* subsp. *chinensis* | Twice-cut | *Betula albosinensis* | Old-growth |
| *Cornus macrophylla* | Twice-cut | *Betula luminifera* | Old-growth |
| *Cornus schindleri* subsp. *poliophylla* | Twice-cut | *Carpinus cordata* | Old-growth |
| *Corylus chinensis* | Twice-cut | *Carpinus turczaninowii* | Old-growth |
| *Corylus heterophylla* | Twice-cut | *Celastrus orbiculatus* | Old-growth |
| *Cotoneaster acutifolius* | Twice-cut | *Cerasus clarofolia* | Old-growth |
| *Crataegus pinnatifida* | Twice-cut | *Clematis heracleifolia* | Old-growth |
| *Elaeagnus angustifolia* | Twice-cut | *Cornus macrophylla* | Old-growth |
| *Elaeagnus umbellata* | Twice-cut | *Corylus chinensis* | Old-growth |
| *Forsythia suspensa* | Twice-cut | *Corylus heterophylla* | Old-growth |
| *Juglans cathayensis* | Twice-cut | *Cotoneaster acutifolius* | Old-growth |
| *Lindera obtusiloba Blume* | Twice-cut | *Cotoneaster tenuipes* | Old-growth |
| *Litsea tsinlingensis* | Twice-cut | *Crataegus wilsonii* | Old-growth |
| *Malus honanensis* | Twice-cut | *Euonymus phellomanus* | Old-growth |
| *Meliosma flexuosa* | Twice-cut | *Euptelea pleiosperma* | Old-growth |
| *Meliosma veitchiorum* | Twice-cut | *Evodia daniellii* | Old-growth |
| *Philadelphus incanus* | Twice-cut | *Forsythia suspensa* | Old-growth |
| *Pinus armandii Franch* | Twice-cut | *Fraxinus chinensis* | Old-growth |
| *Pinus tabuliformis* | Twice-cut | *Fraxinus chinensis* subsp. *Rhynchophylla* | Old-growth |
| *Populus davidiana* | Twice-cut | *Juglans cathayensis* | Old-growth |
| *Quercus aliena* var. *acutiserrata* | Twice-cut | *Lindera obtusiloba* | Old-growth |
| *Quercus serrata* var. *brevipetiolata* | Twice-cut | *Litsea tsinlingensis* | Old-growth |
| *Quercus variabilis* | Twice-cut | *Lonicera tatarinowii* | Old-growth |
| *Rhododendron micranthum* | Twice-cut | *Malus honanensis Rehder* | Old-growth |
| *Sorbaria sorbifolia* | Twice-cut | *Meliosma veitchiorum* | Old-growth |
| *Sorbus alnifolia* | Twice-cut | *Padus buergeriana* | Old-growth |
| *Sorbus hupehensis* | Twice-cut | *Padus velutina* | Old-growth |
| *Styrax obassis* | Twice-cut | *Philadelphus incanus* | Old-growth |
| *Symplocos paniculata* | Twice-cut | *Pinus armandii Franch* | Old-growth |
| *Tilia japonica* | Twice-cut | *Pinus tabuliformis* | Old-growth |
| *Tilia paucicostata* | Twice-cut | *Populus davidiana* | Old-growth |
| *Toxicodendron vernicifluum* | Twice-cut | *Populus simonii* | Old-growth |
| *Viburnum betulifolium* | Twice-cut | *Quercus aliena* var. *acutiserrata* | Old-growth |
| *Yulania denudata* | Twice-cut | *Quercus serrata* var. *brevipetiolata* | Old-growth |
| *Acer davidii* | Once-cut | *Rosa bella* | Old-growth |
| *Acer davidii* subsp*. grosseri* | Once-cut | *Salix chaenomeloides Kimura* | Old-growth |
| *Acer pictum* subsp. *mono* | Once-cut | *Salix shihtsuanensis* | Old-growth |
| *Acer truncatum* | Once-cut | *Sambucus williamsii* | Old-growth |
| *Aralia elata* | Once-cut | *Sorbus alnifolia* | Old-growth |
| *Betula luminifera* | Once-cut | *Sorbus hupehensis* | Old-growth |
| *Betula platyphylla* | Once-cut | *Symplocos paniculata* | Old-growth |
| *Carpinus cordata* | Once-cut | *Tilia japonica* | Old-growth |
| *Carpinus polyneura* | Once-cut | *Toxicodendron vernicifluum* | Old-growth |
| *Carpinus turczaninowii* | Once-cut | *Viburnum opulus* var*. sargentii* | Old-growth |

**Supplementary Table 2.** Significant associations of soil bacteria with the four forest succession (*P* ≤ 0.05 level of significance for torus-translation test). NA represents no significant correlation. (+) indicates positive correlation. (-) indicates negative correlation.

| Species | Succession | | | |
| --- | --- | --- | --- | --- |
|  | Plantation | Twice-cut | Once-cut | Old-growth |
| OTU106 | NA | NA | NA | + |
| OTU84 | - | NA | + | NA |
| OTU1304 | - | NA | + | NA |
| OTU2860 | NA | NA | - | NA |
| OTU9576 | - | + | NA | NA |
| OTU7178 | NA | - | NA | NA |
| OTU9592 | NA | NA | NA | + |
| OTU2748 | NA | - | - | NA |
| OTU4646 | NA | NA | NA | + |
| OTU19176 | + | NA | - | NA |
| OTU15957 | NA | NA | - | NA |
| OTU15058 | - | + | NA | NA |
| OTU15920 | NA | NA | + | NA |
| OTU2057 | - | NA | + | NA |
| OTU5164 | NA | - | + | NA |
| OTU6542 | - | NA | + | NA |
| OTU9078 | - | NA | NA | NA |
| OTU9429 | NA | + | NA | NA |
| OTU2364 | + | NA | - | NA |
| OTU14320 | - | NA | + | NA |
| OTU9642 | - | NA | + | NA |
| OTU15718 | + | NA | NA | NA |
| OTU17213 | - | NA | + | NA |
| OTU9810 | - | NA | + | NA |
| OTU9950 | - | NA | + | NA |
| OTU1355 | NA | NA | NA | NA |
| OTU9938 | - | NA | NA | NA |
| OTU16399 | - | + | NA | NA |
| OTU17457 | - | NA | + | NA |
| OTU12242 | NA | NA | NA | NA |
| OTU17563 | - | NA | + | NA |
| OTU17322 | NA | - | + | NA |
| OTU11247 | - | NA | + | NA |
| OTU7831 | + | NA | - | NA |
| OTU16779 | NA | NA | NA | - |
| OTU3859 | + | NA | - | NA |
| OTU15678 | NA | + | NA | NA |
| OTU15989 | NA | NA | NA | + |
| OTU10023 | - | + | + | NA |
| OTU1563 | + | NA | NA | NA |
| OTU10939 | - | NA | + | NA |
| OTU3109 | NA | NA | NA | NA |
| OTU10416 | - | + | NA | NA |
| OTU2086 | + | NA | NA | NA |
| OTU16694 | - | + | NA | NA |
| OTU4626 | NA | NA | NA | + |
| OTU11241 | NA | NA | NA | + |
| OTU2106 | + | - | NA | NA |
| OTU9820 | - | NA | + | NA |
| OTU15562 | NA | NA | NA | NA |
| OTU10824 | - | NA | NA | NA |
| OTU11786 | - | + | NA | NA |
| OTU15964 | + | NA | - | NA |
| OTU8571 | + | NA | - | NA |
| OTU7308 | NA | NA | NA | NA |
| OTU12157 | NA | NA | NA | NA |
| OTU9746 | - | + | NA | NA |
| OTU12487 | - | NA | + | NA |
| OTU18846 | NA | + | NA | - |
| OTU2259 | NA | NA | - | + |
| OTU4247 | - | + | NA | NA |
| OTU10308 | NA | NA | NA | NA |
| OTU11585 | NA | + | NA | NA |
| OTU17469 | NA | NA | - | + |
| OTU10413 | - | NA | + | NA |
| OTU7055 | - | NA | NA | NA |
| OTU18044 | + | NA | NA | NA |
| OTU10585 | NA | NA | NA | NA |
| OTU2811 | NA | NA | NA | NA |
| OTU16278 | - | NA | + | NA |
| OTU9965 | - | NA | + | NA |
| OTU9192 | NA | NA | + | NA |
| OTU11723 | NA | NA | + | NA |
| OTU17874 | - | NA | + | NA |
| OTU7820 | NA | NA | - | NA |
| OTU4442 | NA | NA | NA | NA |
| OTU8748 | - | NA | NA | NA |
| OTU10061 | NA | - | NA | NA |
| OTU13991 | - | + | + | NA |
| OTU2352 | + | NA | NA | NA |
| OTU11923 | + | NA | NA | NA |
| OTU3931 | NA | NA | NA | NA |
| OTU12506 | NA | NA | + | NA |
| OTU8896 | NA | NA | NA | NA |
| OTU9887 | - | NA | NA | NA |
| OTU17220 | NA | - | NA | NA |
| OTU13749 | + | - | NA | NA |
| OTU3173 | NA | NA | NA | + |
| OTU5321 | - | NA | + | - |
| OTU9788 | - | NA | NA | NA |
| OTU5895 | - | NA | NA | + |
| OTU9972 | NA | NA | + | NA |
| OTU13070 | + | NA | NA | NA |
| OTU15199 | - | + | NA | NA |
| OTU11384 | - | NA | + | NA |
| OTU17360 | + | NA | - | NA |
| OTU11292 | NA | + | - | NA |
| OTU14135 | - | + | NA | NA |
| OTU2419 | + | NA | - | NA |
| OTU3914 | - | NA | + | NA |
| OTU13325 | NA | NA | NA | + |
| OTU9544 | - | NA | NA | + |
| OTU11683 | NA | NA | + | NA |
| OTU10130 | - | NA | + | NA |
| OTU5294 | NA | NA | NA | NA |
| OTU17809 | - | NA | NA | NA |
| OTU19089 | + | NA | - | NA |
| OTU3026 | NA | - | NA | + |
| OTU4529 | - | NA | NA | NA |
| OTU9870 | NA | NA | + | NA |
| OTU14741 | NA | NA | + | NA |
| OTU17050 | + | NA | NA | NA |
| OTU15453 | NA | NA | NA | + |
| OTU9696 | - | NA | + | NA |
| OTU3543 | + | - | NA | NA |
| OTU12341 | + | - | - | NA |
| OTU9840 | NA | + | NA | NA |
| OTU16655 | NA | NA | - | + |
| OTU16941 | + | NA | - | NA |
| OTU4507 | NA | NA | NA | NA |
| OTU11844 | - | NA | + | NA |
| OTU17925 | NA | - | NA | + |
| OTU2095 | NA | + | NA | - |
| OTU16185 | + | NA | - | NA |
| OTU18870 | NA | NA | NA | + |
| OTU2670 | + | NA | NA | NA |
| OTU18492 | NA | NA | NA | + |
| OTU3836 | NA | NA | - | NA |
| OTU2482 | + | NA | - | NA |
| OTU9860 | NA | + | NA | - |
| OTU2229 | - | + | NA | NA |
| OTU19066 | + | NA | NA | NA |
| OTU9858 | NA | NA | NA | + |
| OTU10117 | + | NA | NA | NA |
| OTU17314 | + | - | NA | NA |
| OTU12106 | NA | - | - | NA |
| OTU9909 | - | NA | NA | NA |
| OTU9657 | - | NA | + | NA |
| OTU11659 | NA | NA | NA | + |
| OTU2332 | + | NA | - | NA |
| OTU10114 | NA | - | NA | + |
| OTU16864 | + | NA | - | NA |
| OTU17700 | + | NA | - | NA |
| OTU12563 | NA | NA | NA | - |
| OTU11093 | + | NA | NA | NA |
| OTU17781 | + | NA | - | NA |
| OTU5467 | NA | NA | NA | NA |
| OTU14733 | NA | NA | + | NA |
| OTU2993 | NA | NA | NA | NA |
| OTU9120 | NA | NA | NA | NA |
| OTU10556 | NA | NA | NA | NA |
| OTU3896 | NA | NA | + | NA |
| OTU3133 | - | NA | NA | + |
| OTU3342 | + | NA | NA | NA |
| OTU11549 | NA | - | NA | + |
| OTU1802 | + | NA | - | NA |
| OTU8706 | + | - | NA | NA |
| OTU14599 | + | NA | NA | NA |
| OTU17028 | NA | NA | NA | NA |
| OTU18474 | NA | NA | NA | NA |
| OTU17316 | NA | NA | NA | NA |
| OTU8391 | NA | NA | + | NA |
| OTU117 | NA | NA | - | + |
| OTU10147 | NA | NA | + | NA |
| OTU3575 | + | NA | - | NA |
| OTU15158 | + | NA | - | NA |
| OTU11156 | NA | NA | - | + |
| OTU6406 | NA | NA | NA | NA |
| OTU16936 | + | NA | - | NA |
| OTU10870 | - | NA | NA | NA |
| OTU8612 | - | NA | + | NA |
| OTU9718 | NA | NA | NA | NA |
| OTU18866 | NA | + | NA | NA |
| OTU2269 | NA | NA | NA | NA |
| OTU9070 | NA | - | NA | NA |
| OTU4672 | - | NA | + | NA |
| OTU8742 | NA | NA | + | NA |
| OTU9279 | + | NA | - | NA |
| OTU1096 | NA | NA | NA | NA |
| OTU7665 | NA | NA | NA | NA |
| OTU244 | NA | NA | NA | - |
| OTU10179 | - | + | NA | NA |
| OTU15023 | - | NA | - | + |
| OTU11428 | - | NA | + | NA |
| OTU4688 | - | NA | + | NA |
| OTU16882 | NA | NA | NA | NA |
| OTU18486 | NA | NA | - | + |
| OTU9559 | NA | NA | NA | NA |
| OTU889 | + | NA | - | NA |
| OTU8663 | - | NA | NA | NA |
| OTU2441 | + | NA | - | NA |
| OTU2918 | + | NA | NA | NA |
| OTU15103 | + | NA | NA | NA |
| OTU14503 | + | NA | NA | NA |
| OTU9547 | NA | + | NA | - |
| OTU16148 | NA | NA | NA | NA |
| OTU17612 | + | - | NA | NA |
| OTU12463 | - | + | NA | NA |
| OTU4707 | NA | NA | NA | NA |
| OTU16835 | NA | NA | - | + |
| OTU11323 | NA | NA | + | - |
| OTU9159 | NA | NA | NA | NA |
| OTU18864 | NA | NA | - | NA |
| OTU7045 | - | + | - | NA |
| OTU17819 | NA | NA | - | NA |
| OTU6177 | NA | - | NA | + |
| OTU3499 | NA | NA | NA | NA |
| OTU12566 | - | NA | + | NA |
| OTU16404 | + | NA | NA | NA |
| OTU15910 | + | NA | - | NA |
| OTU17981 | + | - | NA | NA |
| OTU178 | NA | NA | NA | NA |
| OTU4186 | - | NA | + | NA |
| OTU2405 | + | NA | - | NA |
| OTU17260 | + | NA | - | NA |
| OTU5451 | NA | NA | - | NA |
| OTU11092 | NA | NA | - | + |
| OTU11776 | NA | NA | NA | NA |
| OTU18482 | NA | NA | NA | + |
| OTU10850 | NA | NA | + | - |
| OTU11720 | + | NA | NA | NA |
| OTU18076 | NA | NA | NA | NA |
| OTU13455 | NA | NA | - | NA |
| OTU14730 | NA | NA | NA | NA |
| OTU18516 | NA | NA | - | NA |
| OTU10901 | NA | NA | + | NA |
| OTU11084 | + | - | NA | NA |
| OTU1278 | + | - | NA | NA |
| OTU8370 | NA | NA | NA | NA |
| OTU10757 | - | NA | + | NA |
| OTU4671 | NA | NA | + | - |
| OTU15480 | - | NA | + | NA |
| OTU18208 | NA | NA | NA | NA |
| OTU3584 | + | NA | - | NA |
| OTU10033 | - | NA | + | NA |
| OTU8659 | NA | NA | NA | NA |
| OTU11646 | NA | NA | NA | NA |
| OTU18243 | - | NA | + | NA |
| OTU14093 | - | NA | NA | NA |
| OTU16085 | NA | NA | NA | NA |
| OTU3325 | NA | NA | NA | NA |
| OTU18452 | - | NA | NA | NA |
| OTU18808 | NA | NA | - | + |
| OTU2781 | + | NA | NA | NA |
| OTU17119 | NA | - | NA | + |
| OTU3409 | + | NA | NA | NA |
| OTU18466 | NA | NA | NA | NA |
| OTU4516 | + | NA | NA | NA |
| OTU9251 | NA | NA | - | + |
| OTU6873 | NA | NA | NA | NA |
| OTU16257 | - | NA | + | NA |
| OTU13483 | NA | NA | NA | + |
| OTU16744 | NA | NA | NA | NA |
| OTU14676 | NA | NA | + | - |
| OTU18064 | - | NA | + | NA |
| OTU329 | NA | + | NA | NA |
| OTU14751 | - | NA | + | NA |
| OTU7250 | NA | NA | NA | NA |
| OTU17453 | - | NA | NA | NA |
| OTU17556 | NA | NA | NA | + |
| OTU10277 | NA | NA | - | NA |
| OTU13970 | NA | NA | + | NA |
| OTU10012 | NA | NA | NA | NA |
| OTU19095 | + | NA | - | NA |
| OTU16016 | NA | NA | NA | NA |
| OTU17689 | - | NA | NA | NA |
| OTU19203 | + | - | NA | NA |
| OTU9412 | NA | + | NA | - |
| OTU3948 | + | - | NA | NA |
| OTU17974 | + | NA | - | NA |
| OTU17319 | + | NA | NA | NA |
| OTU4200 | + | NA | NA | NA |
| OTU11624 | - | NA | NA | NA |
| OTU10667 | - | + | + | NA |
| OTU9542 | + | NA | NA | NA |
| OTU13829 | NA | NA | NA | NA |
| OTU5493 | - | NA | + | NA |
| OTU6891 | + | NA | NA | NA |
| OTU16271 | - | + | NA | NA |
| OTU12985 | NA | NA | NA | NA |
| OTU7295 | + | NA | - | + |
| OTU116 | NA | NA | - | NA |
| OTU12512 | NA | NA | NA | NA |
| OTU17816 | - | NA | + | NA |
| OTU17876 | + | NA | - | NA |
| OTU3986 | NA | + | NA | - |
| OTU14756 | - | + | NA | NA |
| OTU14743 | NA | NA | NA | NA |
| OTU3869 | NA | NA | - | + |
| OTU13082 | + | NA | - | NA |
| OTU15490 | NA | NA | + | - |
| OTU17827 | + | NA | - | NA |
| OTU10911 | NA | NA | NA | - |
| OTU18948 | NA | NA | NA | + |
| OTU10796 | - | NA | + | NA |
| OTU17812 | NA | NA | NA | NA |
| OTU12488 | - | NA | NA | NA |
| OTU16881 | NA | NA | + | - |
| OTU14973 | NA | NA | NA | NA |
| OTU16175 | - | NA | + | NA |
| OTU15679 | NA | NA | NA | + |
| OTU4178 | NA | NA | NA | NA |
| OTU8 | NA | NA | NA | NA |
| OTU12152 | NA | - | NA | NA |
| OTU13874 | + | NA | NA | NA |
| OTU634 | + | NA | NA | - |
| OTU3172 | NA | NA | NA | + |
| OTU17850 | + | NA | - | NA |
| OTU14605 | - | NA | NA | + |
| OTU11180 | - | NA | + | NA |
| OTU3935 | NA | NA | NA | NA |
| OTU15037 | + | NA | NA | NA |
| OTU16713 | NA | NA | NA | NA |
| OTU17747 | NA | NA | NA | + |
| OTU16861 | - | NA | NA | NA |
| OTU8404 | + | NA | NA | NA |
| OTU2633 | NA | - | NA | + |
| OTU2204 | + | NA | NA | NA |
| OTU14710 | NA | NA | NA | NA |
| OTU9003 | + | NA | NA | NA |
| OTU11550 | NA | + | - | NA |
| OTU14094 | + | - | NA | NA |
| OTU17214 | NA | + | - | NA |
| OTU17713 | - | + | NA | NA |
| OTU15293 | NA | NA | NA | NA |
| OTU5051 | NA | + | NA | NA |
| OTU17216 | NA | NA | NA | + |
| OTU4256 | NA | NA | NA | NA |
| OTU16891 | NA | NA | - | NA |
| OTU16841 | - | NA | NA | NA |
| OTU11203 | NA | NA | + | - |
| OTU1079 | + | NA | NA | NA |
| OTU4188 | + | NA | NA | NA |
| OTU9050 | + | NA | NA | NA |
| OTU13792 | + | NA | NA | NA |
| OTU17593 | + | NA | - | NA |
| OTU10908 | NA | NA | + | - |
| OTU5047 | - | NA | NA | NA |
| OTU1339 | - | + | NA | NA |
| OTU18876 | NA | NA | + | NA |
| OTU18453 | NA | - | NA | + |
| OTU4660 | NA | NA | NA | - |
| OTU1785 | - | + | NA | NA |
| OTU2755 | NA | NA | NA | NA |
| OTU1005 | NA | NA | - | + |
| OTU17732 | + | NA | - | NA |
| OTU19116 | NA | NA | - | NA |
| OTU11841 | - | NA | + | NA |
| OTU18049 | NA | NA | - | + |
| OTU14590 | NA | NA | NA | NA |
| OTU13320 | NA | NA | - | NA |
| OTU9801 | NA | NA | NA | NA |
| OTU10724 | NA | NA | + | - |
| OTU8628 | NA | NA | - | NA |
| OTU13846 | NA | NA | + | - |
| OTU19123 | + | - | - | NA |
| OTU10291 | - | NA | NA | NA |
| OTU4012 | NA | NA | NA | NA |
| OTU3522 | NA | NA | - | NA |
| OTU3805 | - | + | NA | NA |
| OTU16210 | - | NA | NA | NA |
| OTU13654 | NA | - | NA | + |
| OTU14569 | + | NA | - | NA |
| OTU15046 | NA | NA | NA | + |
| OTU14904 | + | - | NA | NA |
| OTU14608 | NA | NA | NA | NA |
| OTU9964 | - | NA | + | NA |
| OTU14940 | + | NA | - | NA |
| OTU4121 | NA | NA | - | NA |
| OTU11727 | + | - | - | NA |
| OTU17484 | NA | NA | - | + |
| OTU886 | NA | NA | NA | NA |
| OTU3114 | NA | NA | NA | NA |
| OTU6925 | - | NA | + | NA |
| OTU18806 | NA | NA | NA | + |
| OTU17799 | NA | NA | NA | NA |
| OTU9031 | + | NA | NA | NA |
| OTU13164 | + | NA | NA | NA |
| OTU4368 | NA | NA | + | NA |
| OTU13837 | NA | NA | + | - |
| OTU2113 | + | NA | NA | NA |
| OTU13141 | NA | - | NA | + |
| OTU2816 | + | NA | - | NA |
| OTU17566 | NA | NA | NA | + |
| OTU14772 | NA | NA | NA | NA |
| OTU13179 | NA | NA | - | NA |
| OTU9554 | - | NA | + | NA |
| OTU18558 | - | NA | + | NA |
| OTU4502 | + | NA | - | NA |
| OTU17595 | - | NA | NA | NA |
| OTU17824 | NA | NA | + | - |
| OTU7791 | + | NA | - | NA |
| OTU743 | + | - | - | NA |
| OTU13227 | + | NA | NA | NA |
| OTU15065 | NA | - | NA | NA |
| OTU18423 | NA | NA | NA | + |
| OTU3534 | NA | NA | NA | - |
| OTU17960 | NA | NA | - | + |
| OTU18059 | - | NA | NA | + |
| OTU14806 | + | NA | NA | NA |
| OTU16026 | + | NA | - | NA |
| OTU104 | NA | - | + | NA |
| OTU15025 | + | NA | - | NA |
| OTU15674 | + | NA | NA | NA |
| OTU9493 | + | NA | - | NA |
| OTU18032 | NA | NA | - | + |
| OTU18875 | NA | NA | NA | NA |
| OTU17445 | + | NA | - | NA |
| OTU14610 | NA | NA | + | NA |
| OTU16 | + | NA | - | + |
| OTU16630 | - | NA | NA | NA |
| OTU13355 | NA | - | NA | + |
| OTU830 | + | NA | NA | NA |
| OTU15105 | NA | NA | NA | + |
| OTU13793 | + | - | NA | NA |
| OTU6553 | - | NA | + | NA |
| OTU10889 | NA | NA | - | + |
| OTU1044 | + | NA | - | NA |
| OTU17709 | + | NA | - | NA |
| OTU13352 | NA | NA | NA | NA |
| OTU2173 | - | NA | NA | + |
| OTU17831 | + | NA | NA | NA |
| OTU17829 | NA | NA | + | NA |
| OTU12257 | NA | NA | + | NA |
| OTU11636 | NA | NA | NA | NA |
| OTU2138 | - | NA | NA | + |
| OTU2992 | + | NA | - | NA |
| OTU13882 | + | NA | - | NA |
| OTU3757 | NA | NA | + | NA |
| OTU14328 | - | NA | + | NA |
| OTU13316 | + | NA | - | NA |
| OTU1002 | NA | NA | NA | NA |
| OTU13207 | + | NA | - | NA |
| OTU11780 | - | + | NA | NA |
| OTU5621 | - | NA | NA | NA |
| OTU13099 | + | NA | - | NA |
| OTU15270 | + | NA | NA | NA |
| OTU6318 | - | + | NA | NA |
| OTU13517 | + | NA | - | NA |
| OTU14716 | NA | NA | NA | NA |
| OTU6712 | NA | NA | NA | NA |
| OTU15774 | - | NA | NA | NA |
| OTU9587 | NA | NA | NA | NA |
| OTU11495 | + | NA | NA | NA |
| OTU17813 | - | NA | + | NA |
| OTU17598 | NA | NA | NA | NA |
| OTU2854 | - | NA | + | NA |
| OTU10096 | NA | NA | NA | NA |
| OTU15620 | - | NA | NA | NA |
| OTU14695 | NA | NA | NA | NA |
| OTU16345 | + | NA | NA | NA |
| OTU8419 | NA | NA | NA | - |
| OTU12295 | NA | NA | NA | NA |
| OTU17719 | NA | NA | NA | NA |
| OTU15580 | + | NA | - | NA |
| OTU5232 | + | NA | NA | NA |
| OTU149 | + | NA | - | + |
| OTU13329 | NA | + | NA | NA |
| OTU14754 | NA | NA | NA | + |
| OTU15533 | NA | NA | - | + |
| OTU17504 | - | NA | + | NA |
| OTU19098 | + | NA | - | NA |
| OTU487 | + | NA | NA | NA |
| OTU18053 | NA | NA | - | + |
| OTU11489 | + | NA | NA | - |
| OTU253 | NA | NA | NA | NA |
| OTU4349 | - | NA | NA | + |
| OTU17801 | NA | NA | NA | NA |
| OTU16113 | NA | NA | NA | NA |
| OTU6202 | - | NA | NA | NA |
| OTU2103 | NA | - | NA | + |
| OTU4140 | - | NA | + | NA |
| OTU13041 | NA | NA | NA | NA |
| OTU4371 | NA | NA | NA | NA |
| OTU18407 | + | NA | NA | NA |
| OTU17805 | NA | NA | NA | NA |
| OTU14758 | NA | NA | NA | NA |
| OTU18039 | - | NA | + | NA |
| OTU12763 | - | NA | + | NA |
| OTU19163 | - | + | + | NA |
| OTU13084 | NA | NA | + | NA |
| OTU13100 | NA | NA | NA | NA |
| OTU13805 | + | NA | NA | NA |
| OTU9295 | + | NA | - | NA |
| OTU14620 | + | NA | NA | NA |
| OTU14707 | + | NA | - | NA |
| OTU13370 | + | NA | - | NA |
| OTU13839 | - | NA | + | NA |
| OTU15925 | NA | - | NA | + |
| OTU1783 | NA | NA | - | + |
| OTU15795 | NA | NA | NA | NA |
| OTU14739 | NA | NA | NA | NA |
| OTU5004 | NA | NA | NA | NA |
| OTU5428 | NA | + | NA | - |
| OTU17873 | NA | + | - | NA |
| OTU13412 | NA | NA | - | + |
| OTU9172 | NA | NA | NA | NA |
| OTU6163 | - | + | - | + |
| OTU81 | + | - | NA | NA |
| OTU14022 | NA | NA | NA | NA |
| OTU13054 | + | NA | - | NA |
| OTU9829 | - | + | NA | NA |
| OTU8278 | + | NA | - | NA |
| OTU8694 | NA | NA | NA | + |
| OTU13359 | + | NA | NA | NA |
| OTU9708 | + | - | NA | NA |
| OTU17076 | - | NA | NA | NA |
| OTU12476 | - | + | NA | NA |
| OTU5513 | NA | NA | + | - |
| OTU2111 | NA | NA | NA | NA |
| OTU14966 | NA | NA | NA | NA |
| OTU14748 | NA | + | + | NA |
| OTU12223 | NA | NA | NA | NA |
| OTU17794 | NA | NA | NA | NA |
| OTU5889 | + | NA | - | NA |
| OTU14469 | + | NA | NA | NA |
| OTU14454 | + | NA | NA | + |
| OTU8707 | NA | NA | NA | NA |
| OTU17861 | - | NA | + | NA |
| OTU12159 | NA | - | NA | + |
| OTU17548 | NA | - | NA | + |
| OTU13193 | + | NA | NA | - |
| OTU11533 | NA | NA | + | NA |
| OTU17828 | - | NA | NA | NA |
| OTU2648 | + | - | NA | NA |
| OTU15523 | NA | NA | NA | NA |
| OTU6077 | - | NA | NA | NA |
| OTU10946 | + | NA | NA | NA |
| OTU17802 | - | NA | NA | NA |
| OTU18055 | NA | NA | NA | NA |
| OTU17830 | NA | NA | NA | NA |
| OTU6789 | NA | NA | - | + |
| OTU17849 | NA | NA | NA | + |
| OTU5262 | NA | NA | NA | NA |
| OTU1752 | NA | NA | NA | NA |
| OTU11105 | NA | NA | - | + |
| OTU13332 | NA | - | NA | + |
| OTU12593 | NA | NA | NA | - |
| OTU17604 | - | NA | NA | + |
| OTU9651 | NA | NA | NA | NA |
| OTU13133 | NA | NA | + | NA |
| OTU10314 | + | NA | - | NA |
| OTU11693 | NA | NA | NA | NA |
| OTU16084 | NA | - | NA | + |
| OTU15307 | + | NA | - | NA |
| OTU3768 | - | NA | + | NA |
| OTU17658 | - | NA | NA | + |
| OTU6156 | NA | NA | - | + |
| OTU5031 | NA | NA | NA | NA |
| OTU12039 | NA | - | NA | NA |
| OTU17791 | - | + | + | NA |
| OTU2096 | NA | NA | - | + |
| OTU16916 | NA | - | NA | + |
| OTU14736 | NA | + | NA | - |
| OTU13850 | NA | NA | NA | + |
| OTU16198 | + | NA | - | NA |
| OTU115 | NA | NA | NA | NA |
| OTU18048 | NA | NA | NA | NA |
| OTU3926 | + | NA | NA | NA |
| OTU9370 | + | NA | - | NA |
| OTU15977 | NA | NA | NA | NA |
| OTU2983 | - | NA | NA | NA |
| OTU15723 | + | NA | NA | NA |
| OTU18066 | - | NA | + | NA |
| OTU4989 | + | NA | - | NA |
| OTU17771 | NA | NA | NA | NA |
| OTU350 | NA | NA | NA | NA |
| OTU18081 | - | NA | + | NA |
| OTU17230 | NA | + | NA | NA |
| OTU10210 | NA | - | NA | + |
| OTU3722 | NA | NA | NA | - |
| OTU789 | + | NA | - | NA |
| OTU15334 | NA | NA | NA | - |
| OTU8629 | - | NA | + | NA |
| OTU15338 | NA | NA | NA | NA |
| OTU16070 | NA | NA | - | + |
| OTU11721 | NA | + | + | - |
| OTU14122 | NA | NA | + | - |
| OTU10805 | NA | NA | - | + |
| OTU2140 | + | - | NA | NA |
| OTU19199 | + | NA | NA | NA |
| OTU2964 | + | NA | - | NA |
| OTU325 | + | NA | - | NA |
| OTU17354 | - | NA | NA | NA |
| OTU9797 | NA | + | + | - |
| OTU1699 | NA | NA | NA | NA |
| OTU18708 | NA | NA | NA | NA |
| OTU8539 | - | NA | + | NA |
| OTU11696 | + | NA | NA | NA |
| OTU8584 | NA | NA | NA | NA |
| OTU13118 | + | NA | NA | NA |
| OTU12447 | NA | NA | NA | NA |
| OTU14142 | NA | NA | NA | NA |
| OTU5860 | NA | NA | NA | NA |
| OTU985 | - | NA | NA | NA |
| OTU16193 | + | NA | NA | NA |
| OTU14715 | NA | - | NA | + |
| OTU17842 | - | NA | NA | NA |
| OTU3882 | NA | + | NA | - |
| OTU12155 | NA | NA | + | - |
| OTU12523 | NA | NA | + | NA |
| OTU3226 | + | NA | - | NA |
| OTU18292 | + | NA | NA | NA |
| OTU4248 | NA | NA | NA | NA |
| OTU6622 | + | NA | NA | NA |
| OTU16219 | NA | NA | NA | NA |
| OTU15019 | NA | NA | NA | + |
| OTU12101 | NA | NA | NA | NA |
| OTU9212 | + | NA | - | NA |
| OTU17869 | - | NA | NA | + |
| OTU12509 | - | NA | NA | NA |
| OTU11901 | - | NA | + | NA |
| OTU8149 | NA | NA | - | NA |
| OTU10287 | NA | + | NA | NA |
| OTU13774 | + | NA | - | NA |
| OTU7461 | NA | NA | NA | - |
| OTU16806 | NA | NA | - | NA |
| OTU15415 | - | NA | - | + |
| OTU3357 | + | NA | - | NA |
| OTU18760 | + | NA | - | NA |
| OTU108 | NA | NA | - | NA |
| OTU11024 | NA | - | NA | NA |
| OTU14936 | + | NA | - | NA |
| OTU6363 | + | NA | NA | NA |
| OTU1004 | NA | NA | NA | + |
| OTU18213 | NA | NA | NA | NA |
| OTU8807 | NA | NA | NA | NA |
| OTU7422 | NA | NA | NA | NA |
| OTU14861 | NA | NA | NA | NA |
| OTU9047 | NA | - | - | + |
| OTU14749 | - | NA | + | NA |
| OTU18779 | + | - | NA | NA |
| OTU2453 | + | NA | NA | NA |
| OTU15274 | + | NA | - | NA |
| OTU10758 | NA | NA | + | NA |
| OTU13291 | NA | + | NA | - |
| OTU13333 | + | NA | NA | NA |
| OTU17577 | - | NA | NA | NA |
| OTU17250 | - | NA | NA | NA |
| OTU2764 | + | NA | - | NA |
| OTU5368 | NA | NA | NA | - |
| OTU10721 | NA | + | NA | NA |
| OTU4522 | NA | NA | NA | NA |
| OTU17900 | - | NA | + | NA |
| OTU14792 | NA | NA | NA | NA |
| OTU17804 | NA | NA | NA | NA |
| OTU15021 | + | NA | NA | NA |
| OTU250 | NA | NA | NA | - |
| OTU17887 | NA | NA | NA | + |
| OTU16855 | + | NA | - | NA |
| OTU15111 | - | + | NA | NA |
| OTU14752 | NA | NA | NA | - |
| OTU14635 | + | - | NA | NA |
| OTU10227 | + | NA | NA | NA |
| OTU1490 | NA | NA | NA | NA |
| OTU13742 | NA | NA | + | - |
| OTU16848 | NA | NA | + | NA |
| OTU15082 | NA | + | NA | - |
| OTU19109 | + | NA | NA | NA |
| OTU17734 | - | NA | NA | NA |
| OTU11903 | NA | NA | NA | + |
| OTU18881 | NA | - | NA | + |
| OTU3775 | NA | NA | NA | NA |
| OTU1657 | + | NA | NA | NA |
| OTU14162 | NA | NA | NA | + |
| OTU18897 | NA | NA | - | NA |
| OTU362 | + | NA | - | + |
| OTU17959 | NA | NA | NA | NA |
| OTU13856 | + | NA | NA | NA |
| OTU1898 | + | NA | - | NA |
| OTU5409 | NA | NA | + | - |
| OTU105 | + | - | NA | NA |
| OTU7230 | + | NA | NA | NA |
| OTU17748 | + | NA | NA | NA |
| OTU13753 | NA | NA | NA | + |
| OTU13121 | NA | NA | NA | NA |
| OTU18410 | + | NA | NA | NA |
| OTU2445 | + | NA | NA | NA |
| OTU18477 | NA | NA | NA | NA |
| OTU3063 | NA | NA | + | - |
| OTU18790 | NA | NA | - | NA |
| OTU15221 | - | NA | + | NA |
| OTU6746 | NA | NA | - | + |
| OTU14182 | NA | NA | + | NA |
| OTU3924 | NA | NA | NA | - |
| OTU9268 | + | NA | NA | NA |
| OTU13356 | - | + | NA | NA |
| OTU9333 | NA | NA | NA | NA |
| OTU11058 | - | + | NA | NA |
| OTU7102 | + | NA | - | NA |
| OTU15330 | NA | NA | NA | NA |
| OTU16580 | + | NA | NA | NA |
| OTU8733 | + | NA | - | NA |
| OTU15627 | NA | + | NA | - |
| OTU11393 | - | NA | NA | + |
| OTU9586 | NA | NA | NA | + |
| OTU11688 | + | NA | - | NA |
| OTU14587 | - | NA | + | NA |
| OTU15758 | + | NA | NA | NA |
| OTU17878 | NA | NA | NA | NA |
| OTU5289 | + | NA | NA | NA |
| OTU13586 | NA | NA | NA | NA |
| OTU16342 | NA | NA | NA | - |
| OTU18443 | + | NA | NA | NA |
| OTU2739 | NA | NA | - | + |
| OTU18047 | NA | - | - | NA |
| OTU16056 | NA | NA | - | + |
| OTU6380 | + | - | NA | NA |
| OTU16505 | NA | - | NA | + |
| OTU14776 | NA | NA | + | - |
| OTU9478 | NA | NA | - | + |
| OTU3727 | - | NA | NA | NA |
| OTU15051 | NA | NA | NA | NA |
| OTU18056 | NA | NA | + | - |
| OTU7599 | NA | NA | + | NA |
| OTU6634 | + | NA | - | NA |
| OTU15275 | NA | NA | - | + |
| OTU4987 | NA | NA | + | - |
| OTU3743 | + | NA | - | NA |
| OTU14659 | NA | NA | NA | + |
| OTU13014 | + | - | NA | NA |
| OTU13312 | NA | NA | + | NA |
| OTU13136 | NA | - | NA | NA |
| OTU14615 | NA | - | NA | + |
| OTU19142 | + | NA | - | NA |
| OTU683 | + | NA | NA | NA |
| OTU11811 | - | NA | + | NA |
| OTU11889 | NA | NA | NA | NA |
| OTU13 | NA | NA | NA | + |
| OTU17750 | NA | + | NA | NA |
| OTU9064 | NA | NA | NA | - |
| OTU17300 | NA | - | NA | NA |
| OTU14614 | - | NA | NA | NA |
| OTU13043 | NA | NA | NA | NA |
| OTU17787 | NA | NA | - | NA |
| OTU14735 | NA | + | + | - |
| OTU8262 | + | NA | NA | NA |
| OTU14675 | NA | - | NA | NA |
| OTU13824 | + | NA | NA | NA |
| OTU14673 | NA | NA | NA | NA |
| OTU2421 | + | NA | - | NA |
| OTU16935 | + | NA | NA | NA |
| OTU13724 | + | - | NA | NA |
| OTU8426 | + | NA | - | NA |
| OTU13197 | + | NA | NA | NA |
| OTU9579 | - | + | NA | NA |
| OTU9450 | + | NA | NA | NA |
| OTU2082 | - | NA | NA | + |
| OTU17645 | - | NA | NA | NA |
| OTU17788 | - | NA | NA | NA |
| OTU18436 | NA | NA | NA | + |
| OTU6725 | NA | NA | NA | + |
| OTU6988 | + | NA | NA | NA |
| OTU12275 | NA | + | - | NA |
| OTU9881 | NA | NA | NA | NA |
| OTU17601 | NA | NA | - | + |
| OTU676 | NA | NA | NA | - |
| OTU9784 | - | + | NA | NA |
| OTU13279 | + | NA | NA | NA |
| OTU17647 | - | NA | + | NA |
| OTU3651 | + | NA | - | NA |
| OTU17947 | NA | NA | + | - |
| OTU13831 | NA | - | NA | NA |
| OTU2521 | NA | - | NA | NA |
| OTU10953 | NA | NA | - | + |
| OTU13678 | NA | NA | + | - |
| OTU3683 | NA | NA | NA | - |
| OTU18070 | NA | NA | NA | NA |
| OTU13162 | NA | + | NA | NA |
| OTU2564 | + | NA | - | NA |
| OTU14790 | + | NA | NA | NA |
| OTU6350 | + | NA | - | NA |
| OTU13501 | + | NA | NA | NA |
| OTU16637 | NA | NA | + | - |
| OTU17807 | NA | NA | NA | NA |
| OTU14740 | NA | NA | + | NA |
| OTU14929 | - | + | NA | NA |
| OTU18464 | NA | NA | NA | NA |
| OTU14101 | + | NA | NA | NA |
| OTU14366 | - | NA | NA | + |
| OTU17851 | NA | NA | NA | NA |
| OTU11449 | + | NA | NA | NA |
| OTU10139 | + | NA | NA | NA |
| OTU18730 | + | NA | - | NA |
| OTU9589 | + | NA | NA | NA |
| OTU17714 | NA | NA | + | NA |
| OTU16574 | NA | NA | - | + |
| OTU17562 | NA | NA | - | + |
| OTU15398 | NA | NA | - | + |
| OTU14900 | NA | NA | NA | NA |
| OTU14878 | NA | + | NA | NA |
| OTU10120 | NA | NA | NA | + |
| OTU5929 | NA | NA | NA | NA |
| OTU11218 | + | NA | NA | NA |
| OTU8745 | - | NA | + | NA |
| OTU4137 | - | NA | + | NA |
| OTU12714 | - | NA | NA | NA |
| OTU13384 | - | + | + | NA |
| OTU9713 | + | - | NA | NA |
| OTU2141 | + | NA | NA | NA |
| OTU12261 | - | NA | NA | NA |
| OTU18145 | - | NA | + | NA |
| OTU18038 | - | NA | + | NA |
| OTU13782 | NA | NA | NA | NA |
| OTU5223 | - | NA | NA | NA |
| OTU13187 | + | NA | - | NA |
| OTU1127 | NA | NA | NA | NA |
| OTU13864 | NA | NA | NA | NA |
| OTU8726 | NA | NA | + | NA |
| OTU4984 | + | NA | - | NA |
| OTU5872 | NA | NA | NA | NA |
| OTU17547 | + | NA | - | NA |
| OTU13821 | NA | - | NA | + |
| OTU16209 | NA | NA | - | + |
| OTU17929 | - | NA | NA | NA |
| OTU18123 | NA | NA | - | + |
| OTU13031 | NA | NA | NA | NA |
| OTU7322 | NA | NA | + | NA |
| OTU2094 | NA | NA | - | NA |
| OTU15036 | - | NA | + | NA |
| OTU4988 | + | NA | - | NA |
| OTU7837 | + | NA | - | NA |
| OTU2464 | NA | NA | + | NA |
| OTU13094 | + | - | - | NA |
| OTU17857 | NA | NA | - | NA |
| OTU9358 | NA | NA | + | NA |
| OTU15129 | - | NA | NA | + |
| OTU4642 | NA | + | NA | NA |
| OTU17779 | NA | NA | NA | NA |
| OTU13593 | + | NA | - | NA |
| OTU17800 | NA | NA | NA | NA |
| OTU18552 | - | + | NA | NA |
| OTU18570 | + | NA | NA | NA |
| OTU13274 | + | NA | - | NA |
| OTU18054 | NA | NA | NA | NA |
| OTU17684 | NA | NA | NA | NA |
| OTU15070 | + | NA | NA | NA |
| OTU3675 | NA | NA | NA | NA |
| OTU18102 | NA | NA | NA | NA |
| OTU15116 | + | NA | - | NA |
| OTU5699 | + | NA | NA | NA |
| OTU3492 | + | NA | - | NA |
| OTU7516 | + | NA | NA | NA |
| OTU18473 | NA | NA | NA | + |
| OTU2105 | NA | NA | NA | NA |
| OTU3559 | NA | + | NA | - |
| OTU2994 | - | NA | NA | NA |
| OTU13104 | NA | NA | - | NA |
| OTU387 | NA | NA | NA | - |
| OTU238 | NA | NA | NA | NA |
| OTU9664 | - | + | NA | NA |
| OTU6948 | NA | NA | NA | NA |
| OTU608 | + | NA | NA | NA |
| OTU13056 | NA | + | NA | - |
| OTU13736 | - | NA | NA | NA |
| OTU11382 | + | - | - | NA |
| OTU13298 | + | NA | - | NA |
| OTU8532 | NA | NA | NA | NA |
| OTU12455 | - | NA | + | NA |
| OTU11864 | NA | NA | NA | NA |
| OTU10468 | NA | NA | NA | NA |
| OTU12448 | + | NA | NA | NA |
| OTU16943 | - | NA | + | NA |
| OTU13124 | NA | NA | NA | NA |
| OTU17580 | + | NA | NA | NA |
| OTU14704 | NA | NA | + | - |
| OTU7696 | + | NA | - | NA |
| OTU11174 | NA | + | NA | NA |
| OTU17952 | NA | NA | + | - |
| OTU13021 | NA | - | NA | + |
| OTU14611 | + | NA | - | NA |
| OTU18062 | NA | NA | NA | NA |
| OTU18042 | NA | NA | NA | NA |
| OTU10420 | NA | + | NA | NA |
| OTU16222 | NA | NA | NA | NA |
| OTU17321 | + | NA | - | NA |
| OTU1820 | NA | NA | - | + |
| OTU8925 | NA | NA | NA | + |
| OTU1673 | NA | NA | NA | NA |
| OTU1125 | NA | NA | NA | + |
| OTU18496 | NA | NA | NA | NA |
| OTU17129 | NA | NA | NA | NA |
| OTU13505 | NA | NA | NA | NA |
| OTU3736 | NA | + | NA | NA |
| OTU17643 | NA | NA | NA | NA |
| OTU239 | + | NA | - | NA |
| OTU7636 | + | NA | - | NA |
| OTU15044 | NA | + | NA | - |
| OTU2084 | NA | NA | NA | NA |
| OTU17817 | NA | NA | - | NA |
| OTU16264 | NA | NA | NA | NA |
| OTU19033 | NA | NA | NA | NA |
| OTU6524 | - | NA | + | - |
| OTU12303 | - | NA | NA | NA |
| OTU17712 | NA | NA | - | + |
| OTU17587 | NA | NA | NA | NA |
| OTU14376 | + | NA | NA | NA |
| OTU13114 | NA | NA | + | - |
| OTU13969 | NA | NA | NA | NA |
| OTU517 | + | NA | NA | NA |
| OTU15239 | NA | NA | NA | NA |
| OTU6870 | - | NA | NA | NA |
| OTU2629 | + | NA | - | NA |
| OTU3398 | - | NA | + | NA |
| OTU15509 | + | NA | - | NA |
| OTU8161 | + | NA | - | NA |
| OTU17534 | NA | NA | - | + |
| OTU13044 | + | - | NA | NA |
| OTU17973 | + | NA | - | NA |
| OTU14794 | - | NA | NA | NA |
| OTU14930 | NA | NA | NA | NA |
| OTU10064 | - | NA | + | NA |
| OTU10923 | NA | NA | NA | NA |
| OTU13083 | - | NA | NA | NA |
| OTU15072 | NA | + | NA | - |
| OTU14963 | NA | NA | NA | NA |
| OTU6126 | NA | + | NA | - |
| OTU14849 | + | NA | NA | NA |
| OTU15063 | - | NA | NA | NA |
| OTU14783 | NA | NA | NA | - |
| OTU2515 | + | NA | NA | NA |
| OTU14760 | - | NA | + | NA |
| OTU14047 | NA | NA | NA | + |
| OTU17624 | NA | + | NA | - |
| OTU13838 | - | NA | NA | NA |
| OTU18251 | + | - | - | NA |
| OTU6190 | NA | NA | NA | NA |
| OTU18468 | NA | NA | NA | NA |
| OTU12664 | - | NA | NA | NA |
| OTU1476 | + | NA | NA | NA |
| OTU2638 | NA | - | NA | + |
| OTU13191 | NA | NA | + | - |
| OTU19195 | + | NA | NA | NA |
| OTU18764 | + | - | NA | NA |
| OTU18086 | NA | NA | - | NA |
| OTU18065 | NA | NA | NA | + |
| OTU13160 | NA | NA | + | - |
| OTU15064 | NA | NA | NA | + |
| OTU13107 | NA | - | NA | + |
| OTU14786 | NA | NA | NA | NA |
| OTU16843 | NA | NA | - | + |
| OTU11298 | NA | NA | NA | NA |
| OTU179 | NA | NA | NA | NA |
| OTU3073 | NA | NA | + | - |
| OTU7144 | + | - | - | NA |
| OTU13692 | + | NA | - | NA |
| OTU5490 | NA | NA | NA | NA |
| OTU14742 | + | NA | - | NA |
| OTU13855 | NA | NA | NA | NA |
| OTU13906 | NA | NA | NA | NA |
| OTU18036 | NA | NA | NA | + |
| OTU17792 | - | NA | + | NA |
| OTU9398 | + | - | NA | NA |
| OTU14630 | + | NA | NA | NA |
| OTU13123 | NA | NA | NA | + |
| OTU17865 | - | NA | + | NA |
| OTU18008 | NA | NA | - | + |
| OTU16873 | + | NA | NA | NA |
| OTU15346 | + | NA | NA | NA |
| OTU2945 | NA | - | NA | + |
| OTU14007 | + | NA | NA | NA |
| OTU14699 | + | NA | - | NA |
| OTU10072 | NA | NA | NA | NA |
| OTU13328 | NA | + | NA | NA |
| OTU7688 | NA | NA | NA | NA |
| OTU18018 | - | + | NA | NA |
| OTU13606 | + | NA | - | NA |
| OTU15042 | NA | NA | NA | NA |
| OTU13671 | NA | NA | NA | NA |
| OTU1063 | + | NA | NA | NA |
| OTU13144 | NA | NA | + | - |
| OTU5689 | NA | NA | NA | NA |
| OTU2795 | NA | NA | NA | - |
| OTU7914 | + | NA | - | NA |
| OTU2626 | + | NA | NA | NA |
| OTU17868 | NA | NA | + | - |
| OTU2579 | + | - | NA | NA |
| OTU16736 | - | NA | NA | NA |
| OTU17685 | NA | NA | NA | NA |
| OTU14896 | + | NA | NA | NA |
| OTU16188 | NA | NA | - | + |
| OTU17715 | NA | - | NA | + |
| OTU13349 | NA | NA | NA | NA |
| OTU394 | + | NA | - | NA |
| OTU14634 | + | NA | NA | + |
| OTU17341 | + | - | - | NA |
| OTU17672 | - | NA | + | NA |
| OTU14902 | NA | NA | - | + |
| OTU17529 | NA | - | NA | NA |
| OTU3714 | NA | NA | - | NA |
| OTU14763 | + | NA | - | NA |
| OTU17922 | NA | NA | NA | NA |
| OTU17996 | NA | NA | NA | NA |
| OTU5630 | NA | NA | NA | NA |
| OTU14664 | + | NA | NA | NA |
| OTU13067 | + | NA | NA | NA |
| OTU9539 | + | - | NA | NA |
| OTU14803 | NA | NA | - | NA |
| OTU4403 | + | NA | - | NA |
| OTU6996 | + | NA | - | NA |
| OTU6322 | + | NA | NA | NA |
| OTU14737 | NA | NA | NA | NA |
| OTU3754 | - | NA | NA | NA |
| OTU15069 | NA | + | - | NA |
| OTU11577 | NA | + | NA | - |
| OTU14753 | NA | NA | + | NA |
| OTU14640 | NA | + | NA | NA |
| OTU7367 | NA | + | NA | NA |
| OTU14657 | NA | NA | NA | NA |
| OTU16493 | NA | NA | NA | + |
| OTU19165 | + | NA | - | NA |
| OTU3781 | + | NA | NA | NA |
| OTU13000 | NA | NA | - | + |
| OTU18098 | NA | NA | NA | NA |
| OTU17752 | NA | NA | - | NA |
| OTU103 | + | NA | NA | NA |
| OTU9262 | + | NA | NA | NA |
| OTU8827 | NA | NA | NA | NA |
| OTU11178 | NA | NA | + | NA |
| OTU15930 | NA | NA | NA | + |
| OTU10293 | + | NA | NA | NA |
| OTU10501 | + | NA | NA | - |
| OTU16469 | NA | NA | NA | + |
| OTU6226 | NA | NA | NA | - |
| OTU14882 | - | NA | NA | NA |
| OTU16901 | NA | NA | NA | + |
| OTU8729 | + | NA | NA | NA |
| OTU19160 | + | NA | - | NA |
| OTU4340 | - | NA | NA | + |
| OTU5347 | + | NA | NA | NA |
| OTU12227 | NA | + | NA | - |
| OTU8901 | NA | + | - | + |
| OTU13324 | NA | NA | NA | NA |
| OTU13462 | NA | NA | NA | NA |
| OTU541 | NA | NA | NA | - |
| OTU2436 | + | NA | - | NA |
| OTU5463 | NA | NA | NA | + |
| OTU3315 | + | NA | NA | NA |
| OTU17561 | NA | NA | NA | + |
| OTU9612 | + | NA | NA | + |
| OTU2139 | NA | NA | + | - |
| OTU18391 | + | NA | - | NA |
| OTU10949 | + | NA | NA | NA |
| OTU2858 | NA | NA | NA | NA |
| OTU7258 | NA | NA | NA | NA |
| OTU7841 | + | NA | NA | NA |
| OTU13150 | NA | NA | NA | - |
| OTU5839 | NA | NA | NA | NA |
| OTU9265 | NA | + | + | - |
| OTU18075 | NA | - | NA | + |
| OTU8385 | + | NA | NA | NA |
| OTU11232 | + | NA | NA | NA |
| OTU9725 | + | NA | - | NA |
| OTU16990 | NA | - | NA | + |
| OTU9917 | NA | NA | NA | NA |
| OTU8613 | NA | - | NA | + |
| OTU19184 | NA | NA | NA | + |
| OTU18085 | NA | NA | NA | NA |
| OTU9676 | - | NA | + | NA |
| OTU13321 | NA | NA | NA | + |
| OTU13350 | + | NA | NA | NA |
| OTU17551 | NA | NA | + | NA |
| OTU18378 | + | - | - | + |
| OTU3298 | NA | NA | NA | - |
| OTU5358 | NA | NA | - | + |
| OTU17751 | NA | NA | - | NA |
| OTU14987 | NA | NA | + | NA |
| OTU15022 | + | NA | NA | NA |
| OTU12686 | + | - | NA | NA |
| OTU7693 | NA | NA | - | NA |
| OTU17872 | NA | NA | NA | NA |
| OTU6503 | NA | - | NA | NA |
| OTU15059 | NA | + | NA | NA |
| OTU14482 | NA | NA | NA | NA |
| OTU4088 | NA | NA | NA | NA |
| OTU8241 | + | NA | - | NA |
| OTU19030 | + | NA | NA | NA |
| OTU6055 | NA | - | NA | NA |
| OTU14633 | NA | NA | + | NA |
| OTU9978 | + | NA | - | NA |
| OTU13835 | - | NA | NA | NA |
| OTU4453 | + | NA | NA | NA |
| OTU9760 | NA | NA | NA | NA |
| OTU13888 | + | NA | NA | NA |
| OTU9959 | NA | NA | + | - |
| OTU13175 | NA | NA | NA | + |
| OTU8939 | NA | NA | + | - |
| OTU9448 | + | - | NA | NA |
| OTU10929 | NA | NA | + | NA |
| OTU7590 | + | NA | - | NA |
| OTU2120 | - | NA | NA | NA |
| OTU14054 | - | NA | + | NA |
| OTU10241 | NA | NA | + | - |
| OTU14974 | + | NA | - | NA |
| OTU18977 | NA | NA | + | NA |
| OTU23 | + | NA | - | NA |
| OTU3130 | - | NA | NA | NA |
| OTU13005 | NA | NA | - | NA |
| OTU17615 | NA | NA | - | + |
| OTU3772 | NA | NA | NA | NA |
| OTU17889 | NA | NA | NA | NA |
| OTU17729 | NA | NA | NA | NA |
| OTU18063 | NA | NA | NA | - |
| OTU18174 | NA | - | NA | + |
| OTU14997 | NA | NA | NA | NA |
| OTU15018 | - | NA | + | NA |
| OTU18073 | - | NA | NA | NA |
| OTU2081 | NA | NA | NA | NA |
| OTU14842 | NA | NA | NA | NA |
| OTU10912 | + | NA | NA | NA |
| OTU17932 | NA | - | NA | + |
| OTU13788 | NA | + | NA | NA |
| OTU18794 | + | NA | NA | NA |
| OTU17695 | NA | NA | NA | NA |
| OTU13061 | NA | NA | NA | NA |
| OTU9997 | NA | NA | NA | NA |
| OTU13977 | + | NA | NA | NA |
| OTU7309 | + | NA | NA | NA |
| OTU18021 | NA | NA | NA | NA |
| OTU17481 | - | NA | + | NA |
| OTU13877 | NA | + | - | NA |
| OTU14652 | NA | NA | + | - |
| OTU14734 | NA | NA | NA | - |
| OTU13303 | NA | NA | NA | - |
| OTU13116 | - | NA | NA | NA |
| OTU4225 | - | NA | + | NA |
| OTU12234 | NA | - | NA | NA |
| OTU13828 | + | NA | NA | NA |
| OTU7643 | + | NA | - | NA |
| OTU16231 | + | NA | - | NA |
| OTU18624 | + | - | - | NA |
| OTU12602 | NA | - | NA | + |
| OTU14429 | + | NA | - | NA |
| OTU12885 | NA | NA | NA | NA |
| OTU1998 | - | NA | + | NA |
| OTU5121 | + | NA | NA | NA |
| OTU16952 | NA | NA | + | - |
| OTU6210 | NA | NA | NA | NA |
| OTU14939 | + | NA | - | NA |
| OTU13885 | NA | + | NA | NA |
| OTU369 | NA | NA | NA | NA |
| OTU4680 | - | + | + | NA |
| OTU4373 | NA | NA | NA | NA |
| OTU5512 | - | NA | + | NA |
| OTU7556 | + | NA | - | NA |
| OTU3725 | NA | NA | NA | NA |
| OTU15204 | NA | NA | - | NA |
| OTU1993 | NA | NA | + | NA |
| OTU12044 | NA | NA | + | - |
| OTU2158 | NA | NA | + | - |
| OTU18052 | + | NA | - | NA |
| OTU18125 | NA | NA | NA | - |
| OTU13068 | + | NA | - | NA |
| OTU1818 | - | NA | + | NA |
| OTU8082 | + | NA | - | NA |
| OTU14573 | NA | NA | NA | NA |
| OTU6364 | + | NA | - | NA |
| OTU15020 | NA | NA | + | NA |
| OTU13103 | NA | NA | + | - |
| OTU11554 | NA | NA | NA | NA |
| OTU15084 | NA | NA | - | + |
| OTU7910 | + | NA | - | NA |
| OTU18382 | + | - | NA | NA |
| OTU2228 | + | NA | - | NA |
| OTU3595 | NA | NA | + | - |
| OTU12324 | NA | NA | - | + |
| OTU13848 | + | NA | - | NA |
| OTU17864 | NA | NA | + | NA |
| OTU10957 | NA | + | NA | NA |
| OTU713 | + | NA | - | NA |
| OTU733 | + | NA | - | NA |
| OTU15359 | - | + | NA | NA |
| OTU7537 | + | NA | - | NA |
| OTU16496 | + | NA | - | NA |
| OTU17618 | NA | NA | NA | NA |
| OTU13109 | NA | NA | - | + |
| OTU1351 | NA | - | NA | + |
| OTU17594 | + | NA | - | NA |
| OTU17790 | NA | NA | - | + |
| OTU6340 | + | NA | - | NA |
| OTU14574 | NA | NA | - | + |
| OTU17820 | NA | NA | NA | + |
| OTU3104 | NA | NA | + | - |
| OTU15591 | NA | NA | - | + |
| OTU16083 | NA | NA | - | + |
| OTU12224 | - | NA | + | NA |
| OTU14550 | + | NA | - | NA |
| OTU14651 | + | NA | - | NA |
| OTU1878 | NA | NA | - | + |
| OTU5312 | NA | NA | NA | NA |
| OTU18046 | NA | NA | NA | + |
| OTU11918 | NA | NA | NA | - |
| OTU2647 | NA | NA | NA | NA |
| OTU15040 | NA | NA | + | NA |
| OTU15026 | NA | NA | NA | + |
| OTU12750 | + | NA | NA | NA |
| OTU5908 | NA | NA | + | - |
| OTU10404 | NA | NA | NA | NA |
| OTU17902 | NA | - | NA | NA |
| OTU18638 | + | NA | - | NA |
| OTU15673 | NA | NA | - | NA |
| OTU3455 | NA | NA | NA | NA |
| OTU1415 | + | NA | - | NA |
| OTU1267 | NA | NA | NA | NA |
| OTU15078 | NA | + | NA | - |
| OTU4515 | NA | NA | NA | NA |
| OTU9340 | NA | NA | NA | NA |
| OTU17718 | NA | + | NA | NA |
| OTU15088 | - | NA | + | NA |
| OTU17972 | NA | + | NA | - |
| OTU17581 | NA | NA | NA | NA |
| OTU9617 | NA | NA | NA | + |
| OTU17777 | NA | - | + | NA |
| OTU12132 | + | NA | NA | NA |
| OTU6237 | + | NA | NA | NA |
| OTU13876 | NA | NA | NA | NA |
| OTU7191 | + | NA | - | NA |
| OTU13195 | NA | NA | + | - |
| OTU15541 | - | NA | NA | NA |
| OTU7591 | NA | + | + | - |
| OTU17916 | NA | + | - | + |
| OTU17238 | NA | - | NA | + |
| OTU6420 | NA | NA | + | - |
| OTU14814 | NA | NA | NA | NA |
| OTU17847 | NA | NA | NA | - |
| OTU5824 | + | NA | - | NA |
| OTU5198 | NA | NA | NA | NA |
| OTU16064 | NA | NA | - | NA |
| OTU12479 | - | + | NA | NA |
| OTU17810 | NA | NA | NA | NA |
| OTU1704 | - | NA | + | NA |
| OTU17979 | NA | NA | + | - |
| OTU11334 | - | + | NA | NA |
| OTU17856 | NA | NA | NA | NA |
| OTU17854 | NA | NA | NA | NA |
| OTU6283 | NA | NA | NA | + |
| OTU6448 | NA | NA | + | - |
| OTU14738 | NA | - | NA | NA |
| OTU8723 | + | NA | - | NA |
| OTU18002 | + | NA | NA | - |
| OTU13929 | NA | NA | NA | + |
| OTU8980 | NA | NA | - | NA |
| OTU13003 | + | NA | NA | NA |
| OTU13131 | NA | - | NA | + |
| OTU5643 | + | NA | - | NA |
| OTU17863 | NA | NA | NA | NA |
| OTU12215 | NA | + | NA | - |
| OTU17944 | NA | NA | NA | + |
| OTU13986 | NA | NA | NA | NA |
| OTU17945 | NA | NA | + | - |
| OTU857 | NA | NA | NA | NA |
| OTU9446 | NA | NA | NA | NA |
| OTU1888 | NA | - | NA | + |
| OTU16807 | - | NA | + | NA |
| OTU3925 | NA | NA | NA | - |
| OTU1452 | NA | NA | NA | NA |
| OTU3335 | + | NA | - | NA |
| OTU7605 | + | NA | NA | NA |
| OTU13112 | + | NA | - | NA |
| OTU15195 | NA | NA | NA | NA |
| OTU17897 | + | - | - | NA |
| OTU13261 | + | NA | - | NA |
| OTU7597 | + | NA | NA | NA |
| OTU2087 | NA | NA | NA | NA |
| OTU11781 | + | NA | - | NA |
| OTU17814 | NA | + | - | NA |
| OTU6461 | + | NA | - | NA |
| OTU15296 | NA | NA | NA | - |
| OTU5166 | - | + | NA | NA |
| OTU17539 | NA | + | - | NA |
| OTU12277 | - | + | NA | NA |
| OTU14378 | NA | NA | NA | + |
| OTU14090 | NA | NA | - | NA |
| OTU14950 | NA | NA | NA | NA |
| OTU6352 | - | NA | NA | NA |
| OTU5307 | NA | NA | NA | NA |
| OTU13117 | NA | NA | NA | NA |
| OTU16738 | NA | NA | NA | NA |
| OTU9137 | NA | NA | - | NA |
| OTU18611 | + | NA | - | NA |
| OTU3676 | NA | NA | NA | NA |
| OTU10388 | NA | NA | NA | NA |
| OTU4955 | + | NA | NA | NA |
| OTU14189 | NA | NA | - | + |
| OTU13907 | + | NA | NA | - |
| OTU1512 | + | - | - | NA |
| OTU13875 | NA | NA | NA | NA |
| OTU1685 | NA | + | NA | NA |
| OTU13111 | - | NA | + | NA |
| OTU4632 | NA | NA | NA | - |
| OTU10515 | NA | + | NA | NA |
| OTU17373 | + | NA | - | NA |
| OTU13139 | NA | NA | + | NA |
| OTU14667 | NA | + | NA | NA |
| OTU17716 | NA | NA | - | + |
| OTU12111 | NA | NA | + | NA |
| OTU6919 | NA | NA | NA | NA |
| OTU14561 | NA | - | NA | NA |
| OTU14799 | NA | NA | NA | - |
| OTU9010 | NA | NA | + | NA |
| OTU18155 | NA | NA | NA | - |
| OTU1637 | NA | NA | NA | - |
| OTU2704 | + | NA | NA | NA |
| OTU14592 | NA | NA | NA | NA |
| OTU8636 | - | NA | NA | NA |
| OTU10080 | - | NA | NA | NA |
| OTU3242 | + | NA | NA | NA |
| OTU17840 | NA | NA | NA | NA |
| OTU9826 | - | NA | NA | NA |
| OTU12236 | NA | NA | NA | NA |
| OTU14613 | - | NA | NA | NA |
| OTU17879 | NA | + | NA | NA |
| OTU13089 | - | NA | + | NA |
| OTU14970 | NA | NA | NA | NA |
| OTU13568 | NA | NA | NA | - |
| OTU13247 | NA | NA | NA | NA |
| OTU9693 | NA | NA | + | NA |
| OTU15299 | NA | NA | + | - |
| OTU17773 | - | + | NA | NA |
| OTU18305 | NA | NA | NA | NA |
| OTU14942 | NA | - | NA | + |
| OTU11372 | - | NA | + | NA |
| OTU14835 | NA | NA | NA | NA |
| OTU4602 | + | NA | NA | NA |
| OTU6277 | + | NA | NA | NA |
| OTU7521 | + | NA | NA | NA |
| OTU14632 | NA | NA | NA | + |
| OTU10162 | NA | - | + | - |
| OTU5876 | - | NA | NA | NA |
| OTU4710 | - | + | NA | NA |
| OTU18006 | NA | NA | - | + |
| OTU7608 | + | - | - | NA |
| OTU13152 | NA | NA | + | NA |
| OTU1883 | NA | NA | NA | NA |
| OTU2870 | + | - | NA | NA |
| OTU17920 | NA | NA | NA | NA |
| OTU7581 | + | NA | - | NA |
| OTU1716 | NA | - | NA | + |
| OTU14827 | + | NA | NA | NA |
| OTU2851 | + | NA | - | NA |
| OTU5366 | NA | NA | NA | NA |
| OTU14340 | + | NA | NA | NA |
| OTU13895 | NA | NA | NA | + |
| OTU7249 | NA | NA | NA | NA |
| OTU368 | NA | - | NA | + |
| OTU18216 | - | + | NA | NA |
| OTU13185 | + | - | NA | NA |
| OTU2034 | NA | + | NA | NA |
| OTU12628 | NA | NA | NA | NA |
| OTU18189 | NA | NA | NA | - |
| OTU17875 | NA | NA | NA | + |
| OTU13363 | NA | + | NA | NA |
| OTU2333 | NA | NA | NA | + |
| OTU19 | NA | NA | NA | NA |
| OTU13343 | + | NA | - | NA |
| OTU7973 | NA | NA | NA | NA |
| OTU10810 | - | NA | + | NA |
| OTU18092 | NA | - | NA | + |
| OTU2655 | + | NA | - | NA |
| OTU7822 | + | NA | - | NA |
| OTU14481 | NA | NA | - | NA |
| OTU3329 | NA | NA | + | NA |
| OTU9266 | NA | NA | NA | - |
| OTU5702 | + | NA | - | NA |
| OTU4789 | NA | NA | NA | NA |
| OTU7026 | + | - | NA | NA |
| OTU14148 | NA | NA | NA | NA |
| OTU14621 | NA | NA | - | NA |
| OTU1943 | NA | NA | - | NA |
| OTU15247 | + | NA | - | NA |
| OTU13796 | - | NA | NA | NA |
| OTU1709 | - | NA | - | + |
| OTU14891 | NA | - | NA | + |
| OTU3490 | NA | NA | NA | NA |
| OTU18727 | NA | NA | NA | - |
| OTU14830 | + | NA | NA | NA |
| OTU17635 | + | - | NA | NA |
| OTU13304 | + | NA | - | NA |
| OTU12758 | NA | - | NA | + |
| OTU13768 | NA | + | NA | NA |
| OTU9181 | + | - | NA | NA |
| OTU16238 | + | NA | NA | NA |
| OTU13818 | + | NA | - | NA |
| OTU1739 | NA | NA | NA | NA |
| OTU15830 | NA | - | NA | + |
| OTU3222 | NA | NA | NA | - |
| OTU2642 | + | NA | NA | NA |
| OTU15041 | NA | NA | NA | NA |
| OTU12796 | NA | NA | NA | - |
| OTU17763 | NA | NA | NA | NA |
| OTU2773 | NA | NA | NA | NA |
| OTU14324 | NA | NA | + | NA |
| OTU14160 | NA | NA | NA | NA |
| OTU13254 | NA | NA | + | - |
| OTU18312 | NA | NA | NA | NA |
| OTU7450 | NA | NA | NA | NA |
| OTU15127 | + | NA | - | NA |
| OTU14333 | NA | - | NA | + |
| OTU12246 | NA | NA | NA | - |
| OTU593 | + | NA | NA | NA |
| OTU16947 | NA | NA | - | + |
| OTU4956 | NA | NA | NA | NA |
| OTU17877 | NA | NA | + | NA |
| OTU4635 | NA | NA | NA | NA |
| OTU8890 | NA | + | - | NA |
| OTU9577 | NA | NA | NA | NA |
| OTU13134 | - | NA | + | NA |
| OTU2125 | NA | NA | + | NA |
| OTU18072 | NA | NA | NA | NA |
| OTU5022 | NA | NA | - | + |
| OTU7008 | NA | NA | NA | + |
| OTU13775 | + | NA | NA | NA |
| OTU18441 | + | NA | - | NA |
| OTU7447 | + | NA | - | NA |
| OTU11584 | NA | NA | NA | NA |
| OTU17954 | NA | NA | NA | NA |
| OTU18178 | + | - | NA | NA |
| OTU17758 | NA | - | NA | + |
| OTU8687 | - | NA | + | NA |
| OTU13827 | NA | NA | + | - |
| OTU8140 | + | - | NA | NA |
| OTU15016 | - | NA | NA | NA |
| OTU5036 | NA | NA | NA | NA |
| OTU4740 | + | NA | NA | NA |
| OTU14867 | - | NA | NA | NA |
| OTU12163 | - | + | NA | NA |
| OTU14351 | NA | NA | NA | NA |
| OTU17975 | NA | - | NA | + |
| OTU6639 | NA | NA | NA | + |
| OTU5863 | NA | NA | NA | NA |
| OTU87 | NA | NA | NA | NA |
| OTU16292 | - | + | NA | NA |
| OTU564 | NA | NA | NA | NA |
| OTU14921 | + | NA | NA | NA |
| OTU11724 | NA | - | NA | NA |
| OTU14773 | + | - | NA | NA |
| OTU9114 | NA | + | NA | NA |
| OTU17910 | + | NA | NA | NA |
| OTU11732 | + | NA | NA | NA |
| OTU5034 | - | + | NA | NA |
| OTU19139 | NA | NA | NA | NA |
| OTU18074 | NA | NA | - | + |
| OTU4098 | NA | NA | NA | NA |
| OTU5880 | NA | NA | - | NA |
| OTU8146 | + | NA | NA | NA |
| OTU15593 | NA | - | - | + |
| OTU19173 | + | NA | NA | NA |
| OTU6417 | NA | NA | - | + |
| OTU17743 | + | NA | NA | NA |
| OTU13165 | + | NA | - | NA |
| OTU18645 | NA | NA | NA | + |
| OTU6193 | NA | + | NA | NA |
| OTU17780 | NA | NA | + | - |
| OTU8045 | + | NA | NA | NA |
| OTU17703 | NA | - | NA | + |
| OTU14140 | + | NA | NA | NA |
| OTU13326 | NA | NA | + | NA |
| OTU2371 | NA | + | + | - |
| OTU776 | + | NA | - | NA |
| OTU13011 | + | NA | NA | NA |
| OTU15563 | NA | NA | NA | NA |
| OTU5873 | NA | NA | NA | NA |
| OTU13814 | + | NA | NA | NA |
| OTU15835 | NA | - | NA | + |
| OTU13217 | + | NA | NA | NA |
| OTU18432 | NA | - | NA | + |
| OTU15087 | - | NA | NA | + |
| OTU6680 | NA | NA | - | + |
| OTU18127 | + | NA | NA | NA |
| OTU19114 | NA | NA | NA | NA |
| OTU14991 | + | NA | NA | NA |
| OTU13833 | NA | NA | NA | NA |
| OTU7650 | NA | NA | NA | NA |
| OTU17736 | NA | NA | NA | NA |
| OTU14922 | NA | NA | + | - |
| OTU4939 | - | + | NA | NA |
| OTU13345 | NA | NA | - | + |
| OTU14871 | NA | NA | + | - |
| OTU15366 | + | - | NA | NA |
| OTU16731 | NA | NA | NA | NA |
| OTU2060 | NA | NA | NA | NA |
| OTU10571 | NA | NA | NA | NA |
| OTU11521 | + | NA | NA | NA |
| OTU17767 | NA | + | NA | - |
| OTU7919 | + | NA | NA | NA |
| OTU14923 | + | NA | NA | NA |
| OTU17584 | NA | NA | NA | NA |
| OTU2118 | NA | NA | + | NA |
| OTU3989 | + | NA | - | NA |
| OTU919 | + | NA | NA | NA |
| OTU18313 | NA | NA | NA | NA |
| OTU17834 | NA | NA | NA | - |
| OTU10994 | NA | NA | + | - |
| OTU17946 | NA | NA | NA | NA |
| OTU13036 | + | NA | - | NA |
| OTU2181 | NA | + | NA | NA |
| OTU16156 | + | NA | NA | NA |
| OTU14726 | - | NA | + | NA |
| OTU17977 | NA | + | NA | NA |
| OTU2902 | NA | NA | - | NA |
| OTU9867 | NA | NA | NA | NA |
| OTU5548 | NA | NA | NA | NA |
| OTU11280 | NA | + | NA | NA |
| OTU14187 | NA | NA | + | - |
| OTU18186 | NA | - | NA | NA |
| OTU14484 | NA | + | NA | - |
| OTU7580 | + | NA | NA | NA |
| OTU6141 | NA | + | NA | - |
| OTU13756 | NA | - | NA | NA |
| OTU2392 | + | NA | - | NA |
| OTU13361 | + | - | NA | + |
| OTU5977 | + | NA | NA | NA |
| OTU5515 | NA | - | NA | NA |
| OTU619 | + | - | NA | NA |
| OTU8796 | NA | NA | - | + |
| OTU3974 | NA | NA | + | - |
| OTU3449 | NA | - | NA | NA |
| OTU4714 | + | NA | NA | NA |
| OTU8289 | NA | NA | NA | + |
| OTU11195 | - | NA | + | NA |
| OTU16073 | NA | - | NA | + |
| OTU17811 | NA | NA | NA | + |
| OTU16215 | NA | NA | + | - |
| OTU8950 | + | NA | NA | NA |
| OTU13766 | NA | NA | NA | NA |
| OTU15845 | + | - | NA | NA |
| OTU11795 | NA | NA | NA | NA |
| OTU1559 | NA | - | NA | NA |
| OTU18005 | NA | NA | NA | NA |
| OTU13317 | NA | NA | NA | NA |
| OTU6327 | + | NA | - | NA |
| OTU17192 | + | NA | - | NA |
| OTU17157 | NA | NA | NA | NA |
| OTU13748 | NA | NA | NA | NA |
| OTU14457 | NA | NA | NA | + |
| OTU1705 | NA | - | NA | NA |
| OTU17983 | - | NA | + | NA |
| OTU6874 | NA | NA | NA | + |
| OTU18275 | NA | NA | - | + |
| OTU7788 | NA | NA | NA | NA |
| OTU13214 | - | + | NA | NA |
| OTU16143 | - | NA | NA | NA |
| OTU15798 | NA | NA | - | + |
| OTU2061 | - | NA | NA | NA |
| OTU7749 | NA | NA | NA | NA |
| OTU18201 | NA | NA | NA | NA |
| OTU4534 | NA | NA | - | NA |
| OTU6253 | NA | NA | NA | NA |
| OTU14300 | NA | NA | NA | NA |
| OTU17839 | - | NA | + | NA |
| OTU5122 | NA | NA | + | NA |
| OTU10516 | NA | NA | + | NA |
| OTU13113 | - | NA | NA | NA |
| OTU9315 | NA | - | NA | + |
| OTU9008 | NA | NA | NA | NA |
| OTU17575 | NA | NA | - | + |
| OTU13667 | NA | NA | NA | NA |
| OTU13787 | - | NA | + | NA |
| OTU5091 | NA | NA | + | - |
| OTU13275 | NA | NA | - | + |
| OTU6347 | + | NA | - | NA |
| OTU13192 | + | - | NA | NA |
| OTU14729 | NA | + | NA | - |
| OTU7579 | + | NA | - | NA |
| OTU13228 | - | NA | NA | NA |
| OTU1778 | - | NA | + | NA |
| OTU9895 | - | NA | + | NA |
| OTU2099 | NA | + | NA | NA |
| OTU9072 | NA | NA | NA | NA |
| OTU19146 | NA | - | NA | NA |
| OTU18041 | NA | NA | NA | + |
| OTU14946 | NA | NA | NA | NA |
| OTU951 | NA | NA | NA | NA |
| OTU3474 | + | - | NA | NA |
| OTU19117 | + | NA | - | NA |
| OTU1068 | + | NA | - | NA |
| OTU12884 | + | NA | NA | NA |
| OTU5693 | NA | NA | - | + |
| OTU13809 | NA | - | NA | NA |
| OTU12181 | - | NA | + | NA |
| OTU9220 | NA | NA | NA | NA |
| OTU1450 | NA | + | NA | - |
| OTU7376 | NA | NA | NA | - |
| OTU7611 | NA | NA | NA | NA |
| OTU4146 | - | NA | NA | NA |
| OTU13791 | + | NA | NA | NA |
| OTU13337 | NA | NA | NA | + |
| OTU5064 | + | NA | NA | NA |
| OTU16179 | - | + | NA | NA |
| OTU14509 | + | NA | - | NA |
| OTU14889 | + | NA | NA | NA |
| OTU17918 | NA | NA | - | + |
| OTU7326 | NA | NA | NA | NA |
| OTU13660 | + | - | - | NA |
| OTU4683 | NA | NA | NA | + |
| OTU15375 | NA | NA | NA | + |
| OTU7459 | NA | NA | + | - |
| OTU9562 | - | + | NA | NA |
| OTU17838 | NA | NA | + | NA |
| OTU2104 | NA | NA | NA | NA |
| OTU7482 | + | NA | - | NA |
| OTU8493 | NA | NA | NA | NA |
| OTU14746 | NA | NA | NA | NA |
| OTU3138 | NA | NA | - | NA |
| OTU6741 | NA | - | NA | NA |
| OTU17381 | NA | NA | - | NA |
| OTU7975 | - | + | NA | NA |
| OTU8152 | + | NA | NA | NA |
| OTU7584 | + | NA | - | NA |
| OTU3343 | + | NA | - | NA |
| OTU18029 | NA | - | NA | + |
| OTU7633 | NA | NA | NA | - |
| OTU17756 | NA | - | NA | + |
| OTU114 | NA | NA | NA | NA |
| OTU19164 | NA | NA | NA | NA |
| OTU18644 | + | NA | NA | NA |
| OTU17740 | NA | NA | NA | NA |
| OTU7254 | + | NA | - | NA |
| OTU7890 | NA | NA | NA | NA |
| OTU13051 | + | NA | NA | NA |
| OTU13795 | NA | - | + | NA |
| OTU13235 | NA | NA | NA | + |
| OTU18848 | NA | NA | NA | NA |
| OTU10875 | NA | NA | NA | NA |
| OTU7985 | + | NA | - | NA |
| OTU18068 | + | NA | NA | NA |
| OTU17931 | NA | NA | NA | NA |
| OTU18166 | NA | NA | - | + |
| OTU14951 | NA | NA | NA | NA |
| OTU6154 | NA | NA | - | + |
| OTU15645 | + | NA | NA | NA |
| OTU8166 | - | NA | NA | NA |
| OTU18268 | + | NA | - | NA |
| OTU14394 | NA | NA | NA | NA |
| OTU8919 | NA | NA | NA | NA |
| OTU17862 | NA | NA | NA | NA |
| OTU3056 | NA | + | NA | - |
| OTU13387 | + | NA | NA | NA |
| OTU17589 | - | NA | NA | + |
| OTU17613 | NA | NA | NA | NA |
| OTU14892 | + | NA | NA | NA |
| OTU17558 | NA | NA | NA | NA |
| OTU8740 | NA | NA | NA | NA |
| OTU9727 | + | NA | - | NA |
| OTU18050 | NA | NA | NA | NA |
| OTU13007 | NA | NA | NA | - |
| OTU3795 | NA | NA | NA | NA |
| OTU12971 | - | NA | NA | + |
| OTU7533 | NA | + | NA | - |
| OTU3635 | + | NA | - | NA |
| OTU5043 | NA | - | NA | + |
| OTU9818 | NA | NA | NA | NA |
| OTU1805 | NA | - | NA | NA |
| OTU3883 | + | NA | - | NA |
| OTU5081 | + | NA | NA | NA |
| OTU15029 | NA | - | NA | NA |
| OTU13264 | - | NA | + | NA |
| OTU3402 | NA | NA | NA | NA |
| OTU14105 | + | - | NA | NA |
| OTU14905 | NA | NA | NA | NA |
| OTU7469 | + | NA | - | NA |
| OTU11658 | NA | NA | NA | NA |
| OTU1900 | NA | NA | NA | NA |
| OTU14531 | NA | + | + | - |
| OTU11761 | - | NA | + | NA |
| OTU17603 | NA | + | NA | NA |
| OTU18007 | + | NA | - | NA |
| OTU6299 | NA | NA | NA | NA |
| OTU5390 | - | NA | + | NA |
| OTU4303 | NA | NA | + | NA |
| OTU11236 | + | - | NA | NA |
| OTU13372 | + | NA | NA | NA |
| OTU17552 | NA | NA | NA | NA |
| OTU2143 | - | NA | NA | NA |
| OTU18134 | NA | - | NA | + |
| OTU14914 | NA | NA | - | NA |
| OTU18392 | NA | NA | NA | + |
| OTU13266 | - | NA | NA | NA |
| OTU17904 | NA | NA | NA | NA |
| OTU8760 | NA | NA | + | NA |
| OTU5741 | NA | + | NA | - |
| OTU17687 | NA | NA | NA | NA |
| OTU5147 | NA | NA | + | - |
| OTU2042 | NA | NA | NA | + |
| OTU5676 | + | NA | - | NA |
| OTU3167 | + | - | NA | NA |
| OTU14665 | NA | NA | NA | NA |
| OTU15053 | NA | NA | NA | NA |
| OTU17949 | NA | NA | NA | + |
| OTU7596 | NA | + | NA | NA |
| OTU14780 | NA | NA | NA | + |
| OTU17200 | + | NA | - | NA |
| OTU2133 | + | NA | NA | NA |
| OTU10177 | NA | NA | NA | + |
| OTU7979 | NA | NA | NA | NA |
| OTU6669 | + | - | NA | NA |
| OTU16038 | NA | NA | - | NA |
| OTU17853 | NA | NA | NA | + |
| OTU1795 | + | NA | - | NA |
| OTU14510 | + | NA | NA | NA |
| OTU12480 | NA | NA | NA | NA |
| OTU17106 | NA | - | NA | NA |
| OTU17950 | NA | NA | - | + |
| OTU14732 | NA | + | NA | NA |
| OTU5858 | NA | NA | - | + |
| OTU9594 | NA | NA | NA | NA |
| OTU12025 | + | NA | - | NA |
| OTU17938 | NA | NA | - | + |
| OTU4432 | + | NA | - | NA |
| OTU7428 | NA | NA | + | NA |
| OTU1760 | + | NA | NA | NA |
| OTU9104 | + | - | - | NA |
| OTU2020 | NA | NA | NA | NA |
| OTU13331 | NA | + | NA | - |
| OTU654 | + | NA | - | NA |
| OTU14414 | - | NA | NA | NA |
| OTU12440 | + | NA | NA | NA |
| OTU17555 | NA | NA | NA | NA |
| OTU9325 | + | NA | NA | NA |
| OTU5742 | NA | NA | - | + |
| OTU6408 | NA | - | NA | + |
| OTU16982 | NA | - | NA | + |
| OTU12145 | NA | NA | NA | - |
| OTU8718 | NA | NA | NA | NA |
| OTU7296 | NA | NA | NA | + |
| OTU13008 | NA | NA | NA | NA |
| OTU13074 | NA | NA | NA | NA |
| OTU15039 | NA | NA | + | - |
| OTU6281 | NA | NA | NA | NA |
| OTU11872 | NA | NA | NA | NA |
| OTU18856 | NA | NA | NA | NA |
| OTU13373 | NA | NA | NA | NA |
| OTU8942 | + | NA | - | NA |
| OTU15755 | + | NA | NA | NA |
| OTU13520 | NA | NA | NA | - |
| OTU10982 | - | NA | NA | NA |
| OTU13367 | NA | NA | - | + |
| OTU14571 | NA | NA | NA | NA |
| OTU15049 | NA | NA | NA | - |
| OTU14654 | NA | NA | + | NA |
| OTU14757 | NA | NA | - | + |
| OTU5684 | + | NA | - | NA |
| OTU14607 | + | NA | - | NA |
| OTU2559 | + | NA | NA | NA |
| OTU17746 | NA | NA | NA | NA |
| OTU13867 | NA | NA | NA | NA |
| OTU13149 | + | NA | NA | NA |
| OTU14721 | NA | NA | - | NA |
| OTU2102 | NA | NA | NA | - |
| OTU18096 | NA | NA | - | + |
| OTU7799 | + | NA | NA | NA |
| OTU12249 | NA | NA | NA | NA |
| OTU14021 | NA | NA | NA | NA |
| OTU13442 | NA | NA | NA | NA |
| OTU17798 | + | NA | NA | NA |
| OTU5317 | NA | - | NA | NA |
| OTU13510 | NA | NA | NA | NA |
| OTU17762 | NA | NA | NA | NA |
| OTU12899 | - | + | NA | NA |
| OTU4260 | + | NA | - | NA |
| OTU14594 | NA | NA | NA | NA |
| OTU2127 | - | NA | NA | NA |
| OTU17578 | NA | - | NA | NA |
| OTU16860 | NA | NA | NA | NA |
| OTU15438 | NA | NA | NA | NA |
| OTU18031 | NA | NA | NA | NA |
| OTU17991 | NA | NA | NA | NA |
| OTU11107 | NA | NA | NA | NA |
| OTU1723 | - | NA | NA | NA |
| OTU1292 | NA | NA | NA | NA |
| OTU9534 | NA | NA | - | NA |
| OTU15080 | NA | NA | NA | NA |
| OTU1501 | NA | NA | NA | NA |
| OTU4505 | NA | NA | NA | NA |
| OTU13318 | NA | NA | NA | NA |
| OTU17852 | NA | NA | + | - |
| OTU13658 | NA | NA | NA | NA |
| OTU17774 | - | NA | NA | + |
| OTU7843 | + | NA | NA | NA |
| OTU13767 | + | NA | NA | NA |
| OTU11447 | NA | NA | NA | NA |
| OTU15066 | NA | NA | NA | NA |
| OTU1171 | + | NA | NA | NA |
| OTU5765 | NA | NA | NA | NA |
| OTU17987 | NA | NA | - | + |
| OTU2114 | NA | NA | NA | NA |
| OTU17512 | NA | - | NA | + |
| OTU3773 | + | NA | - | NA |
| OTU18057 | - | NA | NA | NA |
| OTU13892 | + | NA | NA | NA |
| OTU18093 | - | + | NA | NA |
| OTU17825 | NA | NA | NA | + |
| OTU7768 | + | NA | NA | NA |
| OTU1146 | NA | NA | - | NA |
| OTU18282 | NA | NA | NA | NA |
| OTU1834 | NA | NA | NA | + |
| OTU14203 | - | NA | + | NA |
| OTU15091 | NA | NA | NA | NA |
| OTU7408 | + | NA | - | NA |
| OTU17696 | + | NA | - | NA |
| OTU2205 | + | - | NA | - |
| OTU13079 | NA | - | + | NA |
| OTU2872 | NA | NA | - | NA |
| OTU12634 | NA | NA | NA | - |
| OTU7747 | NA | NA | - | NA |
| OTU17995 | NA | NA | - | + |
| OTU13292 | NA | NA | + | - |
| OTU6449 | NA | NA | NA | - |
| OTU12208 | NA | - | NA | NA |
| OTU14427 | NA | NA | + | - |
| OTU5660 | NA | NA | NA | NA |
| OTU3731 | NA | NA | - | + |
| OTU13327 | NA | NA | NA | NA |
| OTU13594 | NA | - | NA | + |
| OTU13033 | NA | + | NA | NA |
| OTU8731 | - | + | NA | NA |
| OTU9970 | NA | NA | + | NA |
| OTU18101 | NA | NA | NA | NA |
| OTU13424 | NA | NA | NA | - |
| OTU7731 | NA | NA | - | + |
| OTU915 | + | - | NA | NA |
| OTU18188 | NA | NA | - | NA |
| OTU17843 | NA | NA | NA | NA |
| OTU17518 | + | NA | NA | NA |
| OTU16522 | NA | NA | NA | NA |
| OTU7115 | NA | NA | NA | NA |
| OTU13931 | NA | NA | - | NA |
| OTU14593 | NA | NA | - | NA |
| OTU1534 | + | NA | - | NA |
| OTU13556 | NA | NA | NA | + |
| OTU6301 | + | NA | - | NA |
| OTU18692 | NA | NA | NA | + |
| OTU18250 | + | NA | - | NA |
| OTU11896 | NA | NA | NA | NA |
| OTU1265 | + | NA | NA | NA |
| OTU16619 | - | NA | + | NA |
| OTU14811 | NA | NA | + | - |
| OTU278 | NA | NA | NA | + |
| OTU3296 | + | NA | NA | NA |
| OTU18438 | NA | - | NA | + |
| OTU6344 | NA | NA | + | NA |
| OTU14583 | - | NA | NA | NA |
| OTU6146 | NA | + | NA | NA |
| OTU9669 | - | NA | NA | NA |
| OTU18398 | NA | NA | - | + |
| OTU13729 | NA | NA | - | NA |
| OTU8668 | NA | NA | NA | NA |
| OTU1966 | NA | NA | - | + |
| OTU5922 | NA | + | NA | - |
| OTU14779 | NA | + | NA | NA |
| OTU6317 | + | NA | - | NA |
| OTU6376 | NA | NA | NA | - |
| OTU15575 | NA | NA | NA | NA |
| OTU18261 | NA | - | NA | + |
| OTU15068 | + | NA | NA | NA |
| OTU9631 | NA | NA | - | NA |
| OTU18089 | + | NA | NA | - |
| OTU13904 | NA | NA | NA | NA |
| OTU14386 | + | NA | NA | NA |
| OTU335 | NA | NA | NA | NA |
| OTU8376 | + | NA | - | NA |
| OTU1867 | NA | NA | NA | NA |
| OTU3599 | NA | NA | NA | NA |
| OTU2136 | - | NA | NA | NA |
| OTU778 | NA | NA | NA | NA |
| OTU435 | NA | NA | - | NA |
| OTU2083 | - | NA | NA | + |
| OTU14723 | - | NA | NA | NA |
| OTU13097 | + | NA | - | NA |
| OTU7894 | + | NA | - | NA |
| OTU13256 | NA | NA | NA | NA |
| OTU14488 | NA | NA | NA | + |
| OTU15751 | NA | NA | NA | NA |
| OTU14236 | + | NA | NA | NA |
| OTU4550 | NA | NA | NA | NA |
| OTU14012 | NA | NA | NA | NA |
| OTU9864 | - | + | NA | NA |
| OTU10121 | NA | NA | NA | + |
| OTU9363 | + | NA | NA | NA |
| OTU2512 | NA | - | NA | + |
| OTU19021 | NA | - | NA | + |
| OTU2451 | + | - | NA | NA |
| OTU7646 | NA | + | NA | - |
| OTU9868 | - | NA | + | NA |
| OTU16438 | NA | NA | NA | NA |
| OTU13130 | NA | NA | NA | - |
| OTU4662 | - | NA | NA | NA |
| OTU13159 | NA | NA | + | - |
| OTU13158 | + | NA | - | NA |
| OTU7726 | + | NA | NA | NA |
| OTU18176 | NA | NA | NA | NA |
| OTU18300 | NA | NA | NA | NA |
| OTU14680 | + | - | NA | NA |
| OTU9132 | NA | NA | + | - |
| OTU14798 | NA | NA | NA | - |
| OTU7360 | + | NA | - | NA |
| OTU7468 | + | NA | - | NA |
| OTU12701 | - | NA | NA | NA |
| OTU15253 | NA | NA | NA | + |
| OTU13834 | NA | NA | NA | NA |
| OTU9300 | NA | NA | NA | NA |
| OTU16054 | + | NA | NA | NA |
| OTU13368 | + | NA | NA | NA |
| OTU1513 | + | NA | NA | NA |
| OTU17867 | - | NA | + | NA |
| OTU9593 | + | NA | NA | NA |
| OTU6267 | NA | NA | NA | NA |
| OTU4537 | NA | NA | NA | NA |
| OTU6260 | NA | NA | NA | - |
| OTU6285 | + | NA | NA | - |
| OTU14005 | NA | NA | NA | NA |
| OTU14371 | NA | NA | NA | NA |
| OTU5997 | NA | NA | NA | NA |
| OTU438 | + | NA | - | NA |
| OTU2130 | - | + | NA | NA |
| OTU1882 | + | NA | NA | NA |
| OTU17103 | NA | NA | - | + |
| OTU8357 | NA | NA | NA | NA |
| OTU17636 | NA | + | NA | NA |
| OTU13101 | NA | NA | - | + |
| OTU1137 | NA | + | NA | NA |
| OTU18105 | NA | + | NA | NA |
| OTU4999 | NA | NA | + | NA |
| OTU13215 | NA | NA | NA | NA |
| OTU1323 | + | NA | NA | NA |
| OTU14968 | NA | NA | - | + |
| OTU1710 | NA | NA | NA | NA |
| OTU13173 | NA | NA | NA | NA |
| OTU943 | + | NA | - | NA |
| OTU19058 | + | NA | NA | NA |
| OTU13263 | + | + | NA | NA |
| OTU18621 | + | NA | - | NA |
| OTU866 | NA | NA | + | NA |
| OTU7330 | NA | - | NA | NA |
| OTU18420 | NA | + | NA | - |
| OTU7770 | + | - | - | NA |
| OTU9163 | - | + | NA | NA |
| OTU17600 | NA | NA | + | - |
| OTU18306 | NA | + | NA | NA |
| OTU17565 | - | NA | - | NA |
| OTU18379 | NA | NA | NA | NA |
| OTU18180 | NA | NA | - | + |
| OTU9174 | - | + | NA | NA |
| OTU14932 | NA | NA | NA | NA |
| OTU14584 | + | NA | NA | NA |
| OTU14731 | NA | NA | NA | + |
| OTU17345 | - | NA | + | NA |
| OTU3674 | - | NA | + | NA |
| OTU583 | NA | NA | - | + |
| OTU5743 | NA | NA | NA | + |
| OTU8856 | + | NA | - | NA |
| OTU3184 | + | NA | - | NA |
| OTU4611 | - | + | NA | NA |
| OTU1013 | NA | NA | NA | NA |
| OTU6279 | + | NA | - | NA |
| OTU10075 | + | NA | NA | NA |
| OTU6343 | NA | NA | NA | - |
| OTU15009 | NA | NA | + | NA |
| OTU14564 | NA | NA | NA | NA |
| OTU4708 | NA | + | NA | NA |
| OTU14872 | + | NA | NA | NA |
| OTU8987 | NA | NA | NA | NA |
| OTU14555 | NA | NA | NA | NA |
| OTU3350 | NA | NA | NA | - |
| OTU6468 | NA | - | NA | NA |
| OTU1348 | + | NA | - | NA |
| OTU6034 | + | NA | - | NA |
| OTU10279 | - | NA | + | NA |
| OTU18142 | NA | NA | NA | + |
| OTU9846 | NA | NA | + | - |
| OTU9606 | NA | NA | + | NA |
| OTU9598 | NA | + | NA | NA |
| OTU13942 | + | NA | NA | NA |
| OTU732 | NA | NA | NA | - |
| OTU1839 | + | NA | - | NA |
| OTU16721 | + | NA | NA | NA |
| OTU1346 | NA | + | NA | - |
| OTU10432 | NA | - | NA | NA |
| OTU7969 | NA | NA | NA | NA |
| OTU10232 | + | NA | - | NA |
| OTU17937 | NA | NA | NA | NA |
| OTU13243 | - | NA | + | NA |
| OTU9940 | - | NA | NA | + |
| OTU9567 | NA | NA | NA | + |
| OTU11682 | NA | NA | + | NA |
| OTU992 | NA | NA | - | NA |
| OTU18078 | - | NA | NA | NA |
| OTU17301 | NA | NA | - | + |
| OTU14859 | NA | NA | NA | NA |
| OTU7499 | NA | NA | NA | NA |
| OTU2126 | NA | NA | + | - |
| OTU18060 | NA | NA | NA | NA |
| OTU1740 | NA | NA | NA | + |
| OTU1010 | NA | NA | NA | + |
| OTU15507 | NA | NA | + | NA |
| OTU9316 | + | NA | NA | NA |
| OTU16262 | NA | NA | NA | NA |
| OTU393 | NA | - | NA | + |
| OTU12659 | NA | NA | + | NA |
| OTU18262 | NA | NA | NA | NA |
| OTU1914 | NA | NA | NA | NA |
| OTU972 | + | NA | - | NA |
| OTU18924 | NA | NA | NA | + |
| OTU2656 | + | - | NA | NA |
| OTU15894 | NA | NA | NA | NA |
| OTU13110 | - | NA | NA | NA |
| OTU13765 | NA | NA | NA | NA |
| OTU13272 | + | NA | NA | NA |
| OTU14850 | NA | NA | NA | NA |
| OTU12546 | NA | NA | NA | NA |
| OTU14795 | - | NA | + | NA |
| OTU15145 | NA | - | NA | NA |
| OTU5245 | - | + | NA | NA |
| OTU5072 | NA | NA | + | - |
| OTU96 | NA | NA | NA | NA |
| OTU3272 | + | NA | NA | NA |
| OTU937 | - | NA | NA | NA |
| OTU13819 | - | NA | NA | + |
| OTU9518 | + | NA | NA | NA |
| OTU7509 | + | NA | - | NA |
| OTU13366 | + | NA | NA | NA |
| OTU9661 | NA | NA | NA | + |
| OTU18266 | - | NA | + | - |
| OTU4696 | NA | NA | + | NA |
| OTU7634 | NA | + | NA | NA |
| OTU14708 | - | + | NA | NA |
| OTU17930 | NA | NA | NA | - |
| OTU9983 | - | NA | NA | NA |
| OTU9714 | NA | NA | NA | - |
| OTU14818 | NA | NA | NA | NA |
| OTU6008 | NA | NA | - | NA |
| OTU18461 | NA | NA | + | NA |
| OTU17936 | NA | NA | NA | NA |
| OTU18416 | NA | NA | NA | - |
| OTU437 | + | NA | NA | NA |
| OTU14408 | - | NA | NA | + |
| OTU14771 | NA | NA | NA | NA |
| OTU13059 | NA | - | NA | NA |
| OTU13147 | NA | NA | NA | NA |
| OTU8775 | NA | NA | - | NA |
| OTU8793 | + | NA | - | NA |
| OTU901 | + | NA | - | NA |
| OTU18380 | + | - | NA | NA |
| OTU13713 | NA | NA | NA | NA |
| OTU14622 | + | NA | - | NA |
| OTU15485 | NA | - | NA | + |
| OTU1445 | + | NA | NA | NA |
| OTU9167 | NA | NA | NA | NA |
| OTU8999 | NA | NA | NA | NA |
| OTU6378 | NA | NA | NA | + |
| OTU1218 | NA | NA | NA | NA |
| OTU11767 | - | NA | + | NA |
| OTU802 | + | NA | NA | NA |
| OTU14925 | + | NA | NA | NA |
| OTU17585 | NA | NA | NA | NA |
| OTU16009 | + | NA | NA | NA |
| OTU5059 | + | NA | NA | NA |
| OTU13229 | + | NA | - | NA |
| OTU18258 | NA | NA | - | + |
| OTU9242 | NA | NA | NA | NA |
| OTU7667 | + | NA | - | NA |
| OTU14480 | + | NA | NA | NA |
| OTU4218 | - | NA | + | NA |
| OTU1933 | + | - | NA | NA |
| OTU9131 | NA | NA | + | NA |
| OTU2171 | NA | NA | NA | NA |
| OTU7769 | NA | NA | NA | + |
| OTU14672 | - | NA | + | NA |
| OTU14528 | NA | NA | NA | NA |
| OTU4532 | - | + | NA | NA |
| OTU11186 | NA | + | NA | - |
| OTU13746 | NA | NA | NA | NA |
| OTU6296 | NA | NA | NA | NA |
| OTU17997 | - | + | NA | NA |
| OTU17571 | - | NA | + | NA |
| OTU17546 | NA | NA | NA | NA |
| OTU14336 | NA | NA | NA | NA |
| OTU14638 | NA | NA | - | + |
| OTU14312 | + | NA | NA | NA |
| OTU19069 | + | NA | - | NA |
| OTU13277 | + | NA | NA | NA |
| OTU18304 | + | NA | NA | NA |
| OTU14927 | + | NA | - | NA |
| OTU913 | + | NA | - | NA |
| OTU14808 | NA | NA | NA | NA |
| OTU7681 | NA | NA | NA | NA |
| OTU6020 | NA | NA | NA | - |
| OTU620 | + | NA | NA | NA |
| OTU18113 | NA | NA | + | - |
| OTU7546 | NA | NA | NA | NA |
| OTU10424 | + | NA | NA | NA |
| OTU6320 | - | + | NA | NA |
| OTU14958 | NA | NA | + | NA |
| OTU1099 | NA | NA | - | NA |
| OTU14860 | NA | NA | + | - |
| OTU3639 | NA | NA | NA | NA |
| OTU13198 | NA | NA | NA | NA |
| OTU13740 | NA | - | NA | + |
| OTU14624 | NA | NA | NA | NA |
| OTU3657 | NA | NA | + | NA |
| OTU6236 | NA | NA | NA | NA |
| OTU18001 | NA | NA | - | + |
| OTU6349 | - | + | NA | NA |
| OTU17795 | NA | NA | NA | NA |
| OTU9277 | NA | NA | NA | - |
| OTU18324 | - | NA | NA | NA |
| OTU2569 | + | NA | - | NA |
| OTU4238 | - | NA | NA | + |
| OTU14296 | + | NA | NA | NA |
| OTU2632 | NA | NA | + | - |
| OTU13055 | + | NA | NA | NA |
| OTU11823 | NA | NA | + | NA |
| OTU13902 | NA | NA | NA | NA |
| OTU13306 | NA | NA | - | + |
| OTU13253 | NA | - | NA | NA |
| OTU13781 | NA | NA | NA | NA |
| OTU18214 | NA | NA | - | NA |
| OTU13820 | NA | NA | NA | NA |
| OTU9585 | NA | NA | NA | NA |
| OTU1036 | NA | NA | + | - |
| OTU3267 | + | NA | - | NA |
| OTU10989 | NA | NA | NA | NA |
| OTU1407 | NA | NA | NA | NA |
| OTU14310 | NA | NA | + | NA |
| OTU7620 | + | - | NA | NA |
| OTU7598 | + | NA | NA | NA |
| OTU14585 | + | NA | NA | NA |
| OTU14439 | NA | NA | + | - |
| OTU18813 | NA | NA | NA | NA |
| OTU4540 | NA | NA | NA | NA |
| OTU15032 | NA | NA | NA | + |
| OTU14641 | - | NA | NA | NA |
| OTU17990 | + | NA | NA | NA |
| OTU13507 | NA | NA | NA | NA |
| OTU3887 | NA | NA | NA | NA |
| OTU13980 | NA | NA | NA | NA |
| OTU7479 | + | NA | - | NA |
| OTU12556 | NA | NA | NA | NA |
| OTU18883 | NA | NA | NA | + |
| OTU14517 | NA | NA | NA | NA |
| OTU14606 | NA | NA | NA | NA |
| OTU17342 | + | - | NA | NA |
| OTU14424 | - | NA | + | NA |
| OTU18083 | - | + | - | NA |
| OTU11539 | NA | NA | + | - |
| OTU6412 | + | NA | - | NA |
| OTU17821 | NA | NA | NA | NA |
| OTU638 | + | NA | - | NA |
| OTU13163 | NA | - | + | NA |
| OTU13550 | NA | + | NA | NA |
| OTU2115 | NA | NA | + | NA |
| OTU19112 | NA | NA | - | + |
| OTU18128 | NA | NA | NA | NA |
| OTU2119 | NA | NA | NA | NA |
| OTU8702 | NA | - | + | NA |
| OTU18263 | NA | - | NA | NA |
| OTU9309 | NA | NA | NA | NA |
| OTU4597 | NA | NA | NA | + |
| OTU13330 | + | NA | NA | NA |
| OTU14385 | NA | + | NA | - |
| OTU3918 | NA | NA | NA | NA |
| OTU13283 | NA | + | NA | NA |
| OTU5266 | NA | NA | NA | + |
| OTU17826 | + | NA | NA | NA |
| OTU10439 | - | NA | NA | + |
| OTU6300 | + | NA | NA | NA |
| OTU11633 | NA | NA | NA | NA |
| OTU6404 | + | NA | - | NA |
| OTU3288 | + | NA | - | NA |
| OTU14931 | NA | NA | + | NA |
| OTU9331 | + | - | NA | NA |
| OTU5777 | NA | NA | NA | NA |
| OTU9318 | NA | NA | NA | NA |
| OTU15828 | NA | NA | NA | + |
| OTU15033 | NA | NA | NA | NA |
| OTU7063 | + | NA | NA | NA |
| OTU8246 | + | NA | NA | NA |
| OTU9654 | + | - | NA | NA |
| OTU14598 | NA | + | NA | NA |
| OTU13830 | + | NA | NA | NA |
| OTU13230 | NA | NA | NA | NA |
| OTU2131 | - | + | NA | NA |
| OTU12285 | NA | NA | + | - |
| OTU947 | + | NA | NA | NA |
| OTU13066 | NA | NA | NA | + |
| OTU4367 | NA | NA | - | NA |
| OTU3880 | NA | NA | NA | NA |
| OTU5779 | NA | NA | NA | NA |
| OTU10391 | NA | NA | NA | - |
| OTU6465 | NA | NA | NA | NA |
| OTU7500 | + | NA | - | NA |
| OTU17730 | NA | NA | NA | NA |
| OTU17917 | NA | NA | + | NA |
| OTU2071 | NA | NA | NA | NA |
| OTU9056 | NA | NA | NA | NA |
| OTU11427 | + | - | NA | NA |
| OTU18273 | NA | NA | NA | NA |
| OTU17387 | NA | NA | NA | + |
| OTU12851 | NA | NA | NA | NA |
| OTU14302 | + | NA | NA | NA |
| OTU19023 | NA | NA | NA | NA |
| OTU10174 | NA | + | NA | NA |
| OTU2887 | + | NA | - | NA |
| OTU7372 | + | NA | NA | NA |
| OTU9913 | - | NA | - | + |
| OTU18370 | NA | NA | NA | NA |
| OTU13156 | + | NA | - | NA |
| OTU15004 | NA | NA | NA | NA |
| OTU14465 | NA | + | NA | NA |
| OTU3766 | NA | NA | NA | NA |
| OTU6118 | NA | NA | NA | + |
| OTU7637 | + | NA | - | NA |
| OTU16615 | NA | NA | NA | NA |
| OTU6826 | + | - | NA | NA |
| OTU13013 | NA | NA | NA | NA |
| OTU8425 | NA | + | - | NA |
| OTU9684 | NA | NA | + | NA |
| OTU13720 | + | NA | NA | NA |
| OTU13086 | NA | NA | NA | NA |
| OTU11672 | NA | - | NA | NA |
| OTU14832 | + | NA | - | NA |
| OTU1821 | NA | NA | NA | NA |
| OTU14745 | - | NA | NA | NA |
| OTU17745 | NA | NA | NA | NA |
| OTU7916 | NA | NA | + | - |
| OTU17948 | NA | NA | NA | NA |
| OTU5884 | - | + | NA | NA |
| OTU13665 | + | NA | NA | NA |
| OTU1592 | NA | NA | NA | NA |
| OTU3811 | NA | + | NA | NA |
| OTU11976 | NA | NA | NA | NA |
| OTU17808 | NA | NA | - | + |
| OTU14802 | - | NA | NA | NA |
| OTU9205 | NA | NA | NA | - |
| OTU18265 | NA | NA | NA | NA |
| OTU14516 | NA | NA | NA | NA |
| OTU17667 | + | NA | NA | NA |
| OTU6594 | NA | NA | NA | NA |
| OTU11803 | + | NA | - | NA |
| OTU14497 | NA | NA | NA | NA |
| OTU3756 | NA | NA | NA | NA |
| OTU17622 | NA | NA | NA | + |
| OTU15166 | NA | - | NA | + |
| OTU562 | NA | NA | NA | NA |
| OTU19171 | NA | NA | NA | NA |
| OTU18017 | NA | NA | NA | NA |
| OTU6847 | NA | NA | NA | NA |
| OTU18116 | - | NA | NA | NA |
| OTU7759 | NA | NA | NA | NA |
| OTU1879 | - | NA | + | NA |
| OTU13398 | + | NA | NA | + |
| OTU14416 | + | NA | NA | NA |
| OTU63 | + | NA | NA | NA |
| OTU17898 | NA | NA | NA | NA |
| OTU1307 | NA | NA | NA | NA |
| OTU10103 | - | NA | NA | NA |
| OTU12290 | NA | NA | + | - |
| OTU2782 | NA | NA | NA | NA |
| OTU13560 | NA | NA | - | NA |
| OTU7675 | NA | + | NA | NA |
| OTU8019 | + | NA | NA | NA |
| OTU317 | + | NA | NA | NA |
| OTU4654 | NA | NA | - | NA |
| OTU6569 | NA | NA | - | + |
| OTU17905 | NA | NA | NA | + |
| OTU17818 | NA | NA | NA | + |
| OTU16859 | - | NA | NA | NA |
| OTU1827 | NA | NA | NA | + |
| OTU13091 | NA | NA | NA | - |
| OTU1306 | + | NA | NA | NA |
| OTU3363 | NA | NA | NA | - |
| OTU6346 | + | - | NA | NA |
| OTU18095 | NA | NA | NA | + |
| OTU4452 | NA | NA | - | NA |
| OTU397 | + | NA | NA | NA |
| OTU1681 | NA | NA | NA | + |
| OTU13564 | + | - | NA | NA |
| OTU17528 | NA | NA | NA | + |
| OTU4240 | NA | NA | NA | + |
| OTU17522 | NA | NA | + | - |
| OTU14894 | NA | NA | NA | - |
| OTU13840 | NA | NA | - | NA |
| OTU9154 | + | NA | - | NA |
| OTU9441 | + | NA | NA | NA |
| OTU9961 | NA | NA | - | + |
| OTU9096 | + | NA | NA | NA |
| OTU18354 | NA | NA | NA | + |
| OTU5809 | + | - | NA | NA |
| OTU3758 | + | NA | NA | NA |
| OTU17725 | - | NA | NA | NA |
| OTU1973 | NA | NA | + | NA |
| OTU13188 | NA | NA | NA | NA |
| OTU7523 | + | NA | NA | NA |
| OTU3531 | + | NA | NA | NA |
| OTU6659 | NA | NA | - | NA |
| OTU828 | NA | + | NA | NA |
| OTU17735 | - | NA | NA | NA |
| OTU16988 | NA | NA | NA | + |
| OTU11920 | + | NA | NA | NA |
| OTU981 | + | - | NA | NA |
| OTU18302 | NA | NA | - | NA |
| OTU1963 | + | NA | - | NA |
| OTU18193 | NA | - | NA | + |
| OTU2379 | + | NA | NA | NA |
| OTU10351 | + | NA | - | NA |
| OTU15055 | NA | NA | NA | NA |
| OTU1669 | NA | NA | NA | NA |
| OTU18264 | NA | NA | NA | NA |
| OTU12050 | NA | NA | + | - |
| OTU16749 | + | NA | - | NA |
| OTU3951 | NA | NA | + | NA |
| OTU18192 | NA | NA | - | NA |
| OTU2185 | NA | NA | NA | NA |
| OTU17698 | NA | NA | + | NA |
| OTU11504 | NA | NA | + | - |
| OTU13189 | NA | NA | + | - |
| OTU13271 | + | NA | NA | NA |
| OTU1761 | NA | NA | NA | NA |
| OTU12953 | NA | NA | - | NA |
| OTU5572 | NA | NA | NA | NA |
| OTU4687 | + | NA | NA | NA |
| OTU14693 | NA | NA | NA | NA |
| OTU17739 | NA | NA | NA | NA |
| OTU11163 | NA | NA | NA | NA |
| OTU9180 | + | NA | NA | NA |
| OTU2327 | NA | NA | NA | NA |
| OTU18338 | NA | NA | NA | NA |
| OTU806 | + | NA | - | NA |
| OTU18421 | NA | NA | NA | + |
| OTU19016 | NA | NA | NA | NA |
| OTU13714 | + | - | NA | NA |
| OTU7602 | NA | NA | NA | - |
| OTU1000 | + | - | NA | NA |
| OTU1404 | + | NA | - | NA |
| OTU7625 | + | NA | NA | NA |
| OTU13022 | NA | NA | + | NA |
| OTU17671 | NA | NA | NA | + |
| OTU14442 | NA | + | - | NA |
| OTU5260 | - | NA | + | NA |
| OTU14623 | - | NA | NA | NA |
| OTU11915 | + | NA | NA | NA |
| OTU6471 | NA | NA | NA | NA |
| OTU10008 | NA | NA | NA | NA |
| OTU14595 | NA | NA | NA | NA |
| OTU15387 | - | NA | NA | NA |
| OTU5107 | NA | NA | NA | NA |
| OTU7592 | + | - | NA | NA |
| OTU164 | + | NA | NA | NA |
| OTU18206 | NA | NA | NA | NA |
| OTU1401 | + | NA | - | NA |
| OTU17845 | - | NA | + | NA |
| OTU9842 | NA | NA | NA | NA |
| OTU3813 | NA | NA | NA | NA |
| OTU754 | NA | NA | NA | NA |
| OTU18170 | + | NA | NA | NA |
| OTU2651 | + | NA | NA | NA |
| OTU13853 | NA | NA | NA | NA |
| OTU6717 | NA | NA | NA | NA |
| OTU19067 | NA | NA | NA | - |
| OTU14986 | NA | - | NA | NA |
| OTU2369 | + | NA | NA | NA |
| OTU17912 | - | NA | + | NA |
| OTU17890 | NA | NA | NA | + |
| OTU1660 | NA | NA | NA | + |
| OTU5040 | NA | NA | NA | NA |
| OTU13688 | + | NA | NA | NA |
| OTU5791 | NA | + | NA | NA |
| OTU14410 | + | NA | NA | NA |
| OTU7544 | NA | + | NA | - |
| OTU11690 | NA | NA | NA | NA |
| OTU5675 | NA | NA | NA | - |
| OTU6763 | NA | NA | NA | NA |
| OTU7525 | + | NA | NA | NA |
| OTU5446 | - | NA | NA | + |
| OTU13353 | NA | NA | + | NA |
| OTU8049 | NA | NA | NA | NA |
| OTU14677 | NA | NA | + | NA |
| OTU14988 | NA | NA | NA | NA |
| OTU11860 | NA | NA | + | NA |
| OTU12142 | NA | NA | NA | + |
| OTU514 | NA | NA | - | NA |
| OTU7492 | + | NA | - | NA |
| OTU15118 | NA | NA | - | + |
| OTU13357 | + | NA | - | NA |
| OTU15011 | NA | NA | NA | NA |
| OTU8430 | + | - | NA | NA |
| OTU11590 | + | NA | NA | NA |
| OTU14720 | NA | NA | NA | NA |
| OTU7364 | - | + | NA | NA |
| OTU1654 | NA | NA | NA | NA |
| OTU8995 | NA | NA | NA | NA |
| OTU15246 | - | NA | NA | NA |
| OTU14383 | + | NA | NA | NA |
| OTU18357 | NA | NA | NA | NA |
| OTU11620 | NA | NA | NA | NA |
| OTU5816 | + | NA | NA | - |
| OTU2117 | NA | NA | NA | NA |
| OTU9624 | NA | NA | NA | NA |
| OTU7566 | + | NA | NA | NA |
| OTU16398 | NA | - | NA | NA |
| OTU5805 | NA | NA | NA | NA |
| OTU17697 | NA | NA | + | NA |
| OTU17764 | NA | NA | NA | NA |
| OTU3597 | + | NA | - | NA |
| OTU5570 | + | - | NA | NA |
| OTU14413 | NA | NA | NA | NA |
| OTU6355 | + | NA | NA | NA |
| OTU19120 | + | NA | - | NA |
| OTU13351 | NA | NA | NA | NA |
| OTU14917 | NA | NA | NA | NA |
| OTU3045 | NA | - | NA | NA |
| OTU13461 | NA | + | NA | NA |
| OTU9668 | NA | NA | + | NA |
| OTU6345 | NA | NA | - | + |
| OTU3806 | NA | NA | + | NA |
| OTU13813 | NA | NA | + | - |
| OTU2891 | + | NA | NA | NA |
| OTU14713 | NA | NA | NA | NA |
| OTU12461 | NA | NA | NA | NA |
| OTU4617 | - | NA | NA | NA |
| OTU16408 | NA | NA | NA | NA |
| OTU13024 | NA | NA | + | NA |
| OTU1584 | + | NA | NA | NA |
| OTU14013 | NA | NA | NA | NA |
| OTU10797 | NA | NA | + | NA |
| OTU15089 | NA | NA | NA | NA |
| OTU7503 | + | NA | - | NA |
| OTU9190 | NA | NA | - | NA |
| OTU13146 | NA | NA | NA | NA |
| OTU17533 | - | + | NA | NA |
| OTU2156 | NA | NA | NA | NA |
| OTU17933 | NA | NA | NA | NA |
| OTU4836 | NA | NA | NA | NA |
| OTU15117 | NA | NA | NA | NA |
| OTU13871 | + | NA | NA | NA |
| OTU4519 | + | NA | NA | NA |
| OTU18026 | + | - | NA | NA |
| OTU7368 | NA | NA | NA | + |
| OTU2128 | NA | NA | NA | NA |
| OTU17939 | NA | + | NA | NA |
| OTU8224 | NA | NA | NA | NA |
| OTU3300 | NA | NA | NA | - |
| OTU2375 | NA | NA | - | NA |
| OTU19077 | + | NA | - | NA |
| OTU13811 | + | NA | NA | NA |
| OTU11132 | NA | NA | NA | - |
| OTU7302 | NA | NA | NA | NA |
| OTU5831 | - | + | NA | NA |
| OTU13027 | + | NA | NA | NA |
| OTU17402 | NA | NA | + | NA |
| OTU16715 | NA | NA | NA | NA |
| OTU12344 | NA | NA | NA | + |
| OTU3762 | + | - | NA | NA |
| OTU18427 | NA | + | NA | - |
| OTU9555 | - | NA | NA | NA |
| OTU17921 | NA | NA | + | NA |
| OTU32 | + | NA | NA | NA |
| OTU13997 | NA | + | - | NA |
| OTU7639 | + | NA | - | NA |
| OTU17783 | NA | NA | NA | NA |
| OTU15429 | NA | NA | - | + |
| OTU18274 | + | NA | NA | NA |
| OTU3302 | + | NA | NA | NA |
| OTU14321 | - | NA | NA | NA |
| OTU14588 | NA | NA | + | NA |
| OTU17871 | - | NA | - | NA |
| OTU1092 | NA | NA | NA | NA |
| OTU1767 | + | NA | NA | NA |
| OTU15967 | NA | NA | NA | NA |
| OTU348 | + | NA | - | NA |
| OTU13396 | NA | - | NA | NA |
| OTU13960 | NA | + | NA | - |
| OTU4396 | NA | + | NA | NA |
| OTU14536 | NA | + | NA | NA |
| OTU1810 | NA | NA | NA | + |
| OTU14104 | + | NA | NA | NA |
| OTU3645 | NA | NA | NA | NA |
| OTU8998 | NA | NA | NA | NA |
| OTU17860 | NA | NA | NA | + |
| OTU17951 | + | NA | NA | NA |
| OTU18921 | - | NA | NA | NA |
| OTU1868 | NA | + | - | NA |
| OTU10148 | NA | NA | NA | NA |
| OTU11698 | NA | NA | NA | NA |
| OTU15804 | NA | NA | NA | + |
| OTU13777 | NA | NA | + | - |
| OTU4157 | NA | NA | NA | NA |
| OTU7875 | NA | NA | NA | NA |
| OTU1275 | NA | NA | NA | NA |
| OTU14575 | NA | NA | NA | NA |
| OTU17913 | NA | NA | NA | NA |
| OTU3122 | + | NA | NA | NA |
| OTU5598 | NA | NA | - | + |
| OTU1747 | NA | NA | NA | NA |
| OTU18446 | NA | NA | + | - |
| OTU17749 | NA | NA | + | NA |
| OTU11416 | NA | NA | - | NA |
| OTU6431 | NA | NA | - | + |
| OTU14821 | NA | + | NA | NA |
| OTU13062 | NA | + | - | NA |
| OTU14339 | NA | + | NA | NA |
| OTU14009 | NA | NA | + | - |
| OTU8722 | + | NA | - | NA |
| OTU14297 | NA | NA | + | NA |
| OTU7672 | NA | NA | - | NA |
| OTU13842 | NA | NA | NA | NA |
| OTU16447 | NA | NA | NA | NA |
| OTU6278 | NA | NA | NA | NA |
| OTU12382 | NA | NA | NA | NA |
| OTU10370 | NA | NA | NA | NA |
| OTU7290 | NA | NA | NA | NA |
| OTU17833 | NA | NA | + | NA |
| OTU10736 | NA | NA | NA | NA |
| OTU17251 | NA | NA | NA | + |
| OTU14999 | + | NA | - | NA |
| OTU5811 | + | NA | - | NA |
| OTU19151 | + | NA | - | NA |
| OTU931 | - | NA | + | NA |
| OTU8008 | NA | NA | NA | NA |
| OTU2416 | NA | NA | NA | NA |
| OTU14750 | + | NA | - | NA |
| OTU5517 | NA | NA | NA | - |
| OTU7955 | + | NA | - | NA |
| OTU16979 | - | + | - | NA |
| OTU18255 | NA | NA | NA | NA |
| OTU6024 | NA | NA | NA | + |
| OTU18215 | NA | NA | NA | NA |
| OTU4100 | NA | + | NA | - |
| OTU1906 | NA | + | NA | NA |
| OTU6668 | NA | + | NA | - |
| OTU12681 | NA | NA | NA | NA |
| OTU13571 | NA | - | NA | + |
| OTU7049 | + | NA | NA | - |
| OTU16347 | NA | - | + | NA |
| OTU13826 | NA | NA | NA | NA |
| OTU11175 | - | NA | + | NA |
| OTU17586 | NA | NA | NA | NA |
| OTU5808 | NA | NA | NA | NA |
| OTU6575 | NA | NA | NA | NA |
| OTU17538 | NA | NA | NA | NA |
| OTU14619 | NA | NA | NA | - |
| OTU13258 | NA | NA | NA | NA |
| OTU16853 | NA | NA | NA | + |
| OTU7764 | + | NA | NA | NA |
| OTU6326 | NA | NA | NA | NA |
| OTU7483 | NA | NA | NA | NA |
| OTU1636 | NA | NA | NA | NA |
| OTU13661 | + | NA | - | NA |
| OTU1968 | NA | NA | NA | NA |
| OTU17597 | NA | NA | NA | NA |
| OTU5052 | NA | + | NA | NA |
| OTU17567 | + | NA | - | NA |
| OTU4444 | NA | NA | NA | NA |
| OTU14294 | NA | NA | NA | NA |
| OTU14319 | + | NA | NA | NA |
| OTU6933 | NA | NA | NA | NA |
| OTU19108 | NA | + | NA | - |
| OTU18020 | NA | NA | - | NA |
| OTU13282 | NA | NA | NA | + |
| OTU18841 | NA | NA | NA | NA |
| OTU14365 | NA | NA | NA | NA |
| OTU14767 | - | NA | + | NA |
| OTU10496 | NA | - | NA | + |
| OTU2944 | NA | NA | NA | NA |
| OTU7657 | + | NA | - | NA |
| OTU14883 | + | NA | NA | NA |
| OTU13912 | - | NA | NA | NA |
| OTU7986 | NA | NA | NA | NA |
| OTU13145 | + | - | NA | NA |
| OTU13313 | NA | - | + | NA |
| OTU18336 | NA | - | NA | + |
| OTU14722 | NA | NA | - | + |
| OTU9557 | NA | NA | + | - |
| OTU18298 | + | NA | NA | NA |
| OTU852 | NA | NA | NA | NA |
| OTU14977 | - | NA | + | NA |
| OTU1382 | NA | NA | - | NA |
| OTU18132 | NA | NA | NA | NA |
| OTU5751 | NA | NA | - | + |
| OTU11212 | NA | NA | NA | NA |
| OTU7852 | NA | NA | NA | NA |
| OTU14409 | + | NA | NA | NA |
| OTU11355 | NA | NA | NA | NA |
| OTU12108 | NA | NA | NA | NA |
| OTU15061 | NA | + | NA | NA |
| OTU3397 | + | NA | NA | NA |
| OTU5395 | NA | NA | + | - |
| OTU10189 | NA | NA | NA | NA |
| OTU13733 | NA | NA | NA | NA |
| OTU13798 | NA | NA | NA | NA |
| OTU19136 | + | NA | NA | NA |
| OTU4636 | + | NA | NA | - |
| OTU13126 | NA | NA | NA | NA |
| OTU6769 | NA | NA | - | NA |
| OTU584 | + | NA | NA | NA |
| OTU17630 | NA | - | NA | NA |
| OTU4838 | NA | NA | NA | NA |
| OTU17741 | - | NA | + | NA |
| OTU6458 | NA | NA | NA | + |
| OTU11327 | + | NA | NA | NA |
| OTU18270 | NA | NA | NA | NA |
| OTU3215 | NA | NA | NA | NA |
| OTU17778 | NA | + | NA | NA |
| OTU7452 | NA | NA | NA | - |
| OTU2090 | NA | - | NA | NA |
| OTU17131 | NA | NA | NA | NA |
| OTU4438 | NA | NA | NA | - |
| OTU5958 | NA | NA | NA | NA |
| OTU14755 | NA | NA | NA | NA |
| OTU18314 | NA | NA | NA | NA |
| OTU9184 | NA | NA | - | NA |
| OTU14691 | - | NA | + | NA |
| OTU6457 | + | NA | - | NA |
| OTU13611 | NA | NA | NA | NA |
| OTU13276 | NA | NA | NA | NA |
| OTU2897 | NA | NA | NA | - |
| OTU6153 | NA | + | NA | NA |
| OTU13088 | NA | NA | NA | - |
| OTU4771 | NA | NA | NA | NA |
| OTU6830 | NA | + | NA | NA |
| OTU18146 | NA | NA | NA | + |
| OTU17683 | - | + | NA | NA |
| OTU6372 | + | NA | - | NA |
| OTU13290 | NA | NA | NA | NA |
| OTU18674 | NA | NA | NA | + |
| OTU7600 | + | NA | NA | NA |
| OTU9267 | - | + | NA | NA |
| OTU17045 | NA | NA | NA | + |
| OTU9935 | NA | NA | NA | NA |
| OTU12533 | NA | NA | + | NA |
| OTU17609 | NA | NA | NA | NA |
| OTU18015 | NA | NA | + | NA |
| OTU10433 | NA | NA | NA | NA |
| OTU2943 | - | NA | NA | NA |
| OTU13852 | + | NA | NA | NA |
| OTU14933 | NA | NA | NA | NA |
| OTU15418 | - | NA | - | + |
| OTU7812 | NA | NA | NA | NA |
| OTU7327 | + | NA | NA | NA |
| OTU14483 | - | NA | NA | NA |
| OTU13260 | NA | NA | NA | NA |
| OTU6780 | NA | - | NA | NA |
| OTU3923 | NA | - | NA | + |
| OTU6446 | - | + | NA | NA |
| OTU5856 | NA | NA | - | NA |
| OTU9741 | NA | - | NA | + |
| OTU8356 | NA | NA | - | + |
| OTU13757 | + | NA | NA | NA |
| OTU17019 | + | NA | NA | NA |
| OTU9618 | NA | + | NA | - |
| OTU17545 | NA | NA | NA | NA |
| OTU17803 | - | NA | NA | NA |
| OTU3753 | NA | NA | NA | NA |
| OTU3422 | + | NA | NA | NA |
| OTU6900 | - | NA | + | NA |
| OTU18485 | NA | NA | NA | + |
| OTU15610 | NA | NA | NA | NA |
| OTU15000 | NA | - | NA | + |
| OTU4592 | - | + | NA | NA |
| OTU3656 | NA | NA | NA | NA |
| OTU1979 | - | NA | + | NA |
| OTU13211 | NA | NA | NA | NA |
| OTU14764 | - | NA | NA | NA |
| OTU4573 | NA | NA | NA | NA |
| OTU17617 | NA | NA | NA | + |
| OTU1675 | NA | NA | NA | NA |
| OTU14785 | + | - | NA | NA |
| OTU17855 | NA | NA | NA | NA |
| OTU14579 | NA | NA | NA | NA |
| OTU16187 | + | NA | NA | NA |
| OTU492 | + | NA | NA | NA |
| OTU17628 | NA | - | NA | + |
| OTU17024 | + | NA | NA | NA |
| OTU15357 | NA | NA | - | + |
| OTU13894 | NA | NA | NA | NA |
| OTU19075 | + | NA | NA | NA |
| OTU13508 | + | NA | NA | NA |
| OTU18319 | NA | NA | NA | + |
| OTU1454 | NA | NA | NA | - |
| OTU8926 | NA | NA | NA | + |
| OTU7714 | + | NA | - | NA |
| OTU15035 | NA | NA | NA | NA |
| OTU13539 | + | NA | NA | NA |
| OTU13375 | + | NA | NA | NA |
| OTU1720 | - | + | NA | NA |
| OTU1308 | + | NA | NA | NA |
| OTU18459 | NA | NA | + | NA |
| OTU15982 | NA | NA | NA | + |
| OTU17637 | NA | NA | NA | NA |
| OTU2157 | NA | + | NA | NA |
| OTU13731 | NA | + | NA | - |
| OTU3400 | + | NA | - | NA |
| OTU13028 | + | - | + | NA |
| OTU17626 | NA | NA | NA | NA |
| OTU14553 | - | NA | NA | NA |
| OTU14648 | + | NA | NA | NA |
| OTU13966 | NA | NA | NA | - |
| OTU18221 | NA | - | NA | + |
| OTU18582 | + | - | NA | NA |
| OTU13771 | NA | NA | NA | NA |
| OTU6472 | NA | NA | NA | NA |
| OTU7691 | + | NA | - | NA |
| OTU11939 | NA | - | NA | + |
| OTU7557 | + | NA | NA | NA |
| OTU7471 | + | NA | NA | NA |
| OTU2124 | NA | NA | NA | + |
| OTU16211 | - | NA | NA | NA |
| OTU5985 | + | NA | NA | NA |
| OTU13730 | NA | NA | NA | NA |
| OTU7632 | + | NA | - | NA |
| OTU7658 | + | NA | - | NA |
| OTU10095 | NA | NA | NA | NA |
| OTU14626 | NA | NA | + | NA |
| OTU14938 | NA | NA | NA | NA |
| OTU17835 | NA | NA | NA | NA |
| OTU7218 | NA | NA | + | NA |
| OTU1796 | NA | NA | + | - |
| OTU14354 | NA | - | NA | NA |
| OTU11394 | - | + | NA | NA |
| OTU14318 | NA | NA | + | NA |
| OTU15232 | NA | NA | NA | NA |
| OTU15005 | NA | NA | NA | NA |
| OTU18009 | NA | NA | NA | NA |
| OTU13140 | NA | NA | NA | NA |
| OTU13769 | NA | - | NA | NA |
| OTU291 | NA | + | NA | NA |
| OTU18389 | + | NA | - | NA |
| OTU3835 | NA | NA | NA | NA |
| OTU14965 | NA | NA | + | - |
| OTU18090 | NA | NA | NA | + |
| OTU15867 | NA | - | NA | + |
| OTU9275 | NA | + | NA | - |
| OTU15056 | NA | NA | NA | NA |
| OTU2019 | NA | NA | NA | NA |
| OTU17989 | NA | NA | NA | NA |
| OTU14352 | NA | NA | NA | - |
| OTU11930 | NA | NA | NA | NA |
| OTU13703 | NA | NA | + | NA |
| OTU2137 | NA | NA | + | - |
| OTU16834 | NA | NA | NA | NA |
| OTU17144 | + | NA | NA | NA |
| OTU14548 | NA | NA | NA | NA |
| OTU17754 | NA | NA | NA | + |
| OTU17544 | NA | NA | NA | NA |
| OTU10273 | NA | NA | NA | + |
| OTU14532 | NA | NA | NA | NA |
| OTU3733 | NA | NA | NA | NA |
| OTU14317 | - | NA | + | NA |
| OTU8105 | + | NA | NA | NA |
| OTU9051 | + | NA | NA | NA |
| OTU17891 | NA | - | NA | + |
| OTU8903 | NA | NA | NA | - |
| OTU2946 | NA | NA | NA | NA |
| OTU1176 | NA | NA | NA | - |
| OTU18318 | NA | + | NA | NA |
| OTU6700 | NA | NA | - | NA |
| OTU6227 | NA | NA | + | - |
| OTU18860 | NA | NA | NA | NA |
| OTU12433 | NA | + | NA | NA |
| OTU15048 | NA | NA | NA | NA |
| OTU10854 | NA | NA | + | NA |
| OTU5061 | NA | NA | NA | NA |
| OTU17329 | + | - | NA | NA |
| OTU2091 | NA | NA | NA | NA |
| OTU1335 | NA | NA | NA | - |
| OTU1303 | + | NA | - | NA |
| OTU17789 | - | NA | NA | NA |
| OTU6456 | + | NA | - | NA |
| OTU119 | + | NA | - | NA |
| OTU7426 | NA | + | - | NA |
| OTU18458 | NA | NA | - | + |
| OTU15108 | NA | NA | NA | + |
| OTU17083 | NA | - | NA | + |
| OTU12615 | NA | NA | NA | NA |
| OTU9014 | + | NA | NA | NA |
| OTU15462 | NA | NA | NA | NA |
| OTU19110 | NA | NA | + | - |
| OTU13924 | + | - | NA | NA |
| OTU7800 | NA | NA | NA | NA |
| OTU2457 | + | NA | NA | NA |
| OTU8990 | NA | NA | + | - |
| OTU13431 | + | NA | NA | NA |
| OTU5069 | NA | NA | NA | NA |
| OTU9604 | NA | NA | NA | NA |
| OTU1236 | + | NA | NA | NA |
| OTU7446 | + | NA | - | NA |
| OTU16688 | NA | NA | NA | + |
| OTU10806 | - | NA | NA | NA |
| OTU14056 | NA | NA | NA | NA |
| OTU2962 | NA | NA | NA | NA |
| OTU9584 | - | NA | + | NA |
| OTU18012 | NA | - | NA | + |
| OTU1634 | NA | NA | + | NA |
| OTU12599 | NA | + | - | NA |
| OTU18289 | NA | NA | NA | NA |
| OTU10479 | NA | NA | NA | NA |
| OTU13280 | + | NA | - | NA |
| OTU12301 | NA | NA | + | - |
| OTU9812 | NA | NA | NA | NA |
| OTU535 | + | NA | NA | NA |
| OTU17884 | NA | NA | NA | NA |
| OTU13591 | NA | NA | NA | NA |
| OTU15971 | - | NA | + | NA |
| OTU10868 | + | - | NA | NA |
| OTU4230 | NA | NA | + | NA |
| OTU1458 | NA | + | NA | - |
| OTU4518 | NA | NA | NA | - |
| OTU12990 | + | NA | NA | - |
| OTU14627 | NA | NA | NA | NA |
| OTU911 | + | NA | - | NA |
| OTU10637 | + | NA | NA | NA |
| OTU14556 | NA | NA | - | NA |
| OTU4644 | NA | NA | NA | NA |
| OTU7826 | NA | - | NA | NA |
| OTU3424 | + | NA | NA | NA |
| OTU5364 | NA | NA | NA | + |
| OTU18969 | NA | NA | NA | - |
| OTU16059 | NA | NA | - | NA |
| OTU8363 | NA | NA | NA | NA |
| OTU5234 | - | + | NA | NA |
| OTU13629 | + | NA | NA | NA |
| OTU14644 | NA | NA | + | - |
| OTU13615 | + | NA | NA | NA |
| OTU3833 | - | NA | NA | NA |
| OTU18462 | NA | + | NA | - |
| OTU17815 | - | NA | NA | NA |
| OTU8730 | - | NA | NA | NA |
| OTU9103 | + | NA | NA | NA |
| OTU13854 | NA | NA | NA | NA |
| OTU18454 | NA | NA | + | - |
| OTU18362 | NA | NA | - | NA |
| OTU13801 | + | NA | - | NA |
| OTU16075 | NA | - | NA | NA |
| OTU12983 | NA | NA | NA | NA |
| OTU5766 | + | NA | NA | NA |
| OTU6080 | NA | NA | NA | NA |
| OTU2152 | - | NA | NA | NA |
| OTU5712 | NA | NA | NA | NA |
| OTU4884 | - | NA | NA | NA |
| OTU18676 | NA | NA | NA | + |
| OTU1864 | NA | + | NA | NA |
| OTU13644 | + | NA | NA | NA |
| OTU14643 | + | NA | NA | NA |
| OTU18483 | NA | NA | NA | + |
| OTU15076 | NA | NA | NA | + |
| OTU102 | NA | NA | NA | NA |
| OTU14074 | NA | NA | + | - |
| OTU5377 | NA | NA | + | NA |
| OTU5519 | NA | NA | NA | NA |
| OTU14899 | NA | NA | NA | - |
| OTU4520 | - | NA | + | NA |
| OTU7709 | + | NA | NA | NA |
| OTU1274 | + | NA | - | NA |
| OTU14447 | NA | NA | NA | - |
| OTU7779 | NA | - | NA | NA |
| OTU11209 | NA | NA | NA | NA |
| OTU1299 | NA | NA | NA | NA |
| OTU16252 | - | NA | NA | NA |
| OTU2462 | + | - | NA | NA |
| OTU9165 | + | NA | NA | NA |
| OTU14692 | + | NA | NA | NA |
| OTU8116 | + | NA | NA | NA |
| OTU18027 | NA | + | NA | NA |
| OTU8377 | + | NA | NA | NA |
| OTU4561 | + | NA | - | NA |
| OTU14038 | - | NA | + | NA |
| OTU17310 | NA | NA | NA | NA |
| OTU13148 | + | NA | NA | NA |
| OTU364 | + | NA | - | NA |
| OTU12999 | NA | + | NA | NA |
| OTU18058 | - | NA | NA | NA |
| OTU6397 | NA | NA | NA | NA |
| OTU12404 | NA | NA | - | NA |
| OTU4130 | NA | NA | NA | NA |
| OTU794 | NA | NA | NA | NA |
| OTU1456 | + | - | NA | NA |
| OTU18364 | NA | NA | NA | NA |
| OTU3799 | NA | NA | NA | NA |
| OTU7705 | - | + | NA | NA |
| OTU14108 | NA | NA | NA | NA |
| OTU1298 | + | NA | NA | NA |
| OTU14388 | NA | NA | + | NA |
| OTU9765 | NA | NA | NA | NA |
| OTU18084 | NA | NA | NA | NA |
| OTU1104 | + | - | NA | NA |
| OTU17662 | + | NA | NA | NA |
| OTU1295 | + | NA | - | NA |
| OTU15047 | NA | NA | NA | NA |
| OTU12453 | + | NA | NA | - |
| OTU14046 | NA | + | NA | - |
| OTU11278 | + | NA | NA | NA |
| OTU520 | NA | NA | NA | NA |
| OTU14513 | NA | + | NA | - |
| OTU18087 | NA | NA | NA | NA |
| OTU645 | + | NA | NA | NA |
| OTU14935 | - | NA | NA | NA |
| OTU5682 | + | NA | NA | NA |
| OTU18284 | NA | NA | NA | + |
| OTU10466 | NA | - | NA | NA |
| OTU18757 | NA | NA | NA | - |
| OTU6388 | NA | NA | NA | NA |
| OTU17611 | NA | NA | - | + |
| OTU14589 | NA | NA | NA | NA |
| OTU6466 | + | NA | NA | NA |
| OTU13939 | + | NA | NA | NA |
| OTU1302 | + | NA | NA | NA |
| OTU3726 | NA | NA | NA | NA |
| OTU6444 | + | NA | - | NA |
| OTU16098 | NA | NA | - | NA |
| OTU6371 | NA | NA | NA | NA |
| OTU17675 | NA | NA | + | NA |
| OTU13976 | + | NA | NA | NA |
| OTU808 | + | NA | NA | NA |
| OTU7835 | NA | NA | NA | NA |
| OTU18037 | NA | NA | NA | NA |
| OTU4830 | - | NA | + | NA |
| OTU14769 | NA | NA | - | NA |
| OTU13240 | NA | NA | NA | NA |
| OTU10295 | NA | NA | NA | - |
| OTU4113 | NA | NA | NA | NA |
| OTU13425 | + | NA | NA | NA |
| OTU3517 | + | NA | NA | NA |
| OTU11057 | NA | + | NA | NA |
| OTU1366 | + | NA | - | NA |
| OTU11123 | NA | NA | - | NA |
| OTU3710 | NA | + | NA | NA |
| OTU14910 | NA | NA | NA | NA |
| OTU14316 | - | NA | NA | NA |
| OTU5384 | NA | NA | NA | NA |
| OTU14784 | NA | NA | NA | NA |
| OTU12362 | + | NA | - | NA |
| OTU8941 | NA | NA | - | + |
| OTU7765 | + | NA | NA | NA |
| OTU14908 | NA | NA | NA | NA |
| OTU194 | + | NA | NA | - |
| OTU2195 | + | NA | - | NA |
| OTU6339 | - | + | NA | NA |
| OTU2818 | + | NA | NA | NA |
| OTU10812 | NA | NA | NA | + |
| OTU2323 | NA | NA | NA | + |
| OTU18165 | NA | NA | NA | NA |
| OTU2928 | NA | - | NA | NA |
| OTU3932 | NA | NA | NA | + |
| OTU7416 | + | - | NA | NA |
| OTU13575 | + | NA | NA | NA |
| OTU14670 | NA | NA | NA | NA |
| OTU5281 | + | NA | NA | NA |
| OTU7493 | NA | NA | NA | + |
| OTU1680 | NA | NA | NA | NA |
| OTU18315 | NA | NA | NA | NA |
| OTU5910 | NA | + | NA | - |
| OTU1328 | + | NA | NA | NA |
| OTU12979 | + | NA | NA | NA |
| OTU8438 | + | NA | NA | NA |
| OTU7480 | - | + | NA | NA |
| OTU7921 | NA | NA | NA | - |
| OTU3874 | - | NA | + | NA |
| OTU14972 | NA | NA | NA | NA |
| OTU13268 | + | NA | NA | NA |
| OTU17343 | NA | NA | NA | NA |
| OTU5973 | NA | NA | - | NA |
| OTU12401 | NA | NA | NA | NA |
| OTU2065 | NA | NA | NA | NA |
| OTU13347 | NA | NA | NA | NA |
| OTU6520 | NA | NA | NA | + |
| OTU16879 | + | NA | NA | NA |
| OTU18395 | NA | NA | NA | NA |
| OTU16761 | NA | NA | NA | NA |
| OTU14472 | + | NA | - | NA |
| OTU17096 | NA | NA | + | NA |
| OTU8319 | + | NA | NA | NA |
| OTU16758 | + | NA | NA | NA |
| OTU6188 | NA | NA | NA | NA |
| OTU18199 | - | + | NA | NA |
| OTU10788 | NA | NA | + | NA |
| OTU2178 | NA | NA | NA | NA |
| OTU17836 | NA | NA | NA | + |
| OTU6649 | NA | NA | NA | + |
| OTU11563 | NA | NA | + | NA |
| OTU353 | + | NA | - | NA |
| OTU5181 | NA | NA | NA | NA |
| OTU16515 | NA | NA | NA | NA |
| OTU9313 | - | NA | + | NA |
| OTU3513 | + | NA | NA | NA |
| OTU566 | NA | NA | NA | NA |
| OTU572 | NA | NA | + | - |
| OTU13374 | NA | NA | NA | NA |
| OTU13040 | + | NA | - | NA |
| OTU416 | NA | NA | NA | - |
| OTU1252 | + | NA | - | NA |
| OTU18211 | NA | NA | NA | + |
| OTU5041 | NA | NA | NA | NA |
| OTU17515 | NA | NA | NA | NA |
| OTU4943 | NA | - | NA | NA |
| OTU4009 | NA | NA | + | NA |
| OTU7488 | NA | NA | NA | NA |
| OTU3610 | + | NA | - | + |
| OTU7811 | NA | NA | NA | - |
| OTU17378 | NA | + | NA | NA |
| OTU5813 | NA | + | - | NA |
| OTU15822 | NA | - | NA | NA |
| OTU10883 | NA | - | NA | + |
| OTU11847 | - | NA | NA | NA |
| OTU14380 | NA | NA | NA | NA |
| OTU9317 | NA | NA | NA | NA |
| OTU7891 | + | NA | - | NA |
| OTU18867 | NA | NA | + | NA |
| OTU6399 | NA | NA | NA | NA |
| OTU18368 | NA | NA | NA | NA |
| OTU2682 | + | - | NA | NA |
| OTU7353 | + | NA | - | NA |
| OTU14765 | NA | NA | - | + |
| OTU6433 | + | - | NA | NA |
| OTU7554 | NA | NA | NA | + |
| OTU2391 | + | NA | NA | + |
| OTU18022 | NA | NA | - | NA |
| OTU617 | NA | NA | NA | NA |
| OTU15238 | NA | NA | NA | NA |
| OTU6013 | + | NA | NA | NA |
| OTU16118 | NA | NA | NA | NA |
| OTU7766 | NA | NA | NA | NA |
| OTU1582 | NA | NA | NA | NA |
| OTU17210 | - | + | NA | NA |
| OTU6416 | NA | NA | NA | + |
| OTU13605 | NA | NA | NA | + |
| OTU582 | NA | NA | NA | + |
| OTU10948 | NA | NA | NA | + |
| OTU13284 | + | NA | - | NA |
| OTU7674 | + | NA | - | NA |
| OTU9621 | NA | NA | NA | NA |
| OTU18861 | NA | NA | NA | NA |
| OTU4877 | NA | NA | NA | NA |
| OTU4848 | NA | NA | NA | NA |
| OTU11478 | NA | NA | NA | NA |
| OTU12386 | NA | NA | NA | NA |
| OTU17896 | NA | NA | NA | NA |
| OTU10009 | NA | NA | NA | - |
| OTU18307 | NA | NA | NA | + |
| OTU7695 | + | NA | NA | NA |
| OTU13494 | + | NA | - | NA |
| OTU14629 | NA | NA | NA | NA |
| OTU15717 | NA | + | NA | NA |
| OTU16573 | NA | NA | NA | + |
| OTU18117 | NA | NA | NA | + |
| OTU1331 | + | NA | NA | NA |
| OTU6719 | NA | NA | - | NA |
| OTU2109 | NA | NA | NA | NA |
| OTU835 | NA | NA | NA | NA |
| OTU1294 | + | NA | NA | NA |
| OTU9489 | NA | NA | NA | NA |
| OTU8281 | NA | NA | NA | NA |
| OTU16666 | NA | NA | - | + |
| OTU3117 | NA | - | + | NA |
| OTU13180 | NA | NA | + | NA |
| OTU7677 | NA | NA | NA | NA |
| OTU19101 | + | NA | NA | NA |
| OTU18428 | + | - | NA | NA |
| OTU15864 | NA | NA | NA | + |
| OTU5374 | NA | NA | + | - |
| OTU527 | NA | + | NA | - |
| OTU16981 | + | NA | NA | NA |
| OTU17633 | - | + | NA | NA |
| OTU12637 | NA | - | NA | NA |
| OTU15789 | NA | NA | - | + |
| OTU5866 | - | + | NA | NA |
| OTU9911 | NA | NA | NA | NA |
| OTU16978 | NA | + | - | + |
| OTU2026 | NA | NA | NA | NA |
| OTU13138 | NA | NA | + | NA |
| OTU17893 | NA | NA | NA | + |
| OTU17035 | NA | NA | - | + |
| OTU9264 | NA | NA | NA | + |
| OTU8727 | + | NA | - | NA |
| OTU13273 | NA | NA | NA | NA |
| OTU11283 | NA | NA | NA | NA |
| OTU2942 | NA | NA | - | + |
| OTU15266 | NA | - | NA | + |
| OTU15090 | NA | NA | NA | - |
| OTU4545 | NA | NA | NA | + |
| OTU13129 | NA | NA | NA | NA |
| OTU4543 | NA | NA | NA | NA |
| OTU7698 | + | NA | NA | NA |
| OTU12089 | NA | NA | + | - |
| OTU9387 | NA | + | - | NA |
| OTU1305 | + | NA | - | NA |
| OTU17988 | NA | NA | - | + |
| OTU17961 | + | NA | NA | NA |
| OTU6883 | + | NA | NA | NA |
| OTU16946 | NA | NA | - | NA |
| OTU14685 | - | + | NA | NA |
| OTU7609 | + | NA | NA | NA |
| OTU13951 | NA | NA | NA | NA |
| OTU13722 | NA | NA | NA | NA |
| OTU14425 | NA | NA | NA | - |
| OTU17296 | NA | NA | NA | + |
| OTU17138 | NA | NA | NA | NA |
| OTU17172 | + | NA | NA | NA |
| OTU7467 | + | NA | - | NA |
| OTU5650 | - | NA | NA | NA |
| OTU16351 | NA | NA | - | + |
| OTU14668 | NA | NA | NA | NA |
| OTU13047 | NA | NA | - | NA |
| OTU13551 | + | NA | NA | NA |
| OTU17574 | NA | NA | NA | NA |
| OTU5083 | NA | NA | NA | - |
| OTU12544 | - | + | NA | NA |
| OTU2 | NA | NA | - | + |
| OTU1560 | NA | NA | NA | NA |
| OTU13545 | NA | + | NA | NA |
| OTU1420 | + | NA | NA | NA |
| OTU12662 | + | NA | NA | NA |
| OTU14459 | NA | + | - | NA |
| OTU5185 | NA | NA | NA | - |
| OTU5066 | NA | NA | NA | NA |
| OTU17516 | NA | NA | - | + |
| OTU12880 | NA | NA | NA | NA |
| OTU11402 | NA | NA | NA | NA |
| OTU18254 | NA | NA | NA | NA |
| OTU13604 | NA | NA | NA | NA |
| OTU18191 | NA | NA | NA | NA |
| OTU6635 | NA | NA | - | NA |
| OTU9188 | + | NA | NA | NA |
| OTU10378 | NA | NA | NA | NA |
| OTU15368 | NA | NA | NA | + |
| OTU9645 | - | NA | NA | NA |
| OTU13342 | + | NA | NA | - |
| OTU8933 | + | NA | - | NA |
| OTU13913 | NA | NA | + | NA |
| OTU18003 | NA | + | NA | NA |
| OTU8423 | NA | NA | NA | NA |
| OTU13763 | NA | NA | + | NA |
| OTU16660 | - | NA | NA | NA |
| OTU17240 | NA | NA | NA | NA |
| OTU9619 | NA | NA | - | NA |
| OTU13463 | NA | NA | NA | NA |
| OTU13621 | NA | + | NA | NA |
| OTU13675 | NA | NA | NA | NA |
| OTU829 | + | - | NA | NA |
| OTU3199 | NA | NA | NA | NA |
| OTU5778 | + | NA | NA | NA |
| OTU13544 | + | NA | NA | NA |
| OTU11589 | NA | NA | NA | NA |
| OTU16745 | NA | NA | NA | + |
| OTU11222 | NA | NA | NA | NA |
| OTU385 | + | NA | - | NA |
| OTU18797 | + | NA | NA | NA |
| OTU1394 | NA | NA | NA | NA |
| OTU14916 | + | NA | NA | - |
| OTU14642 | NA | NA | NA | NA |
| OTU12700 | NA | + | NA | NA |
| OTU17221 | NA | NA | NA | NA |
| OTU17089 | NA | NA | - | NA |
| OTU8050 | NA | NA | - | NA |
| OTU14565 | NA | + | NA | NA |
| OTU4392 | NA | NA | NA | NA |
| OTU2695 | NA | NA | NA | NA |
| OTU13780 | NA | NA | + | - |
| OTU8213 | NA | NA | NA | NA |
| OTU7001 | NA | NA | NA | NA |
| OTU10456 | + | NA | NA | NA |
| OTU17572 | NA | + | NA | NA |
| OTU18028 | NA | - | NA | + |
| OTU18177 | NA | NA | + | NA |
| OTU13728 | NA | NA | NA | NA |
| OTU19064 | NA | NA | - | NA |
| OTU17653 | NA | NA | NA | NA |
| OTU5180 | NA | NA | NA | NA |
| OTU18080 | NA | NA | + | - |
| OTU2100 | NA | + | - | NA |
| OTU18467 | NA | NA | NA | NA |
| OTU10683 | NA | - | NA | + |
| OTU7352 | + | NA | - | NA |
| OTU1239 | NA | NA | NA | - |
| OTU107 | + | NA | NA | NA |
| OTU13081 | NA | NA | NA | NA |
| OTU14455 | NA | NA | + | NA |
| OTU17883 | NA | NA | NA | + |
| OTU13010 | NA | - | NA | + |
| OTU13702 | + | - | NA | NA |
| OTU13866 | - | NA | + | NA |
| OTU2075 | + | - | NA | - |
| OTU7895 | NA | NA | + | NA |
| OTU14660 | NA | NA | NA | NA |
| OTU11454 | + | NA | NA | - |
| OTU7926 | + | NA | - | NA |
| OTU9439 | + | NA | - | NA |
| OTU7582 | + | NA | NA | NA |
| OTU13847 | NA | - | + | NA |
| OTU13428 | NA | NA | NA | NA |
| OTU626 | NA | NA | NA | NA |
| OTU17605 | + | - | + | - |
| OTU14727 | NA | NA | + | - |
| OTU10866 | NA | NA | + | - |
| OTU17885 | NA | NA | NA | - |
| OTU13073 | + | NA | - | NA |
| OTU14562 | - | NA | NA | NA |
| OTU19174 | + | NA | NA | NA |
| OTU13004 | - | NA | + | NA |
| OTU10616 | NA | NA | NA | NA |
| OTU16412 | NA | NA | - | + |
| OTU16381 | NA | NA | NA | NA |
| OTU18365 | NA | - | NA | NA |
| OTU1729 | NA | NA | NA | NA |
| OTU1836 | NA | NA | - | + |
| OTU12720 | NA | NA | NA | NA |
| OTU14789 | NA | NA | NA | NA |
| OTU13982 | NA | NA | NA | NA |
| OTU4828 | NA | NA | NA | NA |
| OTU625 | + | NA | NA | NA |
| OTU3464 | + | NA | - | NA |
| OTU14981 | NA | NA | NA | NA |
| OTU11223 | NA | NA | + | - |
| OTU4598 | NA | NA | NA | NA |
| OTU18384 | NA | NA | NA | NA |
| OTU13128 | + | NA | NA | NA |
| OTU4993 | + | NA | NA | - |
| OTU14293 | NA | NA | NA | NA |
| OTU10029 | - | NA | NA | NA |
| OTU18352 | NA | NA | NA | NA |
| OTU6298 | NA | + | - | NA |
| OTU13843 | - | NA | NA | NA |
| OTU18033 | NA | NA | + | - |
| OTU5709 | NA | NA | + | NA |
| OTU5914 | NA | NA | - | + |
| OTU2089 | NA | NA | + | - |
| OTU6368 | NA | + | - | NA |
| OTU1162 | NA | NA | NA | NA |
| OTU7873 | NA | NA | NA | NA |
| OTU1811 | NA | NA | NA | NA |
| OTU1260 | + | - | NA | NA |
| OTU3732 | NA | NA | NA | - |
| OTU2162 | NA | NA | + | - |
| OTU2829 | + | NA | - | NA |
| OTU13949 | NA | NA | NA | + |
| OTU14762 | NA | - | NA | NA |
| OTU17806 | NA | NA | NA | NA |
| OTU12343 | + | NA | NA | NA |
| OTU17706 | NA | NA | - | NA |
| OTU6455 | NA | NA | NA | NA |
| OTU209 | NA | NA | - | + |
| OTU13803 | + | NA | NA | NA |
| OTU7872 | NA | NA | NA | NA |
| OTU5903 | NA | NA | NA | NA |
| OTU13509 | + | NA | NA | NA |
| OTU13903 | NA | NA | NA | NA |
| OTU1819 | NA | NA | NA | NA |
| OTU13308 | NA | - | NA | NA |
| OTU18405 | NA | NA | NA | NA |
| OTU5114 | NA | NA | NA | NA |
| OTU1315 | + | NA | NA | NA |
| OTU7845 | + | NA | NA | NA |
| OTU11679 | NA | + | - | NA |
| OTU13802 | NA | NA | + | NA |
| OTU3284 | + | NA | NA | NA |
| OTU18431 | NA | NA | NA | NA |
| OTU14514 | NA | NA | NA | NA |
| OTU14473 | + | NA | - | NA |
| OTU17711 | NA | - | NA | + |
| OTU3759 | NA | NA | - | NA |
| OTU6325 | + | NA | NA | NA |
| OTU13534 | NA | NA | NA | NA |
| OTU5846 | + | NA | - | NA |
| OTU1756 | NA | NA | + | NA |
| OTU7743 | NA | NA | NA | NA |
| OTU13845 | NA | NA | NA | NA |
| OTU3115 | + | NA | - | NA |
| OTU5076 | NA | NA | NA | NA |
| OTU9738 | NA | NA | NA | + |
| OTU624 | + | NA | NA | NA |
| OTU14705 | NA | NA | NA | - |
| OTU17699 | NA | NA | NA | NA |
| OTU2738 | NA | NA | NA | NA |
| OTU17681 | NA | NA | - | NA |
| OTU2160 | NA | NA | + | - |
| OTU13891 | NA | NA | NA | NA |
| OTU4068 | - | + | NA | NA |
| OTU555 | NA | - | NA | + |
| OTU14504 | + | - | NA | NA |
| OTU1332 | NA | NA | NA | NA |
| OTU9305 | NA | NA | NA | NA |
| OTU2054 | NA | - | NA | NA |
| OTU18147 | NA | NA | NA | NA |
| OTU14852 | NA | NA | + | - |
| OTU16987 | + | NA | - | + |
| OTU12875 | + | NA | NA | NA |
| OTU15936 | NA | NA | NA | NA |
| OTU17188 | NA | NA | NA | NA |
| OTU2172 | NA | NA | NA | NA |
| OTU13181 | NA | NA | NA | - |
| OTU17151 | NA | NA | NA | NA |
| OTU8130 | NA | NA | - | NA |
| OTU7288 | NA | NA | NA | NA |
| OTU17784 | + | NA | NA | NA |
| OTU3311 | + | NA | NA | NA |
| OTU6264 | NA | + | NA | NA |
| OTU14350 | + | NA | NA | NA |
| OTU13628 | + | NA | NA | NA |
| OTU14898 | NA | NA | + | - |
| OTU18309 | NA | NA | - | NA |
| OTU7628 | NA | NA | NA | NA |
| OTU5794 | - | NA | NA | NA |
| OTU5738 | + | NA | - | NA |
| OTU13190 | + | NA | - | NA |
| OTU14397 | NA | NA | + | NA |
| OTU14329 | NA | NA | NA | NA |
| OTU2533 | NA | NA | NA | NA |
| OTU5857 | + | NA | - | NA |
| OTU2176 | NA | NA | NA | NA |
| OTU14669 | + | NA | NA | - |
| OTU17425 | NA | NA | + | - |
| OTU13981 | NA | NA | NA | NA |
| OTU9611 | - | NA | NA | NA |
| OTU3940 | NA | NA | NA | NA |
| OTU12803 | NA | NA | NA | NA |
| OTU5768 | NA | NA | NA | NA |
| OTU14591 | - | + | NA | NA |
| OTU14030 | NA | NA | + | NA |
| OTU13859 | + | - | NA | NA |
| OTU18126 | NA | - | NA | NA |
| OTU17652 | NA | NA | NA | + |
| OTU14546 | NA | NA | NA | NA |
| OTU971 | NA | - | NA | + |
| OTU3975 | + | NA | NA | NA |
| OTU18857 | - | NA | NA | NA |
| OTU3051 | NA | NA | NA | NA |
| OTU8147 | NA | NA | NA | NA |
| OTU4355 | NA | NA | + | NA |
| OTU14596 | NA | NA | NA | NA |
| OTU3470 | + | NA | NA | NA |
| OTU2072 | NA | NA | + | - |
| OTU14084 | + | NA | NA | NA |
| OTU18700 | NA | + | - | NA |
| OTU5441 | NA | NA | + | NA |
| OTU265 | NA | NA | NA | NA |
| OTU13177 | + | NA | NA | NA |
| OTU4479 | - | NA | NA | NA |
| OTU343 | + | NA | - | NA |
| OTU7163 | NA | NA | NA | NA |
| OTU1174 | + | - | NA | NA |
| OTU14518 | NA | - | + | NA |
| OTU9287 | + | NA | NA | - |
| OTU16079 | NA | NA | NA | NA |
| OTU13153 | NA | NA | NA | NA |
| OTU2129 | NA | + | NA | NA |
| OTU18394 | + | - | NA | + |
| OTU7006 | NA | NA | NA | NA |
| OTU12647 | NA | NA | NA | NA |
| OTU2234 | + | NA | NA | NA |
| OTU13340 | NA | + | NA | - |
| OTU8808 | + | NA | NA | NA |
| OTU11660 | NA | NA | + | NA |
| OTU13209 | NA | - | NA | NA |
| OTU14404 | NA | NA | NA | NA |
| OTU17673 | NA | NA | NA | NA |
| OTU7938 | + | NA | - | NA |
| OTU13206 | + | NA | NA | NA |
| OTU12230 | NA | NA | NA | - |
| OTU2852 | NA | NA | NA | NA |
| OTU8186 | + | - | NA | NA |
| OTU11378 | NA | NA | NA | NA |
| OTU14412 | NA | NA | NA | - |
| OTU575 | + | NA | NA | NA |
| OTU7444 | + | NA | - | NA |
| OTU5566 | - | NA | NA | NA |
| OTU15079 | + | NA | NA | NA |
| OTU14696 | + | NA | NA | NA |
| OTU14045 | NA | NA | NA | NA |
| OTU17928 | NA | NA | NA | + |
| OTU10800 | NA | NA | NA | + |
| OTU12669 | NA | NA | NA | NA |
| OTU19149 | + | - | NA | NA |
| OTU6689 | + | NA | NA | NA |
| OTU17914 | NA | NA | - | + |
| OTU18317 | NA | NA | NA | NA |
| OTU5771 | NA | NA | NA | NA |
| OTU14560 | NA | NA | + | NA |
| OTU7857 | NA | + | - | NA |
| OTU6731 | NA | NA | NA | NA |
| OTU14341 | NA | NA | NA | NA |
| OTU1285 | NA | NA | NA | - |
| OTU1189 | + | NA | - | NA |
| OTU14313 | NA | NA | NA | NA |
| OTU7867 | NA | NA | NA | NA |
| OTU1905 | NA | NA | + | NA |
| OTU1039 | NA | NA | NA | NA |
| OTU10580 | NA | NA | NA | NA |
| OTU7716 | + | NA | - | NA |
| OTU16904 | + | NA | - | NA |
| OTU15407 | NA | NA | NA | NA |
| OTU15823 | NA | + | NA | - |
| OTU7568 | NA | NA | - | NA |
| OTU7638 | NA | NA | - | + |
| OTU3721 | + | NA | NA | NA |
| OTU18111 | - | NA | + | NA |
| OTU2491 | + | NA | NA | NA |
| OTU7374 | + | NA | NA | NA |
| OTU1703 | NA | NA | NA | + |
| OTU5179 | NA | NA | NA | NA |
| OTU12879 | NA | NA | NA | NA |
| OTU14907 | NA | NA | NA | NA |
| OTU17766 | NA | NA | NA | + |
| OTU5609 | NA | NA | NA | NA |
| OTU12589 | NA | NA | NA | NA |
| OTU1625 | NA | NA | NA | NA |
| OTU14426 | + | NA | NA | NA |
| OTU17720 | NA | NA | NA | NA |
| OTU18267 | NA | NA | NA | + |
| OTU12026 | NA | NA | + | - |
| OTU17724 | NA | NA | NA | + |
| OTU1164 | NA | NA | NA | NA |
| OTU4528 | NA | NA | NA | NA |
| OTU9442 | NA | NA | NA | NA |
| OTU17934 | NA | NA | NA | NA |
| OTU7604 | + | NA | - | NA |
| OTU7621 | - | + | NA | NA |
| OTU5881 | NA | NA | NA | NA |
| OTU9058 | NA | NA | NA | NA |
| OTU9925 | - | NA | NA | + |
| OTU16995 | NA | - | NA | NA |
| OTU6591 | NA | NA | NA | - |
| OTU8721 | NA | NA | NA | NA |
| OTU7108 | NA | + | NA | NA |
| OTU17919 | - | NA | NA | + |
| OTU2694 | + | NA | - | NA |
| OTU10319 | NA | NA | NA | NA |
| OTU13460 | NA | NA | NA | NA |
| OTU1245 | NA | - | NA | + |
| OTU14406 | + | NA | - | NA |
| OTU13469 | NA | NA | NA | NA |
| OTU16922 | NA | NA | NA | + |
| OTU13916 | NA | NA | - | + |
| OTU18249 | - | NA | + | NA |
| OTU9210 | NA | NA | NA | NA |
| OTU5568 | + | NA | - | NA |
| OTU4874 | NA | NA | + | NA |
| OTU18295 | - | NA | NA | NA |
| OTU9924 | + | NA | NA | NA |
| OTU6694 | NA | NA | NA | NA |
| OTU11377 | + | - | NA | NA |
| OTU5269 | NA | NA | NA | + |
| OTU7530 | NA | NA | NA | NA |
| OTU15100 | + | NA | - | NA |
| OTU14959 | + | NA | NA | - |
| OTU11800 | NA | NA | + | - |
| OTU13300 | + | NA | - | NA |
| OTU13686 | NA | NA | NA | - |
| OTU15474 | NA | NA | NA | + |
| OTU15106 | NA | NA | NA | NA |
| OTU13932 | NA | NA | NA | NA |
| OTU14355 | - | + | NA | NA |
| OTU16315 | NA | - | NA | + |
| OTU3209 | NA | NA | NA | NA |
| OTU18185 | + | NA | NA | NA |
| OTU2018 | NA | NA | NA | NA |
| OTU18920 | NA | NA | NA | NA |
| OTU3378 | NA | - | + | NA |
| OTU17439 | NA | NA | NA | - |
| OTU18404 | NA | NA | NA | NA |
| OTU16043 | NA | NA | NA | NA |
| OTU7815 | + | NA | NA | NA |
| OTU12590 | NA | - | + | - |
| OTU6198 | NA | NA | NA | NA |
| OTU17693 | NA | NA | - | + |
| OTU2996 | NA | NA | NA | NA |
| OTU15419 | NA | - | NA | NA |
| OTU4998 | + | NA | NA | NA |
| OTU13267 | + | NA | - | NA |
| OTU18067 | NA | NA | NA | + |
| OTU6334 | NA | NA | NA | NA |
| OTU4472 | - | + | NA | NA |
| OTU16650 | - | NA | - | + |
| OTU13208 | NA | NA | NA | NA |
| OTU1112 | + | NA | NA | NA |
| OTU18035 | + | - | NA | NA |
| OTU1387 | NA | NA | NA | NA |
| OTU6293 | - | NA | NA | NA |
| OTU13452 | + | NA | NA | NA |
| OTU13532 | + | NA | NA | NA |
| OTU840 | NA | NA | + | NA |
| OTU2972 | NA | NA | NA | NA |
| OTU13640 | + | - | NA | NA |
| OTU6194 | NA | NA | NA | NA |
| OTU17583 | NA | NA | NA | NA |
| OTU6160 | + | NA | NA | NA |
| OTU3143 | NA | NA | NA | NA |
| OTU14766 | NA | NA | NA | NA |
| OTU17424 | NA | NA | + | - |
| OTU14812 | NA | NA | NA | - |
| OTU17520 | NA | NA | NA | NA |
| OTU6006 | NA | NA | - | NA |
| OTU15138 | + | NA | NA | NA |
| OTU3545 | + | NA | NA | NA |
| OTU10234 | NA | NA | NA | NA |
| OTU5429 | NA | NA | + | NA |
| OTU6497 | + | NA | NA | NA |
| OTU11555 | NA | NA | + | - |
| OTU17479 | NA | + | NA | NA |
| OTU17727 | NA | - | NA | NA |
| OTU9627 | NA | NA | + | NA |
| OTU17638 | NA | NA | NA | NA |
| OTU19143 | + | NA | NA | NA |
| OTU1167 | + | - | - | NA |
| OTU7866 | NA | NA | NA | NA |
| OTU10696 | NA | NA | NA | + |
| OTU18409 | NA | NA | - | + |
| OTU17866 | NA | NA | + | - |
| OTU16014 | NA | NA | NA | NA |
| OTU15028 | NA | NA | + | NA |
| OTU12397 | + | - | NA | NA |
| OTU15974 | + | NA | NA | NA |
| OTU16091 | - | NA | NA | NA |
| OTU15190 | NA | NA | - | NA |
| OTU2929 | NA | NA | - | + |
| OTU4837 | - | NA | NA | + |
| OTU10010 | NA | - | NA | + |
| OTU15746 | NA | - | NA | NA |
| OTU1321 | + | - | NA | - |
| OTU18829 | + | NA | NA | NA |
| OTU126 | NA | NA | + | - |
| OTU1153 | + | NA | NA | - |
| OTU4692 | NA | NA | NA | NA |
| OTU13210 | NA | NA | NA | NA |
| OTU14961 | + | NA | NA | NA |
| OTU13157 | + | NA | NA | NA |
| OTU5898 | NA | NA | NA | NA |
| OTU5981 | NA | NA | NA | NA |
| OTU16349 | - | NA | NA | NA |
| OTU13962 | NA | NA | NA | NA |
| OTU18411 | NA | NA | NA | NA |
| OTU5923 | NA | NA | NA | NA |
| OTU6323 | NA | NA | NA | NA |
| OTU15604 | NA | NA | NA | NA |
| OTU8552 | NA | NA | NA | NA |
| OTU18196 | NA | NA | NA | NA |
| OTU15764 | NA | NA | NA | NA |
| OTU4723 | NA | NA | NA | NA |
| OTU12536 | + | - | NA | NA |
| OTU6311 | NA | NA | NA | NA |
| OTU6460 | NA | NA | NA | NA |
| OTU17738 | NA | NA | - | NA |
| OTU15219 | + | - | NA | NA |
| OTU8736 | NA | NA | NA | NA |
| OTU8479 | + | NA | - | NA |
| OTU1734 | NA | NA | NA | NA |
| OTU8252 | NA | NA | NA | NA |
| OTU9650 | NA | NA | - | NA |
| OTU4793 | NA | NA | + | NA |
| OTU950 | NA | NA | - | NA |
| OTU15783 | NA | NA | NA | + |
| OTU18114 | NA | - | NA | + |
| OTU7839 | NA | NA | - | NA |
| OTU18169 | + | NA | NA | NA |
| OTU14744 | NA | NA | NA | - |
| OTU14375 | NA | NA | NA | NA |
| OTU13411 | + | NA | NA | NA |
| OTU18104 | NA | NA | NA | NA |
| OTU13017 | NA | NA | NA | NA |
| OTU19060 | NA | + | NA | NA |
| OTU14432 | NA | NA | NA | NA |
| OTU13744 | NA | NA | NA | NA |
| OTU11678 | NA | NA | NA | NA |
| OTU5244 | NA | NA | - | + |
| OTU587 | NA | - | NA | NA |
| OTU17010 | + | - | NA | NA |
| OTU5757 | NA | NA | - | NA |
| OTU15975 | NA | NA | NA | + |
| OTU18224 | NA | NA | NA | + |
| OTU13161 | + | NA | NA | NA |
| OTU18349 | NA | NA | + | - |
| OTU7757 | NA | + | NA | NA |
| OTU5417 | NA | NA | NA | NA |
| OTU5868 | - | + | + | NA |
| OTU848 | + | - | NA | NA |
| OTU8017 | NA | NA | - | + |
| OTU1974 | NA | NA | NA | NA |
| OTU8927 | NA | NA | - | NA |
| OTU18617 | NA | NA | NA | NA |
| OTU15530 | NA | NA | NA | NA |
| OTU5280 | NA | - | NA | + |
| OTU2005 | NA | NA | NA | NA |
| OTU14690 | NA | NA | + | NA |
| OTU15112 | NA | NA | - | NA |
| OTU18316 | NA | NA | NA | + |
| OTU6720 | NA | NA | NA | NA |
| OTU10651 | NA | NA | - | + |
| OTU933 | NA | NA | NA | NA |
| OTU1658 | NA | NA | - | NA |
| OTU12621 | NA | NA | NA | NA |
| OTU5292 | NA | NA | NA | NA |
| OTU7588 | NA | NA | - | NA |
| OTU16390 | NA | - | NA | NA |
| OTU13849 | NA | NA | NA | NA |
| OTU2555 | NA | NA | NA | NA |
| OTU14580 | + | NA | - | NA |
| OTU18424 | NA | NA | NA | NA |
| OTU13948 | NA | NA | NA | NA |
| OTU12439 | NA | NA | NA | NA |
| OTU8189 | NA | NA | NA | NA |
| OTU1961 | NA | NA | - | + |
| OTU8035 | + | NA | - | NA |
| OTU15067 | NA | NA | NA | NA |
| OTU783 | + | NA | NA | NA |
| OTU9170 | NA | NA | NA | NA |
| OTU17692 | NA | NA | NA | + |
| OTU1616 | - | NA | NA | + |
| OTU5334 | - | + | NA | NA |
| OTU13018 | + | NA | NA | NA |
| OTU15128 | NA | + | NA | NA |
| OTU10281 | NA | NA | NA | NA |
| OTU2930 | NA | NA | NA | NA |
| OTU7913 | + | NA | NA | NA |
| OTU17166 | - | NA | NA | NA |
| OTU4431 | NA | NA | NA | NA |
| OTU5785 | NA | NA | NA | + |
| OTU7937 | + | NA | - | NA |
| OTU1088 | NA | NA | - | NA |
| OTU8968 | NA | NA | NA | NA |
| OTU7851 | + | NA | - | NA |
| OTU1665 | NA | NA | NA | NA |
| OTU17911 | NA | NA | NA | NA |
| OTU7565 | + | NA | NA | NA |
| OTU6810 | NA | NA | NA | NA |
| OTU6993 | NA | NA | NA | NA |
| OTU1374 | NA | NA | NA | NA |
| OTU14909 | NA | NA | + | NA |
| OTU18222 | NA | NA | NA | NA |
| OTU5935 | NA | NA | + | NA |
| OTU4605 | NA | NA | NA | NA |
| OTU2678 | + | NA | NA | NA |
| OTU14993 | NA | - | NA | NA |
| OTU13390 | NA | + | NA | NA |
| OTU7623 | + | NA | - | NA |
| OTU543 | + | - | NA | NA |
| OTU14992 | NA | NA | NA | NA |
| OTU18353 | NA | NA | NA | NA |
| OTU19187 | + | - | NA | NA |
| OTU14064 | NA | NA | NA | - |
| OTU16920 | + | NA | NA | NA |
| OTU6617 | NA | NA | NA | NA |
| OTU7460 | NA | NA | NA | NA |
| OTU1019 | NA | NA | NA | NA |
| OTU11310 | - | NA | NA | NA |
| OTU3738 | NA | - | NA | NA |
| OTU5112 | NA | - | NA | + |
| OTU19121 | NA | - | NA | NA |
| OTU14224 | NA | NA | NA | - |
| OTU1140 | + | NA | NA | NA |
| OTU5411 | NA | NA | NA | NA |
| OTU18233 | - | NA | NA | NA |
| OTU4376 | + | NA | NA | NA |
| OTU10259 | + | - | NA | NA |
| OTU11725 | + | NA | NA | - |
| OTU11605 | NA | NA | - | NA |
| OTU3244 | NA | NA | NA | NA |
| OTU3640 | + | NA | NA | NA |
| OTU14107 | NA | NA | + | NA |
| OTU2159 | NA | - | NA | NA |
| OTU17704 | NA | NA | NA | NA |
| OTU1147 | NA | NA | NA | NA |
| OTU11474 | NA | - | NA | NA |
| OTU17941 | NA | NA | NA | NA |
| OTU14186 | NA | NA | NA | NA |
| OTU2074 | NA | NA | NA | + |
| OTU4722 | NA | NA | NA | NA |
| OTU7774 | NA | NA | NA | NA |
| OTU5848 | + | NA | NA | NA |
| OTU9155 | NA | NA | - | NA |
| OTU5556 | NA | + | NA | NA |
| OTU1635 | NA | NA | NA | NA |
| OTU13039 | NA | NA | NA | NA |
| OTU9955 | NA | NA | NA | NA |
| OTU18341 | NA | NA | NA | NA |
| OTU18583 | + | NA | NA | NA |
| OTU3549 | NA | NA | NA | NA |
| OTU2040 | NA | NA | NA | - |
| OTU5241 | NA | NA | NA | NA |
| OTU18449 | NA | NA | - | NA |
| OTU16794 | NA | NA | NA | + |
| OTU1841 | NA | NA | NA | NA |
| OTU7473 | + | NA | NA | NA |
| OTU17068 | NA | NA | NA | NA |
| OTU15181 | NA | NA | + | NA |
| OTU6439 | + | NA | - | NA |
| OTU5890 | NA | NA | NA | NA |
| OTU18966 | + | NA | NA | NA |
| OTU2612 | NA | NA | NA | + |
| OTU7761 | NA | NA | NA | NA |
| OTU13873 | NA | - | NA | NA |
| OTU3953 | NA | NA | NA | NA |
| OTU7552 | + | NA | NA | NA |
| OTU7781 | + | NA | - | NA |
| OTU14138 | NA | NA | NA | - |
| OTU5528 | NA | - | + | NA |
| OTU2116 | NA | - | NA | NA |
| OTU12588 | NA | NA | + | NA |
| OTU18231 | - | NA | NA | NA |
| OTU1890 | NA | NA | NA | NA |
| OTU2806 | NA | NA | NA | NA |
| OTU16626 | NA | NA | NA | + |
| OTU13406 | NA | NA | NA | NA |
| OTU1344 | NA | NA | NA | NA |
| OTU4651 | NA | NA | - | NA |
| OTU14689 | NA | NA | NA | NA |
| OTU14880 | + | NA | NA | NA |
| OTU13784 | NA | NA | NA | NA |
| OTU11906 | NA | - | NA | + |
| OTU7622 | NA | NA | NA | NA |
| OTU15556 | NA | NA | NA | NA |
| OTU770 | + | NA | NA | NA |
| OTU3588 | NA | NA | NA | + |
| OTU1844 | NA | + | NA | NA |
| OTU19094 | NA | - | NA | NA |
| OTU15332 | + | NA | NA | NA |
| OTU10129 | NA | NA | NA | NA |
| OTU7865 | - | + | - | + |
| OTU9774 | + | NA | - | NA |
| OTU3604 | NA | NA | + | - |
| OTU14098 | NA | NA | NA | NA |
| OTU17848 | NA | NA | NA | NA |
| OTU5631 | NA | NA | - | NA |
| OTU14906 | NA | NA | NA | - |
| OTU6429 | NA | NA | NA | - |
| OTU9311 | NA | NA | NA | NA |
| OTU1391 | NA | NA | - | NA |
| OTU18137 | - | + | NA | NA |
| OTU8378 | + | NA | NA | NA |
| OTU13701 | + | NA | NA | NA |
| OTU1714 | NA | NA | + | - |
| OTU18239 | NA | NA | NA | - |
| OTU5906 | - | NA | NA | NA |
| OTU10206 | NA | NA | NA | NA |
| OTU13155 | NA | NA | NA | NA |
| OTU1817 | NA | NA | NA | NA |
| OTU7541 | NA | + | NA | NA |
| OTU13223 | NA | NA | + | NA |
| OTU7727 | + | NA | NA | NA |
| OTU17899 | NA | NA | NA | NA |
| OTU1198 | + | NA | NA | NA |
| OTU9106 | + | NA | - | NA |
| OTU18393 | NA | NA | NA | - |
| OTU14130 | NA | NA | NA | NA |
| OTU18119 | NA | NA | NA | NA |
| OTU13554 | + | NA | NA | NA |
| OTU15297 | + | - | NA | NA |
| OTU9581 | NA | NA | NA | NA |
| OTU17660 | NA | NA | + | NA |
| OTU9574 | NA | NA | NA | NA |
| OTU6496 | + | NA | - | NA |
| OTU3709 | NA | NA | NA | NA |
| OTU10231 | NA | NA | NA | NA |
| OTU18225 | NA | NA | NA | NA |
| OTU8735 | NA | NA | NA | NA |
| OTU17549 | - | + | - | NA |
| OTU3525 | + | - | NA | NA |
| OTU7560 | + | NA | NA | NA |
| OTU15559 | NA | NA | NA | NA |
| OTU1608 | NA | + | NA | NA |
| OTU6467 | NA | NA | NA | NA |
| OTU7813 | + | NA | NA | NA |
| OTU1730 | NA | NA | NA | NA |
| OTU3797 | NA | NA | NA | NA |
| OTU589 | NA | NA | - | NA |
| OTU4209 | NA | NA | NA | - |
| OTU3353 | + | NA | - | NA |
| OTU1544 | NA | NA | NA | - |
| OTU14082 | NA | NA | + | NA |
| OTU13555 | NA | NA | NA | NA |
| OTU17674 | - | NA | NA | NA |
| OTU18361 | + | - | NA | NA |
| OTU18016 | NA | NA | NA | + |
| OTU4623 | NA | NA | NA | NA |
| OTU1926 | NA | NA | - | NA |
| OTU17775 | NA | NA | NA | + |
| OTU15130 | NA | NA | NA | + |
| OTU925 | NA | NA | NA | NA |
| OTU1715 | NA | NA | - | NA |
| OTU13841 | + | NA | NA | NA |
| OTU7877 | NA | NA | NA | NA |
| OTU9146 | NA | + | - | NA |
| OTU14542 | + | NA | NA | NA |
| OTU4695 | NA | NA | NA | - |
| OTU1815 | + | NA | NA | NA |
| OTU7858 | NA | - | NA | NA |
| OTU2079 | NA | NA | NA | NA |
| OTU17109 | NA | NA | NA | + |
| OTU12484 | NA | NA | + | NA |
| OTU5727 | NA | NA | - | NA |
| OTU13045 | + | NA | - | NA |
| OTU17536 | NA | NA | NA | NA |
| OTU7417 | + | NA | - | NA |
| OTU14227 | NA | - | NA | + |
| OTU6289 | NA | NA | NA | NA |
| OTU629 | + | - | NA | NA |
| OTU9173 | - | NA | NA | NA |
| OTU11593 | NA | - | NA | + |
| OTU9245 | NA | NA | NA | NA |
| OTU13296 | + | NA | NA | NA |
| OTU9252 | NA | NA | NA | NA |
| OTU14918 | NA | NA | NA | NA |
| OTU15976 | + | NA | - | NA |
| OTU8322 | NA | - | NA | + |
| OTU5337 | NA | + | NA | NA |
| OTU7050 | NA | NA | + | - |
| OTU11995 | NA | NA | NA | NA |
| OTU13241 | + | - | NA | NA |
| OTU1626 | NA | NA | - | + |
| OTU1090 | + | NA | NA | NA |
| OTU1180 | NA | NA | NA | NA |
| OTU8600 | NA | NA | NA | + |
| OTU7797 | NA | NA | NA | - |
| OTU2833 | + | - | NA | NA |
| OTU8225 | NA | NA | NA | NA |
| OTU14292 | NA | NA | - | NA |
| OTU7874 | - | + | NA | NA |
| OTU14088 | NA | NA | NA | NA |
| OTU5560 | NA | NA | NA | NA |
| OTU9032 | NA | NA | NA | NA |
| OTU17943 | + | NA | NA | NA |
| OTU16477 | NA | NA | - | + |
| OTU1190 | + | NA | NA | NA |
| OTU17772 | NA | NA | NA | + |
| OTU8956 | + | NA | NA | NA |
| OTU5183 | NA | NA | NA | NA |
| OTU4618 | NA | NA | NA | + |
| OTU11487 | NA | NA | NA | NA |
| OTU3443 | + | NA | NA | NA |
| OTU7378 | - | + | NA | NA |
| OTU9059 | NA | NA | NA | - |
| OTU18197 | NA | NA | NA | NA |
| OTU8830 | + | NA | NA | NA |
| OTU7477 | NA | NA | NA | NA |
| OTU18240 | NA | NA | NA | NA |
| OTU9595 | NA | NA | NA | NA |
| OTU11984 | NA | NA | NA | NA |
| OTU3878 | NA | NA | NA | NA |
| OTU15603 | NA | NA | NA | NA |
| OTU7965 | + | NA | NA | NA |
| OTU15713 | NA | NA | NA | NA |
| OTU7712 | NA | - | NA | NA |
| OTU9762 | NA | NA | NA | NA |
| OTU9634 | NA | NA | NA | NA |
| OTU12379 | NA | NA | NA | + |
| OTU15024 | NA | + | NA | - |
| OTU13783 | NA | NA | NA | NA |
| OTU1177 | NA | NA | NA | NA |
| OTU13599 | NA | NA | NA | NA |
| OTU3891 | NA | NA | NA | NA |
| OTU10659 | NA | NA | - | + |
| OTU18672 | NA | NA | - | NA |
| OTU8976 | + | NA | NA | NA |
| OTU6205 | NA | NA | NA | NA |
| OTU2112 | NA | NA | NA | NA |
| OTU14954 | NA | NA | NA | NA |
| OTU16037 | NA | + | NA | NA |
| OTU1593 | NA | NA | + | NA |
| OTU18414 | NA | + | - | + |
| OTU14540 | NA | NA | NA | NA |
| OTU13309 | NA | NA | NA | NA |
| OTU1721 | NA | NA | NA | NA |
| OTU17036 | NA | NA | - | + |
| OTU8714 | NA | NA | + | NA |
| OTU7343 | NA | NA | NA | NA |
| OTU7640 | NA | NA | NA | NA |
| OTU8489 | NA | NA | NA | NA |
| OTU1277 | + | NA | - | NA |
| OTU8957 | NA | NA | NA | NA |
| OTU3667 | NA | NA | NA | NA |
| OTU17071 | - | NA | NA | NA |
| OTU13048 | NA | - | + | - |
| OTU6410 | - | + | - | NA |
| OTU16389 | + | - | NA | NA |
| OTU7134 | + | NA | NA | NA |
| OTU12991 | NA | NA | NA | NA |
| OTU8048 | NA | NA | NA | NA |
| OTU17560 | NA | NA | NA | NA |
| OTU11307 | - | NA | NA | NA |
| OTU13785 | + | NA | - | NA |
| OTU17258 | + | NA | NA | NA |
| OTU3843 | + | NA | NA | NA |
| OTU14076 | NA | NA | NA | NA |
| OTU13865 | NA | - | + | NA |
| OTU4923 | NA | NA | + | NA |
| OTU2766 | + | NA | NA | NA |
| OTU15012 | NA | NA | NA | NA |
| OTU5750 | NA | NA | NA | NA |
| OTU10748 | NA | NA | NA | NA |
| OTU177 | + | NA | NA | NA |
| OTU14002 | + | NA | NA | NA |
| OTU18120 | NA | NA | NA | - |
| OTU3737 | NA | NA | NA | - |
| OTU17982 | NA | NA | - | NA |
| OTU17668 | NA | NA | NA | + |
| OTU1957 | + | NA | NA | NA |
| OTU17521 | NA | NA | + | - |
| OTU14228 | + | NA | NA | NA |
| OTU2832 | + | NA | - | NA |
| OTU2588 | + | NA | - | NA |
| OTU7390 | NA | NA | NA | NA |
| OTU14348 | - | NA | + | NA |
| OTU9238 | + | NA | NA | NA |
| OTU5559 | NA | NA | NA | NA |
| OTU18133 | - | + | NA | NA |
| OTU18136 | NA | - | + | NA |
| OTU4087 | NA | NA | + | - |
| OTU11719 | NA | NA | + | NA |
| OTU10743 | NA | - | NA | NA |
| OTU6185 | + | NA | NA | NA |
| OTU13992 | NA | NA | NA | NA |
| OTU7795 | + | NA | NA | NA |
| OTU5065 | - | NA | NA | NA |
| OTU14065 | NA | NA | NA | NA |
| OTU15844 | NA | NA | - | + |
| OTU12247 | NA | NA | NA | NA |
| OTU14085 | + | NA | NA | NA |
| OTU8867 | NA | NA | NA | NA |
| OTU18348 | NA | NA | - | + |
| OTU17233 | NA | NA | - | + |
| OTU140 | NA | NA | NA | NA |
| OTU16928 | + | NA | NA | NA |
| OTU4712 | NA | NA | - | + |
| OTU6266 | - | NA | NA | NA |
| OTU18051 | - | NA | NA | + |
| OTU14515 | NA | NA | NA | NA |
| OTU13881 | NA | NA | + | - |
| OTU1249 | NA | NA | NA | NA |
| OTU7736 | NA | NA | NA | + |
| OTU18210 | NA | NA | NA | NA |
| OTU7119 | NA | NA | NA | NA |
| OTU14419 | NA | NA | NA | - |
| OTU14823 | NA | NA | NA | NA |
| OTU13436 | NA | NA | NA | - |
| OTU1414 | + | NA | NA | - |
| OTU91 | NA | - | NA | NA |
| OTU7760 | NA | NA | NA | + |
| OTU15919 | NA | NA | NA | NA |
| OTU13305 | NA | NA | NA | NA |
| OTU17629 | NA | NA | NA | + |
| OTU4150 | + | NA | NA | NA |
| OTU14277 | NA | NA | NA | NA |
| OTU13122 | NA | NA | NA | NA |
| OTU5096 | NA | NA | NA | NA |
| OTU4398 | NA | NA | NA | NA |
| OTU13369 | NA | NA | NA | NA |
| OTU5590 | NA | NA | NA | NA |
| OTU17677 | NA | NA | NA | NA |
| OTU17014 | NA | NA | NA | NA |
| OTU4656 | NA | NA | NA | + |
| OTU14697 | NA | - | NA | + |
| OTU3361 | NA | - | NA | + |
| OTU7629 | + | NA | NA | NA |
| OTU14841 | NA | NA | NA | NA |
| OTU3755 | NA | NA | - | NA |
| OTU17348 | + | NA | - | NA |
| OTU14625 | NA | + | NA | NA |
| OTU10418 | NA | NA | - | + |
| OTU3367 | NA | NA | NA | - |
| OTU13601 | NA | NA | NA | NA |
| OTU5120 | NA | NA | NA | NA |
| OTU5733 | NA | NA | NA | NA |
| OTU9716 | NA | NA | NA | NA |
| OTU7771 | NA | NA | NA | - |
| OTU5603 | - | NA | + | NA |
| OTU5801 | NA | NA | NA | NA |
| OTU14768 | NA | NA | NA | NA |
| OTU4491 | + | NA | NA | NA |
| OTU2168 | NA | NA | NA | NA |
| OTU4913 | - | + | NA | NA |
| OTU2821 | + | NA | NA | NA |
| OTU17256 | NA | - | + | NA |
| OTU186 | + | NA | NA | - |
| OTU13709 | NA | NA | - | + |
| OTU14658 | + | NA | NA | NA |
| OTU1418 | NA | NA | - | NA |
| OTU14725 | NA | NA | + | NA |
| OTU14289 | NA | NA | + | NA |
| OTU15909 | NA | NA | NA | + |
| OTU7402 | + | NA | - | NA |
| OTU13898 | + | NA | - | NA |
| OTU13974 | NA | NA | NA | NA |
| OTU639 | NA | - | NA | NA |
| OTU2429 | + | NA | - | NA |
| OTU17159 | + | - | NA | NA |
| OTU912 | + | NA | - | + |
| OTU5725 | NA | NA | NA | NA |
| OTU7776 | + | NA | - | NA |
| OTU6379 | NA | NA | + | NA |
| OTU6733 | - | NA | + | - |
| OTU2819 | + | NA | NA | NA |
| OTU13679 | + | NA | NA | NA |
| OTU6434 | NA | NA | NA | NA |
| OTU11711 | NA | + | NA | NA |
| OTU7827 | NA | NA | NA | NA |
| OTU10950 | NA | - | NA | NA |
| OTU13909 | NA | NA | NA | NA |
| OTU1735 | NA | NA | NA | NA |
| OTU6230 | NA | NA | NA | NA |
| OTU17717 | NA | NA | NA | NA |
| OTU13884 | + | NA | NA | NA |
| OTU4381 | - | NA | + | NA |
| OTU17602 | NA | NA | NA | NA |
| OTU1392 | + | NA | NA | NA |
| OTU5883 | NA | + | NA | NA |
| OTU17726 | NA | NA | NA | NA |
| OTU18232 | NA | NA | NA | + |
| OTU15194 | + | NA | NA | NA |
| OTU11529 | + | NA | NA | NA |
| OTU5849 | + | NA | - | NA |
| OTU14463 | NA | NA | NA | NA |
| OTU6003 | NA | NA | + | NA |
| OTU15131 | + | NA | NA | NA |
| OTU14460 | - | NA | NA | NA |
| OTU18077 | NA | NA | NA | NA |
| OTU16742 | NA | NA | NA | + |
| OTU11143 | NA | NA | NA | NA |
| OTU11480 | + | NA | NA | NA |
| OTU4181 | NA | NA | NA | NA |
| OTU4420 | NA | NA | - | + |
| OTU1567 | + | NA | NA | NA |
| OTU13137 | NA | NA | NA | NA |
| OTU17844 | NA | NA | NA | + |
| OTU10228 | NA | NA | NA | NA |
| OTU3586 | NA | NA | + | NA |
| OTU15546 | NA | NA | NA | NA |
| OTU5776 | + | NA | NA | NA |
| OTU3644 | NA | NA | NA | NA |
| OTU15337 | NA | NA | NA | + |
| OTU14008 | + | NA | NA | NA |
| OTU15343 | - | NA | + | NA |
| OTU10017 | NA | - | NA | + |
| OTU14428 | NA | NA | NA | NA |
| OTU14530 | NA | NA | NA | NA |
| OTU5501 | NA | NA | - | + |
| OTU17542 | NA | - | NA | NA |
| OTU1184 | + | NA | NA | NA |
| OTU14796 | NA | NA | + | - |
| OTU13499 | NA | NA | NA | NA |
| OTU8261 | + | NA | - | NA |
| OTU14347 | NA | NA | NA | NA |
| OTU1676 | NA | NA | - | + |
| OTU1158 | NA | NA | NA | NA |
| OTU1269 | + | - | NA | NA |
| OTU2025 | NA | NA | NA | NA |
| OTU5376 | NA | NA | NA | NA |
| OTU17892 | NA | NA | NA | + |
| OTU6998 | NA | NA | NA | NA |
| OTU16040 | NA | NA | NA | NA |
| OTU9226 | NA | NA | NA | + |
| OTU9382 | NA | NA | NA | NA |
| OTU13603 | NA | NA | NA | - |
| OTU1325 | + | NA | - | NA |
| OTU18242 | NA | NA | NA | + |
| OTU13886 | NA | NA | + | - |
| OTU18599 | NA | + | NA | NA |
| OTU9922 | NA | NA | + | - |
| OTU1425 | NA | NA | NA | NA |
| OTU13807 | NA | NA | NA | NA |
| OTU6384 | NA | NA | + | NA |
| OTU5587 | NA | NA | NA | NA |
| OTU6247 | + | NA | NA | NA |
| OTU1862 | NA | NA | NA | NA |
| OTU1386 | + | NA | NA | NA |
| OTU18148 | NA | NA | - | NA |
| OTU13547 | NA | NA | NA | NA |
| OTU6645 | NA | NA | - | NA |
| OTU15185 | + | NA | NA | NA |
| OTU5647 | NA | NA | NA | NA |
| OTU7678 | NA | NA | - | + |
| OTU8110 | NA | NA | NA | NA |
| OTU6676 | + | - | + | NA |
| OTU2164 | + | NA | - | NA |
| OTU5536 | NA | NA | - | + |
| OTU199 | NA | NA | NA | NA |
| OTU7337 | + | NA | - | NA |
| OTU14851 | NA | NA | NA | NA |
| OTU7676 | NA | NA | + | NA |
| OTU13797 | + | NA | NA | NA |
| OTU6787 | NA | - | NA | + |
| OTU5909 | NA | NA | NA | NA |
| OTU14025 | NA | NA | + | - |
| OTU6294 | NA | NA | NA | NA |
| OTU7920 | NA | NA | - | NA |
| OTU673 | NA | NA | NA | NA |
| OTU7772 | + | NA | - | NA |
| OTU16409 | NA | NA | NA | + |
| OTU12631 | NA | NA | NA | NA |
| OTU1896 | NA | + | NA | - |
| OTU7593 | NA | NA | NA | NA |
| OTU7889 | NA | + | NA | NA |
| OTU6306 | + | NA | NA | NA |
| OTU7347 | + | NA | NA | NA |
| OTU6328 | NA | NA | + | - |
| OTU9141 | NA | + | NA | NA |
| OTU7686 | NA | NA | NA | NA |
| OTU13933 | NA | NA | - | NA |
| OTU8033 | NA | NA | NA | NA |
| OTU2314 | + | NA | NA | NA |
| OTU9893 | NA | NA | NA | NA |
| OTU1520 | NA | NA | NA | NA |
| OTU14372 | NA | - | NA | NA |
| OTU4436 | + | NA | - | NA |
| OTU6631 | + | NA | - | NA |
| OTU1228 | NA | NA | NA | NA |
| OTU15231 | NA | - | NA | + |
| OTU13222 | NA | NA | NA | NA |
| OTU11704 | - | NA | + | NA |
| OTU11538 | NA | NA | NA | NA |
| OTU12594 | NA | NA | NA | NA |
| OTU1702 | NA | NA | NA | + |
| OTU4136 | NA | NA | NA | NA |
| OTU14384 | NA | NA | NA | - |
| OTU8970 | NA | NA | NA | NA |
| OTU2169 | NA | NA | NA | NA |
| OTU18367 | NA | NA | NA | NA |
| OTU6394 | + | NA | NA | NA |
| OTU19074 | + | NA | NA | NA |
| OTU1746 | NA | NA | NA | NA |
| OTU11147 | + | NA | NA | NA |
| OTU7462 | NA | NA | NA | - |
| OTU533 | NA | NA | NA | NA |
| OTU7821 | NA | NA | NA | NA |
| OTU15045 | NA | NA | NA | NA |
| OTU19122 | + | NA | - | NA |
| OTU5208 | NA | NA | NA | NA |
| OTU6149 | NA | NA | - | NA |
| OTU7403 | NA | NA | - | NA |
| OTU8233 | NA | + | NA | NA |
| OTU7980 | NA | NA | NA | NA |
| OTU10860 | + | NA | NA | NA |
| OTU1150 | + | NA | NA | NA |
| OTU11631 | NA | NA | NA | NA |
| OTU11291 | NA | + | NA | - |
| OTU4675 | NA | NA | + | NA |
| OTU9848 | NA | NA | NA | + |
| OTU13664 | NA | - | NA | NA |
| OTU16230 | NA | NA | NA | NA |
| OTU13417 | NA | NA | NA | NA |
| OTU13393 | + | NA | NA | NA |
| OTU15208 | NA | + | - | + |
| OTU693 | + | NA | NA | NA |
| OTU14448 | + | NA | NA | - |
| OTU11924 | NA | NA | NA | + |
| OTU1753 | - | NA | NA | NA |
| OTU1506 | NA | NA | NA | NA |
| OTU176 | NA | NA | NA | NA |
| OTU13715 | + | NA | NA | NA |
| OTU9099 | NA | NA | NA | - |
| OTU5756 | NA | NA | NA | NA |
| OTU7651 | NA | NA | NA | - |
| OTU3723 | NA | NA | + | NA |
| OTU5535 | + | - | NA | NA |
| OTU7859 | + | NA | NA | NA |
| OTU18838 | NA | NA | NA | NA |
| OTU8465 | NA | NA | NA | + |
| OTU7387 | NA | + | - | NA |
| OTU13936 | NA | NA | NA | NA |
| OTU1929 | NA | NA | NA | - |
| OTU8738 | NA | NA | NA | NA |
| OTU3653 | NA | NA | NA | - |
| OTU9125 | + | NA | NA | NA |
| OTU7957 | NA | NA | NA | NA |
| OTU8724 | NA | NA | NA | NA |
| OTU7823 | NA | + | - | NA |
| OTU2578 | NA | NA | NA | NA |
| OTU10822 | NA | NA | NA | NA |
| OTU5354 | NA | NA | NA | NA |
| OTU1650 | - | NA | NA | NA |
| OTU1754 | NA | NA | + | NA |
| OTU3185 | NA | NA | NA | NA |
| OTU5960 | NA | NA | NA | NA |
| OTU9020 | NA | NA | NA | NA |
| OTU4523 | NA | NA | NA | NA |
| OTU5758 | NA | NA | NA | NA |
| OTU1083 | NA | - | NA | + |
| OTU9839 | NA | NA | NA | NA |
| OTU12450 | NA | NA | NA | NA |
| OTU13395 | + | NA | NA | NA |
| OTU17639 | NA | + | - | NA |
| OTU14960 | NA | + | NA | NA |
| OTU8790 | NA | NA | NA | - |
| OTU13922 | + | NA | NA | NA |
| OTU12271 | + | NA | NA | NA |
| OTU17908 | NA | NA | - | NA |
| OTU15863 | NA | - | NA | NA |
| OTU2070 | NA | NA | NA | NA |
| OTU13743 | + | - | NA | + |
| OTU14777 | NA | NA | NA | NA |
| OTU6760 | + | NA | - | NA |
| OTU17665 | NA | NA | NA | + |
| OTU14391 | NA | NA | NA | NA |
| OTU10636 | NA | NA | NA | NA |
| OTU14031 | + | NA | NA | NA |
| OTU6314 | + | NA | - | NA |
| OTU13905 | + | NA | NA | NA |
| OTU17269 | NA | - | NA | + |
| OTU5579 | NA | NA | NA | NA |
| OTU17284 | NA | - | NA | + |
| OTU7773 | NA | NA | NA | NA |
| OTU7424 | NA | NA | NA | NA |
| OTU380 | + | NA | NA | NA |
| OTU15007 | NA | NA | NA | NA |
| OTU12054 | - | NA | + | NA |
| OTU17559 | NA | NA | NA | NA |
| OTU17599 | NA | NA | NA | NA |
| OTU10153 | + | NA | NA | NA |
| OTU13663 | NA | NA | NA | NA |
| OTU5840 | + | NA | NA | NA |
| OTU1852 | + | NA | NA | NA |
| OTU5442 | NA | + | NA | - |
| OTU13934 | NA | NA | + | - |
| OTU6533 | NA | NA | NA | + |
| OTU16065 | NA | NA | NA | NA |
| OTU5739 | NA | NA | - | + |
| OTU5218 | NA | NA | + | - |
| OTU17196 | NA | NA | NA | NA |
| OTU1417 | + | NA | NA | NA |
| OTU11663 | NA | NA | NA | NA |
| OTU7075 | NA | + | NA | NA |
| OTU13402 | NA | NA | NA | NA |
| OTU17480 | + | - | NA | NA |
| OTU2367 | - | NA | NA | NA |
| OTU8739 | NA | + | NA | - |
| OTU8703 | NA | NA | NA | + |
| OTU2836 | NA | NA | NA | + |
| OTU8761 | NA | NA | NA | NA |
| OTU1728 | NA | NA | NA | NA |
| OTU2068 | NA | NA | NA | + |
| OTU18373 | NA | + | - | NA |
| OTU5949 | NA | NA | NA | NA |
| OTU13707 | NA | NA | NA | NA |
| OTU6491 | NA | NA | - | NA |
| OTU4388 | NA | NA | + | NA |
| OTU12780 | NA | NA | NA | NA |
| OTU13647 | NA | NA | - | NA |
| OTU7801 | NA | NA | NA | NA |
| OTU1947 | NA | NA | NA | - |
| OTU8034 | + | NA | - | NA |
| OTU1113 | NA | + | NA | NA |
| OTU13480 | NA | NA | NA | NA |
| OTU2826 | NA | NA | NA | + |
| OTU6050 | NA | NA | NA | NA |
| OTU6470 | NA | NA | NA | + |
| OTU18983 | NA | NA | NA | NA |
| OTU13076 | NA | NA | NA | NA |
| OTU12992 | NA | NA | + | NA |
| OTU6688 | + | - | NA | NA |
| OTU6981 | NA | NA | NA | NA |
| OTU14356 | NA | NA | NA | NA |
| OTU15075 | NA | NA | NA | NA |
| OTU14024 | + | NA | NA | NA |
| OTU10159 | NA | NA | + | - |
| OTU5173 | NA | NA | NA | NA |
| OTU7887 | NA | NA | NA | NA |
| OTU7542 | + | NA | NA | NA |
| OTU14797 | NA | NA | NA | NA |
| OTU18332 | NA | NA | NA | NA |
| OTU1894 | NA | NA | NA | NA |
| OTU4803 | NA | NA | + | NA |
| OTU4526 | NA | NA | + | - |
| OTU3590 | + | NA | NA | + |
| OTU1876 | NA | NA | NA | NA |
| OTU13433 | + | NA | - | NA |
| OTU6547 | NA | NA | NA | NA |
| OTU13764 | NA | NA | NA | NA |
| OTU7610 | + | NA | - | NA |
| OTU3730 | NA | - | NA | NA |
| OTU15384 | NA | NA | + | NA |
| OTU13574 | + | NA | - | NA |
| OTU13119 | NA | NA | + | NA |
| OTU13776 | NA | NA | NA | NA |
| OTU8647 | NA | + | NA | - |
| OTU13645 | NA | NA | NA | NA |
| OTU11281 | NA | - | NA | + |
| OTU18207 | - | NA | NA | NA |
| OTU16276 | NA | NA | NA | NA |
| OTU5362 | - | NA | NA | NA |
| OTU1927 | NA | NA | NA | - |
| OTU17226 | NA | NA | NA | NA |
| OTU13029 | + | NA | NA | NA |
| OTU18664 | NA | NA | NA | + |
| OTU5311 | + | NA | NA | NA |
| OTU1320 | + | NA | - | NA |
| OTU5086 | NA | NA | NA | NA |
| OTU89 | NA | NA | NA | + |
| OTU8753 | - | NA | + | NA |
| OTU18818 | NA | NA | - | NA |
| OTU8981 | + | NA | NA | NA |
| OTU16239 | NA | NA | NA | NA |
| OTU1672 | NA | NA | NA | NA |
| OTU7871 | - | NA | NA | NA |
| OTU14401 | NA | NA | NA | NA |
| OTU10341 | NA | NA | NA | NA |
| OTU13498 | NA | NA | NA | + |
| OTU3745 | NA | NA | NA | NA |
| OTU17557 | NA | NA | NA | NA |
| OTU1899 | NA | NA | NA | NA |
| OTU7950 | NA | NA | NA | NA |
| OTU13920 | NA | NA | + | - |
| OTU11892 | NA | NA | NA | NA |
| OTU10412 | + | NA | NA | NA |
| OTU5248 | NA | NA | NA | NA |
| OTU1301 | + | NA | NA | NA |
| OTU4667 | NA | NA | NA | + |
| OTU14885 | + | NA | NA | NA |
| OTU14718 | NA | + | NA | NA |
| OTU17694 | NA | + | NA | NA |
| OTU17965 | NA | NA | NA | NA |
| OTU9237 | NA | NA | NA | NA |
| OTU10365 | - | + | NA | NA |
| OTU11592 | NA | NA | NA | NA |
| OTU14535 | NA | NA | NA | NA |
| OTU6820 | NA | NA | NA | NA |
| OTU4233 | NA | NA | NA | NA |
| OTU5596 | NA | NA | NA | + |
| OTU8560 | NA | + | NA | NA |
| OTU14663 | NA | NA | + | NA |
| OTU10644 | - | NA | NA | NA |
| OTU9102 | NA | NA | - | NA |
| OTU6361 | NA | + | NA | NA |
| OTU8000 | + | NA | NA | NA |
| OTU6539 | NA | NA | - | NA |
| OTU13170 | NA | NA | NA | NA |
| OTU4967 | NA | + | NA | NA |
| OTU5333 | NA | NA | - | NA |
| OTU7656 | NA | NA | NA | NA |
| OTU2609 | + | NA | NA | NA |
| OTU13058 | + | NA | NA | NA |
| OTU17880 | NA | NA | NA | NA |
| OTU14020 | + | NA | - | NA |
| OTU2859 | + | NA | - | NA |
| OTU14628 | NA | NA | NA | - |
| OTU15849 | NA | NA | NA | NA |
| OTU5126 | + | NA | NA | NA |
| OTU5106 | NA | NA | NA | NA |
| OTU17333 | NA | NA | NA | NA |
| OTU4433 | NA | NA | NA | NA |
| OTU11827 | NA | NA | NA | NA |
| OTU1766 | NA | NA | + | - |
| OTU13035 | NA | NA | NA | NA |
| OTU7708 | + | NA | NA | NA |
| OTU14464 | NA | NA | + | NA |
| OTU2242 | NA | - | NA | NA |
| OTU17140 | NA | NA | NA | NA |
| OTU5274 | NA | NA | NA | NA |
| OTU1960 | NA | NA | + | - |
| OTU5128 | + | NA | NA | - |
| OTU5003 | NA | NA | NA | NA |
| OTU12886 | - | + | NA | NA |
| OTU6258 | NA | NA | NA | NA |
| OTU1066 | NA | NA | NA | NA |
| OTU6758 | NA | - | NA | NA |
| OTU1453 | + | NA | - | NA |
| OTU9932 | NA | NA | NA | NA |
| OTU18140 | NA | NA | NA | NA |
| OTU17733 | + | NA | NA | - |
| OTU19115 | NA | + | - | NA |
| OTU14496 | NA | NA | NA | NA |
| OTU10185 | NA | NA | NA | NA |
| OTU15057 | NA | + | NA | - |
| OTU5592 | NA | NA | NA | NA |
| OTU8624 | NA | NA | - | + |
| OTU4716 | NA | NA | NA | NA |
| OTU11237 | NA | NA | NA | NA |
| OTU13975 | NA | NA | NA | NA |
| OTU9092 | + | NA | NA | NA |
| OTU17940 | NA | NA | - | + |
| OTU1266 | + | NA | NA | - |
| OTU12676 | NA | NA | NA | NA |
| OTU6737 | + | - | NA | NA |
| OTU17742 | NA | NA | NA | + |
| OTU15635 | NA | NA | NA | NA |
| OTU6337 | + | NA | NA | NA |
| OTU9753 | NA | NA | NA | NA |
| OTU18914 | NA | NA | NA | NA |
| OTU7498 | NA | NA | + | - |
| OTU13252 | NA | NA | NA | NA |
| OTU17966 | + | - | NA | NA |
| OTU10077 | NA | NA | NA | NA |
| OTU2262 | NA | NA | - | NA |
| OTU15228 | + | NA | NA | NA |
| OTU5588 | NA | NA | NA | - |
| OTU18194 | NA | NA | NA | NA |
| OTU7704 | NA | NA | NA | NA |
| OTU13471 | NA | NA | NA | NA |
| OTU14962 | + | NA | NA | - |
| OTU14435 | NA | NA | - | + |
| OTU12601 | + | NA | NA | NA |
| OTU15437 | NA | NA | NA | + |
| OTU10987 | NA | NA | NA | NA |
| OTU15098 | NA | NA | NA | NA |
| OTU330 | + | NA | NA | NA |
| OTU6973 | + | - | NA | NA |
| OTU3345 | + | NA | - | NA |
| OTU6687 | NA | NA | NA | NA |
| OTU8548 | NA | + | NA | - |
| OTU7355 | + | - | NA | NA |
| OTU15230 | NA | - | NA | + |
| OTU11601 | NA | NA | + | NA |
| OTU4562 | NA | - | NA | NA |
| OTU15820 | + | NA | NA | NA |
| OTU5514 | NA | NA | NA | NA |
| OTU2037 | NA | NA | NA | + |
| OTU17680 | - | NA | + | NA |
| OTU18285 | + | NA | NA | NA |
| OTU17654 | NA | NA | NA | NA |
| OTU13171 | + | - | NA | NA |
| OTU986 | + | NA | - | NA |
| OTU5178 | - | NA | + | NA |
| OTU9565 | + | - | NA | - |
| OTU3542 | NA | NA | NA | NA |
| OTU8059 | + | - | NA | NA |
| OTU5372 | NA | NA | NA | NA |
| OTU11871 | + | NA | NA | NA |
| OTU3892 | NA | NA | NA | NA |
| OTU18115 | NA | NA | - | NA |
| OTU14911 | NA | NA | NA | NA |
| OTU344 | NA | NA | NA | NA |
| OTU11244 | NA | NA | NA | NA |
| OTU10561 | NA | NA | + | NA |
| OTU1028 | + | NA | NA | NA |
| OTU5050 | NA | NA | NA | NA |
| OTU643 | + | NA | NA | NA |
| OTU9148 | + | NA | NA | NA |
| OTU1131 | NA | NA | NA | NA |
| OTU2861 | + | NA | - | NA |
| OTU10995 | NA | NA | + | NA |
| OTU15174 | NA | NA | NA | NA |
| OTU6894 | NA | + | NA | NA |
| OTU8496 | - | NA | NA | NA |
| OTU9360 | NA | NA | NA | NA |
| OTU7721 | NA | NA | NA | - |
| OTU14639 | NA | NA | - | NA |
| OTU8666 | NA | NA | NA | NA |
| OTU14719 | + | NA | NA | NA |
| OTU18000 | NA | NA | NA | NA |
| OTU13760 | NA | NA | NA | NA |
| OTU632 | + | NA | NA | NA |
| OTU2385 | + | NA | NA | NA |
| OTU8069 | NA | NA | NA | NA |
| OTU11778 | NA | NA | NA | NA |
| OTU18425 | - | NA | NA | + |
| OTU18440 | NA | NA | NA | - |
| OTU17203 | NA | NA | NA | NA |
| OTU5786 | NA | NA | NA | NA |
| OTU13896 | NA | NA | NA | NA |
| OTU6010 | + | - | NA | NA |
| OTU14048 | NA | + | NA | NA |
| OTU17702 | NA | - | + | NA |
| OTU16348 | NA | - | NA | NA |
| OTU5584 | + | NA | NA | NA |
| OTU8698 | + | NA | NA | NA |
| OTU13269 | - | NA | NA | NA |
| OTU341 | NA | NA | - | NA |
| OTU1179 | + | NA | NA | NA |
| OTU2595 | NA | NA | NA | NA |
| OTU627 | NA | NA | NA | NA |
| OTU10931 | NA | NA | NA | NA |
| OTU16985 | NA | NA | NA | NA |
| OTU14524 | NA | NA | - | + |
| OTU13553 | NA | NA | NA | NA |
| OTU5795 | NA | NA | - | NA |
| OTU2012 | + | - | NA | NA |
| OTU14490 | NA | NA | NA | + |
| OTU6375 | NA | NA | NA | NA |
| OTU13680 | NA | NA | NA | NA |
| OTU13567 | + | NA | NA | NA |
| OTU5732 | NA | NA | NA | NA |
| OTU15031 | NA | NA | NA | NA |
| OTU14879 | NA | NA | + | NA |
| OTU18442 | NA | NA | NA | + |
| OTU7339 | NA | - | NA | NA |
| OTU16953 | NA | NA | - | NA |
| OTU13178 | NA | NA | NA | NA |
| OTU5878 | - | NA | + | NA |
| OTU15626 | NA | + | - | NA |
| OTU15793 | NA | NA | NA | NA |
| OTU3659 | NA | - | NA | NA |
| OTU3744 | + | NA | NA | NA |
| OTU14603 | NA | NA | NA | - |
| OTU6357 | NA | NA | NA | NA |
| OTU9444 | NA | NA | NA | + |
| OTU5636 | NA | NA | NA | + |
| OTU11007 | NA | NA | NA | NA |
| OTU11041 | NA | NA | NA | NA |
| OTU17971 | NA | NA | NA | NA |
| OTU3603 | + | NA | NA | - |
| OTU489 | + | NA | NA | NA |
| OTU7464 | NA | NA | NA | NA |
| OTU6321 | NA | NA | NA | NA |
| OTU8127 | + | NA | NA | NA |
| OTU8982 | NA | NA | + | NA |
| OTU15425 | NA | NA | NA | NA |
| OTU5604 | + | - | NA | NA |
| OTU6005 | + | NA | - | NA |
| OTU17519 | NA | NA | NA | NA |
| OTU14500 | NA | NA | NA | NA |
| OTU5995 | NA | NA | NA | + |
| OTU17523 | NA | NA | NA | + |
| OTU6697 | NA | NA | + | NA |
| OTU12516 | + | - | NA | NA |
| OTU4744 | NA | NA | NA | NA |
| OTU4957 | NA | NA | NA | NA |
| OTU4099 | NA | NA | + | NA |
| OTU430 | + | NA | - | NA |
| OTU1670 | NA | NA | NA | - |
| OTU5645 | + | NA | NA | NA |
| OTU7655 | NA | NA | NA | NA |
| OTU13642 | NA | NA | NA | NA |
| OTU17100 | NA | NA | NA | NA |
| OTU6858 | NA | NA | NA | NA |
| OTU18340 | NA | NA | - | + |
| OTU10895 | - | NA | NA | + |
| OTU5626 | + | NA | NA | NA |
| OTU5924 | NA | NA | - | NA |
| OTU17592 | - | NA | NA | NA |
| OTU15574 | NA | NA | NA | NA |
| OTU8418 | + | NA | - | NA |
| OTU9291 | NA | NA | NA | NA |
| OTU8217 | NA | NA | - | NA |
| OTU10871 | NA | NA | NA | - |
| OTU18168 | - | NA | + | NA |
| OTU1091 | + | NA | NA | NA |
| OTU7607 | NA | NA | NA | + |
| OTU5656 | - | NA | NA | + |
| OTU5984 | + | NA | - | NA |
| OTU7526 | NA | NA | NA | NA |
| OTU2177 | NA | NA | NA | NA |
| OTU13674 | NA | NA | NA | NA |
| OTU15819 | + | NA | NA | NA |
| OTU7903 | + | NA | NA | NA |
| OTU17591 | NA | NA | NA | NA |
| OTU11339 | NA | NA | NA | NA |
| OTU14616 | NA | NA | NA | - |
| OTU8129 | + | NA | - | NA |
| OTU6391 | NA | NA | NA | NA |
| OTU18328 | NA | NA | NA | NA |
| OTU19158 | NA | NA | NA | + |
| OTU14443 | NA | NA | + | - |
| OTU936 | NA | NA | NA | NA |
| OTU2444 | + | NA | NA | NA |
| OTU1907 | NA | NA | NA | NA |
| OTU14080 | NA | NA | NA | NA |
| OTU17690 | NA | NA | NA | NA |
| OTU269 | NA | NA | NA | - |
| OTU9216 | NA | + | NA | NA |
| OTU18299 | NA | NA | - | NA |
| OTU2468 | + | - | NA | + |
| OTU7513 | NA | NA | - | + |
| OTU6862 | NA | NA | NA | - |
| OTU14617 | NA | NA | NA | NA |
| OTU6273 | NA | + | - | NA |
| OTU6275 | NA | NA | NA | + |
| OTU5989 | NA | NA | NA | NA |
| OTU14059 | NA | NA | + | NA |
| OTU1813 | NA | NA | NA | NA |
| OTU4841 | NA | NA | NA | NA |
| OTU934 | + | NA | NA | NA |
| OTU1436 | NA | NA | NA | NA |
| OTU248 | NA | - | NA | NA |
| OTU10849 | NA | NA | NA | NA |
| OTU18082 | NA | NA | + | NA |
| OTU4051 | NA | NA | NA | - |
| OTU17669 | NA | NA | NA | + |
| OTU17222 | NA | NA | NA | NA |
| OTU2058 | NA | NA | NA | NA |
| OTU3787 | + | NA | - | NA |
| OTU17640 | NA | NA | + | NA |
| OTU17034 | NA | NA | + | NA |
| OTU17497 | NA | NA | + | NA |
| OTU8106 | - | NA | NA | + |
| OTU5203 | NA | NA | NA | NA |
| OTU5520 | + | - | NA | NA |
| OTU4535 | NA | NA | NA | NA |
| OTU18766 | NA | NA | NA | NA |
| OTU14934 | NA | NA | NA | NA |
| OTU7131 | NA | NA | NA | NA |
| OTU784 | NA | NA | NA | NA |
| OTU18182 | NA | NA | NA | NA |
| OTU14290 | NA | - | NA | NA |
| OTU17526 | NA | NA | NA | NA |
| OTU7700 | NA | NA | NA | NA |
| OTU13990 | NA | NA | NA | NA |
| OTU7946 | NA | NA | NA | NA |
| OTU1653 | NA | NA | NA | NA |
| OTU8749 | NA | NA | NA | NA |
| OTU17181 | + | NA | - | NA |
| OTU17651 | + | NA | NA | NA |
| OTU5062 | + | NA | NA | NA |
| OTU346 | NA | NA | - | NA |
| OTU15261 | NA | - | NA | NA |
| OTU9659 | - | NA | NA | NA |
| OTU6335 | NA | + | NA | NA |
| OTU7068 | + | NA | NA | NA |
| OTU7589 | NA | NA | NA | NA |
| OTU2256 | NA | NA | NA | NA |
| OTU5722 | + | NA | - | NA |
| OTU14888 | NA | NA | NA | NA |
| OTU15517 | NA | NA | NA | + |
| OTU1640 | NA | - | + | NA |
| OTU18782 | + | NA | - | NA |
| OTU15473 | NA | NA | NA | + |
| OTU14498 | NA | NA | NA | NA |
| OTU14360 | - | NA | NA | NA |
| OTU1098 | NA | NA | NA | NA |
| OTU14600 | + | - | NA | NA |
| OTU18110 | - | + | NA | NA |
| OTU17822 | NA | NA | NA | NA |
| OTU5569 | NA | + | NA | NA |
| OTU10746 | NA | NA | NA | NA |
| OTU13794 | NA | - | NA | NA |
| OTU14924 | NA | NA | - | NA |
| OTU8179 | NA | NA | NA | NA |
| OTU18430 | NA | NA | NA | NA |
| OTU15868 | NA | NA | NA | NA |
| OTU4049 | NA | NA | NA | NA |
| OTU373 | - | + | NA | NA |
| OTU2145 | NA | NA | NA | NA |
| OTU14551 | NA | NA | NA | NA |
| OTU17366 | NA | NA | - | NA |
| OTU2989 | NA | NA | NA | NA |
| OTU1918 | + | NA | NA | - |
| OTU6383 | - | NA | NA | NA |
| OTU3379 | NA | NA | NA | NA |
| OTU7438 | NA | NA | NA | - |
| OTU18613 | + | NA | - | NA |
| OTU3977 | NA | NA | NA | NA |
| OTU14534 | + | NA | NA | - |
| OTU9243 | + | - | NA | NA |
| OTU79 | + | NA | NA | NA |
| OTU396 | NA | NA | NA | NA |
| OTU16991 | + | NA | NA | NA |
| OTU1649 | NA | NA | - | NA |
| OTU5236 | NA | NA | NA | - |
| OTU16528 | NA | NA | NA | NA |
| OTU18152 | NA | NA | - | + |
| OTU6510 | NA | NA | + | NA |
| OTU18802 | NA | NA | NA | NA |
| OTU6045 | NA | + | NA | NA |
| OTU7508 | NA | NA | NA | NA |
| OTU16746 | NA | NA | NA | + |
| OTU6419 | NA | + | NA | - |
| OTU5720 | NA | NA | NA | NA |
| OTU8097 | NA | NA | NA | NA |
| OTU18143 | NA | NA | NA | NA |
| OTU15526 | NA | NA | NA | + |
| OTU8387 | + | NA | NA | NA |
| OTU6262 | NA | NA | NA | NA |
| OTU6287 | NA | NA | NA | NA |
| OTU8361 | + | NA | NA | NA |
| OTU7652 | NA | + | NA | - |
| OTU4231 | NA | NA | NA | NA |
| OTU13120 | NA | NA | NA | NA |
| OTU5672 | NA | NA | NA | NA |
| OTU11572 | + | NA | NA | NA |
| OTU7849 | NA | NA | NA | - |
| OTU5045 | NA | + | - | NA |
| OTU15235 | NA | NA | NA | NA |
| OTU13668 | + | NA | NA | NA |
| OTU441 | + | NA | NA | NA |
| OTU14863 | NA | NA | NA | NA |
| OTU2803 | NA | - | NA | + |
| OTU9177 | NA | NA | - | NA |
| OTU14901 | NA | NA | NA | NA |
| OTU1105 | + | NA | NA | NA |
| OTU10981 | + | NA | NA | NA |
| OTU4180 | NA | + | NA | NA |
| OTU1794 | NA | NA | NA | NA |
| OTU14826 | NA | NA | NA | NA |
| OTU755 | NA | NA | NA | NA |
| OTU18922 | NA | NA | NA | NA |
| OTU14180 | NA | NA | NA | NA |
| OTU13476 | NA | + | NA | - |
| OTU6011 | NA | NA | NA | + |
| OTU15413 | NA | NA | NA | + |
| OTU7619 | + | NA | NA | NA |
| OTU14197 | NA | NA | + | - |
| OTU12316 | + | NA | NA | NA |
| OTU10826 | NA | NA | NA | NA |
| OTU8136 | + | NA | NA | NA |
| OTU7876 | NA | + | NA | NA |
| OTU17664 | NA | NA | NA | NA |
| OTU18103 | NA | NA | + | NA |
| OTU12116 | NA | - | NA | NA |
| OTU16602 | NA | NA | NA | NA |
| OTU127 | NA | NA | + | - |
| OTU9019 | NA | - | + | NA |
| OTU1244 | NA | NA | - | NA |
| OTU13529 | NA | NA | + | - |
| OTU4788 | NA | NA | NA | NA |
| OTU17786 | NA | NA | NA | NA |
| OTU16962 | NA | - | NA | NA |
| OTU17122 | NA | NA | NA | NA |
| OTU9438 | + | NA | NA | NA |
| OTU824 | + | NA | - | NA |
| OTU6324 | NA | NA | NA | NA |
| OTU13481 | NA | NA | - | NA |
| OTU3871 | NA | NA | - | NA |
| OTU12624 | NA | NA | NA | NA |
| OTU14096 | NA | NA | + | NA |
| OTU2820 | NA | NA | - | NA |
| OTU4372 | NA | - | + | NA |
| OTU15988 | NA | - | NA | NA |
| OTU5948 | NA | NA | NA | - |
| OTU9491 | + | NA | NA | NA |
| OTU8713 | NA | NA | NA | - |
| OTU17126 | NA | NA | NA | NA |
| OTU17022 | NA | NA | NA | NA |
| OTU15484 | NA | - | NA | NA |
| OTU7586 | + | NA | NA | NA |
| OTU15528 | NA | NA | NA | NA |
| OTU9953 | NA | NA | + | NA |
| OTU14865 | NA | NA | NA | - |
| OTU6319 | NA | NA | NA | NA |
| OTU18356 | NA | NA | NA | NA |
| OTU5553 | NA | NA | - | + |
| OTU11634 | + | NA | NA | NA |
| OTU7362 | + | NA | NA | NA |
| OTU4311 | NA | NA | + | - |
| OTU9189 | NA | NA | NA | NA |
| OTU14609 | NA | NA | NA | NA |
| OTU1573 | + | NA | - | NA |
| OTU13580 | NA | NA | NA | NA |
| OTU18227 | NA | + | NA | NA |
| OTU7918 | NA | NA | NA | NA |
| OTU8608 | NA | NA | NA | NA |
| OTU12469 | NA | NA | NA | NA |
| OTU17621 | NA | - | NA | NA |
| OTU8670 | NA | NA | NA | + |
| OTU18351 | NA | NA | NA | NA |
| OTU15086 | NA | NA | NA | NA |
| OTU3810 | NA | NA | NA | + |
| OTU18290 | + | NA | NA | NA |
| OTU11623 | NA | NA | NA | NA |
| OTU13773 | + | NA | NA | NA |
| OTU18374 | NA | NA | NA | + |
| OTU18345 | NA | NA | NA | + |
| OTU18288 | + | NA | - | NA |
| OTU17092 | NA | NA | NA | - |
| OTU8962 | + | NA | - | NA |
| OTU13622 | NA | NA | NA | + |
| OTU8226 | + | NA | NA | NA |
| OTU1389 | + | NA | NA | NA |
| OTU8256 | NA | NA | NA | NA |
| OTU18144 | NA | NA | - | + |
| OTU7892 | + | NA | NA | - |
| OTU14862 | NA | NA | NA | NA |
| OTU570 | + | NA | NA | NA |
| OTU313 | + | NA | - | NA |
| OTU15008 | - | + | NA | NA |
| OTU11552 | + | NA | NA | NA |
| OTU9273 | - | NA | NA | NA |
| OTU2184 | NA | NA | - | + |
| OTU3107 | NA | NA | NA | NA |
| OTU6685 | NA | + | - | NA |
| OTU17632 | NA | NA | NA | NA |
| OTU9089 | NA | + | NA | NA |
| OTU1858 | NA | NA | NA | NA |
| OTU12218 | NA | NA | NA | NA |
| OTU6748 | NA | NA | NA | NA |
| OTU14086 | NA | NA | NA | NA |
| OTU4309 | NA | NA | NA | NA |
| OTU465 | NA | NA | NA | NA |
| OTU5850 | NA | NA | NA | NA |
| OTU740 | + | - | NA | NA |
| OTU14979 | NA | NA | NA | NA |
| OTU18014 | NA | NA | NA | NA |
| OTU18546 | NA | NA | NA | NA |
| OTU13836 | NA | - | NA | NA |
| OTU7908 | NA | NA | NA | NA |
| OTU9257 | + | NA | NA | NA |
| OTU15043 | NA | NA | NA | NA |
| OTU2180 | NA | NA | NA | NA |
| OTU8737 | NA | NA | NA | NA |
| OTU15807 | NA | NA | NA | NA |
| OTU5748 | NA | NA | NA | NA |
| OTU6315 | NA | NA | NA | NA |
| OTU4659 | NA | NA | NA | NA |
| OTU13049 | + | NA | NA | NA |
| OTU13484 | NA | NA | NA | NA |
| OTU6706 | NA | NA | NA | NA |
| OTU10791 | NA | NA | NA | NA |
| OTU13579 | + | NA | - | NA |
| OTU671 | NA | NA | NA | NA |
| OTU6987 | NA | NA | NA | NA |
| OTU19055 | NA | NA | NA | NA |
| OTU14506 | NA | + | NA | - |
| OTU6808 | NA | NA | + | - |
| OTU5944 | NA | NA | NA | NA |
| OTU5827 | + | NA | NA | NA |
| OTU2610 | NA | + | NA | NA |
| OTU2714 | NA | NA | NA | - |
| OTU110 | NA | NA | NA | NA |
| OTU15094 | NA | NA | NA | NA |
| OTU1771 | NA | NA | NA | NA |
| OTU14522 | NA | NA | NA | NA |
| OTU3507 | NA | NA | NA | NA |
| OTU8022 | NA | NA | NA | NA |
| OTU7728 | NA | NA | NA | NA |
| OTU13655 | NA | NA | NA | - |
| OTU2163 | NA | NA | NA | NA |
| OTU11746 | NA | NA | NA | + |
| OTU12511 | NA | NA | + | NA |
| OTU14095 | + | NA | NA | NA |
| OTU1631 | NA | NA | NA | NA |
| OTU7351 | + | NA | NA | NA |
| OTU7369 | NA | NA | NA | NA |
| OTU7627 | + | NA | NA | - |
| OTU12617 | - | NA | NA | NA |
| OTU12226 | NA | NA | NA | - |
| OTU505 | + | NA | NA | NA |
| OTU7630 | - | NA | NA | + |
| OTU13034 | + | NA | NA | NA |
| OTU17967 | NA | NA | NA | - |
| OTU9357 | + | NA | NA | NA |
| OTU8764 | + | NA | NA | NA |
| OTU3423 | NA | NA | NA | NA |
| OTU5574 | NA | + | - | NA |
| OTU3884 | NA | - | NA | + |
| OTU9221 | NA | NA | NA | NA |
| OTU1480 | + | NA | NA | NA |
| OTU17569 | NA | NA | NA | NA |
| OTU5149 | NA | + | NA | NA |
| OTU585 | NA | NA | NA | NA |
| OTU1322 | NA | NA | - | NA |
| OTU18714 | + | NA | - | NA |
| OTU13050 | NA | NA | NA | NA |
| OTU9065 | NA | NA | NA | NA |
| OTU569 | + | - | NA | NA |
| OTU994 | + | NA | - | NA |
| OTU15945 | + | - | NA | - |
| OTU14781 | NA | NA | NA | - |
| OTU1944 | NA | NA | NA | + |
| OTU17409 | NA | NA | NA | NA |
| OTU15929 | NA | NA | NA | NA |
| OTU3528 | + | - | NA | NA |
| OTU1360 | + | NA | NA | NA |
| OTU18418 | - | NA | NA | + |
| OTU1713 | NA | NA | NA | NA |
| OTU13030 | NA | NA | NA | NA |
| OTU338 | + | - | NA | + |
| OTU180 | NA | NA | - | NA |
| OTU14173 | NA | NA | - | NA |
| OTU3190 | NA | NA | NA | NA |
| OTU229 | NA | NA | NA | NA |
| OTU342 | + | NA | NA | NA |
| OTU14392 | NA | NA | NA | NA |
| OTU2280 | NA | NA | NA | NA |
| OTU5912 | NA | NA | NA | - |
| OTU8734 | NA | NA | NA | NA |
| OTU9200 | NA | NA | NA | NA |
| OTU9816 | NA | NA | + | NA |
| OTU16052 | NA | NA | NA | NA |
| OTU18773 | NA | - | NA | + |
| OTU6869 | NA | NA | NA | + |
| OTU1326 | NA | NA | NA | NA |
| OTU14698 | NA | + | NA | NA |
| OTU7235 | + | NA | NA | NA |
| OTU7624 | NA | NA | + | - |
| OTU4676 | + | NA | NA | NA |
| OTU14645 | + | NA | NA | NA |
| OTU1255 | + | NA | - | NA |
| OTU11712 | NA | NA | NA | + |
| OTU13630 | NA | NA | NA | NA |
| OTU16364 | + | NA | NA | NA |
| OTU6435 | + | - | NA | NA |
| OTU636 | + | NA | NA | NA |
| OTU1205 | NA | NA | NA | NA |
| OTU7870 | + | NA | - | NA |
| OTU13727 | NA | NA | NA | NA |
| OTU11403 | NA | NA | NA | NA |
| OTU2044 | NA | NA | - | NA |
| OTU1419 | NA | NA | NA | NA |
| OTU13278 | + | NA | NA | - |
| OTU4864 | NA | NA | NA | NA |
| OTU17657 | NA | NA | + | NA |
| OTU18429 | NA | + | NA | NA |
| OTU2377 | + | NA | NA | NA |
| OTU9179 | NA | NA | NA | NA |
| OTU13246 | NA | NA | + | - |
| OTU19103 | + | NA | NA | NA |
| OTU3025 | + | NA | NA | NA |
| OTU13972 | NA | NA | + | NA |
| OTU7829 | NA | NA | NA | NA |
| OTU2894 | NA | NA | NA | NA |
| OTU644 | + | NA | NA | NA |
| OTU14947 | NA | NA | NA | - |
| OTU4437 | - | + | NA | NA |
| OTU6698 | NA | NA | - | NA |
| OTU1889 | NA | + | NA | NA |
| OTU13917 | NA | NA | NA | NA |
| OTU18212 | NA | NA | - | + |
| OTU4133 | NA | NA | NA | + |
| OTU14775 | NA | - | NA | NA |
| OTU15002 | NA | NA | NA | NA |
| OTU13952 | NA | + | NA | - |
| OTU13486 | NA | NA | + | NA |
| OTU765 | + | - | NA | NA |
| OTU13440 | NA | NA | + | - |
| OTU2459 | NA | NA | NA | NA |
| OTU14602 | + | - | NA | NA |
| OTU13689 | + | NA | NA | NA |
| OTU18388 | NA | + | NA | - |
| OTU14566 | NA | NA | NA | NA |
| OTU18658 | NA | NA | NA | NA |
| OTU6166 | NA | NA | - | NA |
| OTU11344 | NA | NA | NA | NA |
| OTU18647 | NA | NA | + | NA |
| OTU7777 | NA | NA | NA | + |
| OTU10266 | + | - | + | - |
| OTU5669 | NA | NA | NA | NA |
| OTU15654 | + | NA | NA | NA |
| OTU19029 | NA | NA | NA | NA |
| OTU1595 | + | NA | NA | NA |
| OTU13090 | NA | NA | NA | - |
| OTU4699 | NA | NA | NA | + |
| OTU17627 | - | NA | NA | NA |
| OTU13596 | + | NA | NA | NA |
| OTU18765 | NA | NA | NA | NA |
| OTU1078 | NA | NA | NA | NA |
| OTU10527 | NA | NA | + | NA |
| OTU1428 | NA | NA | NA | NA |
| OTU6359 | NA | + | NA | - |
| OTU13759 | NA | NA | NA | NA |
| OTU14467 | + | NA | NA | NA |
| OTU554 | NA | NA | NA | + |
| OTU13491 | NA | NA | NA | NA |
| OTU14477 | NA | NA | NA | NA |
| OTU7816 | + | NA | NA | NA |
| OTU1434 | + | - | - | NA |
| OTU6609 | NA | NA | NA | NA |
| OTU14399 | NA | NA | + | NA |
| OTU1237 | NA | NA | NA | NA |
| OTU5115 | NA | NA | NA | NA |
| OTU9735 | NA | NA | NA | NA |
| OTU5213 | NA | NA | NA | NA |
| OTU8327 | NA | + | NA | NA |
| OTU9361 | - | NA | NA | + |
| OTU11519 | NA | NA | + | NA |
| OTU17796 | NA | + | NA | NA |
| OTU14307 | NA | NA | NA | NA |
| OTU602 | + | NA | NA | NA |
| OTU11546 | NA | NA | NA | - |
| OTU10753 | NA | NA | NA | NA |
| OTU7722 | NA | NA | NA | NA |
| OTU14855 | NA | NA | + | - |
| OTU3297 | NA | NA | NA | - |
| OTU12903 | + | NA | NA | NA |
| OTU18187 | NA | NA | NA | NA |
| OTU4511 | NA | NA | NA | NA |
| OTU2202 | + | NA | NA | NA |
| OTU6819 | NA | NA | - | NA |
| OTU6487 | NA | NA | NA | + |
| OTU14834 | + | NA | NA | NA |
| OTU18130 | NA | NA | NA | - |
| OTU17901 | NA | NA | NA | NA |
| OTU11317 | NA | NA | NA | NA |
| OTU13810 | + | NA | NA | NA |
| OTU18677 | NA | NA | NA | NA |
| OTU16379 | NA | NA | NA | NA |
| OTU16804 | NA | - | NA | NA |
| OTU1936 | NA | NA | NA | NA |
| OTU4891 | NA | NA | - | + |
| OTU14956 | - | NA | NA | NA |
| OTU7534 | + | NA | - | NA |
| OTU7442 | NA | NA | NA | NA |
| OTU5864 | NA | NA | NA | NA |
| OTU1814 | NA | NA | NA | NA |
| OTU511 | NA | NA | NA | NA |
| OTU17761 | NA | NA | - | + |
| OTU13244 | NA | NA | NA | NA |
| OTU13072 | NA | + | NA | NA |
| OTU12055 | NA | NA | NA | NA |
| OTU16144 | NA | NA | NA | NA |
| OTU1816 | NA | NA | NA | NA |
| OTU11882 | NA | NA | NA | NA |
| OTU17292 | - | NA | NA | NA |
| OTU5012 | NA | NA | NA | NA |
| OTU9278 | NA | + | NA | NA |
| OTU7383 | NA | NA | NA | NA |
| OTU11637 | NA | NA | + | NA |
| OTU6007 | + | NA | NA | - |
| OTU1577 | NA | NA | NA | NA |
| OTU6588 | NA | + | NA | NA |
| OTU8458 | NA | NA | NA | NA |
| OTU19128 | NA | NA | NA | NA |
| OTU1486 | NA | - | NA | NA |
| OTU7833 | NA | + | NA | NA |
| OTU14582 | NA | NA | NA | + |
| OTU12413 | NA | NA | NA | NA |
| OTU17648 | - | NA | NA | NA |
| OTU10225 | + | NA | NA | NA |
| OTU10321 | NA | NA | NA | NA |
| OTU12299 | NA | NA | NA | NA |
| OTU15579 | NA | NA | NA | NA |
| OTU1500 | NA | NA | NA | NA |
| OTU8672 | NA | + | NA | NA |
| OTU3944 | + | NA | NA | NA |
| OTU1863 | NA | NA | NA | NA |
| OTU7869 | NA | NA | NA | NA |
| OTU18629 | NA | NA | NA | + |
| OTU12988 | NA | NA | NA | NA |
| OTU7748 | NA | NA | NA | NA |
| OTU13761 | NA | NA | NA | NA |
| OTU1644 | NA | NA | NA | - |
| OTU14650 | NA | NA | NA | NA |
| OTU14335 | NA | NA | NA | NA |
| OTU11842 | NA | NA | NA | NA |
| OTU16241 | NA | NA | NA | NA |
| OTU13772 | NA | NA | NA | - |
| OTU273 | + | NA | NA | NA |
| OTU2175 | NA | NA | - | NA |
| OTU4508 | NA | NA | NA | NA |
| OTU1370 | NA | NA | NA | NA |
| OTU3419 | NA | NA | NA | NA |
| OTU16793 | NA | NA | NA | NA |
| OTU7349 | NA | + | NA | NA |
| OTU8185 | NA | NA | NA | + |
| OTU1201 | NA | NA | NA | NA |
| OTU3355 | + | NA | NA | - |
| OTU14067 | NA | NA | NA | NA |
| OTU18291 | + | - | NA | NA |
| OTU5975 | NA | - | NA | NA |
| OTU9328 | + | NA | NA | - |
| OTU6630 | NA | NA | + | NA |
| OTU2939 | NA | NA | NA | NA |
| OTU14420 | NA | NA | NA | NA |
| OTU14055 | NA | NA | NA | NA |
| OTU11473 | NA | NA | + | NA |
| OTU1846 | NA | NA | NA | NA |
| OTU1744 | NA | NA | NA | NA |
| OTU2936 | + | NA | NA | NA |
| OTU5175 | NA | NA | NA | NA |
| OTU15412 | NA | NA | NA | NA |
| OTU5830 | NA | NA | NA | + |
| OTU13930 | + | NA | NA | - |
| OTU14139 | NA | NA | NA | NA |
| OTU12548 | NA | NA | NA | NA |
| OTU10862 | NA | NA | NA | + |
| OTU7699 | NA | NA | - | + |
| OTU13213 | NA | NA | + | NA |
| OTU7909 | + | NA | - | NA |
| OTU13946 | NA | NA | NA | NA |
| OTU13639 | NA | NA | NA | NA |
| OTU1850 | NA | + | NA | NA |
| OTU18422 | NA | NA | NA | NA |
| OTU7787 | NA | NA | NA | NA |
| OTU8716 | + | NA | NA | NA |
| OTU1132 | NA | NA | - | NA |
| OTU14239 | NA | NA | NA | NA |
| OTU666 | NA | NA | NA | - |
| OTU17999 | NA | NA | NA | NA |
| OTU18235 | NA | NA | - | NA |
| OTU18363 | NA | NA | NA | NA |
| OTU14576 | NA | - | NA | NA |
| OTU9879 | NA | NA | NA | - |
| OTU15248 | NA | NA | NA | NA |
| OTU11728 | NA | NA | NA | NA |
| OTU11460 | NA | NA | NA | NA |
| OTU1251 | NA | NA | NA | NA |
| OTU530 | NA | NA | NA | NA |
| OTU13233 | + | - | NA | NA |
| OTU252 | + | - | NA | NA |
| OTU17000 | NA | NA | NA | NA |
| OTU1284 | NA | NA | NA | NA |
| OTU18399 | NA | NA | + | NA |
| OTU17174 | NA | + | NA | NA |
| OTU1057 | NA | NA | NA | NA |
| OTU6481 | - | NA | NA | NA |
| OTU13953 | + | NA | NA | NA |
| OTU4921 | NA | + | NA | NA |
| OTU18696 | NA | NA | NA | NA |
| OTU14440 | + | - | NA | NA |
| OTU5219 | - | + | NA | NA |
| OTU17468 | NA | NA | NA | NA |
| OTU5335 | NA | NA | NA | - |
| OTU1671 | NA | NA | + | NA |
| OTU2334 | + | - | NA | NA |
| OTU18112 | NA | NA | NA | NA |
| OTU791 | NA | NA | NA | NA |
| OTU6270 | NA | NA | NA | - |
| OTU7924 | NA | NA | NA | NA |
| OTU7496 | + | NA | NA | NA |
| OTU3641 | NA | NA | NA | + |
| OTU5798 | NA | NA | NA | NA |
| OTU19145 | NA | NA | NA | NA |
| OTU1347 | NA | NA | NA | NA |
| OTU19086 | NA | NA | NA | NA |
| OTU14485 | NA | + | NA | NA |
| OTU14870 | NA | NA | NA | NA |
| OTU13987 | NA | + | NA | NA |
| OTU2486 | + | NA | NA | NA |
| OTU7439 | NA | NA | - | NA |
| OTU6286 | NA | NA | NA | NA |
| OTU6052 | NA | NA | NA | NA |
| OTU13786 | NA | NA | - | NA |
| OTU17367 | NA | NA | NA | NA |
| OTU3684 | NA | NA | NA | NA |
| OTU9036 | + | NA | NA | NA |
| OTU11046 | NA | NA | NA | NA |
| OTU7742 | NA | NA | NA | NA |
| OTU13105 | NA | NA | NA | - |
| OTU13488 | NA | NA | NA | - |
| OTU14912 | + | NA | NA | NA |
| OTU18476 | NA | NA | - | + |
| OTU12527 | + | NA | NA | NA |
| OTU14678 | NA | NA | NA | NA |
| OTU5575 | NA | NA | + | NA |
| OTU11211 | NA | NA | NA | + |
| OTU17550 | NA | NA | NA | NA |
| OTU10553 | NA | + | NA | - |
| OTU18271 | NA | NA | NA | NA |
| OTU989 | NA | NA | NA | NA |
| OTU2027 | NA | NA | NA | NA |
| OTU2690 | NA | NA | - | NA |
| OTU7786 | NA | NA | NA | NA |
| OTU6297 | NA | NA | NA | NA |
| OTU13087 | NA | NA | NA | NA |
| OTU13711 | NA | NA | + | - |
| OTU17002 | NA | NA | NA | NA |
| OTU10634 | - | NA | + | NA |
| OTU4705 | NA | NA | + | NA |
| OTU1333 | + | NA | NA | NA |
| OTU5046 | NA | NA | NA | NA |
| OTU13441 | NA | NA | NA | NA |
| OTU655 | + | NA | - | NA |
| OTU2799 | + | NA | NA | NA |
| OTU15842 | NA | NA | - | NA |
| OTU9499 | NA | + | NA | - |
| OTU12728 | + | NA | NA | NA |
| OTU13531 | NA | NA | NA | - |
| OTU7755 | + | - | NA | NA |
| OTU5273 | + | NA | NA | NA |
| OTU15743 | NA | NA | NA | NA |
| OTU2088 | + | NA | NA | NA |
| OTU17161 | NA | NA | NA | + |
| OTU16331 | NA | NA | NA | NA |
| OTU18456 | NA | NA | - | + |
| OTU7505 | + | - | - | NA |
| OTU17655 | NA | NA | NA | NA |
| OTU8160 | + | NA | - | NA |
| OTU5500 | NA | NA | NA | NA |
| OTU17294 | NA | NA | NA | NA |
| OTU13592 | NA | NA | NA | NA |
| OTU9543 | NA | - | NA | NA |
| OTU565 | + | - | NA | NA |
| OTU13964 | NA | NA | NA | NA |
| OTU9235 | NA | NA | NA | NA |
| OTU7576 | NA | NA | NA | + |
| OTU9121 | + | NA | - | NA |
| OTU16253 | NA | NA | NA | NA |
| OTU18537 | NA | NA | NA | NA |
| OTU891 | NA | NA | + | - |
| OTU1765 | NA | NA | NA | + |
| OTU13712 | NA | NA | NA | NA |
| OTU372 | NA | NA | NA | NA |
| OTU8565 | NA | NA | NA | NA |
| OTU17570 | NA | NA | NA | NA |
| OTU687 | NA | NA | NA | NA |
| OTU508 | NA | NA | NA | NA |
| OTU17927 | NA | NA | NA | NA |
| OTU1939 | + | NA | NA | - |
| OTU5088 | NA | NA | NA | - |
| OTU13443 | - | + | NA | NA |
| OTU11054 | NA | NA | NA | + |
| OTU7670 | NA | NA | NA | NA |
| OTU9227 | NA | NA | NA | NA |
| OTU13314 | NA | NA | NA | NA |
| OTU7723 | NA | NA | NA | NA |
| OTU11528 | NA | NA | NA | NA |
| OTU9143 | NA | + | NA | NA |
| OTU13751 | NA | NA | NA | NA |
| OTU17137 | NA | - | NA | NA |
| OTU17701 | NA | NA | NA | NA |
| OTU10323 | NA | - | NA | NA |
| OTU7052 | NA | NA | NA | NA |
| OTU7617 | NA | NA | NA | - |
| OTU9080 | NA | + | NA | - |
| OTU13817 | NA | NA | NA | NA |
| OTU17537 | NA | NA | NA | NA |
| OTU7377 | + | NA | NA | NA |
| OTU13391 | NA | NA | NA | - |
| OTU4610 | NA | NA | NA | NA |
| OTU5911 | NA | NA | NA | NA |
| OTU15990 | NA | NA | - | NA |
| OTU14063 | NA | NA | NA | NA |
| OTU5915 | NA | NA | NA | NA |
| OTU7571 | + | NA | NA | NA |
| OTU17078 | + | NA | NA | NA |
| OTU12915 | + | NA | NA | NA |
| OTU4314 | + | NA | NA | NA |
| OTU13032 | + | NA | NA | NA |
| OTU5578 | NA | + | NA | NA |
| OTU4242 | NA | NA | NA | NA |
| OTU128 | + | NA | NA | NA |
| OTU13001 | NA | NA | NA | + |
| OTU17247 | NA | NA | NA | NA |
| OTU17644 | - | NA | NA | + |
| OTU14028 | + | NA | NA | NA |
| OTU14240 | NA | - | + | NA |
| OTU16966 | NA | NA | NA | + |
| OTU12781 | NA | NA | NA | NA |
| OTU15188 | NA | NA | NA | + |
| OTU7684 | NA | + | NA | - |
| OTU13287 | NA | NA | NA | - |
| OTU11488 | NA | NA | NA | + |
| OTU13602 | + | NA | NA | NA |
| OTU4074 | NA | NA | NA | NA |
| OTU15915 | NA | NA | NA | NA |
| OTU19083 | NA | NA | NA | NA |
| OTU9963 | NA | NA | NA | NA |
| OTU13299 | NA | NA | NA | NA |
| OTU714 | + | NA | NA | NA |
| OTU9302 | + | NA | NA | NA |
| OTU11430 | NA | NA | NA | NA |
| OTU5671 | NA | - | + | - |
| OTU6392 | NA | NA | NA | NA |
| OTU12015 | NA | NA | NA | NA |
| OTU9697 | NA | NA | NA | NA |
| OTU4591 | NA | NA | NA | NA |
| OTU15054 | NA | NA | + | - |
| OTU15428 | NA | NA | NA | NA |
| OTU18287 | NA | NA | + | NA |
| OTU5449 | NA | + | NA | - |
| OTU18413 | NA | NA | NA | NA |
| OTU14717 | NA | NA | + | - |
| OTU7458 | + | NA | NA | NA |
| OTU9258 | NA | NA | NA | NA |
| OTU15030 | NA | NA | NA | NA |
| OTU1460 | NA | NA | NA | + |
| OTU13989 | NA | NA | NA | NA |
| OTU10536 | NA | NA | NA | NA |
| OTU11064 | NA | NA | NA | - |
| OTU7313 | - | + | NA | NA |
| OTU13789 | NA | - | + | NA |
| OTU18532 | NA | NA | NA | NA |
| OTU3741 | NA | NA | NA | NA |
| OTU3183 | NA | NA | NA | NA |
| OTU290 | NA | NA | NA | NA |
| OTU3002 | + | NA | NA | NA |
| OTU1166 | + | NA | NA | NA |
| OTU4814 | NA | + | NA | NA |
| OTU8389 | NA | NA | - | NA |
| OTU18434 | NA | NA | NA | NA |
| OTU4875 | NA | NA | + | NA |
| OTU5775 | NA | NA | + | NA |
| OTU3718 | NA | NA | NA | NA |
| OTU10171 | + | NA | NA | NA |
| OTU4842 | NA | NA | NA | NA |
| OTU3546 | NA | NA | - | NA |
| OTU5155 | NA | NA | + | NA |
| OTU13585 | NA | NA | NA | - |
| OTU7572 | + | NA | NA | NA |
| OTU5545 | NA | NA | - | NA |
| OTU14089 | NA | + | NA | NA |
| OTU18383 | NA | NA | NA | + |
| OTU18099 | NA | NA | NA | + |
| OTU14817 | NA | NA | NA | + |
| OTU17209 | NA | NA | NA | NA |
| OTU1537 | + | - | NA | NA |
| OTU6793 | NA | NA | NA | NA |
| OTU4565 | NA | + | - | NA |
| OTU4506 | NA | NA | + | NA |
| OTU6415 | NA | NA | NA | - |
| OTU3785 | NA | - | NA | NA |
| OTU4018 | NA | NA | NA | NA |
| OTU7683 | + | NA | - | NA |
| OTU12709 | NA | NA | NA | NA |
| OTU18331 | - | NA | NA | NA |
| OTU15263 | NA | - | NA | NA |
| OTU2033 | NA | NA | + | - |
| OTU5315 | - | NA | + | NA |
| OTU9690 | NA | NA | NA | NA |
| OTU9108 | NA | NA | NA | NA |
| OTU14407 | NA | NA | + | - |
| OTU8174 | + | NA | - | NA |
| OTU16621 | NA | NA | NA | NA |
| OTU2290 | + | NA | - | NA |
| OTU17198 | NA | NA | NA | + |
| OTU13587 | + | NA | NA | NA |
| OTU2035 | NA | NA | NA | + |
| OTU5764 | NA | NA | NA | NA |
| OTU7735 | NA | NA | - | NA |
| OTU1276 | NA | NA | NA | NA |
| OTU5074 | NA | NA | NA | NA |
| OTU17579 | NA | NA | NA | NA |
| OTU12856 | NA | - | NA | NA |
| OTU328 | NA | - | NA | NA |
| OTU10052 | NA | NA | NA | NA |
| OTU10060 | NA | - | NA | + |
| OTU473 | + | - | NA | NA |
| OTU2294 | NA | NA | NA | NA |
| OTU5251 | NA | NA | NA | NA |
| OTU17132 | NA | NA | NA | + |
| OTU9222 | - | NA | NA | NA |
| OTU9768 | NA | NA | + | NA |
| OTU13617 | NA | NA | + | NA |
| OTU4627 | + | NA | - | NA |
| OTU15853 | + | NA | NA | NA |
| OTU14363 | NA | NA | NA | NA |
| OTU8207 | NA | - | NA | + |
| OTU1497 | NA | - | NA | NA |
| OTU2982 | NA | + | NA | NA |
| OTU5620 | + | NA | NA | NA |
| OTU16818 | NA | + | NA | NA |
| OTU2179 | + | NA | NA | NA |
| OTU10835 | NA | NA | NA | NA |
| OTU3693 | + | - | NA | NA |
| OTU11299 | NA | NA | NA | NA |
| OTU3822 | NA | NA | + | - |
| OTU9834 | NA | NA | NA | NA |
| OTU18238 | NA | NA | NA | NA |
| OTU5825 | NA | NA | NA | NA |
| OTU6832 | + | NA | NA | NA |
| OTU1375 | NA | NA | NA | NA |
| OTU377 | NA | - | NA | NA |
| OTU990 | NA | NA | NA | - |
| OTU15790 | NA | NA | - | + |
| OTU18551 | NA | NA | NA | + |
| OTU17275 | + | NA | NA | - |
| OTU7398 | NA | NA | - | + |
| OTU6604 | + | NA | - | NA |
| OTU9236 | + | NA | NA | NA |
| OTU1523 | NA | NA | NA | NA |
| OTU5565 | NA | NA | NA | NA |
| OTU13959 | + | NA | NA | NA |
| OTU13790 | NA | NA | NA | NA |
| OTU3164 | NA | NA | NA | NA |
| OTU13638 | NA | NA | NA | - |
| OTU16397 | + | NA | NA | NA |
| OTU17970 | + | NA | NA | NA |
| OTU7433 | + | NA | - | NA |
| OTU15001 | - | NA | NA | NA |
| OTU13984 | NA | NA | NA | - |
| OTU5638 | NA | NA | NA | NA |
| OTU351 | + | NA | NA | NA |
| OTU13221 | NA | NA | NA | NA |
| OTU9018 | + | NA | - | NA |
| OTU19154 | NA | NA | NA | NA |
| OTU1824 | NA | NA | NA | NA |
| OTU6704 | + | NA | - | NA |
| OTU13200 | - | NA | - | NA |
| OTU17460 | NA | NA | NA | NA |
| OTU11225 | NA | NA | NA | NA |
| OTU11675 | + | NA | NA | NA |
| OTU7359 | NA | NA | NA | NA |
| OTU2383 | NA | + | NA | NA |
| OTU7430 | NA | NA | NA | NA |
| OTU6402 | NA | NA | NA | NA |
| OTU17679 | NA | NA | NA | NA |
| OTU6462 | NA | NA | NA | NA |
| OTU5252 | NA | NA | NA | + |
| OTU6606 | - | NA | NA | NA |
| OTU11591 | NA | NA | + | NA |
| OTU560 | NA | NA | - | NA |
| OTU9191 | NA | NA | NA | - |
| OTU9256 | NA | + | NA | NA |
| OTU7713 | + | NA | NA | NA |
| OTU14487 | NA | NA | + | NA |
| OTU17205 | NA | NA | NA | NA |
| OTU5803 | NA | NA | NA | NA |
| OTU4934 | NA | NA | NA | NA |
| OTU4697 | NA | NA | NA | NA |
| OTU14844 | NA | NA | NA | NA |
| OTU7944 | + | - | NA | NA |
| OTU16796 | NA | + | NA | NA |
| OTU8955 | NA | NA | NA | NA |
| OTU7694 | NA | + | NA | NA |
| OTU1178 | NA | NA | + | NA |
| OTU13466 | NA | NA | NA | NA |
| OTU15408 | NA | NA | NA | NA |
| OTU1694 | NA | NA | NA | + |
| OTU14895 | NA | NA | NA | NA |
| OTU15255 | NA | NA | - | + |
| OTU16590 | NA | NA | NA | NA |
| OTU17915 | NA | NA | + | NA |
| OTU14179 | NA | NA | NA | NA |
| OTU16850 | NA | NA | NA | NA |
| OTU13335 | NA | NA | NA | NA |
| OTU1964 | NA | NA | NA | NA |
| OTU5640 | NA | NA | NA | NA |
| OTU18157 | - | NA | NA | NA |
| OTU14370 | + | - | NA | NA |
| OTU1984 | NA | NA | NA | + |
| OTU2066 | NA | NA | NA | NA |
| OTU3383 | + | NA | NA | NA |
| OTU7097 | NA | NA | NA | NA |
| OTU4107 | + | NA | NA | - |
| OTU5941 | NA | NA | NA | NA |
| OTU14337 | NA | NA | NA | NA |
| OTU17759 | NA | NA | NA | NA |
| OTU14836 | NA | + | NA | NA |
| OTU6151 | NA | + | NA | NA |
| OTU13778 | NA | NA | NA | NA |
| OTU16923 | + | NA | NA | NA |
| OTU15013 | + | NA | NA | NA |
| OTU14631 | NA | + | NA | - |
| OTU2132 | NA | NA | NA | NA |
| OTU5612 | NA | + | NA | NA |
| OTU6035 | NA | NA | NA | NA |
| OTU571 | NA | NA | + | NA |
| OTU9157 | NA | NA | NA | NA |
| OTU13080 | NA | NA | + | NA |
| OTU12622 | NA | NA | NA | NA |
| OTU17688 | - | NA | NA | NA |
| OTU1987 | NA | NA | - | + |
| OTU9183 | NA | NA | NA | - |
| OTU9029 | NA | NA | NA | + |
| OTU10198 | NA | NA | NA | NA |
| OTU18769 | NA | NA | NA | NA |
| OTU7384 | + | NA | NA | NA |
| OTU389 | NA | NA | NA | NA |
| OTU9282 | NA | NA | NA | NA |
| OTU2986 | NA | NA | NA | NA |
| OTU1615 | NA | NA | NA | NA |
| OTU5747 | NA | - | NA | NA |
| OTU363 | NA | NA | NA | NA |
| OTU17527 | - | + | - | NA |
| OTU8044 | NA | + | NA | NA |
| OTU13479 | NA | NA | NA | NA |
| OTU2673 | NA | NA | NA | NA |
| OTU1073 | + | NA | - | NA |
| OTU9076 | NA | NA | NA | NA |
| OTU7663 | NA | NA | NA | NA |
| OTU8590 | NA | NA | NA | NA |
| OTU17195 | NA | NA | NA | + |
| OTU1253 | NA | NA | + | - |
| OTU9507 | NA | NA | NA | NA |
| OTU6537 | NA | NA | NA | + |
| OTU14291 | NA | NA | NA | NA |
| OTU18161 | NA | NA | NA | + |
| OTU17616 | NA | NA | NA | NA |
| OTU9139 | NA | NA | NA | NA |
| OTU17530 | NA | NA | NA | NA |
| OTU7502 | NA | NA | NA | NA |
| OTU18153 | NA | NA | NA | NA |
| OTU5231 | NA | NA | NA | NA |
| OTU10728 | NA | NA | NA | NA |
| OTU5642 | NA | NA | NA | - |
| OTU9995 | NA | NA | NA | NA |
| OTU9632 | NA | NA | NA | NA |
| OTU7472 | + | NA | - | NA |
| OTU14403 | NA | NA | NA | - |
| OTU13166 | NA | NA | - | NA |
| OTU13183 | NA | NA | NA | NA |
| OTU900 | + | NA | NA | NA |
| OTU18260 | NA | NA | NA | NA |
| OTU4780 | NA | NA | NA | NA |
| OTU9231 | + | NA | NA | NA |
| OTU17903 | NA | NA | NA | + |
| OTU6164 | NA | NA | NA | NA |
| OTU5230 | NA | NA | NA | NA |
| OTU16532 | + | NA | NA | NA |
| OTU10246 | NA | NA | NA | NA |
| OTU256 | + | - | NA | NA |
| OTU11980 | NA | NA | NA | - |
| OTU9123 | NA | NA | NA | NA |
| OTU402 | NA | NA | NA | NA |
| OTU1942 | NA | NA | NA | NA |
| OTU5962 | - | NA | NA | + |
| OTU1359 | NA | NA | - | NA |
| OTU17978 | NA | NA | NA | NA |
| OTU4345 | NA | - | NA | NA |
| OTU1655 | NA | NA | NA | NA |
| OTU18759 | NA | - | NA | NA |
| OTU19008 | NA | NA | NA | NA |
| OTU16139 | NA | + | - | NA |
| OTU5697 | NA | NA | - | NA |
| OTU19170 | + | NA | - | NA |
| OTU6009 | NA | NA | NA | NA |
| OTU13516 | + | NA | NA | NA |
| OTU13513 | NA | NA | NA | - |
| OTU16416 | NA | NA | NA | NA |
| OTU16294 | + | NA | - | NA |
| OTU14232 | NA | NA | + | - |
| OTU1529 | + | NA | NA | NA |
| OTU1951 | NA | NA | NA | NA |
| OTU3175 | + | NA | NA | NA |
| OTU5865 | NA | NA | NA | NA |
| OTU1955 | NA | NA | NA | NA |
| OTU15926 | NA | NA | NA | NA |
| OTU13718 | NA | NA | + | NA |
| OTU12984 | NA | NA | NA | NA |
| OTU5627 | + | NA | - | NA |
| OTU4401 | + | NA | NA | NA |
| OTU2134 | NA | NA | + | - |
| OTU8317 | NA | NA | - | + |
| OTU5067 | NA | + | NA | NA |
| OTU5336 | NA | NA | NA | NA |
| OTU6071 | NA | NA | NA | NA |
| OTU12663 | - | + | NA | NA |
| OTU17728 | NA | NA | NA | NA |
| OTU760 | NA | NA | NA | NA |
| OTU6476 | + | NA | NA | NA |
| OTU10208 | NA | NA | NA | NA |
| OTU15788 | + | NA | NA | NA |
| OTU17721 | NA | NA | NA | NA |
| OTU18100 | NA | NA | NA | NA |
| OTU14256 | NA | NA | NA | NA |
| OTU7660 | - | + | NA | NA |
| OTU13956 | NA | + | NA | NA |
| OTU15589 | + | NA | NA | NA |
| OTU8025 | NA | NA | - | NA |
| OTU9425 | + | NA | NA | NA |
| OTU2525 | NA | NA | NA | NA |
| OTU8313 | NA | NA | - | NA |
| OTU3691 | NA | NA | + | NA |
| OTU9878 | NA | NA | NA | NA |
| OTU8549 | NA | NA | NA | NA |
| OTU18517 | NA | NA | NA | NA |
| OTU8229 | NA | NA | NA | NA |
| OTU18715 | NA | NA | NA | NA |
| OTU5734 | NA | NA | NA | NA |
| OTU227 | NA | NA | NA | NA |
| OTU9814 | NA | NA | NA | NA |
| OTU7719 | NA | NA | NA | NA |
| OTU4413 | + | NA | NA | NA |
| OTU3841 | NA | NA | NA | NA |
| OTU3087 | NA | NA | - | + |
| OTU1828 | NA | NA | NA | NA |
| OTU3729 | NA | NA | NA | NA |
| OTU14252 | NA | NA | NA | NA |
| OTU13095 | NA | - | NA | NA |
| OTU14334 | NA | NA | NA | NA |
| OTU7853 | NA | NA | - | + |
| OTU13862 | NA | NA | NA | NA |
| OTU3902 | NA | NA | NA | - |
| OTU8961 | + | NA | NA | NA |
| OTU14688 | + | NA | NA | NA |
| OTU7967 | NA | NA | NA | NA |
| OTU8642 | NA | NA | + | - |
| OTU6220 | NA | NA | - | NA |
| OTU14567 | + | - | NA | NA |
| OTU10397 | NA | - | NA | NA |
| OTU1148 | + | - | NA | NA |
| OTU5867 | NA | NA | NA | NA |
| OTU15476 | NA | - | NA | + |
| OTU17274 | NA | NA | NA | NA |
| OTU12584 | NA | NA | + | NA |
| OTU16266 | + | NA | - | NA |
| OTU395 | NA | - | NA | + |
| OTU8601 | + | NA | NA | NA |
| OTU8099 | NA | NA | NA | NA |
| OTU1467 | NA | NA | + | NA |
| OTU18941 | + | - | - | NA |
| OTU4277 | NA | NA | NA | - |
| OTU19118 | NA | NA | NA | NA |
| OTU13804 | NA | NA | NA | NA |
| OTU1334 | + | NA | NA | - |
| OTU2080 | NA | NA | + | - |
| OTU17514 | NA | NA | NA | NA |
| OTU14559 | NA | NA | NA | - |
| OTU8040 | NA | NA | NA | NA |
| OTU12785 | NA | NA | + | NA |
| OTU3092 | NA | NA | NA | NA |
| OTU3998 | + | NA | NA | NA |
| OTU5851 | NA | NA | NA | NA |
| OTU13650 | NA | NA | NA | NA |
| OTU12016 | NA | NA | NA | NA |
| OTU1574 | NA | NA | - | NA |
| OTU7465 | + | NA | NA | NA |
| OTU1621 | + | NA | NA | NA |
| OTU3618 | NA | NA | NA | NA |
| OTU18400 | NA | NA | NA | NA |
| OTU15572 | + | NA | NA | NA |
| OTU11664 | + | NA | NA | NA |
| OTU10027 | NA | NA | NA | NA |
| OTU5664 | NA | NA | NA | NA |
| OTU17676 | NA | NA | NA | NA |
| OTU11197 | NA | NA | NA | NA |
| OTU17006 | NA | NA | NA | NA |
| OTU5763 | NA | NA | NA | NA |
| OTU12464 | NA | NA | + | NA |
| OTU18666 | NA | NA | + | NA |
| OTU4609 | NA | NA | + | NA |
| OTU9573 | NA | + | NA | NA |
| OTU1722 | - | NA | NA | NA |
| OTU1808 | NA | NA | NA | NA |
| OTU13572 | NA | NA | NA | NA |
| OTU16353 | NA | NA | NA | NA |
| OTU6331 | NA | NA | NA | NA |
| OTU6232 | NA | + | NA | - |
| OTU1553 | NA | NA | NA | NA |
| OTU675 | NA | - | NA | NA |
| OTU15104 | NA | NA | NA | NA |
| OTU381 | + | NA | NA | NA |
| OTU12547 | NA | NA | NA | NA |
| OTU3677 | NA | NA | NA | NA |
| OTU11655 | + | NA | - | NA |
| OTU973 | + | NA | NA | NA |
| OTU2467 | + | - | NA | NA |
| OTU13451 | NA | NA | NA | NA |
| OTU8521 | NA | NA | NA | NA |
| OTU12574 | NA | NA | NA | + |
| OTU14062 | + | NA | NA | NA |
| OTU19147 | + | NA | NA | NA |
| OTU5264 | NA | NA | NA | NA |
| OTU10937 | NA | NA | NA | - |
| OTU7738 | NA | NA | NA | NA |
| OTU9742 | NA | NA | + | NA |
| OTU14493 | NA | NA | NA | NA |
| OTU14468 | - | + | NA | NA |
| OTU7618 | NA | NA | NA | NA |
| OTU18342 | NA | NA | NA | + |
| OTU8969 | NA | NA | NA | NA |
| OTU16328 | NA | + | NA | NA |
| OTU3893 | NA | - | NA | + |
| OTU9219 | + | NA | NA | NA |
| OTU9608 | NA | NA | NA | NA |
| OTU1965 | NA | NA | NA | NA |
| OTU4729 | NA | NA | NA | - |
| OTU4101 | NA | + | NA | NA |
| OTU5745 | NA | NA | NA | + |
| OTU17554 | NA | NA | NA | NA |
| OTU13747 | + | - | NA | NA |
| OTU14853 | NA | NA | NA | NA |
| OTU337 | NA | NA | NA | NA |
| OTU18045 | NA | NA | + | NA |
| OTU13973 | NA | NA | NA | + |
| OTU13562 | + | NA | - | NA |
| OTU15887 | NA | NA | NA | + |
| OTU1470 | NA | NA | NA | NA |
| OTU7832 | + | NA | NA | NA |
| OTU1646 | NA | NA | + | NA |
| OTU326 | NA | NA | NA | NA |
| OTU10716 | + | NA | - | NA |
| OTU11738 | NA | NA | NA | NA |
| OTU12641 | + | - | + | NA |
| OTU1848 | NA | NA | - | NA |
| OTU17649 | NA | NA | NA | NA |
| OTU7682 | NA | NA | NA | NA |
| OTU6548 | NA | NA | NA | NA |
| OTU17244 | NA | NA | NA | NA |
| OTU6029 | NA | NA | - | + |
| OTU14636 | NA | NA | NA | NA |
| OTU9558 | NA | NA | NA | NA |
| OTU648 | + | - | NA | NA |
| OTU18942 | NA | NA | NA | NA |
| OTU3336 | NA | NA | NA | NA |
| OTU3125 | + | NA | NA | NA |
| OTU15436 | NA | NA | NA | NA |
| OTU17765 | NA | + | NA | NA |
| OTU8375 | NA | - | + | NA |
| OTU6411 | NA | NA | NA | NA |
| OTU1901 | NA | NA | NA | NA |
| OTU10764 | NA | NA | NA | NA |
| OTU18603 | NA | NA | + | NA |
| OTU8445 | NA | NA | NA | NA |
| OTU1126 | NA | NA | NA | - |
| OTU5629 | NA | NA | NA | NA |
| OTU15350 | NA | - | NA | + |
| OTU17614 | NA | NA | NA | NA |
| OTU10045 | NA | NA | NA | NA |
| OTU14311 | NA | NA | NA | NA |
| OTU2146 | NA | NA | NA | NA |
| OTU15356 | - | NA | NA | NA |
| OTU14471 | NA | - | NA | NA |
| OTU1743 | + | NA | NA | NA |
| OTU1197 | + | - | NA | NA |
| OTU7881 | NA | NA | NA | NA |
| OTU1045 | + | NA | NA | NA |
| OTU4005 | NA | NA | + | NA |
| OTU2338 | NA | NA | NA | NA |
| OTU10003 | NA | NA | NA | NA |
| OTU5718 | NA | NA | NA | NA |
| OTU13346 | + | NA | NA | - |
| OTU5715 | + | NA | NA | - |
| OTU14211 | NA | NA | NA | - |
| OTU13184 | NA | NA | NA | NA |
| OTU8092 | NA | NA | NA | NA |
| OTU8251 | NA | NA | NA | - |
| OTU14868 | + | - | NA | NA |
| OTU10446 | NA | NA | + | NA |
| OTU5237 | NA | NA | NA | + |
| OTU2746 | + | - | NA | NA |
| OTU11617 | NA | NA | NA | NA |
| OTU15095 | NA | NA | NA | NA |
| OTU6051 | NA | NA | NA | NA |
| OTU14800 | NA | NA | NA | NA |
| OTU1229 | NA | NA | NA | NA |
| OTU16783 | + | - | NA | NA |
| OTU14874 | NA | NA | NA | NA |
| OTU1258 | NA | NA | - | NA |
| OTU8051 | NA | NA | NA | - |
| OTU4866 | NA | NA | NA | NA |
| OTU10437 | NA | NA | NA | NA |
| OTU1135 | NA | NA | NA | NA |
| OTU1798 | NA | NA | + | - |
| OTU7848 | NA | NA | NA | NA |
| OTU7354 | + | NA | NA | NA |
| OTU8308 | NA | NA | NA | NA |
| OTU7522 | NA | + | NA | NA |
| OTU14529 | NA | NA | + | NA |
| OTU13614 | NA | - | + | NA |
| OTU3116 | + | - | NA | NA |
| OTU18097 | NA | NA | - | NA |
| OTU1956 | NA | NA | NA | NA |
| OTU14367 | NA | NA | NA | NA |
| OTU10605 | NA | NA | NA | NA |
| OTU18609 | NA | NA | NA | NA |
| OTU15217 | NA | NA | - | + |
| OTU16135 | NA | NA | NA | + |
| OTU1059 | NA | NA | NA | NA |
| OTU14226 | NA | NA | - | NA |
| OTU6374 | NA | NA | NA | NA |
| OTU7737 | NA | NA | NA | NA |
| OTU8967 | NA | NA | NA | NA |
| OTU15136 | NA | NA | NA | NA |
| OTU340 | + | - | NA | NA |
| OTU9299 | + | - | NA | NA |
| OTU8661 | NA | NA | NA | NA |
| OTU1031 | NA | NA | NA | NA |
| OTU1701 | NA | - | NA | NA |
| OTU10787 | NA | NA | NA | NA |
| OTU11467 | NA | NA | NA | NA |
| OTU8516 | NA | NA | NA | NA |
| OTU5328 | + | NA | NA | - |
| OTU13563 | NA | NA | NA | NA |
| OTU7363 | + | NA | NA | NA |
| OTU2959 | + | NA | NA | NA |
| OTU13816 | NA | NA | NA | NA |
| OTU4455 | + | NA | NA | NA |
| OTU7039 | NA | NA | NA | NA |
| OTU14346 | NA | NA | NA | NA |
| OTU10343 | NA | NA | NA | NA |
| OTU12064 | NA | NA | + | NA |
| OTU5523 | NA | NA | NA | NA |
| OTU3688 | + | - | NA | NA |
| OTU7883 | NA | NA | NA | NA |
| OTU7501 | NA | NA | NA | NA |
| OTU11898 | NA | NA | + | NA |
| OTU15269 | NA | NA | NA | NA |
| OTU8928 | NA | NA | NA | NA |
| OTU5784 | + | NA | NA | NA |
| OTU13812 | NA | NA | NA | - |
| OTU18337 | NA | NA | NA | NA |
| OTU14502 | NA | - | NA | NA |
| OTU1925 | NA | + | NA | NA |
| OTU422 | NA | NA | NA | NA |
| OTU4805 | NA | NA | NA | NA |
| OTU1379 | NA | - | NA | NA |
| OTU2763 | NA | NA | NA | + |
| OTU4560 | - | NA | NA | NA |
| OTU5793 | NA | NA | NA | NA |
| OTU11386 | NA | - | NA | + |
| OTU1793 | NA | NA | NA | NA |
| OTU11492 | + | - | NA | NA |
| OTU10795 | - | NA | NA | NA |
| OTU10211 | NA | NA | NA | NA |
| OTU7076 | NA | NA | NA | - |
| OTU13176 | NA | - | + | NA |
| OTU15777 | NA | NA | NA | + |
| OTU11485 | NA | - | NA | + |
| OTU9061 | NA | + | - | NA |
| OTU12568 | + | NA | NA | NA |
| OTU5822 | + | NA | NA | NA |
| OTU9214 | + | NA | NA | NA |
| OTU3218 | NA | NA | NA | NA |
| OTU9327 | + | NA | NA | NA |
| OTU12004 | NA | NA | NA | NA |
| OTU18592 | + | NA | NA | NA |
| OTU18011 | NA | NA | - | + |
| OTU14456 | NA | NA | NA | NA |
| OTU8339 | + | NA | NA | NA |
| OTU9980 | NA | NA | NA | + |
| OTU6965 | NA | NA | NA | NA |
| OTU10547 | NA | NA | NA | NA |
| OTU9692 | NA | NA | NA | NA |
| OTU2684 | NA | - | NA | NA |
| OTU1338 | + | NA | NA | NA |
| OTU2063 | NA | NA | NA | NA |
| OTU11825 | NA | + | NA | NA |
| OTU6843 | NA | NA | + | NA |
| OTU921 | NA | - | NA | NA |
| OTU8073 | NA | NA | NA | NA |
| OTU19063 | NA | + | NA | NA |
| OTU3162 | + | NA | NA | NA |
| OTU11136 | + | NA | NA | NA |
| OTU15074 | NA | NA | NA | NA |
| OTU4547 | NA | NA | + | - |
| OTU9195 | NA | NA | NA | NA |
| OTU17005 | NA | - | NA | NA |
| OTU1837 | NA | NA | NA | NA |
| OTU17846 | + | NA | NA | NA |
| OTU154 | + | NA | NA | NA |
| OTU18122 | NA | - | NA | NA |
| OTU5089 | NA | NA | NA | - |
| OTU18549 | NA | NA | NA | NA |
| OTU7838 | NA | - | NA | NA |
| OTU15493 | NA | NA | NA | + |
| OTU4896 | NA | NA | NA | NA |
| OTU1437 | NA | NA | NA | NA |
| OTU3170 | NA | NA | NA | NA |
| OTU17661 | NA | + | - | + |
| OTU18465 | NA | NA | NA | NA |
| OTU19059 | + | - | NA | NA |
| OTU4930 | NA | NA | + | NA |
| OTU1502 | + | NA | NA | - |
| OTU18487 | NA | NA | NA | NA |
| OTU16109 | NA | NA | NA | NA |
| OTU18333 | NA | NA | NA | NA |
| OTU5800 | NA | NA | NA | NA |
| OTU16383 | NA | - | NA | NA |
| OTU10558 | NA | - | NA | NA |
| OTU14242 | NA | NA | NA | NA |
| OTU7242 | NA | NA | NA | - |
| OTU17535 | NA | NA | NA | + |
| OTU13286 | + | NA | NA | NA |
| OTU1279 | NA | NA | - | NA |
| OTU1768 | NA | NA | NA | NA |
| OTU19073 | NA | - | NA | NA |
| OTU1101 | NA | NA | NA | NA |
| OTU5668 | NA | NA | NA | NA |
| OTU12680 | NA | NA | NA | NA |
| OTU7517 | NA | NA | NA | NA |
| OTU640 | NA | NA | NA | NA |
| OTU16964 | NA | NA | - | + |
| OTU16997 | NA | NA | NA | NA |
| OTU12473 | NA | NA | NA | NA |
| OTU17008 | NA | NA | NA | + |
| OTU8973 | NA | NA | NA | NA |
| OTU9171 | NA | NA | NA | NA |
| OTU2154 | NA | NA | - | + |
| OTU7150 | + | NA | NA | NA |
| OTU18175 | NA | NA | - | NA |
| OTU4947 | NA | NA | NA | - |
| OTU7395 | NA | NA | NA | NA |
| OTU772 | NA | NA | NA | NA |
| OTU11602 | NA | - | + | NA |
| OTU14815 | NA | NA | NA | NA |
| OTU10190 | NA | NA | NA | NA |
| OTU11036 | NA | NA | NA | NA |
| OTU11977 | - | NA | NA | NA |
| OTU5197 | NA | + | NA | NA |
| OTU1622 | + | - | NA | NA |
| OTU3103 | NA | NA | NA | NA |
| OTU1109 | NA | NA | NA | NA |
| OTU10284 | NA | NA | NA | NA |
| OTU9630 | NA | NA | NA | NA |
| OTU41 | NA | NA | NA | NA |
| OTU3909 | NA | NA | NA | NA |
| OTU4353 | NA | NA | NA | NA |
| OTU6970 | NA | NA | NA | NA |
| OTU5823 | NA | NA | NA | NA |
| OTU5305 | NA | NA | NA | NA |
| OTU1522 | NA | NA | NA | NA |
| OTU17279 | NA | NA | NA | NA |
| OTU7648 | NA | NA | NA | NA |
| OTU1400 | + | NA | NA | NA |
| OTU1548 | NA | NA | NA | NA |
| OTU13999 | NA | NA | + | NA |
| OTU3291 | NA | NA | NA | NA |
| OTU6673 | NA | NA | + | NA |
| OTU1491 | NA | NA | NA | NA |
| OTU11417 | NA | NA | NA | NA |
| OTU8004 | + | NA | NA | NA |
| OTU11700 | NA | NA | NA | NA |
| OTU4770 | NA | NA | NA | - |
| OTU7276 | NA | NA | NA | - |
| OTU16300 | + | NA | NA | NA |
| OTU15399 | + | NA | NA | NA |
| OTU18527 | NA | NA | - | NA |
| OTU2822 | NA | - | NA | + |
| OTU14967 | NA | NA | NA | NA |
| OTU6755 | NA | NA | NA | NA |
| OTU10406 | NA | NA | NA | NA |
| OTU3966 | NA | NA | NA | NA |
| OTU11207 | NA | + | NA | NA |
| OTU7595 | NA | NA | NA | NA |
| OTU13823 | NA | NA | NA | NA |
| OTU18293 | NA | NA | NA | NA |
| OTU16557 | NA | NA | NA | NA |
| OTU15548 | + | NA | NA | NA |
| OTU10254 | NA | NA | NA | + |
| OTU6282 | NA | NA | NA | NA |
| OTU8986 | NA | NA | NA | NA |
| OTU16597 | NA | NA | NA | NA |
| OTU6618 | NA | - | NA | NA |
| OTU18691 | + | - | NA | NA |
| OTU13681 | NA | NA | NA | NA |
| OTU18149 | NA | NA | NA | NA |
| OTU18472 | NA | - | NA | NA |
| OTU7455 | NA | NA | NA | NA |
| OTU6790 | NA | NA | NA | NA |
| OTU9758 | NA | NA | + | NA |
| OTU13336 | NA | NA | + | - |
| OTU3857 | NA | NA | NA | NA |
| OTU9350 | + | NA | NA | NA |
| OTU7974 | NA | + | NA | - |
| OTU16700 | + | NA | NA | NA |
| OTU8931 | + | NA | - | NA |
| OTU3994 | NA | NA | NA | NA |
| OTU6977 | NA | NA | NA | + |
| OTU1192 | NA | NA | NA | NA |
| OTU5901 | + | NA | - | NA |
| OTU1687 | + | NA | NA | NA |
| OTU14666 | NA | NA | NA | NA |
| OTU404 | NA | NA | NA | + |
| OTU9960 | - | NA | NA | NA |
| OTU14011 | NA | - | NA | NA |
| OTU1268 | NA | - | NA | NA |
| OTU6004 | NA | NA | NA | NA |
| OTU4448 | - | NA | NA | NA |
| OTU14220 | + | NA | NA | NA |
| OTU15386 | NA | NA | NA | NA |
| OTU7888 | NA | NA | NA | NA |
| OTU14949 | + | - | NA | NA |
| OTU7644 | NA | NA | NA | NA |
| OTU8686 | NA | NA | NA | NA |
| OTU10979 | NA | NA | NA | NA |
| OTU9679 | NA | NA | + | NA |
| OTU2495 | + | NA | NA | NA |
| OTU3946 | NA | NA | NA | NA |
| OTU10858 | + | NA | - | NA |
| OTU5807 | NA | NA | + | NA |
| OTU17606 | NA | NA | - | + |
| OTU13410 | + | NA | NA | - |
| OTU4980 | NA | NA | NA | NA |
| OTU16158 | NA | NA | - | NA |
| OTU15017 | NA | NA | NA | NA |
| OTU2953 | NA | NA | + | NA |
| OTU18580 | + | - | NA | NA |
| OTU1845 | NA | NA | NA | NA |
| OTU13015 | NA | NA | NA | NA |
| OTU14976 | NA | NA | + | NA |
| OTU9946 | NA | NA | NA | NA |
| OTU18725 | NA | NA | NA | NA |
| OTU1525 | + | NA | NA | NA |
| OTU7466 | NA | NA | - | NA |
| OTU8314 | NA | NA | NA | NA |
| OTU17472 | NA | NA | + | NA |
| OTU5818 | NA | NA | NA | NA |
| OTU6389 | NA | - | NA | NA |
| OTU10552 | NA | NA | + | NA |
| OTU594 | + | NA | NA | NA |
| OTU8032 | NA | NA | NA | NA |
| OTU7334 | NA | NA | NA | NA |
| OTU3493 | NA | NA | NA | NA |
| OTU3658 | NA | NA | NA | NA |
| OTU7977 | NA | NA | - | + |
| OTU18489 | NA | NA | NA | NA |
| OTU18710 | + | NA | NA | NA |
| OTU17488 | NA | - | NA | + |
| OTU18160 | NA | NA | NA | NA |
| OTU420 | NA | NA | - | + |
| OTU6288 | NA | NA | NA | NA |
| OTU1084 | NA | NA | NA | NA |
| OTU9127 | + | NA | NA | NA |
| OTU7265 | NA | NA | NA | NA |
| OTU11926 | NA | NA | NA | + |
| OTU7456 | + | NA | NA | NA |
| OTU14441 | NA | NA | NA | NA |
| OTU14033 | - | NA | + | NA |
| OTU120 | NA | NA | NA | NA |
| OTU13578 | NA | NA | NA | NA |
| OTU15077 | NA | NA | NA | NA |
| OTU3527 | + | NA | NA | NA |
| OTU14557 | NA | - | + | NA |
| OTU17757 | NA | NA | NA | NA |
| OTU1082 | NA | NA | NA | NA |
| OTU6418 | NA | NA | NA | NA |
| OTU13012 | NA | NA | NA | NA |
| OTU2743 | NA | NA | NA | NA |
| OTU7729 | NA | NA | NA | - |
| OTU7817 | + | NA | NA | NA |
| OTU9285 | NA | NA | NA | NA |
| OTU4944 | NA | + | NA | NA |
| OTU7340 | + | NA | NA | NA |
| OTU9428 | NA | - | NA | NA |
| OTU9349 | NA | NA | NA | NA |
| OTU1773 | NA | NA | NA | NA |
| OTU13262 | NA | NA | NA | - |
| OTU13696 | + | NA | NA | NA |
| OTU5278 | - | + | NA | NA |
| OTU3712 | NA | NA | NA | - |
| OTU4494 | NA | NA | - | NA |
| OTU10160 | + | NA | NA | NA |
| OTU8031 | NA | NA | NA | NA |
| OTU1271 | NA | - | NA | + |
| OTU8263 | + | NA | NA | NA |
| OTU8904 | + | NA | NA | NA |
| OTU1623 | NA | + | NA | NA |
| OTU17145 | NA | NA | - | NA |
| OTU6042 | NA | NA | NA | NA |
| OTU8328 | + | NA | NA | NA |
| OTU10221 | NA | NA | + | NA |
| OTU7117 | NA | NA | NA | NA |
| OTU1580 | + | NA | - | NA |
| OTU93 | NA | NA | NA | NA |
| OTU9066 | - | NA | NA | NA |
| OTU14395 | NA | NA | NA | NA |
| OTU2320 | + | - | NA | NA |
| OTU10128 | NA | NA | NA | NA |
| OTU15374 | NA | NA | NA | NA |
| OTU2062 | NA | NA | NA | NA |
| OTU8294 | NA | NA | NA | NA |
| OTU14511 | NA | NA | + | NA |
| OTU5783 | NA | NA | NA | NA |
| OTU3807 | NA | - | NA | + |
| OTU4028 | NA | NA | NA | + |
| OTU5014 | NA | NA | NA | NA |
| OTU11998 | NA | NA | - | NA |
| OTU13717 | NA | NA | NA | NA |
| OTU18387 | NA | NA | - | NA |
| OTU15653 | NA | - | NA | + |
| OTU2123 | NA | NA | NA | - |
| OTU4914 | NA | NA | NA | NA |
| OTU16510 | NA | NA | NA | - |
| OTU1750 | + | NA | - | NA |
| OTU670 | NA | NA | NA | NA |
| OTU14177 | NA | NA | NA | NA |
| OTU3936 | NA | NA | NA | NA |
| OTU16975 | NA | - | NA | + |
| OTU4922 | NA | NA | NA | NA |
| OTU19084 | NA | + | NA | NA |
| OTU13415 | NA | + | NA | NA |
| OTU5507 | NA | + | NA | NA |
| OTU8397 | NA | NA | - | NA |
| OTU8010 | NA | NA | NA | + |
| OTU5133 | NA | NA | NA | NA |
| OTU234 | NA | - | NA | NA |
| OTU17297 | NA | - | NA | + |
| OTU1433 | NA | NA | NA | NA |
| OTU17986 | NA | NA | - | + |
| OTU15733 | + | NA | NA | NA |
| OTU5 | NA | NA | NA | NA |
| OTU3625 | NA | NA | NA | NA |
| OTU4443 | + | NA | NA | NA |
| OTU9986 | NA | NA | NA | + |
| OTU1472 | NA | NA | - | + |
| OTU883 | + | - | NA | NA |
| OTU1352 | NA | NA | NA | NA |
| OTU968 | NA | NA | NA | NA |
| OTU100 | + | NA | - | NA |
| OTU1393 | NA | NA | NA | NA |
| OTU3393 | + | NA | - | NA |
| OTU18426 | NA | NA | - | NA |
| OTU5999 | NA | NA | - | NA |
| OTU9321 | NA | NA | NA | NA |
| OTU11868 | NA | NA | NA | NA |
| OTU14897 | NA | NA | NA | - |
| OTU5290 | NA | NA | + | NA |
| OTU13590 | NA | NA | NA | NA |
| OTU8135 | + | NA | - | NA |
| OTU14682 | NA | NA | + | - |
| OTU2098 | NA | NA | NA | NA |
| OTU1843 | NA | NA | NA | NA |
| OTU13521 | NA | - | NA | NA |
| OTU12180 | NA | NA | NA | NA |
| OTU2048 | NA | NA | NA | - |
| OTU1833 | NA | NA | NA | NA |
| OTU10135 | + | NA | NA | NA |
| OTU15684 | NA | NA | + | NA |
| OTU6500 | - | NA | NA | NA |
| OTU3861 | NA | NA | - | NA |
| OTU1865 | NA | NA | NA | NA |
| OTU1689 | NA | NA | NA | NA |
| OTU9308 | + | NA | NA | NA |
| OTU17003 | NA | NA | - | + |
| OTU13597 | NA | NA | NA | NA |
| OTU10470 | NA | NA | NA | - |
| OTU1755 | NA | NA | NA | NA |
| OTU18344 | NA | NA | NA | NA |
| OTU7689 | NA | NA | NA | NA |
| OTU18203 | NA | NA | - | + |
| OTU9026 | NA | NA | NA | NA |
| OTU12970 | NA | NA | NA | NA |
| OTU6761 | NA | NA | NA | NA |
| OTU5861 | NA | NA | NA | NA |
| OTU1170 | NA | NA | NA | NA |
| OTU964 | NA | NA | NA | NA |
| OTU13038 | NA | NA | NA | NA |
| OTU4033 | NA | NA | NA | + |
| OTU14229 | NA | NA | NA | + |
| OTU13301 | NA | + | NA | NA |
| OTU5714 | + | NA | NA | NA |
| OTU17619 | NA | NA | NA | NA |
| OTU18745 | + | - | NA | NA |
| OTU16809 | - | NA | + | NA |
| OTU9169 | NA | NA | NA | + |
| OTU17958 | NA | NA | NA | NA |
| OTU14470 | - | NA | + | NA |
| OTU7564 | + | NA | - | NA |
| OTU5704 | NA | NA | NA | NA |
| OTU14519 | - | NA | + | NA |
| OTU18385 | - | + | NA | NA |
| OTU19159 | + | NA | NA | NA |
| OTU1080 | NA | NA | - | NA |
| OTU11991 | NA | NA | + | - |
| OTU14494 | NA | + | NA | NA |
| OTU3666 | + | NA | NA | - |
| OTU16829 | NA | NA | NA | NA |
| OTU14793 | NA | NA | NA | NA |
| OTU17278 | NA | NA | NA | NA |
| OTU9178 | - | NA | NA | NA |
| OTU7958 | NA | NA | NA | NA |
| OTU16541 | NA | NA | NA | NA |
| OTU18181 | NA | NA | - | NA |
| OTU148 | + | NA | - | + |
| OTU9620 | NA | NA | NA | + |
| OTU11060 | NA | + | NA | NA |
| OTU13294 | + | NA | - | NA |
| OTU2662 | NA | NA | NA | NA |
| OTU11857 | NA | NA | NA | - |
| OTU13492 | NA | NA | NA | NA |
| OTU6599 | NA | NA | NA | NA |
| OTU4236 | + | NA | NA | NA |
| OTU5138 | NA | NA | NA | NA |
| OTU4757 | NA | NA | NA | + |
| OTU590 | + | NA | NA | NA |
| OTU2006 | NA | NA | NA | NA |
| OTU698 | NA | NA | NA | NA |
| OTU13468 | + | NA | NA | NA |
| OTU9743 | NA | NA | NA | NA |
| OTU14804 | NA | NA | NA | - |
| OTU5207 | NA | NA | + | NA |
| OTU16333 | NA | NA | NA | NA |
| OTU5227 | NA | NA | NA | - |
| OTU5304 | NA | NA | NA | + |
| OTU9145 | NA | NA | NA | NA |
| OTU1934 | NA | - | NA | + |
| OTU11600 | + | NA | - | NA |
| OTU2440 | NA | NA | NA | NA |
| OTU15648 | NA | NA | + | NA |
| OTU8080 | + | NA | NA | NA |
| OTU1990 | NA | NA | NA | - |
| OTU15483 | - | NA | NA | NA |
| OTU7527 | - | NA | NA | NA |
| OTU423 | NA | NA | NA | - |
| OTU6025 | - | NA | NA | NA |
| OTU13651 | NA | NA | NA | NA |
| OTU5109 | NA | NA | NA | NA |
| OTU3720 | NA | NA | + | NA |
| OTU18220 | + | NA | NA | NA |
| OTU3672 | NA | NA | - | + |
| OTU9197 | + | NA | NA | NA |
| OTU6135 | NA | NA | NA | NA |
| OTU6902 | NA | + | NA | NA |
| OTU1579 | NA | + | NA | NA |
| OTU11625 | NA | NA | + | - |
| OTU13512 | NA | NA | NA | NA |
| OTU5301 | NA | NA | NA | NA |
| OTU13632 | - | + | NA | NA |
| OTU7830 | NA | NA | NA | NA |
| OTU13705 | NA | NA | NA | NA |
| OTU3654 | + | NA | NA | NA |
| OTU17346 | NA | NA | NA | NA |
| OTU16808 | NA | NA | NA | NA |
| OTU1851 | NA | + | NA | NA |
| OTU7783 | + | NA | NA | NA |
| OTU9854 | NA | NA | NA | NA |
| OTU14702 | NA | NA | NA | NA |
| OTU378 | + | NA | NA | NA |
| OTU3655 | NA | NA | NA | NA |
| OTU7429 | NA | NA | NA | NA |
| OTU2135 | NA | NA | NA | NA |
| OTU14508 | NA | NA | NA | NA |
| OTU5956 | NA | NA | NA | - |
| OTU13365 | NA | NA | NA | NA |
| OTU5319 | NA | NA | + | NA |
| OTU12606 | NA | + | NA | - |
| OTU14132 | NA | NA | NA | NA |
| OTU14304 | NA | NA | NA | NA |
| OTU10534 | NA | NA | NA | NA |
| OTU1732 | NA | - | NA | NA |
| OTU13257 | + | NA | NA | NA |
| OTU13249 | NA | NA | NA | NA |
| OTU5951 | NA | + | NA | NA |
| OTU3696 | NA | NA | - | + |
| OTU2122 | NA | - | + | NA |
| OTU1373 | + | NA | NA | NA |
| OTU307 | NA | NA | NA | NA |
| OTU18746 | + | NA | NA | NA |
| OTU8983 | NA | NA | NA | NA |
| OTU15970 | NA | NA | NA | NA |
| OTU6878 | NA | NA | NA | NA |
| OTU1473 | NA | NA | NA | NA |
| OTU14124 | NA | + | NA | - |
| OTU17033 | NA | NA | NA | NA |
| OTU17271 | NA | NA | NA | NA |
| OTU13900 | NA | NA | NA | NA |
| OTU5438 | NA | + | NA | NA |
| OTU10674 | NA | NA | NA | NA |
| OTU44 | NA | NA | NA | NA |
| OTU10419 | NA | NA | + | NA |
| OTU18548 | NA | NA | NA | NA |
| OTU13426 | NA | NA | NA | NA |
| OTU9372 | + | NA | NA | NA |
| OTU993 | + | NA | NA | NA |
| OTU13232 | NA | - | + | NA |
| OTU6666 | - | NA | NA | + |
| OTU10192 | NA | NA | NA | NA |
| OTU18927 | NA | NA | NA | NA |
| OTU1977 | NA | NA | NA | NA |
| OTU3459 | NA | NA | NA | - |
| OTU13530 | NA | NA | NA | NA |
| OTU2310 | NA | NA | NA | NA |
| OTU7978 | NA | NA | NA | NA |
| OTU1780 | NA | NA | NA | NA |
| OTU641 | NA | NA | NA | NA |
| OTU5087 | NA | NA | + | - |
| OTU14462 | NA | NA | + | - |
| OTU13808 | + | NA | NA | NA |
| OTU5085 | + | NA | NA | NA |
| OTU9898 | NA | NA | NA | NA |
| OTU15440 | NA | - | NA | NA |
| OTU11119 | NA | NA | NA | NA |
| OTU16137 | NA | NA | NA | + |
| OTU8085 | NA | NA | + | NA |
| OTU2607 | + | NA | - | NA |
| OTU5330 | NA | NA | NA | NA |
| OTU18509 | NA | NA | NA | NA |
| OTU14069 | NA | - | NA | NA |
| OTU3030 | NA | NA | NA | NA |
| OTU1731 | NA | NA | NA | NA |
| OTU17422 | NA | NA | NA | NA |
| OTU5322 | NA | NA | - | NA |
| OTU3821 | NA | NA | NA | NA |
| OTU945 | NA | NA | NA | - |
| OTU1612 | + | NA | NA | NA |
| OTU14159 | NA | + | NA | NA |
| OTU11869 | NA | NA | NA | NA |
| OTU14549 | NA | NA | + | NA |
| OTU4408 | NA | NA | - | NA |
| OTU2981 | NA | NA | NA | NA |
| OTU8265 | NA | NA | NA | NA |
| OTU1856 | NA | NA | NA | NA |
| OTU4128 | NA | + | - | NA |
| OTU13224 | NA | NA | + | NA |
| OTU13721 | NA | NA | NA | NA |
| OTU16799 | + | - | NA | NA |
| OTU15997 | + | - | NA | + |
| OTU5332 | NA | NA | + | NA |
| OTU5769 | NA | NA | NA | NA |
| OTU6535 | NA | NA | NA | NA |
| OTU5383 | NA | NA | NA | NA |
| OTU15124 | NA | NA | NA | NA |
| OTU18670 | NA | NA | NA | NA |
| OTU14251 | NA | NA | NA | NA |
| OTU3767 | NA | NA | NA | NA |
| OTU14700 | NA | + | NA | NA |
| OTU10366 | + | - | NA | NA |
| OTU14243 | NA | NA | + | NA |
| OTU13624 | NA | NA | NA | NA |
| OTU9259 | NA | NA | NA | NA |
| OTU5187 | NA | NA | NA | NA |
| OTU15547 | NA | NA | NA | NA |
| OTU13851 | + | NA | NA | NA |
| OTU8183 | + | NA | NA | NA |
| OTU9126 | NA | NA | NA | NA |
| OTU1503 | NA | - | NA | NA |
| OTU18448 | NA | NA | NA | NA |
| OTU1505 | NA | NA | NA | NA |
| OTU3911 | NA | NA | + | - |
| OTU3912 | NA | NA | NA | + |
| OTU12399 | NA | NA | NA | + |
| OTU2032 | NA | NA | NA | NA |
| OTU18088 | NA | NA | NA | NA |
| OTU14325 | + | NA | NA | NA |
| OTU6208 | NA | NA | NA | NA |
| OTU4135 | NA | + | NA | - |
| OTU8138 | + | NA | NA | NA |
| OTU4713 | NA | NA | NA | - |
| OTU5804 | NA | NA | - | + |
| OTU16617 | NA | NA | NA | NA |
| OTU6310 | NA | NA | - | NA |
| OTU17659 | NA | NA | NA | NA |
| OTU8215 | + | NA | NA | NA |
| OTU4715 | NA | NA | - | NA |
| OTU6728 | NA | NA | NA | NA |
| OTU7379 | NA | NA | NA | NA |
| OTU5437 | NA | NA | NA | + |
| OTU9149 | NA | NA | NA | NA |
| OTU6017 | + | NA | NA | - |
| OTU5202 | NA | NA | - | NA |
| OTU603 | + | NA | NA | NA |
| OTU12036 | NA | - | + | NA |
| OTU7357 | + | NA | NA | NA |
| OTU601 | + | NA | - | NA |
| OTU3863 | NA | NA | NA | NA |
| OTU3100 | NA | NA | NA | NA |
| OTU6304 | NA | + | - | NA |
| OTU15280 | NA | NA | - | + |
| OTU8618 | NA | NA | NA | + |
| OTU6913 | NA | NA | NA | NA |
| OTU11081 | NA | NA | NA | NA |
| OTU9206 | NA | NA | NA | NA |
| OTU8949 | NA | NA | NA | NA |
| OTU5917 | NA | NA | NA | NA |
| OTU8865 | + | NA | NA | NA |
| OTU4593 | - | NA | NA | NA |
| OTU11308 | NA | NA | NA | NA |
| OTU6485 | NA | NA | NA | NA |
| OTU13979 | + | NA | NA | NA |
| OTU8405 | NA | NA | NA | NA |
| OTU14964 | NA | NA | NA | NA |
| OTU8109 | NA | NA | - | NA |
| OTU7782 | + | NA | NA | NA |
| OTU8433 | + | NA | NA | NA |
| OTU6796 | NA | - | + | NA |
| OTU1350 | NA | NA | - | NA |
| OTU4917 | NA | NA | NA | NA |
| OTU12860 | NA | NA | NA | - |
| OTU5239 | NA | NA | NA | NA |
| OTU7328 | NA | NA | NA | NA |
| OTU7988 | NA | NA | NA | NA |
| OTU125 | NA | - | NA | NA |
| OTU1378 | NA | + | NA | NA |
| OTU10051 | NA | NA | NA | NA |
| OTU11635 | NA | NA | + | NA |
| OTU2553 | NA | NA | NA | NA |
| OTU5386 | NA | NA | NA | - |
| OTU10544 | NA | NA | NA | NA |
| OTU4655 | NA | + | NA | NA |
| OTU15565 | NA | NA | NA | NA |
| OTU1633 | NA | NA | + | NA |
| OTU13319 | NA | NA | NA | NA |
| OTU2007 | NA | - | NA | + |
| OTU9150 | NA | NA | NA | NA |
| OTU7819 | NA | NA | NA | NA |
| OTU4548 | NA | NA | NA | NA |
| OTU8384 | NA | NA | NA | - |
| OTU1668 | NA | NA | NA | - |
| OTU8868 | NA | NA | NA | NA |
| OTU10181 | NA | NA | NA | + |
| OTU1641 | NA | NA | NA | NA |
| OTU10945 | NA | NA | NA | NA |
| OTU5246 | NA | NA | - | + |
| OTU4827 | NA | NA | NA | NA |
| OTU8946 | NA | NA | + | NA |
| OTU1696 | NA | NA | + | - |
| OTU6385 | NA | NA | NA | + |
| OTU14527 | NA | NA | NA | NA |
| OTU18071 | NA | NA | NA | + |
| OTU16801 | NA | NA | NA | NA |
| OTU8952 | NA | NA | NA | NA |
| OTU5483 | - | + | NA | NA |
| OTU293 | NA | NA | NA | NA |
| OTU5399 | NA | NA | NA | NA |
| OTU6365 | NA | NA | NA | NA |
| OTU184 | NA | NA | NA | - |
| OTU9975 | - | NA | NA | NA |
| OTU7942 | NA | NA | NA | NA |
| OTU18355 | NA | NA | NA | NA |
| OTU18988 | NA | NA | NA | NA |
| OTU5249 | NA | NA | NA | NA |
| OTU4415 | NA | NA | NA | NA |
| OTU14858 | NA | NA | + | NA |
| OTU10715 | NA | NA | NA | NA |
| OTU18646 | + | - | NA | NA |
| OTU18926 | NA | NA | NA | NA |
| OTU8195 | NA | NA | NA | NA |
| OTU13447 | NA | NA | NA | NA |
| OTU13889 | + | NA | NA | NA |
| OTU11367 | NA | NA | NA | NA |
| OTU1246 | NA | NA | NA | NA |
| OTU17075 | NA | NA | NA | NA |
| OTU5879 | NA | NA | + | - |
| OTU17608 | NA | NA | NA | NA |
| OTU2935 | NA | - | + | NA |
| OTU2078 | NA | - | NA | + |
| OTU12607 | NA | NA | NA | NA |
| OTU5127 | NA | NA | NA | NA |
| OTU8429 | NA | NA | NA | NA |
| OTU577 | NA | NA | - | NA |
| OTU5987 | NA | NA | NA | NA |
| OTU10951 | NA | NA | NA | NA |
| OTU5008 | NA | NA | NA | NA |
| OTU17418 | NA | NA | NA | NA |
| OTU4878 | NA | + | NA | NA |
| OTU16457 | NA | NA | NA | NA |
| OTU9241 | NA | NA | NA | NA |
| OTU5896 | NA | NA | NA | NA |
| OTU17487 | NA | NA | NA | NA |
| OTU2359 | + | - | NA | NA |
| OTU3742 | NA | NA | NA | NA |
| OTU746 | NA | NA | NA | NA |
| OTU17461 | NA | NA | NA | NA |
| OTU966 | NA | NA | - | NA |
| OTU1309 | NA | NA | NA | NA |
| OTU428 | NA | NA | NA | NA |
| OTU18121 | NA | NA | + | - |
| OTU14215 | NA | NA | NA | NA |
| OTU2602 | + | NA | NA | NA |
| OTU6356 | NA | - | NA | NA |
| OTU4539 | NA | NA | NA | NA |
| OTU10694 | NA | NA | + | NA |
| OTU13477 | NA | NA | NA | NA |
| OTU9747 | NA | NA | NA | NA |
| OTU13937 | NA | NA | NA | NA |
| OTU10607 | NA | + | - | NA |
| OTU4354 | NA | NA | NA | NA |
| OTU3489 | NA | NA | NA | NA |
| OTU10584 | NA | + | NA | NA |
| OTU10269 | NA | NA | NA | NA |
| OTU15446 | NA | NA | - | + |
| OTU10369 | NA | NA | NA | NA |
| OTU3734 | NA | NA | NA | NA |
| OTU7653 | + | NA | - | NA |
| OTU553 | NA | NA | NA | + |
| OTU3831 | NA | NA | NA | NA |
| OTU5913 | NA | NA | - | + |
| OTU1327 | NA | NA | NA | NA |
| OTU3275 | NA | NA | - | NA |
| OTU5479 | NA | NA | NA | NA |
| OTU5032 | NA | NA | NA | NA |
| OTU10344 | NA | NA | NA | NA |
| OTU14761 | NA | NA | NA | NA |
| OTU633 | + | NA | NA | NA |
| OTU11440 | NA | NA | + | NA |
| OTU10099 | NA | NA | + | - |
| OTU8545 | + | NA | NA | NA |
| OTU7415 | NA | NA | NA | NA |
| OTU1526 | + | - | NA | NA |
| OTU1922 | NA | NA | NA | NA |
| OTU9269 | + | NA | NA | NA |
| OTU13026 | + | NA | NA | NA |
| OTU166 | + | NA | NA | NA |
| OTU798 | + | NA | NA | NA |
| OTU12557 | NA | NA | NA | NA |
| OTU17723 | + | - | NA | NA |
| OTU18311 | NA | NA | - | + |
| OTU6906 | NA | NA | NA | NA |
| OTU18253 | NA | NA | NA | NA |
| OTU8953 | NA | NA | NA | NA |
| OTU6424 | NA | NA | NA | NA |
| OTU9389 | NA | NA | NA | + |
| OTU1629 | NA | NA | NA | NA |
| OTU18616 | NA | NA | NA | NA |
| OTU2076 | NA | NA | NA | NA |
| OTU5306 | NA | NA | NA | NA |
| OTU16780 | NA | NA | NA | NA |
| OTU11945 | NA | NA | NA | NA |
| OTU7804 | + | NA | NA | NA |
| OTU15631 | + | NA | NA | NA |
| OTU15469 | NA | NA | NA | NA |
| OTU11311 | NA | NA | + | NA |
| OTU3904 | NA | NA | NA | NA |
| OTU14903 | + | - | NA | NA |
| OTU5123 | NA | NA | NA | NA |
| OTU9201 | NA | NA | NA | NA |
| OTU6047 | NA | NA | NA | NA |
| OTU12597 | NA | NA | NA | NA |
| OTU3553 | - | NA | NA | NA |
| OTU5716 | NA | NA | NA | NA |
| OTU6907 | NA | NA | - | NA |
| OTU5191 | NA | NA | NA | NA |
| OTU14586 | NA | NA | NA | - |
| OTU991 | NA | NA | NA | NA |
| OTU9383 | NA | + | NA | - |
| OTU158 | NA | NA | NA | NA |
| OTU1052 | NA | NA | + | - |
| OTU14475 | NA | NA | NA | + |
| OTU1072 | NA | NA | NA | NA |
| OTU17423 | - | NA | + | NA |
| OTU17541 | NA | NA | NA | NA |
| OTU10079 | NA | NA | NA | NA |
| OTU13957 | + | - | + | - |
| OTU14545 | NA | NA | NA | - |
| OTU591 | NA | NA | NA | NA |
| OTU466 | NA | NA | NA | NA |
| OTU11695 | NA | NA | NA | NA |
| OTU3048 | NA | NA | + | NA |
| OTU11586 | + | NA | NA | NA |
| OTU19138 | + | NA | - | NA |
| OTU6257 | NA | NA | - | NA |
| OTU14286 | NA | NA | NA | - |
| OTU3832 | NA | NA | NA | NA |
| OTU7612 | NA | NA | + | NA |
| OTU9068 | NA | + | NA | NA |
| OTU18973 | NA | + | NA | NA |
| OTU11020 | - | NA | NA | NA |
| OTU2750 | NA | NA | NA | NA |
| OTU9500 | + | NA | NA | NA |
| OTU2532 | NA | - | + | NA |
| OTU892 | + | NA | NA | - |
| OTU16644 | NA | NA | NA | NA |
| OTU12154 | NA | NA | NA | - |
| OTU18540 | NA | NA | NA | NA |
| OTU1958 | NA | NA | NA | NA |
| OTU1860 | NA | NA | NA | NA |
| OTU7929 | NA | NA | + | NA |
| OTU5253 | + | NA | NA | NA |
| OTU16303 | NA | NA | NA | NA |
| OTU9354 | - | + | NA | NA |
| OTU15431 | NA | NA | + | NA |
| OTU7490 | NA | NA | NA | NA |
| OTU13779 | NA | NA | NA | NA |
| OTU14219 | - | + | NA | NA |
| OTU6522 | NA | NA | NA | NA |
| OTU14280 | NA | NA | + | NA |
| OTU12718 | NA | NA | NA | NA |
| OTU10958 | NA | NA | NA | NA |
| OTU6686 | NA | NA | NA | NA |
| OTU14985 | NA | NA | NA | NA |
| OTU14326 | NA | NA | NA | NA |
| OTU1459 | NA | NA | NA | NA |
| OTU11387 | NA | NA | NA | NA |
| OTU8190 | + | NA | NA | NA |
| OTU13610 | NA | NA | NA | NA |
| OTU7818 | NA | NA | - | NA |
| OTU6316 | NA | NA | NA | - |
| OTU6263 | NA | NA | NA | + |
| OTU2850 | NA | - | NA | + |
| OTU17344 | + | NA | NA | NA |
| OTU11003 | NA | NA | NA | NA |
| OTU5677 | + | NA | NA | NA |
| OTU15827 | NA | NA | NA | NA |
| OTU9297 | NA | NA | NA | + |
| OTU8188 | + | NA | NA | NA |
| OTU6958 | NA | NA | - | NA |
| OTU19040 | NA | NA | NA | NA |
| OTU1986 | NA | NA | NA | NA |
| OTU3613 | NA | - | NA | NA |
| OTU5836 | NA | NA | NA | NA |
| OTU17906 | - | NA | NA | NA |
| OTU1231 | NA | NA | - | + |
| OTU6577 | NA | NA | NA | NA |
| OTU17841 | NA | NA | NA | NA |
| OTU11499 | NA | NA | NA | NA |
| OTU7960 | NA | NA | + | NA |
| OTU13454 | NA | NA | NA | NA |
| OTU4316 | NA | NA | NA | NA |
| OTU5719 | + | NA | NA | NA |
| OTU12917 | NA | NA | NA | NA |
| OTU9043 | + | NA | NA | NA |
| OTU2022 | NA | NA | NA | NA |
| OTU14728 | NA | NA | NA | - |
| OTU6691 | NA | - | + | NA |
| OTU6284 | NA | NA | NA | + |
| OTU15259 | NA | NA | NA | NA |
| OTU17976 | NA | NA | NA | NA |
| OTU5806 | NA | NA | NA | + |
| OTU14381 | NA | - | NA | + |
| OTU17607 | NA | NA | + | - |
| OTU17768 | NA | NA | + | NA |
| OTU15273 | NA | NA | NA | NA |
| OTU4745 | - | NA | NA | NA |
| OTU10350 | NA | NA | NA | NA |
| OTU7536 | NA | NA | NA | NA |
| OTU13297 | NA | NA | NA | NA |
| OTU13434 | NA | NA | NA | NA |
| OTU2425 | NA | NA | NA | NA |
| OTU18346 | NA | NA | NA | NA |
| OTU17894 | NA | - | NA | + |
| OTU11040 | NA | NA | NA | NA |
| OTU13925 | NA | NA | NA | NA |
| OTU14167 | NA | NA | NA | NA |
| OTU513 | NA | NA | NA | + |
| OTU10224 | NA | NA | NA | NA |
| OTU5925 | NA | NA | NA | - |
| OTU13409 | NA | NA | NA | NA |
| OTU10535 | NA | NA | NA | NA |
| OTU10186 | NA | - | NA | + |
| OTU11935 | NA | NA | NA | NA |
| OTU4743 | NA | NA | NA | NA |
| OTU233 | NA | NA | NA | NA |
| OTU6390 | NA | NA | + | - |
| OTU13635 | NA | NA | + | - |
| OTU2013 | NA | NA | NA | NA |
| OTU4589 | NA | NA | NA | NA |
| OTU11733 | + | NA | NA | NA |
| OTU12124 | + | NA | - | NA |
| OTU15594 | NA | NA | + | - |
| OTU13626 | NA | NA | + | - |
| OTU17288 | NA | NA | NA | + |
| OTU7635 | NA | NA | NA | NA |
| OTU6801 | NA | NA | NA | NA |
| OTU7047 | + | NA | NA | NA |
| OTU339 | NA | NA | NA | NA |
| OTU18040 | NA | NA | NA | - |
| OTU6757 | NA | NA | - | + |
| OTU1081 | + | NA | NA | NA |
| OTU5740 | NA | NA | NA | + |
| OTU14601 | NA | NA | + | - |
| OTU17993 | NA | NA | NA | NA |
| OTU490 | + | - | NA | NA |
| OTU8036 | + | NA | NA | NA |
| OTU13662 | NA | NA | NA | NA |
| OTU10178 | NA | NA | NA | NA |
| OTU310 | NA | NA | NA | NA |
| OTU18642 | + | NA | NA | NA |
| OTU5486 | NA | + | NA | NA |
| OTU318 | + | NA | NA | NA |
| OTU13739 | + | NA | NA | NA |
| OTU7904 | NA | NA | NA | + |
| OTU6092 | NA | NA | NA | NA |
| OTU8860 | NA | + | NA | NA |
| OTU324 | NA | NA | NA | NA |
| OTU13429 | NA | NA | + | NA |
| OTU8197 | NA | NA | NA | NA |
| OTU14839 | NA | + | NA | NA |
| OTU9832 | NA | NA | NA | NA |
| OTU10053 | NA | NA | NA | NA |
| OTU1056 | NA | NA | NA | NA |
| OTU4283 | + | NA | NA | NA |
| OTU4043 | NA | NA | NA | NA |
| OTU9332 | NA | NA | NA | NA |
| OTU5075 | + | NA | NA | - |
| OTU18154 | + | NA | NA | NA |
| OTU16489 | NA | NA | NA | NA |
| OTU5694 | NA | + | - | NA |
| OTU6475 | + | NA | NA | NA |
| OTU8108 | + | NA | NA | NA |
| OTU6879 | + | NA | NA | NA |
| OTU5753 | NA | - | NA | NA |
| OTU4484 | + | - | NA | NA |
| OTU5940 | + | NA | NA | NA |
| OTU16312 | NA | NA | NA | NA |
| OTU6943 | NA | NA | NA | NA |
| OTU11994 | NA | + | NA | NA |
| OTU16658 | + | NA | NA | NA |
| OTU4933 | NA | NA | + | - |
| OTU1602 | + | NA | NA | NA |
| OTU996 | + | NA | NA | NA |
| OTU18350 | NA | + | NA | NA |
| OTU16285 | + | NA | NA | NA |
| OTU16468 | NA | NA | NA | NA |
| OTU11824 | NA | NA | NA | NA |
| OTU15101 | NA | NA | NA | + |
| OTU6442 | NA | NA | NA | NA |
| OTU8134 | NA | NA | NA | NA |
| OTU5902 | NA | NA | NA | - |
| OTU1969 | NA | - | NA | NA |
| OTU1015 | + | NA | NA | - |
| OTU1861 | NA | NA | NA | NA |
| OTU1358 | + | NA | NA | NA |
| OTU18025 | NA | NA | NA | + |
| OTU18329 | NA | NA | + | NA |
| OTU6722 | + | NA | NA | NA |
| OTU4804 | NA | NA | + | NA |
| OTU17146 | NA | NA | NA | + |
| OTU4583 | NA | NA | NA | NA |
| OTU13203 | NA | NA | + | NA |
| OTU953 | NA | NA | NA | NA |
| OTU6767 | NA | NA | NA | NA |
| OTU4306 | NA | NA | NA | NA |
| OTU3500 | + | NA | NA | NA |
| OTU17273 | + | NA | - | NA |
| OTU14322 | + | NA | NA | - |
| OTU4478 | NA | NA | NA | NA |
| OTU1182 | NA | NA | NA | NA |
| OTU11442 | NA | - | NA | + |
| OTU16168 | NA | NA | NA | + |
| OTU18457 | NA | NA | NA | + |
| OTU5182 | NA | NA | NA | NA |
| OTU10503 | NA | NA | NA | NA |
| OTU4016 | NA | + | NA | NA |
| OTU18747 | + | - | NA | NA |
| OTU5628 | NA | NA | NA | NA |
| OTU9371 | + | NA | NA | NA |
| OTU9204 | + | NA | NA | NA |
| OTU1611 | NA | NA | NA | NA |
| OTU9147 | NA | NA | NA | NA |
| OTU13965 | + | NA | NA | NA |
| OTU7794 | + | NA | NA | NA |
| OTU1738 | NA | NA | NA | NA |
| OTU15829 | NA | NA | NA | NA |
| OTU7585 | NA | NA | NA | NA |
| OTU17610 | NA | NA | + | NA |
| OTU6209 | NA | NA | - | + |
| OTU14837 | NA | NA | NA | NA |
| OTU929 | NA | NA | NA | NA |
| OTU2427 | NA | NA | NA | NA |
| OTU11043 | NA | NA | NA | NA |
| OTU15443 | NA | NA | NA | NA |
| OTU2073 | + | NA | - | NA |
| OTU5229 | NA | NA | NA | NA |
| OTU579 | NA | NA | NA | NA |
| OTU5683 | NA | NA | + | - |
| OTU7414 | NA | NA | NA | NA |
| OTU7606 | NA | NA | NA | NA |
| OTU6580 | NA | NA | NA | - |
| OTU6619 | NA | NA | NA | NA |
| OTU4454 | NA | NA | NA | - |
| OTU1455 | + | NA | NA | NA |
| OTU5979 | NA | NA | NA | NA |
| OTU1871 | NA | NA | NA | NA |
| OTU2415 | + | NA | NA | NA |
| OTU2069 | NA | NA | NA | NA |
| OTU3735 | NA | NA | NA | NA |
| OTU18862 | NA | NA | NA | + |
| OTU6078 | + | NA | NA | NA |
| OTU15459 | NA | NA | NA | NA |
| OTU18109 | NA | NA | - | + |
| OTU11699 | NA | - | + | NA |
| OTU2052 | NA | NA | NA | NA |
| OTU5744 | NA | NA | NA | NA |
| OTU1770 | NA | NA | NA | NA |
| OTU5103 | NA | - | NA | NA |
| OTU7358 | + | NA | NA | NA |
| OTU9591 | NA | NA | NA | NA |
| OTU13996 | NA | NA | NA | - |
| OTU11692 | NA | NA | NA | NA |
| OTU1561 | + | NA | - | NA |
| OTU8991 | NA | NA | NA | NA |
| OTU13334 | NA | NA | NA | - |
| OTU17107 | NA | NA | NA | + |
| OTU18777 | NA | NA | NA | NA |
| OTU7577 | + | NA | NA | NA |
| OTU14941 | NA | NA | NA | NA |
| OTU5371 | NA | NA | NA | NA |
| OTU14234 | NA | NA | NA | NA |
| OTU3711 | NA | NA | NA | NA |
| OTU2285 | NA | NA | NA | NA |
| OTU14444 | NA | NA | NA | NA |
| OTU10270 | + | - | NA | NA |
| OTU10965 | NA | NA | NA | NA |
| OTU1749 | NA | NA | NA | NA |
| OTU14276 | NA | NA | + | NA |
| OTU4802 | NA | NA | NA | + |
| OTU6564 | NA | NA | + | NA |
| OTU6610 | NA | NA | NA | NA |
| OTU13627 | NA | NA | NA | NA |
| OTU9346 | NA | NA | NA | NA |
| OTU9915 | NA | - | NA | NA |
| OTU9156 | NA | + | - | NA |
| OTU14061 | NA | NA | NA | - |
| OTU13921 | + | NA | - | NA |
| OTU6353 | NA | NA | NA | NA |
| OTU2800 | + | NA | NA | NA |
| OTU5068 | NA | NA | NA | NA |
| OTU7519 | NA | NA | NA | NA |
| OTU4552 | NA | NA | NA | NA |
| OTU11194 | NA | NA | NA | NA |
| OTU2460 | NA | + | NA | NA |
| OTU12632 | NA | NA | NA | NA |
| OTU2055 | NA | NA | NA | NA |
| OTU13673 | NA | NA | NA | - |
| OTU2728 | NA | - | NA | NA |
| OTU8143 | NA | NA | + | NA |
| OTU7583 | NA | NA | - | NA |
| OTU5116 | NA | NA | - | NA |
| OTU15193 | NA | NA | NA | NA |
| OTU14353 | NA | NA | NA | NA |
| OTU4530 | - | + | NA | NA |
| OTU17259 | NA | - | NA | + |
| OTU15996 | NA | NA | NA | NA |
| OTU1117 | + | NA | NA | NA |
| OTU9057 | NA | NA | NA | NA |
| OTU8164 | NA | NA | NA | NA |
| OTU14846 | - | NA | + | NA |
| OTU1700 | NA | NA | NA | NA |
| OTU6735 | NA | NA | NA | - |
| OTU13619 | NA | NA | NA | NA |
| OTU15550 | + | NA | NA | NA |
| OTU16001 | NA | NA | - | NA |
| OTU6037 | NA | NA | NA | NA |
| OTU10722 | + | NA | NA | NA |
| OTU9807 | NA | NA | NA | NA |
| OTU4290 | + | NA | - | NA |
| OTU18396 | NA | NA | NA | NA |
| OTU10176 | NA | - | NA | NA |
| OTU10793 | NA | NA | NA | NA |
| OTU2827 | NA | NA | NA | NA |
| OTU2447 | + | NA | NA | NA |
| OTU13685 | NA | NA | + | NA |
| OTU5216 | NA | NA | NA | NA |
| OTU13901 | NA | NA | NA | NA |
| OTU5097 | NA | NA | NA | NA |
| OTU1679 | + | NA | - | NA |
| OTU5073 | NA | + | - | NA |
| OTU661 | NA | NA | - | NA |
| OTU5233 | NA | NA | NA | + |
| OTU3055 | NA | NA | NA | NA |
| OTU7943 | NA | NA | NA | - |
| OTU15813 | NA | - | NA | + |
| OTU10394 | NA | NA | NA | + |
| OTU12365 | NA | NA | NA | NA |
| OTU4727 | NA | NA | NA | NA |
| OTU5044 | NA | NA | NA | NA |
| OTU17517 | + | NA | NA | NA |
| OTU616 | NA | NA | NA | - |
| OTU11647 | NA | NA | NA | NA |
| OTU18226 | NA | NA | NA | NA |
| OTU4267 | NA | NA | NA | NA |
| OTU1043 | NA | NA | NA | - |
| OTU5600 | NA | NA | NA | NA |
| OTU13255 | NA | NA | NA | NA |
| OTU8449 | + | NA | - | NA |
| OTU4357 | NA | NA | NA | NA |
| OTU7494 | NA | NA | - | + |
| OTU7809 | + | NA | NA | NA |
| OTU7385 | NA | NA | NA | NA |
| OTU5323 | NA | NA | NA | NA |
| OTU13535 | NA | NA | + | - |
| OTU7752 | + | NA | NA | NA |
| OTU10152 | NA | NA | NA | - |
| OTU7897 | NA | NA | NA | NA |
| OTU31 | NA | NA | NA | NA |
| OTU5375 | NA | NA | - | NA |
| OTU7491 | NA | NA | NA | NA |
| OTU4280 | NA | NA | NA | + |
| OTU9294 | NA | NA | NA | NA |
| OTU5080 | NA | NA | NA | NA |
| OTU18138 | NA | NA | NA | + |
| OTU15451 | NA | NA | NA | NA |
| OTU15984 | NA | NA | NA | NA |
| OTU13182 | NA | NA | NA | NA |
| OTU2121 | NA | NA | NA | - |
| OTU6436 | NA | NA | NA | NA |
| OTU15060 | NA | - | NA | NA |
| OTU8016 | + | NA | NA | - |
| OTU17189 | NA | - | NA | + |
| OTU5947 | NA | NA | NA | NA |
| OTU14016 | NA | NA | NA | NA |
| OTU15886 | NA | NA | NA | NA |
| OTU5199 | NA | NA | NA | NA |
| OTU12322 | NA | NA | + | NA |
| OTU14081 | NA | NA | NA | NA |
| OTU10531 | NA | NA | NA | NA |
| OTU8696 | NA | NA | NA | NA |
| OTU15471 | NA | NA | NA | + |
| OTU7016 | NA | NA | NA | NA |
| OTU9947 | NA | NA | NA | NA |
| OTU15262 | NA | NA | + | - |
| OTU8070 | NA | NA | NA | NA |
| OTU11997 | + | NA | NA | NA |
| OTU18162 | NA | - | NA | NA |
| OTU1822 | NA | NA | NA | NA |
| OTU7229 | + | NA | NA | NA |
| OTU7984 | NA | NA | NA | NA |
| OTU17097 | NA | NA | NA | + |
| OTU17769 | NA | NA | NA | + |
| OTU18004 | NA | NA | NA | NA |
| OTU14864 | + | NA | NA | NA |
| OTU18904 | NA | NA | NA | NA |
| OTU3558 | NA | NA | NA | NA |
| OTU3128 | NA | NA | - | NA |
| OTU14208 | NA | - | + | NA |
| OTU2780 | NA | NA | NA | NA |
| OTU361 | + | NA | - | NA |
| OTU14390 | NA | NA | NA | NA |
| OTU8273 | + | NA | NA | NA |
| OTU14809 | NA | NA | NA | NA |
| OTU5580 | - | NA | NA | NA |
| OTU12452 | NA | NA | NA | NA |
| OTU4348 | NA | NA | NA | NA |
| OTU4785 | NA | NA | NA | NA |
| OTU7184 | NA | NA | NA | NA |
| OTU11622 | NA | NA | NA | NA |
| OTU6514 | NA | NA | NA | NA |
| OTU11966 | NA | NA | NA | NA |
| OTU1446 | NA | NA | NA | NA |
| OTU13527 | NA | NA | NA | NA |
| OTU2492 | NA | NA | NA | NA |
| OTU17223 | NA | NA | NA | NA |
| OTU17998 | NA | NA | NA | NA |
| OTU1047 | NA | NA | NA | NA |
| OTU14881 | + | NA | NA | NA |
| OTU6525 | NA | NA | NA | NA |
| OTU5670 | NA | NA | NA | NA |
| OTU18544 | NA | NA | NA | NA |
| OTU2797 | NA | + | NA | - |
| OTU2358 | NA | NA | NA | NA |
| OTU19113 | + | NA | NA | NA |
| OTU15097 | NA | NA | NA | NA |
| OTU2149 | NA | NA | NA | NA |
| OTU9030 | NA | NA | NA | NA |
| OTU984 | NA | - | NA | NA |
| OTU2716 | NA | NA | NA | NA |
| OTU15687 | + | NA | NA | NA |
| OTU2970 | NA | NA | + | NA |
| OTU8172 | NA | NA | NA | NA |
| OTU8298 | NA | NA | NA | NA |
| OTU6168 | NA | NA | + | NA |
| OTU11028 | NA | NA | NA | NA |
| OTU12688 | NA | NA | NA | - |
| OTU17202 | NA | NA | NA | NA |
| OTU4840 | NA | NA | NA | NA |
| OTU13473 | NA | NA | + | NA |
| OTU979 | + | NA | NA | NA |
| OTU2848 | NA | NA | NA | + |
| OTU169 | NA | NA | NA | NA |
| OTU14331 | NA | NA | NA | NA |
| OTU15328 | NA | NA | NA | + |
| OTU16871 | NA | NA | NA | + |
| OTU14597 | NA | NA | NA | - |
| OTU7715 | + | - | NA | NA |
| OTU9158 | NA | NA | NA | NA |
| OTU5926 | NA | NA | NA | NA |
| OTU11866 | NA | NA | NA | NA |
| OTU17447 | NA | NA | NA | NA |
| OTU1341 | NA | - | NA | + |
| OTU12528 | NA | - | NA | NA |
| OTU5639 | NA | NA | NA | NA |
| OTU2726 | NA | NA | NA | NA |
| OTU10074 | NA | NA | + | NA |
| OTU14453 | NA | NA | NA | NA |
| OTU11458 | NA | NA | NA | NA |
| OTU1187 | NA | - | + | NA |
| OTU15697 | + | NA | NA | NA |
| OTU5320 | NA | NA | NA | NA |
| OTU245 | + | NA | NA | NA |
| OTU793 | NA | NA | NA | NA |
| OTU17004 | NA | NA | NA | + |
| OTU942 | + | NA | NA | NA |
| OTU4746 | NA | + | NA | NA |
| OTU7567 | NA | NA | NA | + |
| OTU18522 | NA | NA | NA | NA |
| OTU3414 | + | - | NA | NA |
| OTU18124 | NA | NA | NA | NA |
| OTU1108 | + | NA | NA | NA |
| OTU8461 | NA | NA | NA | NA |
| OTU2498 | + | NA | NA | NA |
| OTU14349 | + | NA | NA | NA |
| OTU7208 | + | - | NA | NA |
| OTU3885 | NA | NA | NA | NA |
| OTU14244 | NA | NA | + | NA |
| OTU2449 | + | NA | NA | NA |
| OTU18205 | NA | NA | NA | NA |
| OTU8711 | NA | NA | NA | NA |
| OTU13536 | NA | NA | NA | NA |
| OTU9643 | NA | NA | NA | NA |
| OTU10294 | NA | NA | NA | NA |
| OTU7489 | NA | + | NA | NA |
| OTU13861 | NA | + | NA | NA |
| OTU2985 | NA | NA | NA | NA |
| OTU1924 | NA | NA | NA | + |
| OTU17011 | + | NA | - | NA |
| OTU5401 | NA | NA | NA | NA |
| OTU11963 | NA | NA | + | - |
| OTU14434 | NA | NA | NA | NA |
| OTU431 | NA | NA | NA | NA |
| OTU3135 | NA | NA | NA | NA |
| OTU6677 | NA | NA | NA | NA |
| OTU1996 | + | NA | NA | NA |
| OTU13656 | NA | NA | NA | NA |
| OTU13078 | NA | NA | NA | NA |
| OTU17886 | NA | NA | NA | NA |
| OTU11877 | NA | NA | NA | NA |
| OTU9902 | NA | - | + | NA |
| OTU15394 | NA | NA | - | NA |
| OTU2500 | NA | NA | NA | NA |
| OTU1866 | + | - | NA | NA |
| OTU1008 | + | NA | NA | NA |
| OTU9175 | NA | NA | NA | NA |
| OTU1769 | NA | NA | - | NA |
| OTU1632 | NA | NA | NA | NA |
| OTU2174 | NA | NA | NA | NA |
| OTU547 | NA | NA | NA | + |
| OTU9437 | + | - | NA | NA |
| OTU17431 | NA | NA | NA | NA |
| OTU18503 | + | NA | NA | NA |
| OTU4513 | NA | NA | NA | NA |
| OTU18724 | NA | NA | NA | NA |
| OTU2041 | NA | NA | NA | NA |
| OTU19062 | NA | - | NA | NA |
| OTU860 | NA | NA | NA | - |
| OTU3418 | NA | NA | NA | - |
| OTU367 | NA | NA | - | + |
| OTU14801 | NA | + | NA | NA |
| OTU4346 | NA | - | NA | NA |
| OTU14661 | NA | NA | NA | NA |
| OTU11537 | NA | NA | + | NA |
| OTU484 | + | NA | NA | NA |
| OTU12565 | NA | NA | NA | NA |
| OTU8410 | + | NA | - | NA |
| OTU18683 | + | - | NA | NA |
| OTU1912 | NA | NA | NA | + |
| OTU10705 | NA | NA | + | NA |
| OTU16291 | NA | - | NA | NA |
| OTU1514 | + | NA | NA | NA |
| OTU5877 | NA | NA | NA | NA |
| OTU6437 | + | NA | NA | NA |
| OTU1200 | + | NA | NA | NA |
| OTU8388 | NA | NA | NA | NA |
| OTU4166 | NA | + | NA | NA |
| OTU15424 | NA | NA | NA | NA |
| OTU17169 | NA | NA | NA | NA |
| OTU13832 | NA | NA | NA | NA |
| OTU2085 | NA | NA | NA | NA |
| OTU7214 | + | NA | - | NA |
| OTU17582 | NA | NA | NA | + |
| OTU6534 | NA | NA | NA | NA |
| OTU19157 | NA | NA | NA | NA |
| OTU3661 | NA | NA | NA | NA |
| OTU14843 | + | NA | NA | NA |
| OTU17753 | NA | NA | NA | NA |
| OTU3401 | NA | NA | NA | NA |
| OTU16035 | NA | NA | NA | NA |
| OTU1046 | NA | NA | - | NA |
| OTU13338 | NA | NA | NA | NA |
| OTU19087 | NA | NA | NA | NA |
| OTU13582 | NA | NA | NA | NA |
| OTU18294 | NA | + | NA | NA |
| OTU12751 | NA | NA | NA | - |
| OTU6759 | NA | - | NA | NA |
| OTU11932 | NA | NA | NA | NA |
| OTU524 | NA | NA | NA | + |
| OTU305 | + | NA | NA | NA |
| OTU16030 | NA | NA | NA | NA |
| OTU17018 | NA | - | NA | + |
| OTU8332 | + | NA | NA | NA |
| OTU1477 | NA | NA | NA | NA |
| OTU9926 | NA | NA | + | NA |
| OTU410 | + | NA | NA | NA |
| OTU14505 | NA | NA | NA | NA |
| OTU12471 | NA | NA | NA | NA |
| OTU18406 | + | - | NA | NA |
| OTU7685 | + | NA | NA | NA |
| OTU9795 | NA | NA | NA | NA |
| OTU3039 | NA | NA | NA | NA |
| OTU11838 | NA | NA | NA | NA |
| OTU9122 | NA | NA | NA | NA |
| OTU17858 | NA | NA | NA | NA |
| OTU7932 | NA | NA | NA | NA |
| OTU12888 | + | NA | NA | NA |
| OTU7025 | NA | NA | NA | NA |
| OTU7746 | NA | NA | NA | NA |
| OTU17021 | NA | NA | - | + |
| OTU18034 | NA | - | NA | NA |
| OTU1804 | NA | NA | NA | + |
| OTU1875 | NA | + | NA | NA |
| OTU18141 | NA | NA | NA | + |
| OTU8921 | NA | NA | NA | NA |
| OTU5834 | NA | NA | NA | NA |
| OTU6562 | NA | NA | NA | NA |
| OTU12749 | NA | NA | NA | NA |
| OTU11706 | NA | NA | NA | NA |
| OTU450 | NA | NA | NA | NA |
| OTU15495 | NA | NA | NA | NA |
| OTU1596 | NA | NA | + | NA |
| OTU15441 | NA | NA | NA | + |
| OTU6421 | NA | NA | - | NA |
| OTU11267 | NA | NA | + | - |
| OTU16129 | NA | - | NA | + |
| OTU4481 | + | NA | NA | NA |
| OTU10935 | NA | NA | NA | NA |
| OTU15393 | NA | NA | - | NA |
| OTU5210 | NA | NA | NA | NA |
| OTU6422 | NA | NA | NA | - |
| OTU982 | + | - | NA | NA |
| OTU977 | + | NA | - | NA |
| OTU2817 | NA | - | NA | + |
| OTU15083 | NA | - | NA | NA |
| OTU9384 | NA | NA | NA | NA |
| OTU6657 | NA | - | NA | NA |
| OTU10071 | NA | NA | NA | - |
| OTU7824 | NA | NA | NA | NA |
| OTU890 | + | - | NA | NA |
| OTU1923 | NA | NA | + | - |
| OTU5893 | + | NA | NA | NA |
| OTU2455 | NA | NA | NA | NA |
| OTU9060 | NA | NA | - | + |
| OTU9306 | NA | + | NA | NA |
| OTU5591 | NA | NA | NA | NA |
| OTU1289 | NA | NA | NA | NA |
| OTU5139 | NA | NA | NA | NA |
| OTU456 | NA | NA | NA | NA |
| OTU647 | + | - | NA | NA |
| OTU5257 | NA | NA | NA | NA |
| OTU1624 | NA | NA | NA | - |
| OTU8658 | NA | NA | NA | NA |
| OTU3437 | NA | NA | NA | NA |
| OTU14285 | NA | NA | NA | NA |
| OTU11034 | NA | - | + | NA |
| OTU6354 | NA | NA | NA | NA |
| OTU8042 | NA | NA | NA | NA |
| OTU6425 | NA | NA | NA | NA |
| OTU17859 | NA | - | NA | + |
| OTU11582 | NA | + | NA | NA |
| OTU18771 | NA | NA | NA | NA |
| OTU15553 | + | NA | - | NA |
| OTU365 | NA | NA | NA | NA |
| OTU16831 | NA | NA | NA | + |
| OTU7662 | NA | NA | - | + |
| OTU12807 | NA | NA | NA | NA |
| OTU11734 | NA | NA | NA | NA |
| OTU15850 | + | NA | NA | NA |
| OTU19099 | + | NA | - | NA |
| OTU5534 | NA | NA | NA | NA |
| OTU14612 | NA | NA | + | - |
| OTU13914 | NA | NA | NA | NA |
| OTU7400 | NA | NA | + | - |
| OTU11610 | NA | NA | - | NA |
| OTU4362 | NA | NA | NA | NA |
| OTU6979 | + | NA | - | NA |
| OTU13419 | + | - | + | NA |
| OTU8678 | NA | NA | NA | NA |
| OTU1049 | + | NA | NA | NA |
| OTU7710 | NA | NA | + | NA |
| OTU5666 | NA | NA | NA | NA |
| OTU12524 | NA | NA | NA | NA |
| OTU17823 | NA | NA | NA | NA |
| OTU9116 | NA | NA | NA | NA |
| OTU9215 | NA | NA | - | NA |
| OTU1647 | NA | - | NA | NA |
| OTU7069 | NA | NA | NA | NA |
| OTU1483 | + | NA | NA | NA |
| OTU18513 | NA | - | NA | NA |
| OTU17224 | NA | NA | NA | NA |
| OTU11279 | NA | NA | + | NA |
| OTU1763 | NA | NA | NA | NA |
| OTU5904 | NA | NA | NA | NA |
| OTU13944 | NA | NA | NA | NA |
| OTU4021 | NA | + | NA | NA |
| OTU13625 | NA | NA | NA | NA |
| OTU6031 | NA | NA | NA | NA |
| OTU6513 | NA | NA | NA | NA |
| OTU18156 | + | - | NA | NA |
| OTU8245 | + | NA | - | NA |
| OTU15455 | NA | NA | NA | NA |
| OTU17016 | NA | NA | NA | NA |
| OTU18504 | NA | NA | NA | NA |
| OTU5882 | NA | NA | + | NA |
| OTU3533 | NA | NA | NA | NA |
| OTU14489 | NA | NA | + | - |
| OTU9109 | NA | NA | NA | NA |
| OTU12333 | NA | NA | NA | NA |
| OTU5542 | NA | NA | NA | NA |
| OTU9038 | + | NA | NA | NA |
| OTU11883 | NA | NA | NA | NA |
| OTU7654 | NA | NA | NA | NA |
| OTU8770 | NA | NA | NA | NA |
| OTU744 | + | NA | NA | NA |
| OTU17241 | NA | NA | NA | NA |
| OTU16394 | NA | NA | NA | NA |
| OTU1412 | NA | NA | NA | NA |
| OTU6480 | NA | NA | NA | NA |
| OTU10926 | NA | NA | NA | NA |
| OTU8683 | NA | NA | NA | NA |
| OTU10645 | NA | NA | NA | - |
| OTU18276 | NA | NA | NA | NA |
| OTU653 | NA | NA | NA | NA |
| OTU19068 | + | - | NA | - |
| OTU2036 | NA | NA | NA | NA |
| OTU15289 | NA | NA | NA | NA |
| OTU5309 | NA | NA | NA | NA |
| OTU6896 | NA | NA | NA | NA |
| OTU14207 | + | - | NA | NA |
| OTU5255 | + | NA | NA | NA |
| OTU15167 | + | - | NA | NA |
| OTU18301 | NA | NA | NA | NA |
| OTU14345 | NA | NA | NA | NA |
| OTU1604 | NA | NA | NA | NA |
| OTU1806 | NA | - | NA | NA |
| OTU1916 | NA | NA | NA | NA |
| OTU15260 | NA | - | NA | NA |
| OTU9892 | NA | NA | NA | NA |
| OTU14014 | + | NA | - | NA |
| OTU18381 | NA | NA | NA | NA |
| OTU11047 | NA | - | + | NA |
| OTU8365 | + | NA | - | NA |
| OTU1413 | NA | - | NA | NA |
| OTU3550 | NA | NA | NA | NA |
| OTU674 | + | NA | - | NA |
| OTU16306 | NA | NA | NA | NA |
| OTU5557 | NA | NA | NA | NA |
| OTU8316 | NA | NA | - | NA |
| OTU6782 | NA | NA | + | - |
| OTU1915 | NA | NA | + | - |
| OTU281 | NA | NA | NA | NA |
| OTU8501 | NA | NA | + | - |
| OTU3865 | NA | NA | NA | NA |
| OTU13897 | + | NA | - | NA |
| OTU17167 | NA | NA | NA | + |
| OTU11423 | NA | NA | NA | NA |
| OTU16598 | NA | NA | NA | NA |
| OTU18651 | NA | NA | NA | NA |
| OTU8159 | NA | NA | NA | NA |
| OTU9641 | NA | NA | NA | NA |
| OTU15799 | NA | NA | NA | NA |
| OTU9610 | NA | NA | NA | NA |
| OTU13967 | + | NA | NA | NA |
| OTU357 | NA | NA | NA | - |
| OTU4769 | NA | NA | NA | NA |
| OTU5729 | NA | NA | + | NA |
| OTU17123 | + | - | NA | + |
| OTU8929 | NA | NA | NA | NA |
| OTU2016 | NA | NA | NA | - |
| OTU13464 | NA | NA | NA | NA |
| OTU1870 | NA | NA | NA | NA |
| OTU5550 | - | NA | NA | NA |
| OTU322 | NA | NA | NA | NA |
| OTU7680 | NA | NA | NA | NA |
| OTU13238 | NA | NA | NA | NA |
| OTU803 | NA | - | + | NA |
| OTU5946 | NA | NA | NA | NA |
| OTU12091 | NA | NA | NA | NA |
| OTU2045 | NA | + | NA | NA |
| OTU11850 | NA | NA | NA | NA |
| OTU1235 | NA | NA | NA | NA |
| OTU9228 | NA | NA | NA | - |
| OTU7949 | NA | NA | NA | NA |
| OTU13666 | NA | NA | + | - |
| OTU4958 | NA | NA | NA | NA |
| OTU6398 | NA | + | NA | NA |
| OTU3686 | + | - | NA | NA |
| OTU6730 | + | NA | NA | NA |
| OTU7159 | - | + | NA | NA |
| OTU5936 | NA | + | - | NA |
| OTU246 | NA | NA | NA | NA |
| OTU5504 | NA | NA | NA | - |
| OTU8750 | NA | NA | NA | NA |
| OTU12834 | NA | NA | NA | NA |
| OTU4201 | NA | NA | NA | NA |
| OTU3957 | NA | NA | NA | NA |
| OTU8447 | + | NA | NA | NA |
| OTU1411 | NA | NA | NA | NA |
| OTU14581 | NA | + | NA | - |
| OTU19082 | + | - | NA | NA |
| OTU5659 | NA | + | NA | NA |
| OTU631 | + | NA | - | + |
| OTU16388 | NA | - | NA | NA |
| OTU436 | NA | NA | NA | NA |
| OTU11992 | NA | NA | NA | - |
| OTU12611 | NA | NA | + | - |
| OTU13609 | NA | NA | NA | NA |
| OTU6671 | NA | NA | NA | NA |
| OTU7574 | NA | NA | NA | NA |
| OTU7805 | NA | NA | NA | NA |
| OTU15433 | NA | NA | - | NA |
| OTU19150 | + | NA | NA | NA |
| OTU11527 | NA | NA | + | NA |
| OTU12255 | NA | NA | NA | NA |
| OTU16467 | NA | NA | NA | NA |
| OTU4890 | NA | + | NA | NA |
| OTU7532 | + | NA | NA | NA |
| OTU1940 | + | NA | NA | NA |
| OTU3421 | NA | NA | NA | NA |
| OTU3159 | NA | NA | NA | NA |
| OTU5698 | NA | - | NA | NA |
| OTU14254 | NA | NA | NA | NA |
| OTU3706 | NA | NA | NA | NA |
| OTU17253 | NA | NA | NA | NA |
| OTU16830 | NA | NA | NA | NA |
| OTU2224 | + | NA | NA | NA |
| OTU5735 | NA | NA | NA | NA |
| OTU5054 | NA | NA | NA | NA |
| OTU4782 | NA | NA | NA | NA |
| OTU8041 | NA | NA | - | NA |
| OTU2381 | NA | NA | + | - |
| OTU6523 | NA | + | NA | NA |
| OTU9623 | NA | NA | NA | NA |
| OTU14770 | NA | NA | - | NA |
| OTU383 | NA | NA | - | NA |
| OTU17337 | NA | NA | NA | NA |
| OTU13216 | NA | NA | NA | NA |
| OTU18402 | NA | NA | NA | NA |
| OTU10525 | + | NA | NA | NA |
| OTU14694 | NA | NA | NA | NA |
| OTU18159 | NA | NA | NA | NA |
| OTU954 | + | NA | NA | NA |
| OTU2974 | NA | + | NA | NA |
| OTU9298 | NA | NA | - | NA |
| OTU12052 | NA | NA | + | NA |
| OTU7086 | NA | NA | NA | NA |
| OTU11948 | + | NA | NA | - |
| OTU10884 | + | NA | NA | NA |
| OTU18139 | NA | + | NA | NA |
| OTU4942 | NA | NA | NA | NA |
| OTU4726 | NA | + | - | NA |
| OTU15768 | + | NA | NA | NA |
| OTU18685 | NA | NA | NA | NA |
| OTU4081 | NA | NA | NA | NA |
| OTU3252 | NA | NA | NA | NA |
| OTU1009 | + | NA | NA | NA |
| OTU14073 | NA | NA | + | NA |
| OTU15619 | NA | NA | NA | + |
| OTU7998 | NA | NA | NA | NA |
| OTU6705 | NA | NA | - | NA |
| OTU5595 | NA | NA | NA | NA |
| OTU16628 | NA | NA | NA | NA |
| OTU18705 | + | NA | NA | NA |
| OTU16933 | NA | NA | NA | NA |
| OTU6463 | NA | NA | NA | NA |
| OTU12472 | NA | NA | NA | NA |
| OTU9353 | NA | NA | NA | NA |
| OTU1100 | + | NA | NA | NA |
| OTU3728 | NA | NA | NA | NA |
| OTU5933 | NA | NA | NA | NA |
| OTU10652 | NA | NA | NA | NA |
| OTU13310 | NA | NA | NA | NA |
| OTU2266 | NA | NA | NA | NA |
| OTU10104 | NA | NA | + | - |
| OTU6625 | NA | NA | NA | NA |
| OTU7810 | NA | NA | + | NA |
| OTU17785 | NA | NA | NA | - |
| OTU9369 | NA | - | NA | NA |
| OTU5387 | NA | NA | NA | - |
| OTU8175 | NA | NA | NA | NA |
| OTU5814 | NA | NA | NA | NA |
| OTU8218 | + | NA | NA | NA |
| OTU13676 | + | NA | NA | - |
| OTU8400 | NA | NA | - | NA |
| OTU1917 | NA | NA | NA | NA |
| OTU3663 | NA | NA | NA | NA |
| OTU4971 | NA | NA | NA | NA |
| OTU4521 | NA | NA | NA | NA |
| OTU14854 | NA | NA | NA | NA |
| OTU6152 | + | NA | NA | NA |
| OTU6785 | + | NA | NA | NA |
| OTU680 | + | NA | - | NA |
| OTU9130 | + | NA | NA | NA |
| OTU3319 | + | NA | - | + |
| OTU766 | NA | NA | NA | - |
| OTU952 | + | NA | NA | NA |
| OTU18729 | NA | NA | NA | NA |
| OTU9229 | NA | NA | NA | NA |
| OTU3381 | + | NA | NA | NA |
| OTU11420 | + | NA | NA | NA |
| OTU1551 | + | NA | NA | NA |
| OTU17124 | NA | NA | NA | NA |
| OTU13822 | NA | NA | NA | NA |
| OTU17177 | + | NA | NA | NA |
| OTU13052 | NA | NA | NA | NA |
| OTU17909 | NA | NA | NA | NA |
| OTU4754 | NA | NA | NA | NA |
| OTU3003 | NA | NA | NA | NA |
| OTU1474 | + | NA | NA | NA |
| OTU660 | NA | NA | NA | NA |
| OTU8836 | NA | NA | NA | NA |
| OTU9981 | NA | NA | NA | NA |
| OTU4359 | NA | NA | NA | NA |
| OTU7559 | NA | + | NA | NA |
| OTU14078 | NA | NA | NA | NA |
| OTU13993 | NA | NA | + | NA |
| OTU9202 | NA | NA | NA | NA |
| OTU7350 | + | NA | NA | NA |
| OTU4429 | NA | NA | NA | - |
| OTU9144 | NA | NA | NA | NA |
| OTU7113 | NA | - | NA | NA |
| OTU2203 | + | NA | - | NA |
| OTU3217 | + | NA | NA | NA |
| OTU13968 | NA | NA | NA | NA |
| OTU4032 | NA | NA | + | - |
| OTU5214 | NA | NA | NA | - |
| OTU14282 | NA | NA | NA | NA |
| OTU1857 | NA | NA | NA | NA |
| OTU6708 | + | NA | NA | NA |
| OTU11113 | NA | NA | NA | NA |
| OTU7156 | NA | NA | NA | NA |
| OTU4463 | NA | NA | NA | NA |
| OTU13102 | NA | NA | NA | NA |
| OTU414 | + | NA | NA | NA |
| OTU16166 | NA | - | NA | + |
| OTU8794 | NA | NA | NA | + |
| OTU13470 | + | NA | - | NA |
| OTU5105 | NA | NA | NA | NA |
| OTU6290 | NA | + | NA | NA |
| OTU7784 | NA | NA | NA | NA |
| OTU18997 | NA | NA | NA | + |
| OTU1745 | NA | NA | + | NA |
| OTU5267 | NA | NA | NA | NA |
| OTU5942 | NA | + | NA | NA |
| OTU3265 | NA | NA | NA | NA |
| OTU17473 | NA | NA | NA | NA |
| OTU3299 | + | NA | - | NA |
| OTU6001 | NA | NA | NA | NA |
| OTU8979 | + | NA | - | NA |
| OTU15099 | NA | NA | NA | NA |
| OTU1627 | + | NA | NA | NA |
| OTU17532 | NA | NA | NA | NA |
| OTU12683 | NA | + | NA | NA |
| OTU16912 | + | NA | NA | NA |
| OTU4633 | NA | NA | NA | NA |
| OTU6756 | NA | NA | NA | NA |
| OTU3690 | NA | NA | NA | NA |
| OTU11697 | NA | NA | NA | NA |
| OTU8675 | NA | NA | NA | NA |
| OTU13525 | NA | NA | NA | + |
| OTU5773 | + | NA | NA | NA |
| OTU4134 | + | NA | NA | NA |
| OTU1552 | NA | NA | NA | + |
| OTU1369 | NA | NA | - | NA |
| OTU2639 | + | NA | NA | NA |
| OTU9015 | NA | NA | - | + |
| OTU8043 | NA | NA | NA | NA |
| OTU9012 | NA | NA | NA | NA |
| OTU14944 | NA | NA | NA | - |
| OTU15339 | NA | NA | NA | NA |
| OTU6000 | NA | NA | NA | NA |
| OTU6016 | NA | NA | NA | NA |
| OTU5250 | NA | + | NA | NA |
| OTU18533 | + | - | NA | NA |
| OTU18204 | NA | NA | NA | NA |
| OTU4711 | NA | NA | + | NA |
| OTU13212 | NA | - | NA | NA |
| OTU13285 | NA | NA | NA | NA |
| OTU16689 | NA | NA | - | NA |
| OTU11116 | NA | NA | + | - |
| OTU345 | NA | NA | NA | NA |
| OTU13616 | + | NA | NA | NA |
| OTU1757 | NA | NA | NA | NA |
| OTU17691 | NA | NA | NA | + |
| OTU18525 | NA | NA | NA | NA |
| OTU11609 | NA | NA | NA | NA |
| OTU8443 | + | NA | NA | NA |
| OTU9857 | NA | NA | NA | NA |
| OTU14106 | + | - | NA | NA |
| OTU5707 | NA | NA | NA | NA |
| OTU11758 | NA | NA | NA | - |
| OTU18774 | NA | NA | NA | + |
| OTU6189 | NA | NA | NA | NA |
| OTU896 | + | NA | NA | NA |
| OTU7409 | NA | NA | NA | NA |
| OTU2813 | NA | NA | NA | NA |
| OTU6165 | NA | NA | NA | NA |
| OTU1349 | NA | NA | NA | NA |
| OTU17924 | NA | NA | NA | NA |
| OTU6660 | NA | NA | NA | NA |
| OTU4328 | + | NA | NA | NA |
| OTU11949 | NA | NA | + | NA |
| OTU3623 | NA | NA | NA | NA |
| OTU13493 | NA | NA | NA | - |
| OTU14437 | NA | NA | NA | NA |
| OTU6872 | NA | NA | NA | NA |
| OTU14206 | NA | NA | + | - |
| OTU13612 | + | NA | NA | NA |
| OTU4031 | NA | NA | NA | NA |
| OTU6601 | + | NA | NA | NA |
| OTU12748 | NA | NA | NA | NA |
| OTU8631 | NA | + | NA | NA |
| OTU10493 | NA | NA | + | - |
| OTU8559 | + | NA | NA | NA |
| OTU7514 | NA | NA | NA | NA |
| OTU14820 | NA | NA | NA | - |
| OTU19137 | NA | - | NA | NA |
| OTU17907 | NA | NA | NA | NA |
| OTU13322 | NA | NA | NA | NA |
| OTU10449 | NA | NA | NA | NA |
| OTU173 | NA | NA | NA | NA |
| OTU17249 | NA | NA | NA | NA |
| OTU7603 | + | NA | NA | NA |
| OTU3717 | NA | NA | NA | NA |
| OTU2151 | NA | NA | NA | NA |
| OTU7692 | NA | NA | NA | NA |
| OTU9707 | NA | NA | NA | NA |
| OTU8424 | NA | NA | NA | NA |
| OTU8284 | NA | NA | NA | NA |
| OTU13659 | + | NA | NA | NA |
| OTU12554 | NA | NA | NA | NA |
| OTU5853 | NA | NA | NA | NA |
| OTU7578 | + | NA | NA | NA |
| OTU11716 | NA | NA | NA | NA |
| OTU988 | NA | NA | NA | NA |
| OTU11796 | NA | NA | + | NA |
| OTU18498 | + | NA | NA | NA |
| OTU7356 | NA | NA | NA | NA |
| OTU14281 | NA | NA | NA | NA |
| OTU1440 | + | NA | NA | NA |
| OTU14034 | + | NA | NA | NA |
| OTU15726 | NA | NA | NA | NA |
| OTU12074 | + | - | NA | - |
| OTU5594 | NA | NA | + | NA |
| OTU4737 | NA | NA | + | NA |
| OTU6734 | + | - | - | NA |
| OTU12668 | NA | NA | NA | NA |
| OTU15612 | NA | NA | NA | + |
| OTU14072 | NA | NA | + | NA |
| OTU8650 | + | NA | NA | NA |
| OTU6493 | NA | NA | NA | NA |
| OTU8015 | NA | NA | NA | NA |
| OTU4464 | + | NA | NA | NA |
| OTU10038 | NA | NA | NA | NA |
| OTU1859 | NA | NA | NA | NA |
| OTU11263 | NA | NA | NA | NA |
| OTU14364 | NA | NA | NA | NA |
| OTU5696 | NA | + | NA | NA |
| OTU8303 | + | NA | NA | NA |
| OTU13915 | NA | NA | - | NA |
| OTU13770 | NA | NA | NA | NA |
| OTU3384 | NA | NA | NA | NA |
| OTU51 | NA | + | NA | NA |
| OTU5869 | NA | NA | NA | NA |
| OTU4538 | - | + | NA | NA |
| OTU6859 | NA | NA | NA | + |
| OTU8320 | + | - | NA | NA |
| OTU8056 | + | NA | - | NA |
| OTU18327 | NA | NA | NA | + |
| OTU19105 | + | NA | NA | NA |
| OTU7981 | + | NA | NA | NA |
| OTU2835 | NA | NA | NA | NA |
| OTU11603 | NA | NA | NA | NA |
| OTU17625 | + | - | - | NA |
| OTU9001 | NA | NA | NA | NA |
| OTU13566 | NA | NA | NA | NA |
| OTU17150 | NA | NA | NA | + |
| OTU14308 | NA | NA | NA | NA |
| OTU7397 | NA | NA | NA | NA |
| OTU12122 | NA | NA | + | NA |
| OTU4365 | NA | NA | NA | + |
| OTU16865 | NA | NA | + | NA |
| OTU2524 | + | NA | NA | NA |
| OTU7911 | NA | NA | NA | + |
| OTU5564 | NA | NA | NA | NA |
| OTU955 | + | NA | NA | NA |
| OTU13588 | NA | - | NA | NA |
| OTU5104 | NA | NA | NA | NA |
| OTU13893 | NA | - | NA | NA |
| OTU9600 | NA | NA | NA | NA |
| OTU9974 | NA | NA | NA | NA |
| OTU1388 | NA | NA | - | NA |
| OTU13880 | NA | NA | NA | NA |
| OTU10248 | NA | NA | - | NA |
| OTU5690 | NA | NA | NA | NA |
| OTU5048 | NA | NA | NA | NA |
| OTU8372 | NA | NA | NA | NA |
| OTU13063 | NA | NA | NA | + |
| OTU11076 | NA | NA | + | - |
| OTU8444 | + | - | NA | NA |
| OTU9356 | NA | NA | NA | NA |
| OTU13910 | NA | NA | + | NA |
| OTU13890 | NA | NA | + | NA |
| OTU5963 | NA | NA | NA | NA |
| OTU12150 | NA | NA | NA | NA |
| OTU2965 | NA | NA | NA | + |
| OTU12260 | NA | NA | NA | NA |
| OTU5810 | NA | NA | NA | NA |
| OTU3826 | NA | NA | NA | NA |
| OTU8805 | + | NA | NA | NA |
| OTU11570 | NA | NA | NA | - |
| OTU580 | + | NA | NA | NA |
| OTU6505 | NA | NA | - | + |
| OTU6254 | NA | NA | NA | NA |
| OTU12792 | NA | NA | NA | - |
| OTU532 | NA | NA | NA | NA |
| OTU10662 | NA | NA | NA | NA |
| OTU6749 | NA | NA | - | NA |
| OTU13538 | NA | NA | NA | - |
| OTU3371 | NA | NA | NA | NA |
| OTU1465 | + | NA | NA | NA |
| OTU76 | + | NA | NA | NA |
| OTU8470 | NA | + | NA | NA |
| OTU18217 | NA | NA | + | NA |
| OTU9706 | NA | NA | NA | NA |
| OTU2951 | + | NA | NA | NA |
| OTU4211 | NA | + | NA | NA |
| OTU19078 | + | NA | NA | NA |
| OTU17225 | NA | NA | NA | NA |
| OTU2809 | NA | - | NA | NA |
| OTU5648 | NA | NA | NA | NA |
| OTU14649 | NA | NA | NA | NA |
| OTU7906 | + | NA | NA | NA |
| OTU4059 | NA | NA | NA | NA |
| OTU4717 | NA | NA | NA | NA |
| OTU18803 | NA | NA | NA | NA |
| OTU5038 | NA | NA | NA | - |
| OTU7065 | NA | NA | - | NA |
| OTU4261 | + | NA | NA | NA |
| OTU7927 | NA | NA | NA | NA |
| OTU12492 | NA | NA | NA | NA |
| OTU1207 | + | NA | NA | NA |
| OTU10377 | NA | NA | NA | NA |
| OTU5781 | NA | + | NA | NA |
| OTU12298 | NA | NA | - | NA |
| OTU7733 | NA | NA | NA | NA |
| OTU1803 | NA | NA | NA | NA |
| OTU14807 | NA | NA | NA | NA |
| OTU10542 | NA | NA | NA | NA |
| OTU2637 | NA | NA | NA | NA |
| OTU1030 | NA | NA | NA | NA |
| OTU17968 | NA | NA | NA | NA |
| OTU5201 | NA | NA | NA | NA |
| OTU2021 | NA | NA | NA | + |
| OTU16548 | NA | NA | NA | NA |
| OTU5078 | + | NA | NA | NA |
| OTU11955 | NA | NA | NA | NA |
| OTU6773 | NA | NA | NA | + |
| OTU12936 | NA | NA | NA | NA |
| OTU477 | NA | NA | NA | NA |
| OTU16491 | NA | NA | NA | NA |
| OTU18202 | NA | NA | NA | + |
| OTU969 | + | NA | - | NA |
| OTU17257 | NA | NA | NA | NA |
| OTU17888 | NA | NA | NA | NA |
| OTU691 | + | NA | NA | NA |
| OTU14875 | NA | NA | NA | NA |
| OTU3665 | NA | NA | + | NA |
| OTU7923 | NA | NA | NA | NA |
| OTU963 | NA | NA | - | NA |
| OTU1549 | NA | + | NA | - |
| OTU97 | NA | NA | + | - |
| OTU7382 | NA | NA | NA | NA |
| OTU11588 | NA | NA | NA | NA |
| OTU392 | NA | NA | NA | NA |
| OTU1060 | NA | NA | NA | NA |
| OTU17737 | - | NA | + | NA |
| OTU14258 | NA | - | NA | NA |
| OTU17357 | NA | NA | NA | NA |
| OTU5367 | NA | NA | NA | NA |
| OTU6561 | NA | + | NA | - |
| OTU1280 | NA | - | + | NA |
| OTU1256 | NA | NA | NA | NA |
| OTU14646 | NA | NA | NA | NA |
| OTU17590 | NA | NA | NA | NA |
| OTU6612 | NA | NA | NA | NA |
| OTU10519 | NA | NA | NA | NA |
| OTU4428 | NA | NA | NA | - |
| OTU5308 | NA | NA | NA | + |
| OTU17065 | NA | NA | NA | NA |
| OTU5567 | NA | NA | NA | NA |
| OTU8379 | NA | NA | NA | NA |
| OTU8153 | NA | + | NA | - |
| OTU7703 | NA | NA | NA | NA |
| OTU975 | NA | NA | NA | NA |
| OTU14091 | NA | NA | NA | + |
| OTU15554 | NA | NA | NA | NA |
| OTU5023 | NA | + | NA | NA |
| OTU2170 | NA | NA | NA | NA |
| OTU8415 | NA | NA | NA | - |
| OTU18281 | NA | NA | NA | NA |
| OTU2988 | NA | NA | NA | NA |
| OTU14983 | NA | NA | NA | NA |
| OTU18164 | NA | + | NA | NA |
| OTU9004 | NA | NA | NA | - |
| OTU17290 | NA | NA | NA | NA |
| OTU3052 | + | NA | NA | NA |
| OTU13738 | NA | NA | NA | NA |
| OTU12166 | NA | NA | NA | NA |
| OTU14405 | NA | - | + | - |
| OTU18323 | NA | NA | + | NA |
| OTU1451 | NA | NA | NA | + |
| OTU4579 | NA | NA | NA | NA |
| OTU18013 | NA | NA | NA | NA |
| OTU17368 | NA | NA | NA | NA |
| OTU443 | + | NA | NA | NA |
| OTU1885 | NA | NA | NA | NA |
| OTU6806 | NA | NA | NA | NA |
| OTU13963 | NA | NA | NA | NA |
| OTU15921 | NA | NA | NA | NA |
| OTU16023 | NA | NA | NA | NA |
| OTU8002 | NA | NA | NA | NA |
| OTU1155 | NA | NA | NA | - |
| OTU15979 | NA | NA | NA | NA |
| OTU8959 | NA | NA | NA | + |
| OTU17141 | NA | NA | - | NA |
| OTU12784 | NA | NA | + | NA |
| OTU12958 | NA | NA | NA | NA |
| OTU1123 | + | NA | NA | NA |
| OTU3406 | NA | NA | NA | NA |
| OTU18820 | NA | NA | NA | NA |
| OTU9421 | NA | NA | NA | NA |
| OTU3071 | NA | NA | NA | NA |
| OTU9894 | NA | NA | NA | NA |
| OTU10013 | NA | NA | NA | NA |
| OTU7324 | NA | NA | NA | NA |
| OTU453 | NA | NA | NA | NA |
| OTU17656 | NA | NA | NA | NA |
| OTU5287 | NA | + | NA | NA |
| OTU7862 | NA | NA | NA | - |
| OTU4335 | + | NA | NA | NA |
| OTU13234 | NA | NA | NA | NA |
| OTU3919 | NA | NA | NA | NA |
| OTU153 | NA | NA | NA | NA |
| OTU5346 | NA | NA | - | + |
| OTU3066 | + | NA | NA | NA |
| OTU6187 | NA | NA | NA | NA |
| OTU5957 | NA | NA | NA | - |
| OTU763 | + | NA | NA | NA |
| OTU17710 | NA | NA | NA | NA |
| OTU8038 | NA | NA | NA | NA |
| OTU17596 | NA | NA | NA | NA |
| OTU10262 | NA | - | NA | NA |
| OTU15422 | NA | NA | NA | NA |
| OTU14231 | NA | NA | + | - |
| OTU12530 | NA | NA | NA | NA |
| OTU13726 | + | NA | NA | NA |
| OTU9240 | + | NA | NA | NA |
| OTU13378 | NA | + | NA | NA |
| OTU15427 | NA | NA | NA | NA |
| OTU18339 | NA | NA | NA | + |
| OTU14847 | NA | + | NA | NA |
| OTU5950 | NA | NA | NA | NA |
| OTU8336 | NA | NA | NA | - |
| OTU2246 | NA | NA | NA | NA |
| OTU6611 | NA | NA | - | + |
| OTU1377 | NA | NA | NA | NA |
| OTU1212 | NA | NA | NA | NA |
| OTU6018 | NA | NA | NA | NA |
| OTU14955 | NA | - | + | NA |
| OTU15521 | NA | NA | NA | NA |
| OTU18463 | NA | NA | NA | NA |
| OTU4517 | NA | NA | NA | NA |
| OTU14205 | + | NA | NA | NA |
| OTU7844 | NA | NA | NA | NA |
| OTU16297 | + | NA | NA | NA |
| OTU15871 | NA | NA | NA | NA |
| OTU1970 | NA | - | + | NA |
| OTU8067 | NA | NA | NA | NA |
| OTU12575 | + | NA | NA | NA |
| OTU7081 | NA | NA | NA | NA |
| OTU1337 | NA | NA | NA | NA |
| OTU5589 | + | NA | NA | NA |
| OTU18628 | NA | NA | - | + |
| OTU6482 | NA | NA | NA | NA |
| OTU6633 | NA | NA | + | NA |
| OTU2316 | NA | NA | NA | NA |
| OTU4344 | NA | NA | NA | NA |
| OTU19092 | + | NA | NA | NA |
| OTU14957 | NA | NA | + | NA |
| OTU17158 | NA | NA | NA | + |
| OTU11534 | NA | NA | + | NA |
| OTU7323 | NA | + | NA | - |
| OTU8337 | NA | NA | NA | - |
| OTU14824 | NA | NA | + | NA |
| OTU6614 | NA | NA | NA | NA |
| OTU12652 | NA | NA | NA | NA |
| OTU14332 | + | - | NA | NA |
| OTU14543 | NA | NA | NA | - |
| OTU11718 | NA | NA | NA | NA |
| OTU13125 | NA | NA | + | NA |
| OTU9112 | + | NA | - | NA |
| OTU13631 | + | - | NA | NA |
| OTU12522 | NA | NA | NA | - |
| OTU1186 | + | NA | NA | NA |
| OTU12793 | + | NA | NA | NA |
| OTU1692 | + | NA | NA | NA |
| OTU6176 | NA | + | NA | NA |
| OTU1421 | NA | NA | NA | NA |
| OTU16490 | NA | NA | NA | - |
| OTU9391 | NA | + | - | NA |
| OTU6159 | NA | NA | NA | NA |
| OTU19002 | NA | NA | NA | NA |
| OTU17776 | NA | NA | NA | + |
| OTU716 | NA | NA | NA | NA |
| OTU14303 | NA | NA | + | NA |
| OTU11749 | NA | NA | NA | NA |
| OTU13589 | NA | NA | NA | NA |
| OTU11421 | NA | NA | NA | - |
| OTU5297 | NA | NA | NA | NA |
| OTU13515 | NA | NA | NA | NA |
| OTU16192 | NA | NA | NA | NA |
| OTU14267 | + | NA | NA | NA |
| OTU7167 | NA | NA | NA | NA |
| OTU5728 | + | NA | NA | NA |
| OTU9152 | NA | NA | NA | NA |
| OTU16141 | NA | - | NA | NA |
| OTU1368 | NA | NA | NA | NA |
| OTU14637 | NA | NA | NA | NA |
| OTU18245 | NA | NA | NA | NA |
| OTU15754 | NA | NA | NA | NA |
| OTU2053 | NA | NA | NA | NA |
| OTU6809 | NA | NA | NA | NA |
| OTU2411 | + | NA | NA | NA |
| OTU5077 | NA | NA | NA | NA |
| OTU17760 | NA | NA | NA | NA |
| OTU10913 | NA | NA | - | NA |
| OTU4763 | NA | NA | NA | + |
| OTU14273 | NA | NA | NA | NA |
| OTU9105 | + | NA | NA | NA |
| OTU15951 | + | NA | NA | NA |
| OTU1547 | NA | NA | NA | NA |
| OTU461 | NA | NA | NA | NA |
| OTU9312 | NA | NA | NA | NA |
| OTU6213 | NA | - | NA | NA |
| OTU95 | NA | NA | NA | NA |
| OTU3578 | NA | NA | NA | + |
| OTU16259 | NA | NA | NA | NA |
| OTU1169 | NA | NA | NA | NA |
| OTU5746 | NA | + | NA | NA |
| OTU7562 | NA | NA | NA | NA |
| OTU14890 | NA | NA | + | NA |
| OTU7587 | + | NA | NA | NA |
| OTU1017 | NA | + | NA | NA |
| OTU1667 | NA | NA | NA | NA |
| OTU1575 | NA | NA | NA | NA |
| OTU8280 | + | NA | NA | NA |
| OTU6512 | NA | + | NA | NA |
| OTU174 | + | NA | + | NA |
| OTU7418 | NA | NA | NA | NA |
| OTU74 | NA | NA | NA | - |
| OTU5070 | NA | NA | NA | NA |
| OTU1225 | NA | NA | NA | + |
| OTU2911 | NA | NA | NA | NA |
| OTU7724 | NA | NA | NA | NA |
| OTU6703 | + | NA | NA | NA |
| OTU5481 | NA | NA | NA | + |
| OTU10127 | NA | NA | NA | NA |
| OTU7855 | + | NA | NA | NA |
| OTU15861 | - | + | NA | NA |
| OTU5875 | NA | NA | NA | - |
| OTU615 | NA | + | NA | NA |
| OTU7529 | NA | NA | NA | NA |
| OTU6373 | NA | NA | - | + |
| OTU7219 | NA | NA | - | NA |
| OTU12972 | + | NA | NA | - |
| OTU8964 | NA | + | NA | NA |
| OTU42 | NA | NA | NA | NA |
| OTU19015 | NA | NA | NA | NA |
| OTU2030 | NA | NA | - | NA |
| OTU4234 | NA | NA | NA | NA |
| OTU1598 | NA | NA | NA | NA |
| OTU6899 | NA | NA | NA | NA |
| OTU4580 | NA | + | NA | NA |
| OTU7972 | NA | + | NA | NA |
| OTU12349 | NA | NA | - | NA |
| OTU3403 | NA | NA | + | NA |
| OTU12725 | NA | NA | NA | NA |
| OTU6923 | NA | NA | NA | NA |
| OTU15265 | NA | NA | NA | + |
| OTU10873 | NA | NA | + | NA |
| OTU9767 | NA | NA | NA | NA |
| OTU18826 | NA | NA | NA | NA |
| OTU9140 | + | NA | NA | NA |
| OTU15160 | NA | NA | NA | NA |
| OTU17705 | NA | NA | - | + |
| OTU7717 | + | NA | - | NA |
| OTU15294 | NA | NA | NA | NA |
| OTU2199 | NA | NA | NA | NA |
| OTU472 | + | NA | NA | NA |
| OTU4935 | NA | NA | NA | NA |
| OTU13115 | NA | NA | NA | NA |
| OTU5285 | NA | NA | NA | NA |
| OTU13641 | NA | NA | NA | NA |
| OTU48 | + | - | NA | NA |
| OTU9968 | NA | NA | NA | - |
| OTU983 | + | NA | NA | NA |
| OTU5610 | NA | NA | NA | NA |
| OTU1290 | + | NA | NA | NA |
| OTU2843 | + | NA | NA | NA |
| OTU6292 | NA | NA | NA | NA |
| OTU13938 | NA | NA | NA | NA |
| OTU18917 | NA | NA | NA | NA |
| OTU9329 | NA | NA | NA | NA |
| OTU14102 | NA | NA | NA | NA |
| OTU14877 | NA | NA | NA | NA |
| OTU16918 | NA | NA | NA | NA |
| OTU17650 | NA | NA | NA | + |
| OTU2166 | NA | NA | NA | NA |
| OTU15815 | NA | NA | NA | NA |
| OTU15081 | NA | NA | NA | NA |
| OTU7631 | NA | NA | - | NA |
| OTU5388 | NA | NA | NA | NA |
| OTU4657 | NA | NA | NA | NA |
| OTU3942 | NA | NA | NA | NA |
| OTU2097 | NA | NA | NA | NA |
| OTU5015 | NA | + | NA | NA |
| OTU4083 | NA | NA | NA | NA |
| OTU5024 | NA | NA | NA | NA |
| OTU17452 | NA | NA | NA | NA |
| OTU17942 | NA | NA | + | NA |
| OTU11662 | NA | NA | NA | - |
| OTU16174 | NA | NA | NA | NA |
| OTU3445 | NA | NA | NA | NA |
| OTU5398 | NA | NA | NA | - |
| OTU6866 | NA | NA | NA | NA |
| OTU262 | NA | NA | NA | + |
| OTU15781 | NA | NA | NA | + |
| OTU6450 | NA | NA | NA | NA |
| OTU9677 | NA | NA | NA | NA |
| OTU8870 | NA | NA | NA | NA |
| OTU9675 | NA | NA | NA | NA |
| OTU15960 | NA | NA | NA | NA |
| OTU9916 | NA | - | + | NA |
| OTU11643 | NA | NA | NA | NA |
| OTU16690 | NA | NA | NA | NA |
| OTU8746 | + | NA | NA | NA |
| OTU523 | + | NA | NA | NA |
| OTU10960 | + | NA | NA | NA |
| OTU17098 | NA | NA | NA | + |
| OTU7594 | NA | NA | - | + |
| OTU14359 | NA | NA | + | NA |
| OTU11080 | NA | NA | NA | NA |
| OTU6840 | NA | NA | NA | NA |
| OTU14525 | NA | NA | + | NA |
| OTU18675 | + | NA | NA | NA |
| OTU5837 | NA | NA | NA | NA |
| OTU8296 | NA | NA | NA | NA |
| OTU11082 | NA | - | + | NA |
| OTU6224 | NA | NA | NA | NA |
| OTU16760 | NA | NA | NA | NA |
| OTU6234 | NA | NA | NA | NA |
| OTU85 | NA | NA | NA | NA |
| OTU1607 | NA | NA | NA | NA |
| OTU15390 | NA | NA | NA | NA |
| OTU6054 | NA | NA | NA | NA |
| OTU498 | NA | NA | NA | + |
| OTU11188 | NA | NA | + | NA |
| OTU15491 | NA | NA | NA | + |
| OTU232 | + | NA | NA | NA |
| OTU1842 | + | NA | NA | NA |
| OTU12104 | NA | NA | NA | NA |
| OTU7999 | NA | NA | NA | NA |
| OTU9185 | NA | NA | NA | NA |
| OTU321 | NA | NA | NA | NA |
| OTU17320 | NA | NA | NA | NA |
| OTU13399 | NA | NA | NA | NA |
| OTU5954 | NA | NA | NA | NA |
| OTU12877 | + | NA | NA | NA |
| OTU9320 | + | NA | NA | NA |
| OTU6679 | + | NA | NA | NA |
| OTU17118 | NA | NA | NA | + |
| OTU4109 | NA | + | NA | NA |
| OTU14245 | + | - | NA | NA |
| OTU14687 | + | NA | NA | NA |
| OTU15519 | NA | NA | NA | NA |
| OTU12350 | + | NA | NA | NA |
| OTU6934 | - | + | NA | NA |
| OTU7814 | NA | NA | NA | NA |
| OTU4153 | + | NA | NA | - |
| OTU18360 | NA | NA | NA | NA |
| OTU18608 | NA | - | NA | NA |
| OTU9337 | NA | NA | - | NA |
| OTU14819 | NA | NA | + | NA |
| OTU15498 | NA | - | NA | + |
| OTU3303 | NA | NA | NA | NA |
| OTU6800 | NA | NA | NA | NA |
| OTU9907 | NA | NA | NA | NA |
| OTU9233 | + | NA | NA | NA |
| OTU16123 | NA | NA | NA | + |
| OTU299 | NA | - | NA | + |
| OTU14137 | NA | NA | NA | NA |
| OTU15896 | NA | NA | NA | NA |
| OTU1185 | NA | NA | NA | NA |
| OTU1829 | NA | - | NA | NA |
| OTU6014 | NA | NA | NA | NA |
| OTU3475 | + | NA | NA | NA |
| OTU6033 | NA | NA | NA | + |
| OTU1449 | + | - | NA | NA |
| OTU15605 | NA | NA | NA | NA |
| OTU3062 | NA | NA | NA | NA |
| OTU12537 | + | NA | NA | NA |
| OTU6834 | NA | NA | NA | NA |
| OTU17576 | + | NA | NA | NA |
| OTU519 | NA | NA | NA | NA |
| OTU1800 | NA | - | NA | + |
| OTU5625 | NA | NA | NA | - |
| OTU15404 | NA | - | NA | + |
| OTU4046 | NA | NA | NA | NA |
| OTU4410 | NA | NA | NA | NA |
| OTU8144 | NA | NA | NA | NA |
| OTU5101 | NA | NA | NA | NA |
| OTU7896 | NA | NA | NA | NA |
| OTU2276 | NA | NA | NA | NA |
| OTU539 | + | NA | NA | NA |
| OTU201 | NA | NA | NA | NA |
| OTU13394 | NA | NA | NA | NA |
| OTU14249 | NA | NA | NA | NA |
| OTU12626 | NA | NA | + | NA |
| OTU4569 | NA | NA | NA | NA |
| OTU3338 | NA | NA | NA | NA |
| OTU9702 | NA | NA | NA | - |
| OTU11897 | NA | NA | + | NA |
| OTU4045 | NA | NA | NA | NA |
| OTU18550 | NA | NA | NA | + |
| OTU12115 | NA | NA | NA | NA |
| OTU6607 | NA | NA | NA | - |
| OTU6291 | NA | NA | NA | NA |
| OTU11607 | NA | NA | NA | NA |
| OTU914 | + | NA | NA | NA |
| OTU16330 | + | - | NA | NA |
| OTU1214 | + | - | NA | NA |
| OTU1264 | + | NA | NA | NA |
| OTU3601 | NA | - | NA | NA |
| OTU16425 | NA | NA | NA | NA |
| OTU1139 | NA | NA | NA | NA |
| OTU9989 | NA | - | NA | + |
| OTU8118 | NA | NA | - | NA |
| OTU557 | NA | NA | NA | NA |
| OTU18326 | NA | NA | NA | + |
| OTU6556 | + | NA | NA | NA |
| OTU668 | NA | NA | NA | NA |
| OTU17070 | NA | + | NA | NA |
| OTU8383 | + | NA | NA | NA |
| OTU14431 | NA | NA | NA | NA |
| OTU1495 | NA | NA | NA | NA |
| OTU2408 | NA | NA | NA | NA |
| OTU6002 | NA | NA | NA | NA |
| OTU15092 | NA | NA | NA | - |
| OTU11122 | NA | NA | NA | NA |
| OTU2355 | NA | NA | NA | NA |
| OTU8131 | NA | NA | NA | NA |
| OTU3995 | NA | + | - | NA |
| OTU13360 | NA | + | NA | NA |
| OTU18693 | NA | NA | NA | + |
| OTU17039 | - | + | NA | NA |
| OTU15482 | NA | NA | + | NA |
| OTU18657 | + | NA | NA | NA |
| OTU16797 | NA | - | + | NA |
| OTU10975 | NA | NA | NA | NA |
| OTU18795 | NA | NA | NA | NA |
| OTU16571 | + | NA | NA | NA |
| OTU15191 | NA | NA | NA | NA |
| OTU5920 | + | NA | - | NA |
| OTU18821 | NA | NA | NA | NA |
| OTU13723 | NA | + | NA | NA |
| OTU17494 | NA | NA | NA | + |
| OTU13169 | NA | NA | NA | NA |
| OTU121 | + | - | + | - |
| OTU965 | NA | NA | - | NA |
| OTU7548 | NA | NA | NA | NA |
| OTU9290 | NA | NA | NA | NA |
| OTU8286 | NA | NA | NA | NA |
| OTU433 | NA | NA | NA | NA |
| OTU12364 | + | - | NA | NA |
| OTU11833 | NA | NA | NA | NA |
| OTU14265 | NA | NA | NA | NA |
| OTU8966 | NA | NA | NA | NA |
| OTU5945 | NA | + | NA | NA |
| OTU4843 | NA | NA | + | NA |
| OTU16163 | NA | - | NA | + |
| OTU14474 | NA | NA | NA | NA |
| OTU12531 | NA | NA | + | NA |
| OTU10157 | NA | NA | NA | NA |
| OTU4824 | NA | NA | NA | NA |
| OTU3548 | NA | NA | - | NA |
| OTU10819 | + | NA | NA | NA |
| OTU8150 | + | NA | NA | NA |
| OTU10272 | NA | NA | NA | + |
| OTU12311 | NA | NA | NA | NA |
| OTU3481 | + | NA | NA | NA |
| OTU7673 | NA | NA | NA | NA |
| OTU412 | NA | NA | NA | NA |
| OTU10015 | + | - | NA | NA |
| OTU16803 | NA | NA | + | NA |
| OTU15797 | NA | NA | NA | NA |
| OTU10792 | NA | NA | NA | NA |
| OTU17985 | NA | NA | NA | NA |
| OTU10146 | NA | NA | + | - |
| OTU8232 | NA | NA | NA | NA |
| OTU14083 | NA | NA | NA | + |
| OTU5296 | NA | NA | NA | NA |
| OTU9113 | + | NA | NA | NA |
| OTU11014 | NA | - | NA | NA |
| OTU16170 | + | - | NA | NA |
| OTU18286 | NA | - | + | NA |
| OTU6636 | NA | NA | NA | NA |
| OTU9075 | NA | NA | NA | - |
| OTU3670 | NA | NA | NA | NA |
| OTU10312 | NA | NA | NA | NA |
| OTU3897 | NA | - | NA | + |
| OTU6303 | NA | NA | NA | NA |
| OTU6222 | NA | NA | NA | + |
| OTU11887 | NA | NA | NA | NA |
| OTU14953 | + | NA | NA | NA |
| OTU12675 | NA | NA | + | NA |
| OTU2627 | NA | NA | NA | NA |
| OTU2562 | + | - | NA | NA |
| OTU11022 | NA | NA | NA | NA |
| OTU3137 | + | - | NA | NA |
| OTU12273 | NA | NA | NA | NA |
| OTU6195 | NA | NA | NA | NA |
| OTU3473 | NA | NA | NA | + |
| OTU6584 | + | NA | NA | NA |
| OTU3438 | NA | NA | NA | NA |
| OTU13092 | NA | NA | NA | NA |
| OTU1799 | NA | NA | NA | NA |
| OTU14653 | NA | NA | + | NA |
| OTU1772 | + | NA | NA | NA |
| OTU3760 | NA | NA | NA | NA |
| OTU9100 | NA | NA | NA | NA |
| OTU9622 | NA | NA | NA | NA |
| OTU17493 | NA | NA | NA | NA |
| OTU8553 | + | NA | NA | NA |
| OTU1540 | NA | NA | NA | NA |
| OTU4556 | NA | - | NA | NA |
| OTU5724 | NA | NA | NA | NA |
| OTU5687 | NA | NA | - | NA |
| OTU6155 | NA | NA | NA | NA |
| OTU15434 | NA | NA | NA | NA |
| OTU18698 | NA | NA | NA | + |
| OTU5608 | NA | NA | NA | NA |
| OTU8757 | NA | + | NA | NA |
| OTU18150 | NA | NA | NA | + |
| OTU13710 | NA | NA | NA | NA |
| OTU7939 | + | NA | NA | NA |
| OTU663 | NA | NA | NA | NA |
| OTU7074 | NA | NA | NA | NA |
| OTU3856 | NA | NA | NA | NA |
| OTU1781 | NA | NA | NA | NA |
| OTU2941 | NA | NA | NA | NA |
| OTU5108 | NA | + | NA | NA |
| OTU7487 | + | NA | NA | NA |
| OTU14759 | + | - | NA | NA |
| OTU6157 | NA | NA | NA | NA |
| OTU10331 | NA | NA | NA | NA |
| OTU15470 | NA | NA | NA | NA |
| OTU2274 | NA | NA | NA | NA |
| OTU7361 | NA | NA | NA | NA |
| OTU8176 | NA | NA | NA | NA |
| OTU15901 | NA | NA | NA | + |
| OTU13065 | NA | NA | NA | NA |
| OTU19106 | NA | NA | NA | NA |
| OTU11321 | NA | NA | NA | NA |
| OTU9597 | NA | NA | NA | + |
| OTU15497 | NA | NA | NA | + |
| OTU12291 | NA | NA | NA | NA |
| OTU9281 | NA | NA | NA | NA |
| OTU12627 | NA | NA | + | NA |
| OTU2015 | + | NA | NA | NA |
| OTU13926 | NA | NA | NA | NA |
| OTU11513 | NA | NA | NA | NA |
| OTU10278 | NA | NA | NA | NA |
| OTU11088 | NA | NA | + | NA |
| OTU12671 | NA | NA | NA | + |
| OTU11722 | NA | NA | NA | NA |
| OTU6848 | NA | NA | NA | NA |
| OTU4509 | NA | NA | NA | + |
| OTU11199 | NA | NA | + | NA |
| OTU8362 | + | - | NA | NA |
| OTU4342 | + | NA | NA | NA |
| OTU3585 | NA | NA | NA | NA |
| OTU10676 | NA | NA | NA | NA |
| OTU13341 | NA | NA | NA | NA |
| OTU3155 | NA | NA | NA | NA |
| OTU5453 | NA | + | NA | - |
| OTU122 | NA | NA | NA | NA |
| OTU3191 | NA | NA | - | NA |
| OTU14255 | NA | NA | + | NA |
| OTU6333 | + | NA | NA | NA |
| OTU14164 | NA | NA | + | NA |
| OTU11755 | NA | NA | NA | NA |
| OTU3837 | NA | NA | NA | NA |
| OTU12660 | NA | NA | NA | NA |
| OTU4689 | NA | NA | NA | NA |
| OTU14825 | NA | NA | NA | + |
| OTU6256 | + | NA | NA | NA |
| OTU2286 | NA | NA | NA | NA |
| OTU2688 | NA | NA | NA | NA |
| OTU10828 | NA | NA | + | NA |
| OTU2319 | + | NA | NA | NA |
| OTU2153 | NA | NA | NA | NA |
| OTU6765 | NA | NA | NA | NA |
| OTU10968 | NA | NA | NA | NA |
| OTU13652 | NA | NA | + | NA |
| OTU8544 | NA | + | NA | NA |
| OTU12194 | NA | NA | NA | NA |
| OTU13413 | NA | NA | NA | NA |
| OTU11946 | NA | NA | + | NA |
| OTU3404 | + | NA | NA | NA |
| OTU7956 | + | NA | NA | NA |
| OTU17229 | NA | NA | - | + |
| OTU16063 | NA | NA | NA | NA |
| OTU3258 | NA | NA | - | + |
| OTU3598 | + | NA | NA | NA |
| OTU17153 | NA | - | NA | + |
| OTU18706 | NA | NA | NA | NA |
| OTU4641 | + | NA | NA | NA |
| OTU10917 | NA | NA | NA | NA |
| OTU8310 | + | NA | NA | NA |
| OTU12187 | NA | NA | NA | NA |
| OTU738 | + | NA | NA | NA |
| OTU311 | NA | NA | NA | NA |
| OTU10656 | NA | NA | NA | NA |
| OTU12810 | NA | NA | NA | NA |
| OTU8755 | NA | NA | NA | + |
| OTU3921 | NA | - | NA | NA |
| OTU11541 | + | NA | NA | NA |
| OTU15966 | + | - | NA | NA |
| OTU1682 | NA | NA | NA | NA |
| OTU11752 | + | NA | NA | NA |
| OTU5457 | NA | NA | NA | NA |
| OTU563 | NA | NA | NA | NA |
| OTU2092 | + | NA | NA | NA |
| OTU15694 | NA | NA | NA | NA |
| OTU16177 | NA | NA | NA | NA |
| OTU47 | NA | NA | NA | + |
| OTU6521 | NA | NA | NA | NA |
| OTU6538 | NA | NA | NA | NA |
| OTU4321 | NA | NA | NA | NA |
| OTU5700 | NA | + | NA | NA |
| OTU4317 | + | NA | NA | NA |
| OTU5506 | NA | NA | NA | NA |
| OTU10927 | + | NA | NA | NA |
| OTU16945 | NA | NA | NA | - |
| OTU17994 | NA | NA | NA | + |
| OTU12630 | NA | NA | + | NA |
| OTU499 | NA | - | NA | + |
| OTU1204 | NA | NA | NA | NA |
| OTU16335 | NA | - | NA | NA |
| OTU8302 | + | NA | NA | NA |
| OTU6710 | NA | NA | + | NA |
| OTU17007 | NA | NA | NA | NA |
| OTU11452 | + | - | NA | - |
| OTU13194 | NA | + | NA | NA |
| OTU9813 | NA | NA | NA | NA |
| OTU15551 | NA | NA | + | NA |
| OTU12043 | NA | NA | + | NA |
| OTU3605 | + | NA | - | NA |
| OTU18320 | NA | NA | + | NA |
| OTU7423 | + | NA | NA | NA |
| OTU445 | + | NA | NA | NA |
| OTU6137 | + | NA | NA | NA |
| OTU893 | NA | NA | NA | NA |
| OTU4044 | NA | NA | NA | NA |
| OTU15512 | NA | NA | NA | NA |
| OTU15344 | NA | NA | NA | + |
| OTU15520 | NA | - | NA | NA |
| OTU16128 | NA | NA | NA | NA |
| OTU9 | NA | NA | NA | NA |
| OTU1775 | NA | NA | NA | NA |
| OTU12296 | NA | - | NA | NA |
| OTU17620 | NA | NA | - | + |
| OTU4981 | NA | NA | NA | NA |
| OTU4916 | NA | NA | NA | NA |
| OTU6600 | NA | NA | NA | NA |
| OTU11231 | NA | NA | NA | NA |
| OTU1690 | NA | NA | NA | NA |
| OTU3519 | NA | NA | NA | + |
| OTU11908 | NA | - | + | NA |
| OTU15481 | NA | NA | NA | NA |
| OTU2225 | NA | NA | NA | + |
| OTU10984 | NA | NA | NA | NA |
| OTU10025 | + | - | NA | NA |
| OTU10028 | NA | NA | NA | NA |
| OTU1226 | NA | NA | NA | NA |
| OTU5646 | NA | NA | NA | NA |
| OTU2885 | + | NA | NA | NA |
| OTU5254 | NA | NA | NA | NA |
| OTU1222 | NA | - | + | NA |
| OTU10381 | NA | NA | NA | NA |
| OTU5042 | NA | NA | NA | NA |
| OTU6400 | + | NA | NA | NA |
| OTU13653 | NA | NA | NA | NA |
| OTU15539 | NA | - | + | NA |
| OTU16616 | NA | NA | NA | + |
| OTU1209 | NA | NA | + | NA |
| OTU11475 | NA | NA | NA | NA |
| OTU15211 | NA | - | NA | + |
| OTU4865 | NA | NA | NA | NA |
| OTU10988 | + | NA | NA | NA |
| OTU8573 | NA | NA | NA | NA |
| OTU12532 | NA | NA | NA | NA |
| OTU13064 | NA | NA | NA | NA |
| OTU11615 | NA | NA | + | NA |
| OTU259 | + | NA | NA | NA |
| OTU18279 | NA | NA | NA | NA |
| OTU10817 | NA | NA | + | NA |
| OTU12456 | NA | NA | NA | NA |
| OTU9919 | + | - | NA | NA |
| OTU1618 | NA | NA | NA | NA |
| OTU596 | + | NA | NA | NA |
| OTU17093 | NA | NA | NA | NA |
| OTU9326 | NA | NA | NA | NA |
| OTU7297 | + | NA | NA | NA |
| OTU11306 | NA | NA | NA | NA |
| OTU7645 | NA | + | NA | NA |
| OTU4027 | NA | NA | NA | NA |
| OTU5663 | NA | NA | NA | NA |
| OTU6248 | NA | NA | NA | NA |
| OTU15178 | NA | NA | NA | + |
| OTU16734 | NA | + | NA | NA |
| OTU12581 | NA | NA | NA | NA |
| OTU11016 | NA | - | NA | NA |
| OTU13009 | NA | NA | NA | NA |
| OTU18919 | NA | NA | NA | NA |
| OTU5953 | NA | NA | NA | NA |
| OTU15139 | NA | NA | NA | NA |
| OTU5774 | NA | + | NA | NA |
| OTU11258 | NA | NA | NA | NA |
| OTU11450 | NA | NA | NA | NA |
| OTU6835 | NA | NA | NA | NA |
| OTU1545 | + | NA | NA | NA |
| OTU3314 | NA | NA | NA | + |
| OTU11437 | NA | NA | NA | NA |
| OTU10402 | NA | - | NA | NA |
| OTU15705 | + | NA | NA | NA |
| OTU11371 | + | NA | + | NA |
| OTU14192 | NA | NA | NA | NA |
| OTU3333 | + | NA | NA | NA |
| OTU12 | NA | NA | NA | NA |
| OTU4334 | NA | NA | NA | NA |
| OTU10663 | NA | NA | NA | - |
| OTU7246 | + | NA | NA | NA |
| OTU1342 | NA | NA | NA | NA |
| OTU13376 | NA | NA | NA | + |
| OTU1884 | NA | NA | NA | NA |
| OTU11902 | + | NA | NA | NA |
| OTU9952 | NA | NA | NA | NA |
| OTU7443 | NA | NA | NA | NA |
| OTU18358 | NA | - | NA | NA |
| OTU12874 | NA | NA | NA | NA |
| OTU1144 | + | NA | NA | NA |
| OTU2977 | NA | NA | NA | NA |
| OTU50 | NA | NA | NA | NA |
| OTU11486 | + | NA | NA | NA |
| OTU9847 | NA | NA | + | NA |
| OTU9880 | NA | NA | NA | NA |
| OTU618 | + | NA | NA | NA |
| OTU3072 | + | NA | NA | NA |
| OTU16858 | NA | NA | NA | NA |
| OTU5006 | NA | NA | NA | NA |
| OTU3386 | NA | NA | NA | NA |
| OTU17568 | NA | NA | - | + |
| OTU12595 | NA | - | + | NA |
| OTU1588 | NA | NA | - | NA |
| OTU5624 | NA | NA | NA | NA |
| OTU6336 | NA | NA | + | - |
| OTU14423 | NA | NA | + | NA |
| OTU14015 | NA | NA | NA | NA |
| OTU4347 | NA | NA | NA | NA |
| OTU10422 | NA | NA | NA | NA |
| OTU17086 | NA | - | NA | NA |
| OTU13380 | NA | NA | + | NA |
| OTU17837 | NA | NA | NA | NA |
| OTU354 | + | NA | NA | NA |
| OTU8077 | NA | NA | NA | NA |
| OTU6644 | + | NA | NA | NA |
| OTU15173 | NA | NA | NA | NA |
| OTU6589 | NA | NA | NA | NA |
| OTU15222 | NA | NA | NA | NA |
| OTU8920 | NA | NA | NA | NA |
| OTU12908 | NA | NA | NA | NA |
| OTU8975 | NA | NA | NA | NA |
| OTU559 | + | NA | NA | NA |
| OTU18280 | NA | NA | NA | + |
| OTU9628 | NA | NA | NA | NA |
| OTU13085 | NA | NA | NA | NA |
| OTU14233 | NA | NA | NA | NA |
| OTU18296 | + | NA | NA | NA |
| OTU1203 | NA | NA | NA | NA |
| OTU6430 | NA | NA | NA | NA |
| OTU5897 | NA | NA | NA | NA |
| OTU15461 | NA | NA | NA | + |
| OTU3358 | NA | NA | NA | + |
| OTU14077 | + | NA | NA | NA |
| OTU14210 | NA | NA | NA | NA |
| OTU10372 | + | - | NA | NA |
| OTU15447 | NA | NA | - | + |
| OTU5916 | NA | NA | - | NA |
| OTU6451 | NA | + | NA | NA |
| OTU8685 | NA | NA | NA | - |
| OTU9872 | NA | NA | NA | NA |
| OTU17964 | NA | NA | NA | NA |
| OTU398 | + | NA | NA | NA |
| OTU6452 | NA | NA | NA | NA |
| OTU5859 | NA | NA | NA | NA |
| OTU17935 | NA | NA | NA | NA |
| OTU18272 | NA | NA | NA | + |
| OTU12805 | NA | NA | NA | NA |
| OTU5026 | NA | NA | NA | NA |
| OTU1554 | NA | NA | NA | NA |
| OTU6540 | NA | NA | NA | - |
| OTU1482 | + | NA | - | NA |
| OTU15748 | NA | NA | NA | + |
| OTU18297 | NA | NA | NA | NA |
| OTU12893 | NA | NA | NA | NA |
| OTU10526 | NA | - | + | - |
| OTU18118 | NA | NA | NA | NA |
| OTU3254 | + | NA | NA | NA |
| OTU8047 | NA | NA | NA | NA |
| OTU4555 | NA | NA | NA | NA |
| OTU3671 | NA | NA | NA | - |
| OTU5833 | NA | + | NA | NA |
| OTU16157 | NA | NA | NA | NA |
| OTU5662 | NA | + | NA | NA |
| OTU131 | NA | + | NA | NA |
| OTU8720 | NA | NA | - | + |
| OTU3900 | NA | NA | NA | NA |
| OTU19134 | NA | NA | + | - |
| OTU1777 | NA | NA | + | NA |
| OTU1930 | NA | NA | NA | NA |
| OTU12870 | NA | NA | NA | NA |
| OTU7346 | NA | NA | NA | - |
| OTU13439 | NA | NA | NA | NA |
| OTU16311 | NA | NA | NA | NA |
| OTU1390 | + | NA | NA | NA |
| OTU276 | NA | NA | + | NA |
| OTU17926 | NA | NA | NA | NA |
| OTU2522 | NA | NA | NA | NA |
| OTU12913 | NA | NA | NA | NA |
| OTU5455 | NA | NA | + | NA |
| OTU14945 | NA | NA | NA | NA |
| OTU17634 | NA | NA | NA | NA |
| OTU12987 | NA | NA | NA | NA |
| OTU14070 | NA | NA | NA | NA |
| OTU2378 | + | NA | NA | NA |
| OTU16236 | NA | NA | NA | NA |
| OTU11579 | NA | - | + | NA |
| OTU5713 | NA | NA | NA | NA |
| OTU4078 | NA | NA | NA | NA |
| OTU1590 | + | NA | NA | NA |
| OTU347 | NA | NA | NA | NA |
| OTU11063 | + | - | NA | NA |
| OTU5583 | NA | NA | NA | NA |
| OTU729 | NA | NA | NA | NA |
| OTU13752 | NA | NA | NA | NA |
| OTU11736 | NA | NA | NA | NA |
| OTU17682 | + | - | NA | NA |
| OTU4897 | NA | NA | NA | - |
| OTU15153 | NA | NA | NA | NA |
| OTU940 | NA | NA | NA | NA |
| OTU15304 | NA | - | NA | NA |
| OTU16648 | NA | + | NA | NA |
| OTU17312 | NA | NA | NA | NA |
| OTU10031 | NA | NA | NA | NA |
| OTU16591 | NA | NA | NA | NA |
| OTU12600 | NA | NA | + | NA |
| OTU10382 | NA | NA | NA | + |
| OTU7476 | NA | NA | NA | NA |
| OTU12843 | - | + | NA | NA |
| OTU2395 | NA | NA | NA | NA |
| OTU11894 | NA | NA | + | NA |
| OTU3417 | + | NA | - | NA |
| OTU14712 | NA | NA | + | - |
| OTU7042 | NA | NA | NA | NA |
| OTU6049 | NA | NA | NA | NA |
| OTU18984 | NA | NA | NA | NA |
| OTU19081 | NA | NA | NA | NA |
| OTU12520 | NA | NA | NA | NA |
| OTU8325 | NA | NA | NA | NA |
| OTU5195 | NA | NA | NA | NA |
| OTU5538 | NA | NA | NA | NA |
| OTU5641 | NA | + | NA | - |
| OTU14402 | NA | NA | NA | NA |
| OTU7009 | NA | NA | NA | NA |
| OTU11801 | NA | NA | NA | NA |
| OTU4476 | + | NA | - | NA |
| OTU2990 | + | NA | NA | NA |
| OTU11947 | NA | NA | NA | NA |
| OTU6351 | + | NA | NA | NA |
| OTU11342 | NA | - | NA | NA |
| OTU2658 | NA | NA | NA | - |
| OTU11737 | NA | NA | NA | NA |
| OTU12642 | NA | NA | NA | NA |
| OTU8381 | + | NA | NA | NA |
| OTU9344 | NA | NA | NA | NA |
| OTU17287 | NA | NA | NA | NA |
| OTU5544 | NA | NA | NA | NA |
| OTU4818 | NA | NA | NA | NA |
| OTU5053 | NA | NA | NA | NA |
| OTU14204 | + | NA | NA | NA |
| OTU16462 | NA | NA | NA | NA |
| OTU12699 | + | - | NA | NA |
| OTU13519 | + | NA | NA | NA |
| OTU10204 | NA | NA | NA | - |
| OTU5789 | NA | NA | NA | NA |
| OTU18397 | NA | NA | NA | NA |
| OTU4364 | NA | NA | NA | NA |
| OTU9551 | NA | NA | NA | NA |
| OTU13046 | NA | NA | NA | NA |
| OTU1628 | NA | NA | NA | NA |
| OTU9082 | NA | NA | NA | NA |
| OTU14706 | NA | NA | NA | NA |
| OTU9359 | NA | NA | NA | NA |
| OTU1762 | NA | NA | NA | NA |
| OTU14230 | + | NA | NA | - |
| OTU2855 | + | - | NA | NA |
| OTU6655 | NA | NA | NA | + |
| OTU4469 | + | NA | NA | NA |
| OTU4082 | NA | NA | NA | NA |
| OTU7436 | + | NA | - | NA |
| OTU17187 | NA | NA | NA | + |
| OTU17114 | NA | NA | NA | NA |
| OTU10256 | NA | NA | NA | - |
| OTU14223 | NA | NA | NA | NA |
| OTU6724 | NA | NA | NA | NA |
| OTU1065 | NA | - | NA | NA |
| OTU1287 | + | NA | NA | NA |
| OTU5113 | NA | NA | NA | NA |
| OTU15912 | NA | NA | NA | NA |
| OTU17208 | NA | NA | NA | NA |
| OTU12610 | NA | NA | NA | NA |
| OTU12138 | NA | - | NA | NA |
| OTU16407 | NA | NA | NA | NA |
| OTU500 | NA | NA | NA | NA |
| OTU5242 | NA | NA | NA | NA |
| OTU7758 | NA | NA | NA | NA |
| OTU9869 | NA | NA | NA | NA |
| OTU11470 | + | - | NA | NA |
| OTU18248 | NA | NA | NA | + |
| OTU16116 | + | - | NA | NA |
| OTU1163 | NA | NA | NA | NA |
| OTU3031 | NA | NA | NA | NA |
| OTU470 | NA | NA | NA | NA |
| OTU13860 | + | - | NA | NA |
| OTU5919 | NA | NA | NA | NA |
| OTU16224 | NA | NA | NA | NA |
| OTU6571 | + | NA | NA | NA |
| OTU16611 | NA | NA | NA | NA |
| OTU5027 | NA | NA | NA | NA |
| OTU18230 | NA | NA | NA | NA |
| OTU5220 | NA | NA | NA | NA |
| OTU16232 | NA | NA | NA | NA |
| OTU12998 | NA | NA | NA | NA |
| OTU14344 | NA | NA | NA | NA |
| OTU45 | NA | NA | + | NA |
| OTU10680 | NA | NA | NA | NA |
| OTU12139 | NA | NA | NA | NA |
| OTU10783 | NA | NA | NA | - |
| OTU16679 | NA | NA | NA | NA |
| OTU18277 | NA | NA | NA | NA |
| OTU14097 | NA | NA | NA | NA |
| OTU15644 | NA | NA | NA | NA |
| OTU10511 | NA | NA | + | NA |
| OTU17646 | NA | NA | NA | + |
| OTU15706 | NA | NA | NA | + |
| OTU14092 | NA | - | + | NA |
| OTU15477 | NA | NA | NA | NA |
| OTU18575 | NA | + | NA | NA |
| OTU371 | NA | NA | NA | NA |
| OTU6443 | NA | NA | NA | NA |
| OTU10689 | NA | NA | NA | NA |
| OTU14191 | NA | NA | NA | NA |
| OTU14003 | NA | + | NA | NA |
| OTU9546 | NA | NA | NA | NA |
| OTU13825 | NA | NA | NA | NA |
| OTU3591 | NA | NA | NA | NA |
| OTU10627 | NA | NA | + | - |
| OTU9044 | NA | NA | - | NA |
| OTU19027 | NA | + | NA | NA |
| OTU9292 | NA | NA | NA | NA |
| OTU10050 | NA | NA | NA | NA |
| OTU13682 | NA | NA | NA | NA |
| OTU16233 | NA | NA | NA | NA |
| OTU2950 | NA | NA | NA | NA |
| OTU7531 | NA | + | NA | NA |
| OTU5131 | NA | NA | NA | NA |
| OTU14978 | NA | NA | NA | NA |
| OTU13899 | NA | + | NA | NA |
| OTU6605 | NA | - | NA | NA |
| OTU568 | + | NA | NA | NA |
| OTU17128 | NA | NA | NA | NA |
| OTU12306 | NA | NA | NA | NA |
| OTU1530 | NA | NA | NA | NA |
| OTU3251 | NA | - | NA | + |
| OTU4001 | NA | NA | NA | NA |
| OTU16518 | NA | - | NA | NA |
| OTU7260 | NA | NA | NA | NA |
| OTU13106 | NA | NA | NA | NA |
| OTU10062 | + | NA | NA | NA |
| OTU14314 | NA | NA | NA | NA |
| OTU5678 | NA | NA | NA | NA |
| OTU15500 | NA | NA | NA | NA |
| OTU4736 | NA | NA | NA | NA |
| OTU17046 | NA | NA | NA | NA |
| OTU11573 | NA | NA | NA | NA |
| OTU17197 | NA | NA | NA | NA |
| OTU15421 | NA | NA | NA | NA |
| OTU10112 | NA | NA | NA | NA |
| OTU3647 | NA | NA | NA | NA |
| OTU4927 | NA | NA | NA | NA |
| OTU6650 | + | NA | NA | NA |
| OTU17770 | NA | NA | NA | + |
| OTU17176 | NA | NA | NA | NA |
| OTU15207 | NA | NA | NA | NA |
| OTU18775 | NA | NA | NA | NA |
| OTU10991 | + | - | + | NA |
| OTU8398 | + | NA | NA | NA |
| OTU4299 | + | NA | NA | NA |
| OTU10405 | NA | NA | NA | - |
| OTU1509 | NA | NA | NA | NA |
| OTU8170 | + | NA | NA | NA |
| OTU6795 | + | NA | NA | NA |
| OTU15534 | NA | NA | NA | NA |
| OTU15659 | + | NA | NA | NA |
| OTU3085 | NA | NA | NA | NA |
| OTU5826 | NA | + | NA | NA |
| OTU11545 | NA | NA | NA | NA |
| OTU6563 | + | - | NA | NA |
| OTU10649 | NA | - | NA | + |
| OTU11598 | NA | NA | NA | NA |
| OTU16410 | NA | NA | NA | + |
| OTU2884 | + | NA | NA | NA |
| OTU5381 | NA | + | NA | NA |
| OTU13943 | NA | NA | NA | NA |
| OTU16151 | NA | NA | NA | NA |
| OTU18377 | + | NA | NA | NA |
| OTU7389 | NA | NA | NA | NA |
| OTU7697 | NA | NA | NA | NA |
| OTU9161 | NA | NA | NA | NA |
| OTU2871 | NA | NA | - | + |
| OTU4915 | NA | NA | NA | NA |
| OTU11524 | NA | NA | NA | NA |
| OTU12406 | + | NA | NA | NA |
| OTU5324 | NA | NA | NA | NA |
| OTU10520 | NA | NA | NA | NA |
| OTU16900 | + | NA | NA | NA |
| OTU2428 | NA | NA | NA | NA |
| OTU7394 | + | NA | NA | NA |
| OTU2214 | + | NA | NA | NA |
| OTU14538 | NA | NA | + | NA |
| OTU8243 | + | NA | NA | NA |
| OTU711 | + | NA | NA | NA |
| OTU17236 | NA | NA | NA | + |
| OTU4387 | NA | NA | NA | NA |
| OTU5094 | - | + | NA | NA |
| OTU17125 | NA | NA | NA | NA |
| OTU10641 | NA | NA | NA | NA |
| OTU6280 | NA | NA | NA | NA |
| OTU6490 | NA | + | NA | NA |
| OTU6138 | + | NA | NA | NA |
| OTU5654 | NA | NA | NA | NA |
| OTU1297 | NA | NA | NA | NA |
| OTU1272 | NA | NA | NA | NA |
| OTU2303 | + | NA | NA | NA |
| OTU5135 | NA | NA | NA | NA |
| OTU6360 | NA | NA | NA | NA |
| OTU37 | NA | NA | NA | NA |
| OTU7432 | NA | + | NA | NA |
| OTU7780 | + | NA | NA | NA |
| OTU17286 | + | NA | NA | NA |
| OTU18237 | NA | NA | NA | NA |
| OTU6729 | NA | NA | NA | NA |
| OTU3660 | NA | NA | NA | NA |
| OTU3139 | NA | NA | NA | NA |
| OTU3648 | NA | NA | + | - |
| OTU3812 | NA | NA | NA | NA |
| OTU12707 | NA | NA | NA | NA |
| OTU8684 | + | NA | NA | NA |
| OTU16545 | NA | NA | NA | NA |
| OTU8471 | NA | NA | NA | NA |
| OTU16956 | + | NA | NA | NA |
| OTU8231 | + | NA | NA | NA |
| OTU5792 | NA | NA | NA | NA |
| OTU8476 | NA | NA | NA | NA |
| OTU2487 | + | NA | NA | NA |
| OTU7570 | NA | NA | NA | + |
| OTU6969 | NA | + | NA | NA |
| OTU11446 | NA | NA | NA | NA |
| OTU10648 | NA | NA | NA | NA |
| OTU6936 | NA | NA | NA | NA |
| OTU10020 | NA | - | NA | + |
| OTU4965 | NA | NA | NA | NA |
| OTU5079 | NA | NA | NA | NA |
| OTU14000 | NA | NA | + | NA |
| OTU16984 | NA | NA | NA | + |
| OTU17185 | NA | NA | - | NA |
| OTU3075 | + | NA | NA | NA |
| OTU6477 | + | NA | NA | NA |
| OTU8037 | + | NA | NA | NA |
| OTU15873 | NA | NA | NA | NA |
| OTU17394 | NA | NA | NA | NA |
| OTU16959 | + | NA | NA | NA |
| OTU15862 | NA | NA | NA | + |
| OTU16896 | + | NA | NA | NA |
| OTU113 | NA | NA | NA | NA |
| OTU9863 | NA | NA | NA | NA |
| OTU9271 | NA | NA | NA | NA |
| OTU649 | NA | NA | NA | NA |
| OTU15875 | NA | NA | NA | NA |
| OTU9874 | NA | NA | NA | NA |
| OTU10196 | NA | NA | NA | + |
| OTU13448 | NA | NA | NA | NA |
| OTU1733 | NA | NA | NA | NA |
| OTU8892 | NA | NA | NA | NA |
| OTU11345 | NA | NA | NA | - |
| OTU12940 | NA | NA | NA | NA |
| OTU13708 | NA | NA | NA | NA |
| OTU12744 | + | - | NA | NA |
| OTU14886 | NA | NA | NA | NA |
| OTU10251 | NA | NA | + | NA |
| OTU6718 | NA | NA | NA | NA |
| OTU13618 | NA | NA | NA | NA |
| OTU7094 | NA | NA | NA | NA |
| OTU7212 | NA | NA | NA | NA |
| OTU3351 | NA | NA | NA | NA |
| OTU10475 | NA | NA | NA | NA |
| OTU11587 | NA | NA | NA | NA |
| OTU8089 | + | NA | NA | NA |
| OTU5327 | NA | NA | NA | NA |
| OTU14452 | NA | NA | NA | NA |
| OTU14037 | NA | NA | NA | NA |
| OTU1790 | NA | NA | NA | NA |
| OTU8112 | NA | NA | NA | NA |
| OTU3687 | NA | NA | NA | NA |
| OTU1120 | + | NA | NA | NA |
| OTU6203 | + | NA | NA | NA |
| OTU5494 | NA | NA | NA | NA |
| OTU9048 | + | NA | NA | NA |
| OTU4581 | NA | NA | NA | NA |
| OTU11412 | + | - | + | - |
| OTU14172 | NA | NA | NA | NA |
| OTU8026 | NA | NA | NA | NA |
| OTU11709 | + | NA | NA | NA |
| OTU18713 | NA | NA | NA | + |
| OTU1324 | NA | NA | NA | - |
| OTU17152 | NA | NA | NA | NA |
| OTU8075 | + | NA | NA | NA |
| OTU1774 | NA | NA | + | NA |
| OTU18834 | NA | NA | NA | + |
| OTU12996 | NA | + | NA | NA |
| OTU1383 | + | NA | NA | NA |
| OTU16622 | NA | NA | NA | NA |
| OTU8586 | NA | NA | NA | NA |
| OTU5525 | NA | NA | NA | NA |
| OTU5271 | NA | NA | NA | NA |
| OTU17091 | NA | NA | NA | NA |
| OTU15254 | NA | NA | NA | + |
| OTU16781 | NA | NA | NA | NA |
| OTU1550 | NA | NA | - | NA |
| OTU12292 | NA | NA | NA | NA |
| OTU13416 | NA | NA | NA | NA |
| OTU18783 | + | NA | NA | NA |
| OTU12895 | NA | NA | + | NA |
| OTU8699 | NA | NA | NA | NA |
| OTU6269 | NA | NA | NA | NA |
| OTU8096 | NA | - | NA | NA |
| OTU12618 | NA | NA | NA | NA |
| OTU16473 | NA | NA | NA | NA |
| OTU1224 | + | - | NA | NA |
| OTU1609 | + | NA | NA | NA |
| OTU18744 | NA | NA | NA | + |
| OTU16498 | NA | NA | NA | NA |
| OTU9942 | NA | NA | NA | NA |
| OTU18247 | NA | NA | NA | NA |
| OTU15759 | + | NA | NA | NA |
| OTU8689 | NA | NA | NA | NA |
| OTU18010 | NA | NA | NA | + |
| OTU1661 | NA | NA | NA | NA |
| OTU440 | NA | NA | NA | + |
| OTU16813 | NA | NA | NA | NA |
| OTU1160 | + | NA | NA | NA |
| OTU10909 | NA | NA | + | NA |
| OTU17179 | NA | NA | + | NA |
| OTU6090 | NA | NA | NA | NA |
| OTU10000 | NA | NA | NA | NA |
| OTU11702 | + | NA | NA | NA |
| OTU12665 | NA | NA | NA | NA |
| OTU16301 | NA | NA | NA | NA |
| OTU14975 | + | - | NA | NA |
| OTU15148 | NA | - | NA | + |
| OTU1094 | NA | NA | NA | NA |
| OTU6776 | NA | NA | NA | NA |
| OTU4553 | NA | NA | NA | NA |
| OTU14323 | NA | - | NA | NA |
| OTU11000 | NA | NA | NA | NA |
| OTU18257 | NA | NA | NA | NA |
| OTU9484 | NA | NA | NA | NA |
| OTU14512 | + | NA | NA | NA |
| OTU4887 | NA | NA | NA | NA |
| OTU15209 | NA | NA | NA | + |
| OTU7399 | NA | NA | NA | NA |
| OTU11905 | NA | NA | NA | + |
| OTU12482 | NA | NA | NA | NA |
| OTU10499 | NA | NA | NA | NA |
| OTU7280 | NA | NA | NA | NA |
| OTU5855 | NA | NA | NA | NA |
| OTU1981 | NA | NA | NA | + |
| OTU14195 | NA | NA | + | NA |
| OTU3614 | NA | NA | NA | + |
| OTU13502 | NA | NA | NA | NA |
| OTU13450 | + | NA | NA | NA |
| OTU7112 | NA | + | NA | NA |
| OTU4382 | + | NA | - | NA |
| OTU10352 | NA | + | NA | NA |
| OTU1439 | NA | NA | NA | NA |
| OTU16403 | + | NA | NA | NA |
| OTU18689 | NA | + | NA | NA |
| OTU7239 | NA | NA | NA | NA |
| OTU1921 | NA | NA | NA | NA |
| OTU15560 | + | NA | NA | NA |
| OTU13432 | NA | NA | NA | + |
| OTU16371 | NA | NA | NA | NA |
| OTU18711 | + | NA | NA | NA |
| OTU6661 | NA | NA | NA | NA |
| OTU407 | + | NA | NA | NA |
| OTU421 | NA | NA | + | NA |
| OTU15187 | NA | NA | NA | NA |
| OTU5754 | NA | NA | NA | NA |
| OTU18682 | NA | NA | NA | NA |
| OTU12841 | NA | - | + | NA |
| OTU8403 | NA | NA | NA | NA |
| OTU7745 | NA | NA | NA | NA |
| OTU2793 | NA | NA | NA | NA |
| OTU2697 | + | NA | NA | NA |
| OTU5057 | NA | NA | NA | NA |
| OTU11536 | NA | NA | NA | NA |
| OTU7642 | NA | NA | NA | NA |
| OTU2483 | NA | + | NA | NA |
| OTU18755 | + | NA | NA | NA |
| OTU12782 | NA | NA | NA | NA |
| OTU5618 | NA | NA | + | NA |
| OTU10426 | NA | NA | NA | NA |
| OTU13354 | NA | NA | NA | NA |
| OTU9368 | NA | + | NA | NA |
| OTU1585 | + | NA | NA | NA |
| OTU16973 | NA | NA | NA | + |
| OTU8563 | NA | NA | NA | NA |
| OTU1594 | NA | NA | NA | NA |
| OTU18868 | NA | NA | NA | NA |
| OTU10985 | NA | NA | NA | NA |
| OTU7197 | NA | NA | NA | NA |
| OTU4072 | NA | NA | NA | NA |
| OTU9039 | NA | - | NA | NA |
| OTU14884 | NA | NA | NA | NA |
| OTU4391 | NA | NA | NA | NA |
| OTU6893 | NA | NA | NA | NA |
| OTU6881 | NA | + | NA | NA |
| OTU5427 | NA | NA | + | NA |
| OTU15859 | + | - | NA | NA |
| OTU10622 | NA | NA | NA | NA |
| OTU14374 | + | NA | NA | NA |
| OTU7900 | + | NA | NA | NA |
| OTU333 | NA | NA | NA | NA |
| OTU68 | NA | + | NA | NA |
| OTU6175 | NA | NA | NA | NA |
| OTU11594 | + | NA | NA | NA |
| OTU3771 | NA | NA | NA | NA |
| OTU888 | NA | + | NA | NA |
| OTU4125 | NA | NA | NA | NA |
| OTU11443 | NA | NA | NA | - |
| OTU11927 | NA | NA | NA | NA |
| OTU9117 | NA | NA | NA | NA |
| OTU4350 | + | NA | + | - |
| OTU11328 | NA | NA | + | NA |
| OTU8193 | NA | NA | NA | NA |
| OTU8710 | + | NA | - | NA |
| OTU5586 | NA | NA | NA | + |
| OTU13311 | NA | NA | + | NA |
| OTU13414 | NA | NA | NA | NA |
| OTU11498 | NA | NA | + | NA |
| OTU6938 | NA | NA | NA | NA |
| OTU16060 | NA | NA | NA | NA |
| OTU18978 | NA | NA | NA | NA |
| OTU6395 | NA | NA | NA | NA |
| OTU12808 | NA | NA | NA | NA |
| OTU8938 | NA | NA | NA | NA |
| OTU18343 | NA | NA | + | NA |
| OTU7706 | NA | NA | NA | NA |
| OTU2165 | + | NA | NA | NA |
| OTU18094 | NA | NA | NA | + |
| OTU7371 | NA | NA | NA | NA |
| OTU16456 | NA | NA | NA | NA |
| OTU15323 | NA | NA | NA | NA |
| OTU6250 | NA | NA | NA | NA |
| OTU15831 | NA | NA | NA | NA |
| OTU18471 | NA | NA | NA | NA |
| OTU6754 | + | NA | NA | NA |
| OTU7702 | NA | NA | NA | NA |
| OTU1484 | NA | + | - | NA |
| OTU6312 | NA | + | NA | - |
| OTU6148 | NA | NA | NA | NA |
| OTU664 | NA | NA | NA | NA |
| OTU6638 | NA | NA | NA | NA |
| OTU1159 | NA | NA | NA | NA |
| OTU16465 | + | NA | NA | NA |
| OTU16190 | + | NA | NA | NA |
| OTU8996 | NA | NA | NA | NA |
| OTU12811 | NA | NA | NA | NA |
| OTU16708 | NA | NA | NA | NA |
| OTU15186 | NA | NA | NA | NA |
| OTU2110 | + | NA | NA | NA |
| OTU4911 | NA | NA | NA | NA |
| OTU9876 | NA | NA | NA | NA |
| OTU7138 | NA | NA | NA | NA |
| OTU295 | NA | NA | NA | NA |
| OTU16730 | + | NA | NA | NA |
| OTU18520 | NA | NA | NA | NA |
| OTU2024 | NA | NA | NA | NA |
| OTU17477 | NA | NA | NA | NA |
| OTU11326 | + | NA | NA | NA |
| OTU7393 | NA | + | NA | NA |
| OTU2003 | NA | NA | NA | NA |
| OTU8021 | NA | NA | NA | NA |
| OTU13870 | NA | NA | NA | NA |
| OTU12130 | NA | NA | NA | NA |
| OTU16476 | NA | NA | NA | NA |
| OTU3673 | + | NA | NA | NA |
| OTU9908 | NA | NA | NA | NA |
| OTU7275 | NA | NA | NA | NA |
| OTU15420 | NA | NA | NA | NA |
| OTU9087 | NA | + | - | NA |
| OTU9824 | NA | NA | NA | NA |
| OTU9603 | NA | NA | NA | NA |
| OTU5749 | NA | NA | NA | NA |
| OTU4104 | NA | NA | NA | NA |
| OTU1426 | NA | NA | NA | NA |
| OTU46 | + | NA | - | NA |
| OTU7245 | NA | NA | NA | NA |
| OTU14170 | NA | NA | NA | NA |
| OTU1216 | NA | NA | NA | NA |
| OTU6532 | NA | NA | NA | NA |
| OTU3788 | NA | NA | NA | NA |
| OTU15403 | NA | NA | NA | + |
| OTU18269 | NA | NA | NA | NA |
| OTU13225 | + | - | NA | NA |
| OTU17456 | NA | NA | NA | NA |
| OTU15468 | NA | NA | NA | NA |
| OTU12500 | + | NA | NA | NA |
| OTU5847 | NA | NA | NA | NA |
| OTU136 | NA | NA | NA | NA |
| OTU967 | NA | NA | NA | NA |
| OTU9933 | NA | NA | NA | NA |
| OTU13475 | + | - | + | - |
| OTU17895 | NA | NA | NA | NA |
| OTU10899 | + | NA | NA | NA |
| OTU5205 | NA | NA | NA | NA |
| OTU12812 | NA | NA | + | NA |
| OTU15784 | + | NA | NA | NA |
| OTU7041 | NA | NA | NA | NA |
| OTU2365 | + | NA | NA | NA |
| OTU11436 | NA | NA | NA | NA |
| OTU17386 | NA | + | NA | NA |
| OTU15934 | NA | NA | - | NA |
| OTU1662 | NA | NA | + | NA |
| OTU10647 | NA | NA | NA | NA |
| OTU16090 | NA | NA | NA | NA |
| OTU4486 | + | NA | NA | NA |
| OTU7551 | NA | NA | NA | NA |
| OTU6212 | NA | NA | + | NA |
| OTU17525 | NA | NA | NA | + |
| OTU9028 | NA | NA | NA | NA |
| OTU8457 | + | NA | NA | NA |
| OTU17082 | NA | NA | NA | NA |
| OTU9844 | NA | NA | NA | NA |
| OTU1684 | NA | NA | NA | NA |
| OTU40 | + | NA | NA | NA |
| OTU15786 | + | NA | NA | NA |
| OTU10233 | NA | NA | + | NA |
| OTU10655 | - | NA | NA | NA |
| OTU10733 | NA | NA | + | NA |
| OTU3196 | NA | NA | NA | NA |
| OTU11938 | NA | NA | NA | NA |
| OTU16314 | NA | NA | NA | NA |
| OTU9081 | NA | NA | NA | NA |
| OTU9119 | NA | NA | NA | NA |
| OTU8413 | NA | NA | NA | NA |
| OTU11325 | NA | - | NA | NA |
| OTU2666 | + | NA | NA | NA |
| OTU17154 | NA | NA | NA | NA |
| OTU6579 | NA | NA | NA | NA |
| OTU9962 | NA | - | + | NA |
| OTU14127 | NA | NA | NA | - |
| OTU9435 | + | NA | NA | NA |
| OTU5842 | NA | NA | NA | NA |
| OTU10897 | NA | NA | NA | NA |
| OTU18322 | NA | NA | NA | NA |
| OTU5060 | NA | NA | NA | NA |
| OTU16810 | NA | NA | NA | NA |
| OTU19102 | NA | NA | + | NA |
| OTU12243 | NA | NA | NA | NA |
| OTU9263 | NA | NA | NA | NA |
| OTU10976 | NA | NA | NA | NA |
| OTU15571 | NA | NA | NA | NA |
| OTU18681 | NA | NA | NA | NA |
| OTU7744 | NA | NA | NA | NA |
| OTU211 | NA | NA | NA | NA |
| OTU8013 | NA | NA | NA | NA |
| OTU4158 | NA | NA | NA | NA |
| OTU14995 | NA | NA | NA | NA |
| OTU12265 | NA | NA | NA | NA |
| OTU16931 | NA | NA | NA | NA |
| OTU18762 | NA | NA | NA | NA |
| OTU17463 | NA | NA | NA | NA |
| OTU7064 | NA | NA | + | NA |
| OTU8924 | NA | NA | NA | NA |
| OTU3503 | + | NA | NA | NA |
| OTU3702 | + | - | NA | NA |
| OTU12006 | NA | NA | NA | NA |
| OTU15492 | NA | NA | NA | + |
| OTU2613 | + | NA | NA | NA |
| OTU12882 | NA | NA | NA | NA |
| OTU4604 | NA | NA | NA | NA |
| OTU1448 | NA | NA | NA | NA |
| OTU2047 | NA | NA | NA | NA |
| OTU11445 | NA | NA | NA | NA |
| OTU14298 | NA | NA | NA | NA |
| OTU16341 | + | - | NA | NA |
| OTU6019 | NA | NA | NA | NA |
| OTU9176 | NA | NA | NA | NA |
| OTU7453 | NA | NA | NA | NA |
| OTU2008 | NA | NA | NA | NA |
| OTU8795 | + | NA | NA | NA |
| OTU418 | + | NA | NA | NA |
| OTU4129 | NA | NA | NA | NA |
| OTU5561 | NA | NA | NA | NA |
| OTU9884 | NA | NA | NA | NA |
| OTU5788 | NA | + | NA | NA |
| OTU17207 | NA | NA | NA | NA |
| OTU9355 | NA | NA | NA | NA |
| OTU16393 | NA | NA | NA | NA |
| OTU14193 | NA | NA | NA | NA |
| OTU4885 | NA | NA | NA | NA |
| OTU8248 | + | NA | NA | NA |
| OTU18837 | NA | NA | NA | + |
| OTU13518 | NA | NA | NA | NA |
| OTU5145 | NA | NA | NA | NA |
| OTU11751 | NA | NA | NA | - |
| OTU11438 | NA | NA | NA | NA |
| OTU11155 | NA | NA | + | - |
| OTU17980 | NA | NA | NA | + |
| OTU4257 | NA | + | NA | NA |
| OTU2403 | + | NA | NA | NA |
| OTU1443 | NA | NA | NA | NA |
| OTU10081 | NA | NA | NA | NA |
| OTU16627 | NA | NA | NA | NA |
| OTU4845 | NA | NA | NA | - |
| OTU60 | NA | NA | NA | + |
| OTU359 | + | NA | NA | NA |
| OTU4773 | NA | NA | NA | NA |
| OTU4251 | NA | + | NA | NA |
| OTU4698 | NA | NA | + | NA |
| OTU6674 | NA | NA | NA | NA |
| OTU4084 | NA | NA | NA | NA |
| OTU1569 | NA | NA | NA | NA |
| OTU2729 | NA | NA | NA | NA |
| OTU13758 | NA | NA | NA | NA |
| OTU1070 | NA | NA | NA | NA |
| OTU13381 | NA | NA | NA | NA |
| OTU2376 | + | NA | NA | NA |
| OTU561 | NA | NA | NA | NA |
| OTU14199 | NA | NA | NA | NA |
| OTU8238 | NA | NA | NA | NA |
| OTU4184 | NA | NA | NA | NA |
| OTU6184 | NA | NA | NA | NA |
| OTU6369 | NA | + | NA | NA |
| OTU14202 | NA | NA | NA | - |
| OTU8681 | NA | NA | NA | NA |
| OTU10317 | NA | NA | NA | + |
| OTU19048 | NA | NA | NA | NA |
| OTU5658 | NA | NA | NA | NA |
| OTU15597 | NA | NA | NA | NA |
| OTU16463 | NA | NA | NA | + |
| OTU7329 | NA | NA | NA | NA |
| OTU12415 | NA | NA | NA | NA |
| OTU3289 | NA | NA | NA | NA |
| OTU3301 | NA | NA | NA | NA |
| OTU5211 | NA | NA | NA | NA |
| OTU17012 | NA | NA | NA | + |
| OTU17237 | NA | NA | NA | NA |
| OTU9111 | NA | NA | NA | NA |
| OTU17104 | NA | NA | NA | + |
| OTU18815 | NA | + | NA | NA |
| OTU1363 | + | NA | NA | NA |
| OTU101 | NA | NA | NA | NA |
| OTU9882 | NA | NA | NA | NA |
| OTU14305 | NA | NA | NA | - |
| OTU14461 | NA | NA | NA | NA |
| OTU5601 | NA | NA | NA | NA |
| OTU18252 | NA | NA | NA | + |
| OTU83 | NA | NA | NA | NA |
| OTU5927 | NA | NA | NA | NA |
| OTU9954 | NA | NA | + | - |
| OTU2107 | + | NA | NA | NA |
| OTU5653 | NA | NA | NA | NA |
| OTU762 | NA | NA | NA | NA |
| OTU12651 | + | NA | NA | NA |
| OTU5435 | NA | NA | NA | NA |
| OTU10701 | NA | NA | NA | NA |
| OTU3274 | + | NA | NA | NA |
| OTU59 | + | NA | NA | NA |
| OTU15014 | NA | NA | NA | NA |
| OTU6696 | + | NA | NA | NA |
| OTU7305 | NA | NA | NA | NA |
| OTU16965 | NA | NA | NA | NA |
| OTU2050 | + | - | + | NA |
| OTU15113 | NA | NA | NA | NA |
| OTU1006 | + | NA | NA | NA |
| OTU10276 | NA | NA | NA | + |
| OTU13800 | + | - | NA | NA |
| OTU165 | NA | NA | NA | NA |
| OTU4011 | NA | NA | NA | - |
| OTU8879 | NA | NA | NA | NA |
| OTU6413 | NA | NA | NA | NA |
| OTU3724 | + | - | NA | NA |
| OTU11381 | NA | NA | NA | NA |
| OTU8101 | NA | NA | NA | NA |
| OTU9394 | NA | NA | NA | - |
| OTU13248 | NA | NA | NA | NA |
| OTU2476 | + | NA | NA | NA |
| OTU16954 | + | NA | NA | NA |
| OTU14724 | NA | NA | NA | NA |
| OTU2077 | NA | NA | NA | NA |
| OTU19085 | NA | - | NA | - |
| OTU69 | NA | NA | NA | NA |
| OTU12934 | NA | NA | NA | NA |
| OTU5293 | NA | NA | NA | NA |
| OTU5369 | NA | + | NA | NA |
| OTU9319 | NA | NA | - | NA |
| OTU7882 | NA | NA | NA | NA |
| OTU10990 | NA | - | + | NA |
| OTU4425 | NA | NA | + | - |
| OTU5170 | NA | NA | NA | NA |
| OTU18229 | NA | NA | - | + |
| OTU16741 | NA | NA | NA | NA |
| OTU5165 | NA | NA | NA | NA |
| OTU17455 | NA | NA | NA | NA |
| OTU2288 | NA | NA | NA | NA |
| OTU18585 | NA | NA | NA | NA |
| OTU1949 | NA | NA | NA | NA |
| OTU3580 | NA | NA | NA | NA |
| OTU17282 | NA | NA | NA | + |
| OTU1286 | NA | NA | NA | NA |
| OTU6597 | NA | NA | NA | NA |
| OTU14165 | + | - | NA | NA |
| OTU8063 | + | NA | NA | NA |
| OTU7289 | NA | NA | NA | NA |
| OTU17013 | NA | NA | NA | NA |
| OTU8209 | + | NA | NA | NA |
| OTU7659 | NA | NA | NA | NA |
| OTU3380 | NA | NA | NA | NA |
| OTU18501 | NA | NA | NA | + |
| OTU11772 | NA | NA | NA | NA |
| OTU11025 | NA | NA | + | - |
| OTU18200 | NA | NA | NA | NA |
| OTU4123 | NA | + | NA | NA |
| OTU10073 | NA | NA | NA | - |
| OTU844 | + | NA | NA | NA |
| OTU1156 | + | - | NA | NA |
| OTU4318 | NA | NA | NA | NA |
| OTU1758 | NA | NA | NA | NA |
| OTU17027 | NA | - | NA | NA |
| OTU1920 | NA | NA | NA | + |
| OTU9037 | NA | NA | NA | NA |
| OTU17066 | NA | NA | NA | + |
| OTU8747 | NA | NA | - | NA |
| OTU3044 | NA | NA | NA | NA |
| OTU16499 | NA | NA | NA | NA |
| OTU7022 | NA | NA | - | NA |
| OTU18278 | NA | NA | NA | NA |
| OTU10021 | NA | NA | NA | NA |
| OTU7543 | NA | NA | NA | NA |
| OTU3901 | NA | NA | NA | NA |
| OTU1143 | NA | NA | NA | NA |
| OTU18810 | NA | NA | NA | + |
| OTU12542 | NA | - | NA | NA |
| OTU8838 | + | NA | NA | NA |
| OTU1168 | NA | NA | NA | - |
| OTU14272 | NA | NA | + | NA |
| OTU6305 | NA | NA | NA | NA |
| OTU9322 | NA | NA | + | NA |
| OTU17414 | NA | NA | NA | NA |
| OTU308 | + | - | NA | NA |
| OTU9990 | NA | NA | NA | NA |
| OTU15540 | NA | NA | NA | NA |
| OTU4700 | NA | NA | NA | NA |
| OTU4835 | NA | + | - | NA |
| OTU349 | + | NA | NA | NA |
| OTU14250 | NA | NA | NA | NA |
| OTU4724 | NA | NA | NA | NA |
| OTU16651 | + | NA | NA | NA |
| OTU14989 | NA | NA | NA | NA |
| OTU9378 | NA | NA | NA | NA |
| OTU9040 | + | NA | NA | NA |
| OTU6084 | NA | NA | NA | NA |
| OTU11741 | NA | NA | NA | NA |
| OTU8463 | NA | NA | NA | NA |
| OTU18173 | NA | NA | NA | + |
| OTU1589 | + | NA | NA | NA |
| OTU2289 | NA | NA | NA | NA |
| OTU6158 | NA | NA | + | NA |
| OTU5607 | NA | NA | NA | NA |
| OTU6531 | NA | - | NA | NA |
| OTU10304 | + | NA | NA | NA |
| OTU697 | + | NA | NA | NA |
| OTU2426 | + | NA | NA | NA |
| OTU7538 | + | NA | NA | NA |
| OTU18789 | NA | NA | NA | NA |
| OTU16171 | NA | NA | NA | NA |
| OTU15981 | NA | NA | NA | NA |
| OTU14943 | NA | NA | NA | - |
| OTU17243 | NA | NA | NA | NA |
| OTU6238 | NA | NA | + | NA |
| OTU14315 | NA | NA | NA | NA |
| OTU11482 | NA | NA | NA | NA |
| OTU4143 | NA | + | NA | NA |
| OTU12562 | NA | NA | NA | NA |
| OTU16581 | NA | NA | NA | NA |
| OTU1288 | NA | NA | NA | NA |
| OTU4703 | NA | NA | NA | NA |
| OTU15410 | NA | NA | NA | NA |
| OTU1991 | NA | NA | NA | + |
| OTU9054 | NA | NA | NA | - |
| OTU9011 | NA | NA | NA | NA |
| OTU15487 | NA | NA | NA | NA |
| OTU16391 | NA | NA | NA | + |
| OTU11033 | + | - | NA | NA |
| OTU15818 | NA | NA | NA | NA |
| OTU1311 | + | NA | NA | NA |
| OTU18650 | NA | NA | - | NA |
| OTU5605 | + | - | NA | NA |
| OTU16336 | NA | NA | NA | NA |
| OTU6252 | NA | NA | NA | NA |
| OTU17298 | NA | NA | NA | NA |
| OTU12395 | + | - | NA | NA |
| OTU1055 | + | NA | NA | NA |
| OTU15213 | NA | NA | NA | + |
| OTU4622 | NA | + | NA | NA |
| OTU8414 | NA | NA | NA | NA |
| OTU5124 | NA | NA | + | NA |
| OTU4457 | NA | NA | + | NA |
| OTU1928 | NA | NA | NA | NA |
| OTU12732 | NA | NA | NA | NA |
| OTU7020 | NA | + | NA | NA |
| OTU6723 | + | NA | NA | NA |
| OTU16036 | NA | - | NA | NA |
| OTU4291 | + | NA | NA | NA |
| OTU16757 | NA | NA | NA | NA |
| OTU4498 | NA | NA | NA | NA |
| OTU43 | NA | NA | NA | NA |
| OTU1570 | NA | NA | NA | NA |
| OTU2283 | NA | NA | NA | NA |
| OTU4852 | NA | NA | NA | NA |
| OTU4015 | NA | NA | NA | NA |
| OTU1085 | NA | NA | NA | NA |
| OTU15071 | NA | NA | NA | NA |
| OTU2794 | NA | - | NA | NA |
| OTU3038 | NA | NA | NA | NA |
| OTU13649 | NA | NA | NA | NA |
| OTU14913 | NA | NA | + | NA |
| OTU1336 | NA | NA | NA | NA |
| OTU6684 | NA | NA | NA | NA |
| OTU1719 | NA | NA | NA | NA |
| OTU5651 | NA | NA | NA | NA |
| OTU8744 | NA | NA | NA | NA |
| OTU5419 | NA | NA | NA | NA |
| OTU4904 | NA | NA | NA | NA |
| OTU18807 | + | NA | NA | NA |
| OTU17331 | + | NA | NA | NA |
| OTU13677 | NA | NA | NA | NA |
| OTU4963 | NA | NA | NA | NA |
| OTU2801 | NA | NA | NA | NA |
| OTU3943 | NA | NA | NA | NA |
| OTU15225 | NA | NA | NA | + |
| OTU8346 | NA | NA | NA | NA |
| OTU3426 | NA | NA | NA | NA |
| OTU1233 | + | NA | NA | NA |
| OTU4219 | NA | NA | NA | NA |
| OTU4026 | NA | NA | NA | - |
| OTU4451 | NA | NA | NA | NA |
| OTU7162 | NA | NA | NA | NA |
| OTU7332 | NA | NA | NA | NA |
| OTU9255 | NA | NA | NA | NA |
| OTU11315 | NA | NA | NA | NA |
| OTU15905 | NA | NA | NA | NA |
| OTU2625 | + | NA | NA | NA |
| OTU15383 | NA | - | NA | NA |
| OTU16066 | NA | NA | NA | NA |
| OTU800 | + | NA | NA | NA |
| OTU12613 | NA | NA | NA | NA |
| OTU8791 | + | NA | NA | NA |
| OTU4948 | NA | NA | NA | NA |
| OTU3561 | NA | NA | NA | NA |
| OTU3286 | NA | NA | NA | NA |
| OTU14990 | NA | NA | + | NA |
| OTU4076 | NA | NA | NA | NA |
| OTU12444 | NA | NA | NA | NA |
| OTU6464 | + | NA | NA | NA |
| OTU5348 | NA | NA | NA | NA |
| OTU12742 | NA | NA | + | NA |
| OTU4378 | NA | NA | NA | NA |
| OTU8071 | NA | NA | NA | NA |
| OTU1432 | + | NA | NA | NA |
| OTU6850 | NA | NA | NA | NA |
| OTU17641 | NA | NA | NA | NA |
| OTU14655 | NA | NA | + | NA |
| OTU10356 | NA | NA | NA | NA |
| OTU3816 | + | - | NA | NA |
| OTU280 | + | NA | NA | NA |
| OTU5577 | NA | + | NA | NA |
| OTU4017 | NA | NA | NA | NA |
| OTU8062 | + | NA | NA | NA |
| OTU15154 | NA | - | NA | NA |
| OTU775 | NA | NA | NA | NA |
| OTU1706 | NA | - | NA | NA |
| OTU12643 | NA | NA | + | NA |
| OTU12351 | NA | NA | NA | NA |
| OTU17052 | NA | NA | NA | NA |
| OTU8815 | NA | NA | NA | NA |
| OTU2414 | + | NA | NA | NA |
| OTU17956 | NA | NA | NA | NA |
| OTU9091 | + | NA | NA | NA |
| OTU16636 | NA | NA | NA | NA |
| OTU14185 | NA | NA | + | - |
| OTU12923 | NA | NA | NA | NA |
| OTU2867 | + | NA | NA | NA |
| OTU8847 | NA | NA | NA | - |
| OTU12994 | + | NA | NA | NA |
| OTU2023 | NA | NA | NA | NA |
| OTU9049 | + | NA | NA | NA |
| OTU5404 | NA | + | NA | NA |
| OTU16436 | NA | NA | NA | NA |
| OTU11108 | NA | NA | NA | NA |
| OTU3408 | + | NA | NA | NA |
| OTU4554 | NA | NA | NA | NA |
| OTU5314 | NA | NA | NA | NA |
| OTU12616 | NA | NA | + | NA |
| OTU16101 | NA | NA | - | NA |
| OTU4985 | NA | NA | NA | NA |
| OTU2362 | NA | NA | NA | NA |
| OTU18163 | NA | NA | NA | NA |
| OTU16507 | + | NA | NA | NA |
| OTU8264 | NA | NA | NA | NA |
| OTU2685 | NA | NA | NA | NA |
| OTU944 | NA | NA | NA | NA |
| OTU12529 | NA | NA | NA | NA |
| OTU13694 | NA | NA | NA | NA |
| OTU15891 | + | NA | NA | NA |
| OTU1365 | NA | NA | NA | NA |
| OTU10532 | NA | NA | + | NA |
| OTU536 | NA | NA | NA | NA |
| OTU6623 | NA | NA | NA | NA |
| OTU15236 | NA | NA | NA | NA |
| OTU3494 | NA | NA | NA | NA |
| OTU15489 | + | - | NA | NA |
| OTU11957 | NA | NA | NA | NA |
| OTU10888 | NA | NA | NA | NA |
| OTU10719 | NA | NA | NA | NA |
| OTU8472 | NA | NA | NA | - |
| OTU1527 | NA | NA | NA | NA |
| OTU1018 | + | NA | NA | NA |
| OTU11972 | NA | NA | NA | NA |
| OTU16277 | NA | NA | NA | NA |
| OTU5614 | NA | NA | NA | NA |
| OTU13607 | NA | NA | NA | NA |
| OTU18184 | + | NA | NA | NA |
| OTU8741 | NA | NA | NA | NA |
| OTU10239 | NA | NA | NA | NA |
| OTU12345 | NA | NA | NA | NA |
| OTU8237 | NA | NA | - | NA |
| OTU15609 | + | NA | NA | NA |
| OTU2000 | NA | NA | NA | NA |
| OTU11595 | NA | NA | NA | NA |
| OTU12171 | NA | NA | NA | NA |
| OTU11066 | NA | NA | NA | NA |
| OTU8498 | NA | NA | NA | + |
| OTU16428 | NA | NA | NA | NA |
| OTU10543 | NA | NA | + | NA |
| OTU7528 | NA | NA | NA | NA |
| OTU1345 | NA | NA | NA | NA |
| OTU8258 | NA | NA | NA | NA |
| OTU5581 | NA | NA | NA | NA |
| OTU1791 | NA | NA | NA | + |
| OTU6707 | NA | NA | NA | + |
| OTU956 | NA | NA | NA | NA |
| OTU16989 | NA | NA | NA | NA |
| OTU1151 | NA | NA | NA | NA |
| OTU16970 | NA | NA | NA | NA |
| OTU8874 | NA | NA | NA | NA |
| OTU4758 | NA | NA | NA | NA |
| OTU7989 | + | NA | NA | NA |
| OTU3454 | NA | NA | NA | + |
| OTU15426 | NA | NA | NA | NA |
| OTU10357 | NA | - | NA | NA |
| OTU3425 | + | NA | NA | NA |
| OTU14604 | + | NA | NA | NA |
| OTU2275 | NA | NA | NA | NA |
| OTU2869 | NA | NA | NA | NA |
| OTU3037 | NA | NA | NA | NA |
| OTU2227 | NA | NA | NA | NA |
| OTU17081 | NA | NA | NA | + |
| OTU6474 | NA | + | NA | NA |
| OTU405 | NA | NA | NA | NA |
| OTU10399 | NA | NA | NA | NA |
| OTU14747 | NA | NA | NA | NA |
| OTU9885 | NA | NA | NA | NA |
| OTU13245 | NA | NA | NA | NA |
| OTU18799 | NA | NA | NA | + |
| OTU6469 | NA | NA | NA | NA |
| OTU15073 | NA | + | NA | NA |
| OTU2446 | + | NA | NA | NA |
| OTU4312 | NA | NA | NA | NA |
| OTU16788 | NA | NA | NA | NA |
| OTU13700 | NA | NA | NA | NA |
| OTU3165 | + | NA | NA | NA |
| OTU14213 | NA | NA | NA | NA |
| OTU1011 | NA | NA | NA | NA |
| OTU12414 | + | NA | NA | NA |
| OTU7850 | NA | NA | NA | NA |
| OTU12582 | NA | NA | NA | - |
| OTU11571 | NA | NA | NA | + |
| OTU193 | NA | NA | NA | NA |
| OTU845 | + | - | NA | NA |
| OTU16385 | + | NA | NA | NA |
| OTU10664 | NA | NA | NA | NA |
| OTU12118 | NA | NA | NA | NA |
| OTU9035 | NA | NA | NA | NA |
| OTU8517 | NA | NA | NA | NA |
| OTU9409 | NA | NA | NA | NA |
| OTU98 | NA | NA | + | NA |
| OTU15162 | NA | NA | NA | NA |
| OTU2244 | NA | NA | NA | NA |
| OTU7701 | + | NA | NA | NA |
| OTU14476 | NA | NA | NA | NA |
| OTU11091 | NA | NA | NA | NA |
| OTU13691 | NA | NA | NA | NA |
| OTU3236 | NA | NA | NA | NA |
| OTU8767 | NA | + | NA | NA |
| OTU10706 | NA | NA | NA | NA |
| OTU13719 | NA | NA | NA | NA |
| OTU2271 | NA | NA | NA | NA |
| OTU12918 | NA | NA | NA | NA |
| OTU12062 | NA | + | NA | NA |
| OTU17175 | NA | NA | NA | NA |
| OTU18444 | NA | NA | NA | NA |
| OTU10920 | NA | NA | NA | + |
| OTU12497 | NA | NA | NA | NA |
| OTU18737 | NA | NA | NA | NA |
| OTU11859 | NA | NA | + | - |
| OTU6764 | + | NA | NA | NA |
| OTU7647 | NA | + | NA | NA |
| OTU13204 | NA | NA | NA | NA |
| OTU18366 | NA | NA | NA | NA |
| OTU12193 | NA | NA | NA | NA |
| OTU17467 | NA | NA | NA | NA |
| OTU5291 | NA | NA | NA | NA |
| OTU10297 | NA | NA | NA | NA |
| OTU15329 | NA | NA | NA | NA |
| OTU15897 | NA | - | - | + |
| OTU16663 | NA | NA | - | + |
| OTU374 | + | NA | NA | NA |
| OTU7101 | NA | NA | NA | NA |
| OTU8597 | NA | NA | NA | NA |
| OTU14674 | - | NA | NA | NA |
| OTU5341 | NA | NA | NA | NA |
| OTU18518 | + | NA | NA | NA |
| OTU12059 | NA | NA | NA | NA |
| OTU15817 | NA | NA | NA | + |
| OTU11042 | NA | NA | NA | NA |
| OTU14433 | NA | NA | NA | + |
| OTU14915 | NA | NA | NA | NA |
| OTU7613 | + | NA | NA | NA |
| OTU15608 | NA | NA | NA | NA |
| OTU17079 | NA | NA | NA | NA |
| OTU9224 | NA | + | NA | NA |
| OTU8619 | NA | NA | NA | NA |
| OTU9682 | NA | NA | NA | NA |
| OTU9969 | NA | NA | NA | NA |
| OTU2709 | NA | NA | NA | NA |
| OTU672 | NA | NA | NA | NA |
| OTU4691 | NA | + | NA | NA |
| OTU9362 | NA | NA | NA | NA |
| OTU2696 | NA | NA | NA | NA |
| OTU10199 | NA | NA | NA | NA |
| OTU5110 | NA | NA | NA | NA |
| OTU6039 | NA | NA | NA | NA |
| OTU18577 | NA | - | NA | + |
| OTU6261 | NA | NA | NA | NA |
| OTU6012 | NA | NA | - | + |
| OTU17219 | NA | NA | NA | NA |
| OTU2708 | NA | NA | NA | NA |
| OTU13098 | NA | NA | NA | NA |
| OTU3800 | NA | NA | NA | NA |
| OTU4322 | NA | NA | NA | NA |
| OTU3560 | NA | NA | NA | NA |
| OTU4524 | NA | NA | NA | NA |
| OTU3689 | NA | NA | NA | NA |
| OTU17231 | + | NA | NA | NA |
| OTU5780 | NA | NA | NA | NA |
| OTU9750 | NA | NA | NA | NA |
| OTU18770 | NA | NA | NA | NA |
| OTU16050 | NA | - | NA | NA |
| OTU275 | NA | NA | NA | NA |
| OTU3120 | NA | NA | NA | - |
| OTU18882 | NA | NA | NA | NA |
| OTU2621 | NA | - | NA | NA |
| OTU12817 | NA | NA | NA | NA |
| OTU3068 | + | NA | NA | NA |
| OTU1493 | NA | NA | NA | NA |
| OTU15223 | NA | NA | + | NA |
| OTU16762 | NA | NA | NA | + |
| OTU12776 | NA | - | + | NA |
| OTU17110 | NA | NA | NA | NA |
| OTU12658 | NA | NA | NA | NA |
| OTU6651 | + | NA | NA | NA |
| OTU1241 | NA | NA | NA | NA |
| OTU1381 | NA | NA | NA | NA |
| OTU3692 | NA | NA | NA | NA |
| OTU6842 | NA | NA | NA | NA |
| OTU2051 | NA | NA | NA | NA |
| OTU970 | NA | NA | NA | NA |
| OTU3790 | + | NA | NA | NA |
| OTU12462 | + | NA | NA | NA |
| OTU14027 | NA | + | NA | - |
| OTU1736 | NA | NA | NA | NA |
| OTU526 | NA | NA | NA | + |
| OTU11229 | NA | NA | NA | NA |
| OTU10164 | + | NA | NA | NA |
| OTU2374 | NA | NA | NA | NA |
| OTU17044 | NA | NA | NA | NA |
| OTU15878 | NA | NA | NA | NA |
| OTU10180 | NA | NA | NA | NA |
| OTU11687 | + | - | NA | NA |
| OTU15808 | NA | NA | NA | NA |
| OTU13408 | NA | NA | + | NA |
| OTU13199 | NA | NA | NA | NA |
| OTU18951 | NA | NA | NA | NA |
| OTU2834 | + | NA | NA | NA |
| OTU4665 | NA | NA | NA | NA |
| OTU18481 | NA | NA | NA | NA |
| OTU476 | NA | NA | NA | NA |
| OTU1695 | NA | NA | NA | NA |
| OTU1438 | + | NA | NA | NA |
| OTU4639 | NA | NA | NA | NA |
| OTU9101 | NA | NA | NA | NA |
| OTU14275 | NA | NA | NA | NA |
| OTU16889 | + | NA | NA | NA |
| OTU5930 | NA | NA | NA | + |
| OTU998 | + | NA | NA | NA |
| OTU3563 | NA | NA | NA | NA |
| OTU4292 | + | NA | NA | - |
| OTU827 | + | NA | NA | NA |
| OTU12508 | NA | NA | NA | NA |
| OTU10375 | NA | NA | NA | + |
| OTU13132 | NA | NA | + | NA |
| OTU7679 | NA | NA | NA | NA |
| OTU13858 | NA | - | NA | NA |
| OTU9247 | NA | NA | NA | NA |
| OTU4819 | NA | NA | NA | NA |
| OTU4739 | NA | NA | NA | NA |
| OTU12935 | NA | NA | NA | NA |
| OTU9605 | NA | + | NA | NA |
| OTU7834 | + | NA | NA | NA |
| OTU314 | + | NA | NA | NA |
| OTU11988 | NA | NA | + | - |
| OTU9614 | NA | NA | NA | NA |
| OTU7310 | NA | NA | NA | NA |
| OTU16214 | NA | NA | NA | NA |
| OTU14829 | NA | NA | NA | NA |
| OTU12794 | NA | NA | NA | NA |
| OTU11910 | NA | NA | NA | NA |
| OTU5159 | + | NA | NA | NA |
| OTU312 | + | NA | NA | NA |
| OTU6142 | NA | NA | - | NA |
| OTU13420 | NA | NA | NA | NA |
| OTU1364 | + | NA | NA | NA |
| OTU6453 | NA | NA | NA | NA |
| OTU2636 | NA | NA | NA | + |
| OTU9272 | + | NA | NA | NA |
| OTU455 | + | NA | NA | NA |
| OTU17215 | NA | NA | NA | NA |
| OTU16905 | + | - | NA | NA |
| OTU14175 | NA | NA | NA | NA |
| OTU1610 | + | NA | NA | NA |
| OTU1118 | NA | NA | NA | NA |
| OTU6963 | NA | NA | NA | NA |
| OTU2295 | + | NA | NA | NA |
| OTU9569 | NA | NA | NA | NA |
| OTU7549 | NA | NA | NA | NA |
| OTU12430 | + | NA | NA | NA |
| OTU10673 | NA | NA | NA | NA |
| OTU5943 | NA | NA | NA | NA |
| OTU12518 | NA | NA | + | NA |
| OTU12355 | + | NA | NA | NA |
| OTU2474 | + | NA | NA | NA |
| OTU13537 | NA | NA | NA | NA |
| OTU8030 | NA | NA | NA | NA |
| OTU9658 | NA | NA | NA | NA |
| OTU938 | + | NA | NA | NA |
| OTU12578 | NA | NA | + | NA |
| OTU12216 | NA | NA | NA | NA |
| OTU4857 | NA | NA | NA | NA |
| OTU3238 | NA | NA | NA | NA |
| OTU19127 | NA | NA | NA | NA |
| OTU9561 | NA | NA | NA | NA |
| OTU7149 | NA | NA | + | NA |
| OTU9637 | NA | NA | NA | NA |
| OTU7425 | NA | NA | NA | NA |
| OTU7405 | NA | NA | NA | NA |
| OTU5489 | NA | NA | NA | NA |
| OTU4936 | NA | NA | NA | NA |
| OTU16081 | NA | NA | - | + |
| OTU7027 | NA | + | NA | NA |
| OTU17262 | NA | NA | NA | NA |
| OTU8873 | NA | NA | NA | NA |
| OTU14004 | NA | NA | NA | - |
| OTU12258 | NA | NA | + | NA |
| OTU5894 | NA | NA | NA | NA |
| OTU12572 | NA | NA | + | NA |
| OTU8960 | NA | NA | NA | NA |
| OTU13548 | + | NA | NA | NA |
| OTU1175 | NA | NA | NA | NA |
| OTU18907 | NA | NA | NA | NA |
| OTU18822 | NA | NA | NA | NA |
| OTU10036 | NA | NA | NA | NA |
| OTU16524 | NA | NA | NA | + |
| OTU18959 | NA | NA | NA | NA |
| OTU6414 | NA | NA | NA | NA |
| OTU459 | + | - | NA | NA |
| OTU8511 | NA | NA | NA | NA |
| OTU2724 | NA | NA | NA | NA |
| OTU4817 | NA | + | NA | NA |
| OTU13693 | NA | NA | NA | NA |
| OTU9944 | NA | NA | NA | NA |
| OTU12920 | + | NA | NA | NA |
| OTU11626 | + | - | NA | NA |
| OTU2742 | + | NA | NA | NA |
| OTU17881 | NA | NA | NA | + |
| OTU17466 | NA | NA | NA | NA |
| OTU8394 | NA | NA | NA | NA |
| OTU6550 | NA | - | NA | NA |
| OTU10684 | NA | NA | NA | NA |
| OTU4217 | NA | NA | NA | NA |
| OTU10247 | NA | NA | NA | NA |
| OTU1121 | + | NA | NA | NA |
| OTU1913 | NA | NA | NA | NA |
| OTU4286 | + | NA | NA | NA |
| OTU17135 | + | NA | NA | NA |
| OTU14848 | + | NA | NA | NA |
| OTU4003 | NA | NA | NA | NA |
| OTU292 | NA | NA | NA | NA |
| OTU10769 | NA | - | NA | NA |
| OTU2144 | NA | NA | NA | NA |
| OTU10342 | + | NA | NA | NA |
| OTU11032 | NA | NA | NA | NA |
| OTU7481 | NA | NA | NA | NA |
| OTU13985 | NA | NA | NA | NA |
| OTU13295 | NA | NA | NA | NA |
| OTU13577 | NA | NA | NA | NA |
| OTU12205 | NA | NA | NA | NA |
| OTU1291 | + | NA | NA | NA |
| OTU15034 | NA | NA | + | NA |
| OTU4615 | NA | NA | NA | NA |
| OTU5313 | NA | NA | NA | NA |
| OTU18949 | NA | NA | NA | NA |
| OTU2715 | NA | NA | NA | NA |
| OTU8972 | + | NA | NA | NA |
| OTU1603 | NA | NA | NA | NA |
| OTU3389 | + | NA | NA | NA |
| OTU14100 | NA | NA | NA | NA |
| OTU17127 | NA | NA | NA | NA |
| OTU3612 | NA | NA | NA | NA |
| OTU17149 | NA | NA | NA | NA |
| OTU6778 | + | NA | NA | NA |
| OTU13549 | NA | NA | NA | NA |
| OTU1789 | NA | NA | NA | NA |
| OTU12952 | NA | - | NA | NA |
| OTU4124 | NA | NA | NA | NA |
| OTU3017 | + | NA | NA | NA |
| OTU18069 | NA | NA | NA | NA |
| OTU1663 | NA | NA | NA | NA |
| OTU13474 | NA | NA | + | NA |
| OTU1946 | NA | NA | NA | NA |
| OTU1639 | NA | NA | NA | + |
| OTU6221 | NA | + | NA | NA |
| OTU5871 | + | NA | NA | NA |
| OTU5888 | NA | NA | + | NA |
| OTU14446 | NA | NA | NA | NA |
| OTU9042 | NA | NA | NA | NA |
| OTU13060 | NA | NA | + | NA |
| OTU13687 | NA | NA | + | NA |
| OTU9549 | NA | NA | NA | NA |
| OTU2762 | NA | NA | NA | NA |
| OTU8100 | NA | NA | + | NA |
| OTU18321 | NA | NA | NA | NA |
| OTU11680 | NA | NA | NA | NA |
| OTU6876 | + | NA | NA | NA |
| OTU5310 | NA | NA | + | NA |
| OTU12390 | NA | NA | NA | NA |
| OTU10037 | + | NA | NA | NA |
| OTU4002 | NA | + | - | NA |
| OTU8667 | + | NA | NA | NA |
| OTU8572 | NA | NA | NA | NA |
| OTU14110 | NA | NA | + | NA |
| OTU16887 | + | NA | NA | NA |
| OTU9343 | NA | + | NA | NA |
| OTU8656 | NA | NA | NA | NA |
| OTU4882 | NA | NA | NA | - |
| OTU5226 | NA | + | NA | NA |
| OTU6912 | NA | NA | NA | NA |
| OTU3006 | NA | NA | NA | NA |
| OTU17056 | NA | NA | NA | + |
| OTU4574 | NA | NA | NA | - |
| OTU18801 | + | - | NA | NA |
| OTU13523 | + | NA | NA | NA |
| OTU18511 | NA | NA | NA | NA |
| OTU11726 | NA | NA | + | NA |
| OTU12799 | NA | NA | NA | NA |
| OTU10665 | NA | NA | NA | NA |
| OTU6845 | NA | NA | NA | NA |
| OTU17440 | NA | NA | NA | NA |
| OTU12129 | NA | NA | NA | NA |
| OTU17369 | NA | NA | NA | + |
| OTU15657 | NA | NA | NA | NA |
| OTU8625 | NA | NA | NA | NA |
| OTU4483 | NA | NA | NA | NA |
| OTU137 | NA | NA | - | + |
| OTU11569 | NA | NA | NA | - |
| OTU3886 | NA | NA | NA | + |
| OTU10202 | NA | NA | NA | NA |
| OTU9889 | NA | + | NA | NA |
| OTU2351 | NA | NA | NA | NA |
| OTU6403 | NA | NA | NA | NA |
| OTU1877 | NA | NA | + | NA |
| OTU13745 | NA | NA | - | + |
| OTU5623 | NA | NA | NA | NA |
| OTU18827 | NA | NA | NA | NA |
| OTU14396 | NA | NA | NA | NA |
| OTU15749 | NA | NA | NA | + |
| OTU3985 | NA | NA | NA | NA |
| OTU18451 | NA | NA | NA | NA |
| OTU1115 | + | NA | NA | NA |
| OTU14379 | NA | NA | NA | NA |
| OTU9335 | NA | NA | NA | NA |
| OTU8936 | NA | + | NA | NA |
| OTU6342 | NA | NA | NA | NA |
| OTU3176 | NA | NA | NA | NA |
| OTU5852 | NA | NA | NA | NA |
| OTU12514 | NA | NA | + | - |
| OTU390 | + | NA | NA | NA |
| OTU15377 | NA | NA | NA | NA |
| OTU16530 | NA | NA | NA | NA |
| OTU3146 | NA | NA | NA | NA |
| OTU1496 | NA | NA | NA | NA |
| OTU11928 | NA | NA | NA | NA |
| OTU3719 | NA | - | NA | NA |
| OTU8997 | NA | NA | NA | NA |
| OTU8849 | NA | NA | NA | NA |
| OTU19100 | NA | NA | NA | - |
| OTU12411 | NA | NA | NA | NA |
| OTU5379 | NA | NA | NA | NA |
| OTU10238 | NA | NA | NA | NA |
| OTU761 | + | NA | NA | NA |
| OTU16302 | NA | NA | NA | NA |
| OTU13383 | NA | NA | + | NA |
| OTU401 | + | NA | NA | NA |
| OTU14042 | NA | NA | NA | NA |
| OTU5755 | NA | NA | NA | NA |
| OTU11913 | NA | NA | NA | NA |
| OTU586 | NA | NA | NA | NA |
| OTU10400 | NA | NA | NA | NA |
| OTU3622 | NA | NA | + | NA |
| OTU1989 | NA | NA | NA | NA |
| OTU12545 | NA | NA | + | NA |
| OTU4823 | NA | + | - | NA |
| OTU2485 | NA | NA | NA | NA |
| OTU15652 | NA | NA | NA | NA |
| OTU18241 | NA | NA | NA | NA |
| OTU7344 | NA | NA | NA | NA |
| OTU8095 | NA | NA | NA | NA |
| OTU10102 | + | NA | NA | NA |
| OTU2010 | NA | - | NA | NA |
| OTU1614 | NA | NA | NA | NA |
| OTU6931 | NA | NA | NA | NA |
| OTU8951 | NA | NA | NA | NA |
| OTU2363 | + | NA | NA | - |
| OTU2979 | NA | NA | NA | NA |
| OTU4625 | + | NA | NA | NA |
| OTU15513 | NA | NA | NA | NA |
| OTU15257 | NA | NA | NA | NA |
| OTU13646 | NA | NA | NA | NA |
| OTU15372 | NA | NA | NA | NA |
| OTU9541 | + | NA | NA | NA |
| OTU8227 | NA | NA | NA | NA |
| OTU10827 | NA | NA | NA | NA |
| OTU6732 | NA | NA | NA | NA |
| OTU457 | NA | NA | NA | + |
| OTU2167 | NA | NA | NA | NA |
| OTU8616 | NA | NA | NA | NA |
| OTU1129 | NA | NA | NA | NA |
| OTU9994 | NA | NA | NA | NA |
| OTU8293 | NA | NA | NA | NA |
| OTU3505 | NA | NA | NA | NA |
| OTU12939 | NA | NA | NA | NA |
| OTU8295 | NA | NA | NA | NA |
| OTU11389 | NA | NA | + | NA |
| OTU12759 | + | NA | NA | NA |
| OTU4903 | NA | NA | NA | NA |
| OTU7427 | NA | NA | NA | + |
| OTU7434 | NA | NA | NA | NA |
| OTU13075 | NA | NA | NA | NA |
| OTU8257 | NA | NA | NA | NA |
| OTU5284 | NA | NA | NA | NA |
| OTU7666 | NA | + | NA | NA |
| OTU13935 | NA | NA | NA | NA |
| OTU1630 | NA | NA | NA | NA |
| OTU18560 | NA | NA | NA | NA |
| OTU1854 | NA | NA | NA | NA |
| OTU2603 | NA | - | + | - |
| OTU12712 | NA | NA | NA | NA |
| OTU5119 | NA | NA | NA | NA |
| OTU11668 | NA | NA | NA | NA |
| OTU13543 | + | - | NA | NA |
| OTU3632 | + | NA | NA | NA |
| OTU11110 | NA | NA | NA | NA |
| OTU4794 | NA | NA | NA | NA |
| OTU426 | + | NA | NA | NA |
| OTU7287 | NA | NA | NA | NA |
| OTU11929 | NA | NA | NA | NA |
| OTU243 | NA | NA | NA | NA |
| OTU7484 | NA | NA | NA | NA |
| OTU15666 | NA | NA | NA | NA |
| OTU16596 | NA | NA | NA | NA |
| OTU7902 | NA | NA | NA | NA |
| OTU8086 | + | NA | NA | NA |
| OTU4131 | NA | NA | NA | NA |
| OTU14507 | NA | NA | NA | NA |
| OTU19049 | NA | NA | NA | NA |
| OTU7023 | NA | NA | NA | NA |
| OTU12196 | + | NA | NA | NA |
| OTU4964 | NA | NA | NA | NA |
| OTU1050 | + | NA | NA | NA |
| OTU12359 | NA | NA | + | NA |
| OTU1471 | NA | NA | NA | NA |
| OTU7132 | NA | NA | - | + |
| OTU17476 | NA | NA | NA | NA |
| OTU11465 | NA | NA | NA | NA |
| OTU2792 | NA | NA | NA | NA |
| OTU5016 | NA | NA | NA | NA |
| OTU2272 | NA | NA | NA | NA |
| OTU12427 | NA | NA | NA | NA |
| OTU9007 | NA | NA | NA | NA |
| OTU8866 | + | NA | NA | NA |
| OTU5644 | NA | NA | NA | NA |
| OTU17234 | NA | NA | NA | NA |
| OTU1130 | + | NA | NA | NA |
| OTU14266 | NA | - | NA | NA |
| OTU12376 | + | NA | NA | NA |
| OTU6214 | NA | NA | NA | NA |
| OTU8439 | NA | NA | NA | NA |
| OTU9745 | NA | NA | NA | NA |
| OTU7333 | + | NA | NA | NA |
| OTU226 | + | NA | NA | NA |
| OTU4287 | + | NA | NA | NA |
| OTU14782 | NA | NA | NA | NA |
| OTU411 | + | NA | NA | NA |
| OTU11476 | NA | NA | NA | NA |
| OTU17489 | NA | NA | NA | NA |
| OTU1188 | + | NA | NA | NA |
| OTU2002 | NA | NA | NA | NA |
| OTU980 | NA | NA | NA | NA |
| OTU16173 | NA | NA | NA | + |
| OTU13595 | NA | NA | NA | NA |
| OTU7917 | NA | NA | NA | NA |
| OTU10687 | NA | NA | NA | NA |
| OTU5288 | NA | NA | NA | NA |
| OTU8315 | NA | NA | NA | NA |
| OTU10488 | NA | NA | NA | NA |
| OTU10848 | NA | NA | + | NA |
| OTU17543 | NA | NA | NA | + |
| OTU1531 | + | NA | NA | NA |
| OTU4010 | NA | NA | NA | NA |
| OTU18610 | + | NA | NA | NA |
| OTU18618 | NA | NA | NA | NA |
| OTU6484 | NA | NA | NA | NA |
| OTU3088 | NA | NA | NA | NA |
| OTU3980 | NA | NA | NA | NA |
| OTU10657 | NA | NA | NA | NA |
| OTU2270 | NA | NA | NA | NA |
| OTU16112 | NA | NA | NA | NA |
| OTU8155 | + | - | NA | NA |
| OTU5551 | NA | NA | NA | NA |
| OTU16977 | NA | NA | NA | NA |
| OTU10699 | NA | NA | NA | NA |
| OTU4439 | NA | NA | NA | NA |
| OTU451 | + | NA | NA | NA |
| OTU4409 | NA | NA | NA | - |
| OTU6218 | NA | NA | NA | NA |
| OTU8954 | NA | NA | NA | NA |
| OTU5037 | NA | NA | NA | NA |
| OTU8634 | NA | NA | NA | NA |
| OTU10346 | NA | - | + | NA |
| OTU2101 | NA | NA | + | NA |
| OTU18375 | NA | NA | NA | NA |
| OTU15516 | NA | NA | NA | + |
| OTU8154 | NA | NA | NA | NA |
| OTU11921 | NA | NA | NA | NA |
| OTU4578 | NA | NA | NA | NA |
| OTU15326 | NA | - | NA | NA |
| OTU9314 | NA | NA | NA | NA |
| OTU8664 | NA | NA | NA | NA |
| OTU2589 | + | NA | NA | NA |
| OTU9033 | NA | NA | NA | NA |
| OTU623 | NA | NA | NA | + |
| OTU1869 | NA | NA | NA | NA |
| OTU6103 | NA | NA | NA | NA |
| OTU8604 | NA | NA | NA | NA |
| OTU16426 | NA | NA | NA | NA |
| OTU9988 | NA | NA | NA | NA |
| OTU10245 | NA | NA | NA | NA |
| OTU6023 | NA | NA | NA | NA |
| OTU10658 | + | NA | NA | NA |
| OTU6833 | NA | NA | NA | NA |
| OTU15472 | NA | NA | NA | + |
| OTU8072 | NA | NA | NA | NA |
| OTU11884 | NA | NA | NA | NA |
| OTU6027 | NA | NA | NA | NA |
| OTU9339 | + | NA | NA | NA |
| OTU12735 | NA | NA | NA | NA |
| OTU13500 | NA | NA | NA | NA |
| OTU17955 | NA | - | NA | NA |
| OTU18720 | NA | NA | NA | NA |
| OTU10847 | + | NA | NA | NA |
| OTU11798 | NA | - | NA | NA |
| OTU2966 | NA | NA | NA | NA |
| OTU12864 | NA | NA | + | NA |
| OTU4422 | + | NA | NA | NA |
| OTU17088 | NA | NA | NA | NA |
| OTU18091 | NA | NA | NA | NA |
| OTU5540 | NA | + | NA | NA |
| OTU10640 | NA | NA | NA | NA |
| OTU18859 | NA | NA | NA | NA |
| OTU34 | NA | NA | NA | - |
| OTU932 | NA | NA | NA | NA |
| OTU14520 | NA | + | NA | NA |
| OTU8396 | NA | NA | NA | NA |
| OTU15854 | NA | NA | NA | NA |
| OTU17797 | NA | NA | NA | NA |
| OTU3332 | NA | NA | NA | NA |
| OTU14450 | + | - | NA | NA |
| OTU12459 | NA | NA | NA | NA |
| OTU8406 | NA | NA | NA | NA |
| OTU14362 | NA | NA | + | NA |
| OTU6440 | + | NA | NA | NA |
| OTU6658 | NA | NA | NA | NA |
| OTU10820 | NA | NA | NA | NA |
| OTU9497 | + | NA | NA | NA |
| OTU1904 | NA | NA | NA | NA |
| OTU5972 | NA | NA | NA | NA |
| OTU4315 | + | NA | NA | NA |
| OTU7641 | NA | NA | NA | NA |
| OTU7767 | NA | NA | NA | + |
| OTU16191 | NA | NA | NA | NA |
| OTU18495 | NA | NA | NA | NA |
| OTU17085 | NA | NA | NA | + |
| OTU11911 | NA | NA | NA | NA |
| OTU16414 | NA | - | NA | NA |
| OTU6640 | NA | NA | NA | NA |
| OTU18719 | NA | NA | NA | NA |
| OTU5338 | NA | NA | NA | NA |
| OTU4302 | NA | NA | NA | NA |
| OTU6813 | NA | NA | + | - |
| OTU18526 | NA | NA | NA | NA |
| OTU7091 | NA | NA | NA | NA |
| OTU9286 | NA | NA | NA | NA |
| OTU10218 | NA | NA | NA | NA |
| OTU143 | NA | NA | NA | NA |
| OTU10296 | NA | NA | NA | NA |
| OTU15504 | NA | NA | NA | NA |
| OTU6499 | NA | NA | NA | NA |
| OTU8940 | NA | NA | NA | NA |
| OTU9680 | NA | NA | NA | NA |
| OTU5994 | NA | NA | NA | NA |
| OTU15993 | NA | NA | NA | NA |
| OTU8582 | NA | NA | NA | NA |
| OTU958 | NA | NA | NA | NA |
| OTU7497 | + | NA | NA | NA |
| OTU4480 | NA | NA | NA | NA |
| OTU1141 | NA | NA | NA | NA |
| OTU8864 | + | NA | NA | NA |
| OTU6962 | NA | NA | NA | NA |
| OTU4868 | NA | + | NA | NA |
| OTU12946 | NA | NA | NA | NA |
| OTU11848 | NA | NA | NA | + |
| OTU1797 | NA | NA | NA | NA |
| OTU8786 | NA | NA | NA | NA |
| OTU17448 | NA | NA | NA | NA |
| OTU10324 | NA | NA | NA | NA |
| OTU12654 | NA | NA | + | NA |
| OTU13546 | NA | NA | NA | NA |
| OTU8526 | NA | + | NA | NA |
| OTU1659 | NA | NA | NA | + |
| OTU4400 | + | NA | NA | NA |
| OTU15376 | NA | NA | NA | NA |
| OTU9740 | NA | NA | NA | NA |
| OTU18824 | NA | - | NA | NA |
| OTU18565 | + | NA | NA | NA |
| OTU18634 | NA | NA | NA | + |
| OTU16165 | NA | NA | NA | NA |
| OTU6308 | NA | NA | NA | NA |
| OTU10612 | NA | NA | NA | NA |
| OTU1782 | NA | NA | NA | + |
| OTU12435 | NA | NA | NA | NA |
| OTU14029 | NA | NA | + | - |
| OTU16755 | NA | NA | NA | NA |
| OTU12608 | NA | NA | NA | NA |
| OTU18918 | NA | NA | NA | NA |
| OTU14051 | NA | NA | NA | NA |
| OTU5939 | NA | + | NA | NA |
| OTU1145 | NA | NA | NA | NA |
| OTU18778 | NA | NA | NA | + |
| OTU15529 | NA | NA | NA | NA |
| OTU3364 | NA | NA | NA | NA |
| OTU8858 | NA | NA | NA | NA |
| OTU15953 | NA | NA | NA | NA |
| OTU6147 | NA | NA | NA | NA |
| OTU17087 | NA | NA | NA | NA |
| OTU1423 | NA | NA | NA | NA |
| OTU5679 | NA | NA | NA | NA |
| OTU11271 | NA | NA | NA | NA |
| OTU16767 | NA | NA | NA | NA |
| OTU9304 | NA | NA | NA | NA |
| OTU18845 | + | - | NA | NA |
| OTU9648 | NA | NA | NA | NA |
| OTU5703 | NA | NA | NA | NA |
| OTU4185 | NA | NA | NA | NA |
| OTU9665 | NA | + | NA | NA |
| OTU14261 | NA | NA | NA | NA |
| OTU17524 | NA | NA | NA | + |
| OTU5759 | NA | NA | NA | + |
| OTU8503 | NA | NA | NA | NA |
| OTU9485 | NA | NA | NA | NA |
| OTU17254 | NA | NA | NA | + |
| OTU2580 | + | NA | NA | NA |
| OTU5619 | NA | NA | NA | NA |
| OTU17744 | NA | NA | NA | + |
| OTU5224 | NA | NA | NA | NA |
| OTU10097 | + | NA | NA | NA |
| OTU1708 | NA | NA | NA | NA |
| OTU3685 | NA | NA | NA | NA |
| OTU17193 | NA | NA | NA | NA |
| OTU5258 | NA | NA | NA | NA |
| OTU6574 | NA | NA | NA | NA |
| OTU3698 | + | NA | NA | NA |
| OTU4496 | NA | NA | NA | - |
| OTU4102 | NA | NA | NA | NA |
| OTU12102 | NA | NA | NA | NA |
| OTU16097 | NA | NA | NA | NA |
| OTU14982 | NA | NA | NA | NA |
| OTU8292 | NA | NA | NA | NA |
| OTU4336 | NA | NA | NA | NA |
| OTU19130 | + | - | NA | NA |
| OTU18401 | NA | NA | NA | NA |
| OTU6794 | + | NA | NA | NA |
| OTU3789 | NA | NA | NA | NA |
| OTU12304 | NA | NA | NA | NA |
| OTU11023 | NA | NA | + | NA |
| OTU16346 | NA | NA | NA | NA |
| OTU5146 | NA | NA | NA | NA |
| OTU13978 | NA | NA | NA | NA |
| OTU17793 | NA | NA | NA | + |
| OTU13315 | NA | NA | NA | NA |
| OTU327 | NA | NA | NA | NA |
| OTU2309 | NA | NA | NA | NA |
| OTU16010 | NA | NA | NA | NA |
| OTU13623 | NA | NA | NA | NA |
| OTU8005 | NA | NA | NA | NA |
| OTU4271 | NA | NA | NA | NA |
| OTU388 | + | NA | NA | NA |
| OTU3694 | + | - | NA | NA |
| OTU688 | + | NA | NA | NA |
| OTU2221 | + | NA | NA | NA |
| OTU9261 | + | NA | NA | NA |
| OTU11548 | NA | NA | NA | NA |
| OTU3609 | + | NA | NA | NA |
| OTU14327 | NA | NA | + | NA |
| OTU11922 | NA | NA | + | - |
| OTU18940 | NA | NA | NA | NA |
| OTU5613 | NA | NA | NA | NA |
| OTU9710 | NA | NA | NA | NA |
| OTU14618 | NA | NA | NA | NA |
| OTU3161 | NA | NA | NA | NA |
| OTU8645 | NA | NA | NA | NA |
| OTU502 | + | NA | NA | NA |
| OTU3468 | NA | NA | NA | NA |
| OTU1293 | NA | NA | NA | NA |
| OTU15250 | NA | NA | NA | NA |
| OTU7272 | NA | NA | NA | NA |
| OTU13344 | NA | NA | + | NA |
| OTU9825 | NA | NA | NA | NA |
| OTU550 | NA | NA | NA | NA |
| OTU1823 | + | - | NA | NA |
| OTU2219 | NA | NA | NA | NA |
| OTU6545 | + | NA | NA | NA |
| OTU12605 | NA | NA | + | - |
| OTU16538 | NA | NA | NA | NA |
| OTU1568 | NA | NA | + | NA |
| OTU3144 | + | NA | NA | NA |
| OTU3701 | NA | NA | + | NA |
| OTU15311 | NA | NA | NA | NA |
| OTU15691 | NA | NA | NA | NA |
| OTU16384 | + | NA | NA | NA |
| OTU6423 | NA | NA | NA | NA |
| OTU6711 | + | NA | NA | NA |
| OTU475 | + | NA | NA | NA |
| OTU12126 | NA | NA | NA | NA |
| OTU8853 | NA | NA | NA | NA |
| OTU9168 | NA | NA | NA | NA |
| OTU3182 | NA | NA | NA | NA |
| OTU7535 | + | NA | NA | NA |
| OTU3212 | NA | NA | NA | NA |
| OTU13430 | NA | NA | NA | NA |
| OTU224 | NA | NA | NA | NA |
| OTU14838 | NA | NA | + | NA |
| OTU17364 | NA | NA | NA | NA |
| OTU15810 | NA | NA | NA | NA |
| OTU15832 | NA | NA | + | NA |
| OTU6583 | NA | NA | NA | NA |
| OTU6041 | NA | NA | NA | NA |
| OTU17173 | NA | NA | NA | NA |
| OTU11137 | NA | NA | NA | NA |
| OTU12031 | NA | NA | NA | NA |
| OTU1995 | NA | NA | NA | NA |
| OTU9564 | NA | NA | NA | NA |
| OTU7096 | NA | + | NA | NA |
| OTU8464 | + | NA | NA | NA |
| OTU3650 | NA | NA | NA | NA |
| OTU1026 | NA | NA | NA | NA |
| OTU8630 | NA | NA | NA | NA |
| OTU13250 | NA | NA | NA | NA |
| OTU10070 | NA | NA | NA | NA |
| OTU8646 | NA | NA | NA | NA |
| OTU7284 | NA | NA | NA | NA |
| OTU18619 | + | NA | NA | NA |
| OTU10220 | NA | NA | + | NA |
| OTU7936 | NA | NA | NA | NA |
| OTU1518 | NA | NA | NA | NA |
| OTU7448 | NA | NA | NA | NA |
| OTU17315 | NA | NA | NA | NA |
| OTU12535 | NA | NA | NA | NA |
| OTU15165 | NA | - | NA | NA |
| OTU9787 | NA | NA | NA | NA |
| OTU6951 | NA | NA | NA | NA |
| OTU17540 | NA | NA | NA | NA |
| OTU15286 | NA | NA | NA | NA |
| OTU263 | NA | NA | NA | NA |
| OTU995 | + | NA | NA | NA |
| OTU2387 | + | NA | NA | NA |
| OTU2517 | NA | NA | NA | NA |
| OTU17228 | NA | NA | NA | NA |
| OTU6144 | NA | NA | NA | NA |
| OTU9937 | NA | NA | NA | NA |
| OTU8828 | NA | NA | NA | NA |
| OTU5723 | NA | NA | NA | NA |
| OTU15956 | NA | NA | NA | NA |
| OTU4637 | NA | NA | NA | NA |
| OTU16319 | + | NA | NA | NA |
| OTU1238 | NA | NA | NA | NA |
| OTU9458 | + | NA | NA | NA |
| OTU9773 | NA | - | NA | NA |
| OTU18694 | NA | NA | NA | NA |
| OTU11717 | + | NA | NA | NA |
| OTU11665 | + | NA | NA | NA |
| OTU14330 | NA | NA | + | NA |
| OTU3331 | + | - | NA | NA |
| OTU283 | + | NA | NA | NA |
| OTU15288 | NA | NA | + | NA |
| OTU19051 | NA | NA | NA | NA |
| OTU11671 | NA | NA | NA | NA |
| OTU8910 | NA | NA | NA | NA |
| OTU14681 | NA | NA | NA | NA |
| OTU11118 | NA | NA | NA | NA |
| OTU4490 | NA | NA | NA | NA |
| OTU13754 | NA | NA | NA | NA |
| OTU11026 | NA | NA | + | NA |
| OTU6841 | NA | NA | + | NA |
| OTU468 | + | NA | NA | NA |
| OTU529 | NA | - | NA | NA |
| OTU8123 | NA | NA | NA | NA |
| OTU13583 | NA | NA | NA | NA |
| OTU3629 | NA | NA | NA | NA |
| OTU10082 | NA | NA | NA | NA |
| OTU16862 | NA | NA | NA | NA |
| OTU5018 | NA | NA | NA | NA |
| OTU5817 | NA | NA | NA | NA |
| OTU14369 | NA | NA | NA | NA |
| OTU948 | NA | NA | NA | NA |
| OTU4718 | NA | NA | NA | NA |
| OTU2933 | NA | NA | + | NA |
| OTU11814 | NA | NA | NA | NA |
| OTU4679 | NA | NA | NA | NA |
| OTU488 | NA | NA | NA | NA |
| OTU18598 | NA | NA | NA | + |
| OTU13983 | NA | NA | + | NA |
| OTU8651 | NA | NA | NA | NA |
| OTU6527 | NA | NA | NA | NA |
| OTU756 | + | NA | NA | NA |
| OTU11170 | NA | NA | + | NA |
| OTU4141 | NA | NA | NA | + |
| OTU10685 | NA | NA | NA | NA |
| OTU10048 | NA | NA | NA | NA |
| OTU331 | NA | NA | NA | NA |
| OTU7153 | NA | NA | NA | NA |
| OTU12754 | NA | NA | NA | NA |
| OTU9118 | + | NA | NA | NA |
| OTU13219 | NA | NA | NA | NA |
| OTU946 | + | NA | NA | NA |
| OTU9890 | NA | NA | NA | NA |
| OTU15904 | NA | NA | NA | NA |
| OTU11448 | NA | NA | NA | NA |
| OTU8804 | + | NA | NA | NA |
| OTU7034 | NA | NA | NA | NA |
| OTU9073 | NA | NA | NA | NA |
| OTU7825 | + | NA | NA | NA |
| OTU11532 | NA | NA | + | NA |
| OTU699 | + | NA | NA | NA |
| OTU13911 | NA | NA | NA | NA |
| OTU5125 | + | NA | NA | NA |
| OTU5505 | NA | NA | NA | NA |
| OTU8451 | NA | NA | NA | - |
| OTU5726 | NA | + | NA | NA |
| OTU16872 | NA | NA | NA | NA |
| OTU14521 | NA | NA | NA | NA |
| OTU6822 | NA | NA | NA | NA |
| OTU4331 | + | NA | NA | NA |
| OTU16025 | NA | NA | NA | NA |
| OTU183 | NA | NA | NA | NA |
| OTU8318 | NA | NA | NA | NA |
| OTU15457 | NA | - | NA | NA |
| OTU15987 | NA | NA | NA | NA |
| OTU4414 | + | NA | NA | NA |
| OTU11205 | NA | NA | NA | NA |
| OTU4419 | NA | NA | NA | NA |
| OTU17025 | NA | NA | NA | NA |
| OTU11895 | NA | NA | NA | NA |
| OTU3460 | NA | NA | NA | NA |
| OTU8688 | NA | NA | NA | NA |
| OTU11650 | NA | NA | NA | NA |
| OTU10282 | NA | NA | NA | NA |
| OTU6441 | NA | NA | NA | NA |
| OTU92 | NA | NA | NA | NA |
| OTU4870 | NA | NA | NA | NA |
| OTU6229 | NA | NA | NA | NA |
| OTU16584 | + | NA | NA | - |
| OTU8023 | NA | NA | NA | NA |
| OTU18439 | NA | NA | NA | NA |
| OTU10329 | NA | NA | NA | NA |
| OTU13940 | NA | NA | NA | NA |
| OTU16074 | NA | NA | NA | NA |
| OTU16367 | NA | NA | NA | NA |
| OTU1776 | NA | NA | NA | NA |
| OTU7114 | NA | NA | NA | NA |
| OTU10603 | NA | NA | NA | NA |
| OTU7539 | NA | NA | NA | NA |
| OTU599 | NA | NA | NA | NA |
| OTU4226 | NA | NA | NA | NA |
| OTU16531 | NA | NA | NA | NA |
| OTU191 | NA | NA | NA | NA |
| OTU2538 | NA | NA | NA | NA |
| OTU6753 | NA | - | NA | NA |
| OTU695 | NA | NA | NA | NA |
| OTU5295 | NA | NA | NA | NA |
| OTU10518 | NA | NA | NA | NA |
| OTU6721 | + | NA | NA | NA |
| OTU9647 | NA | NA | NA | NA |
| OTU17491 | NA | NA | NA | NA |
| OTU12598 | NA | NA | NA | NA |
| OTU15264 | NA | NA | NA | NA |
| OTU16525 | NA | NA | NA | NA |
| OTU6812 | NA | NA | NA | NA |
| OTU11391 | NA | NA | + | NA |
| OTU16683 | NA | NA | NA | NA |
| OTU3356 | NA | NA | NA | NA |
| OTU12679 | + | NA | NA | NA |
| OTU1273 | NA | NA | NA | NA |
| OTU5891 | + | - | + | NA |
| OTU8701 | NA | NA | NA | NA |
| OTU5206 | NA | - | NA | NA |
| OTU12694 | NA | NA | NA | NA |
| OTU4004 | NA | NA | NA | NA |
| OTU6504 | NA | NA | NA | + |
| OTU8307 | NA | NA | NA | NA |
| OTU3793 | NA | NA | NA | NA |
| OTU5632 | NA | NA | NA | NA |
| OTU9656 | NA | NA | + | NA |
| OTU10557 | NA | NA | NA | NA |
| OTU4568 | NA | NA | NA | NA |
| OTU1999 | NA | NA | NA | + |
| OTU11661 | + | NA | NA | NA |
| OTU6898 | NA | NA | NA | NA |
| OTU10700 | NA | NA | NA | NA |
| OTU11670 | NA | NA | NA | NA |
| OTU14684 | NA | NA | NA | NA |
| OTU18723 | NA | NA | NA | NA |
| OTU11456 | NA | NA | NA | NA |
| OTU6587 | NA | NA | NA | NA |
| OTU452 | + | NA | NA | NA |
| OTU2389 | + | NA | NA | NA |
| OTU728 | + | NA | NA | NA |
| OTU15244 | NA | NA | NA | + |
| OTU15711 | NA | NA | NA | NA |
| OTU6307 | NA | NA | NA | NA |
| OTU2802 | + | NA | NA | NA |
| OTU12403 | NA | NA | NA | NA |
| OTU15522 | NA | NA | NA | NA |
| OTU14866 | NA | NA | + | NA |
| OTU15917 | NA | NA | NA | NA |
| OTU15302 | NA | NA | NA | NA |
| OTU18605 | NA | NA | NA | NA |
| OTU449 | + | NA | NA | NA |
| OTU8536 | NA | NA | NA | NA |
| OTU1316 | + | NA | NA | NA |
| OTU863 | + | NA | NA | NA |
| OTU5090 | NA | NA | NA | NA |
| OTU690 | NA | NA | NA | NA |
| OTU8934 | NA | + | NA | NA |
| OTU16329 | NA | NA | NA | + |
| OTU12289 | NA | NA | NA | NA |
| OTU8614 | NA | NA | NA | NA |
| OTU1075 | NA | NA | NA | NA |
| OTU4487 | + | NA | NA | NA |
| OTU13364 | NA | NA | NA | NA |
| OTU9025 | NA | NA | NA | + |
| OTU11333 | NA | NA | + | NA |
| OTU10414 | NA | NA | NA | NA |
| OTU4269 | NA | NA | NA | NA |
| OTU1318 | NA | NA | NA | NA |
| OTU400 | + | NA | NA | NA |
| OTU16488 | NA | NA | NA | NA |
| OTU6814 | + | NA | NA | NA |
| OTU4386 | NA | NA | NA | NA |
| OTU11357 | NA | NA | NA | NA |
| OTU16554 | NA | NA | NA | NA |
| OTU16205 | NA | NA | NA | NA |
| OTU12661 | NA | NA | NA | NA |
| OTU14980 | NA | NA | NA | - |
| OTU7474 | + | NA | NA | NA |
| OTU15852 | NA | NA | NA | NA |
| OTU8577 | NA | NA | NA | NA |
| OTU16556 | NA | NA | NA | NA |
| OTU15664 | NA | NA | NA | NA |
| OTU8288 | + | NA | NA | NA |
| OTU7569 | NA | NA | NA | NA |
| OTU12449 | NA | NA | NA | NA |
| OTU2302 | NA | NA | NA | NA |
| OTU13496 | NA | NA | NA | NA |
| OTU8393 | NA | NA | NA | NA |
| OTU14035 | NA | NA | NA | NA |
| OTU471 | NA | NA | NA | NA |
| OTU10107 | NA | NA | NA | NA |
| OTU15992 | NA | NA | NA | NA |
| OTU16899 | + | NA | NA | NA |
| OTU17276 | NA | NA | NA | NA |
| OTU10149 | NA | NA | NA | NA |
| OTU10973 | + | NA | NA | NA |
| OTU2543 | NA | NA | NA | NA |
| OTU1220 | NA | NA | NA | NA |
| OTU4380 | NA | NA | NA | NA |
| OTU2561 | + | NA | NA | NA |
| OTU5652 | NA | NA | NA | + |
| OTU3354 | NA | NA | NA | NA |
| OTU11822 | NA | NA | NA | - |
| OTU16890 | NA | NA | NA | NA |
| OTU1215 | NA | NA | NA | NA |
| OTU15212 | NA | NA | NA | NA |
| OTU3136 | NA | NA | NA | NA |
| OTU3477 | NA | NA | NA | NA |
| OTU15182 | NA | NA | NA | + |
| OTU15364 | NA | NA | NA | NA |
| OTU4305 | NA | - | + | - |
| OTU2384 | + | NA | NA | NA |
| OTU6200 | NA | NA | NA | NA |
| OTU5635 | NA | NA | NA | NA |
| OTU16068 | NA | NA | NA | + |
| OTU7763 | NA | NA | NA | NA |
| OTU5661 | NA | NA | NA | NA |
| OTU12682 | NA | NA | NA | NA |
| OTU7868 | NA | NA | NA | NA |
| OTU16937 | + | NA | NA | NA |
| OTU7720 | + | NA | NA | NA |
| OTU7846 | + | NA | NA | NA |
| OTU9939 | + | NA | + | NA |
| OTU747 | + | NA | NA | NA |
| OTU8993 | NA | NA | NA | NA |
| OTU9063 | NA | NA | NA | NA |
| OTU1779 | NA | NA | NA | NA |
| OTU10874 | NA | NA | NA | NA |
| OTU506 | NA | NA | NA | NA |
| OTU8491 | NA | NA | NA | NA |
| OTU11050 | NA | NA | + | NA |
| OTU6953 | NA | NA | NA | NA |
| OTU9958 | NA | NA | NA | NA |
| OTU1416 | NA | NA | NA | NA |
| OTU3323 | NA | NA | NA | NA |
| OTU5161 | NA | NA | NA | NA |
| OTU5470 | NA | NA | NA | NA |
| OTU3004 | NA | NA | NA | NA |
| OTU9348 | NA | NA | NA | NA |
| OTU2681 | + | NA | NA | NA |
| OTU18106 | NA | NA | NA | NA |
| OTU3703 | + | NA | NA | NA |
| OTU3160 | + | NA | NA | NA |
| OTU5151 | NA | NA | NA | NA |
| OTU6401 | NA | NA | NA | NA |
| OTU8909 | NA | NA | NA | NA |
| OTU6167 | NA | NA | NA | NA |
| OTU7040 | NA | NA | NA | NA |
| OTU4889 | NA | NA | NA | NA |
| OTU9756 | NA | NA | + | NA |
| OTU6494 | NA | NA | NA | NA |
| OTU10244 | NA | NA | NA | NA |
| OTU10320 | NA | NA | NA | + |
| OTU1931 | NA | NA | NA | NA |
| OTU13735 | NA | NA | NA | NA |
| OTU10264 | NA | NA | NA | NA |
| OTU4994 | NA | NA | NA | NA |
| OTU15378 | NA | NA | NA | NA |
| OTU7396 | + | NA | NA | NA |
| OTU1788 | NA | NA | NA | - |
| OTU5708 | NA | NA | NA | NA |
| OTU18879 | NA | NA | NA | NA |
| OTU12800 | NA | NA | NA | NA |
| OTU16729 | NA | NA | NA | NA |
| OTU5762 | + | NA | NA | NA |
| OTU10476 | NA | NA | + | NA |
| OTU3259 | NA | NA | NA | NA |
| OTU12739 | NA | NA | NA | NA |
| OTU9759 | NA | NA | NA | NA |
| OTU4728 | NA | NA | NA | NA |
| OTU9225 | NA | NA | NA | NA |
| OTU9067 | NA | NA | NA | + |
| OTU17291 | NA | NA | NA | + |
| OTU11010 | + | NA | NA | NA |
| OTU1247 | NA | NA | NA | NA |
| OTU2437 | NA | NA | NA | NA |
| OTU8524 | NA | NA | NA | NA |
| OTU1678 | NA | NA | + | - |
| OTU15229 | NA | NA | NA | NA |
| OTU15851 | + | - | NA | NA |
| OTU3774 | + | NA | NA | NA |
| OTU15355 | NA | NA | NA | + |
| OTU5673 | NA | NA | NA | NA |
| OTU4546 | NA | NA | NA | NA |
| OTU7404 | NA | NA | NA | NA |
| OTU3620 | NA | NA | NA | NA |
| OTU5952 | NA | NA | NA | + |
| OTU11045 | NA | NA | NA | NA |
| OTU6972 | NA | NA | NA | NA |
| OTU16450 | NA | NA | NA | NA |
| OTU2791 | NA | NA | NA | NA |
| OTU12540 | NA | NA | + | NA |
| OTU14269 | NA | NA | + | NA |
| OTU444 | + | NA | NA | NA |
| OTU19193 | NA | NA | NA | NA |
| OTU13401 | NA | + | NA | NA |
| OTU3999 | NA | NA | NA | NA |
| OTU18653 | NA | NA | NA | NA |
| OTU787 | + | NA | NA | NA |
| OTU8705 | NA | NA | NA | NA |
| OTU8468 | NA | NA | NA | NA |
| OTU8239 | + | NA | NA | NA |
| OTU10867 | NA | NA | NA | + |
| OTU6407 | NA | NA | NA | NA |
| OTU12964 | NA | NA | NA | NA |
| OTU9655 | + | NA | NA | NA |
| OTU4938 | NA | NA | NA | NA |
| OTU4485 | NA | NA | NA | NA |
| OTU288 | NA | NA | NA | NA |
| OTU14299 | NA | NA | NA | NA |
| OTU2298 | NA | NA | NA | NA |
| OTU3954 | NA | NA | NA | + |
| OTU9792 | NA | NA | + | NA |
| OTU1638 | NA | NA | NA | NA |
| OTU12336 | + | NA | NA | NA |
| OTU18887 | NA | NA | NA | NA |
| OTU6173 | NA | NA | NA | NA |
| OTU14121 | NA | NA | NA | NA |
| OTU4584 | NA | NA | NA | NA |
| OTU10631 | NA | NA | NA | NA |
| OTU18785 | + | NA | NA | NA |
| OTU12865 | NA | NA | NA | NA |
| OTU2412 | NA | NA | NA | + |
| OTU11742 | NA | NA | NA | NA |
| OTU11990 | NA | NA | + | NA |
| OTU18652 | NA | NA | NA | + |
| OTU8469 | NA | NA | NA | NA |
| OTU16055 | NA | NA | NA | NA |
| OTU11099 | NA | NA | NA | NA |
| OTU8156 | NA | NA | NA | NA |
| OTU5167 | NA | NA | NA | NA |
| OTU11120 | NA | NA | NA | + |
| OTU6228 | NA | NA | NA | NA |
| OTU5475 | NA | NA | NA | NA |
| OTU1478 | + | NA | NA | NA |
| OTU6567 | NA | NA | NA | NA |
| OTU16485 | NA | NA | NA | NA |
| OTU10338 | NA | NA | NA | NA |
| OTU16419 | NA | NA | NA | NA |
| OTU18581 | NA | NA | NA | + |
| OTU15954 | NA | NA | NA | NA |
| OTU2497 | + | NA | NA | NA |
| OTU11461 | NA | NA | + | NA |
| OTU17371 | NA | NA | NA | NA |
| OTU13636 | NA | NA | NA | NA |
| OTU7796 | NA | NA | NA | NA |
| OTU6624 | NA | NA | NA | NA |
| OTU8270 | NA | NA | NA | NA |
| OTU16698 | NA | NA | NA | NA |
| OTU16234 | NA | NA | NA | + |
| OTU5576 | NA | NA | + | NA |
| OTU3448 | NA | NA | NA | NA |
| OTU10353 | NA | NA | NA | NA |
| OTU13302 | NA | + | NA | NA |
| OTU9023 | NA | NA | NA | NA |
| OTU10695 | NA | NA | + | NA |
| OTU3557 | NA | NA | NA | NA |
| OTU7626 | NA | NA | NA | NA |
| OTU3820 | + | NA | NA | NA |
| OTU10774 | NA | NA | NA | NA |
| OTU12951 | NA | + | NA | NA |
| OTU1651 | NA | NA | NA | NA |
| OTU18809 | + | NA | NA | NA |
| OTU6608 | + | NA | NA | NA |
| OTU4377 | NA | NA | NA | NA |
| OTU5021 | NA | NA | NA | NA |
| OTU12702 | NA | NA | NA | NA |
| OTU1911 | NA | NA | NA | NA |
| OTU12326 | + | NA | NA | NA |
| OTU9196 | NA | NA | - | NA |
| OTU19020 | NA | NA | NA | NA |
| OTU15889 | + | NA | NA | NA |
| OTU5261 | NA | NA | NA | NA |
| OTU9198 | NA | NA | NA | NA |
| OTU5705 | NA | NA | NA | NA |
| OTU5240 | NA | NA | NA | NA |
| OTU18751 | NA | NA | NA | NA |
| OTU8029 | NA | NA | NA | NA |
| OTU4276 | NA | NA | NA | NA |
| OTU6447 | NA | + | NA | NA |
| OTU11508 | NA | NA | NA | NA |
| OTU6502 | NA | NA | NA | NA |
| OTU5000 | NA | NA | + | NA |
| OTU12217 | NA | NA | NA | + |
| OTU3791 | + | NA | NA | NA |
| OTU7226 | NA | NA | NA | NA |
| OTU16506 | NA | NA | NA | NA |
| OTU1067 | NA | NA | NA | NA |
| OTU8122 | NA | NA | NA | NA |
| OTU11479 | NA | NA | NA | - |
| OTU870 | + | NA | NA | NA |
| OTU10813 | NA | NA | NA | NA |
| OTU15943 | NA | - | + | NA |
| OTU384 | NA | NA | NA | + |
| OTU2721 | NA | - | NA | NA |
| OTU17686 | NA | NA | NA | NA |
| OTU8682 | NA | NA | NA | NA |
| OTU15176 | NA | NA | NA | NA |
| OTU3174 | NA | NA | NA | NA |
| OTU1893 | NA | NA | NA | NA |
| OTU7321 | NA | NA | NA | NA |
| OTU4833 | NA | NA | NA | NA |
| OTU2828 | NA | NA | NA | + |
| OTU15622 | + | NA | NA | NA |
| OTU861 | NA | NA | NA | NA |
| OTU5448 | NA | NA | NA | NA |
| OTU6026 | NA | NA | NA | NA |
| OTU5357 | NA | NA | NA | NA |
| OTU3016 | NA | NA | NA | NA |
| OTU544 | NA | NA | NA | NA |
| OTU2399 | + | NA | NA | NA |
| OTU16638 | NA | NA | NA | NA |
| OTU14368 | + | NA | NA | NA |
| OTU1832 | NA | NA | NA | NA |
| OTU2737 | NA | NA | NA | NA |
| OTU1825 | NA | NA | NA | + |
| OTU1206 | NA | NA | NA | NA |
| OTU10668 | NA | NA | NA | NA |
| OTU8576 | + | NA | NA | NA |
| OTU5571 | + | NA | NA | NA |
| OTU454 | NA | - | NA | NA |
| OTU1035 | NA | NA | NA | NA |
| OTU5988 | NA | NA | NA | + |
| OTU10529 | + | NA | NA | NA |
| OTU12769 | NA | NA | NA | NA |
| OTU11686 | NA | NA | NA | NA |
| OTU6150 | NA | NA | NA | NA |
| OTU3266 | NA | NA | NA | NA |
| OTU7899 | + | NA | NA | NA |
| OTU10878 | NA | NA | NA | NA |
| OTU18902 | NA | NA | NA | NA |
| OTU4797 | NA | NA | NA | NA |
| OTU18968 | NA | NA | NA | NA |
| OTU1152 | + | NA | NA | NA |
| OTU3214 | + | NA | NA | NA |
| OTU9009 | NA | NA | + | NA |
| OTU18830 | NA | NA | NA | NA |
| OTU216 | + | NA | NA | NA |
| OTU16093 | NA | NA | NA | NA |
| OTU3240 | NA | NA | NA | NA |
| OTU4 | NA | NA | NA | NA |
| OTU6271 | NA | + | NA | NA |
| OTU5095 | NA | NA | NA | NA |
| OTU8240 | NA | NA | NA | NA |
| OTU156 | NA | NA | NA | NA |
| OTU4858 | NA | NA | NA | NA |
| OTU18732 | + | NA | NA | NA |
| OTU8452 | NA | NA | NA | NA |
| OTU10435 | NA | NA | NA | NA |
| OTU15027 | NA | NA | NA | NA |
| OTU19155 | NA | NA | NA | NA |
| OTU1330 | + | NA | NA | NA |
| OTU4295 | + | NA | NA | NA |
| OTU739 | NA | NA | NA | NA |
| OTU2461 | + | NA | NA | NA |
| OTU6916 | NA | NA | NA | NA |
| OTU6751 | + | NA | NA | NA |
| OTU13459 | NA | NA | NA | NA |
| OTU1396 | + | NA | NA | NA |
| OTU19090 | + | NA | NA | NA |
| OTU16284 | NA | - | NA | NA |
| OTU8236 | NA | NA | NA | NA |
| OTU12233 | NA | NA | NA | NA |
| OTU10980 | + | - | NA | NA |
| OTU12549 | NA | NA | + | NA |
| OTU10285 | NA | NA | NA | NA |
| OTU14558 | NA | NA | NA | NA |
| OTU12957 | NA | NA | NA | NA |
| OTU18190 | NA | NA | NA | NA |
| OTU2237 | + | NA | NA | NA |
| OTU14451 | NA | NA | NA | NA |
| OTU16659 | NA | NA | NA | NA |
| OTU18371 | NA | NA | NA | + |
| OTU2653 | NA | NA | NA | NA |
| OTU9094 | NA | NA | NA | NA |
| OTU5838 | NA | NA | NA | NA |
| OTU17631 | NA | NA | NA | NA |
| OTU18686 | NA | NA | NA | NA |
| OTU10089 | + | NA | NA | NA |
| OTU1116 | NA | NA | NA | NA |
| OTU16249 | NA | NA | NA | NA |
| OTU6079 | NA | NA | NA | NA |
| OTU5425 | NA | NA | NA | NA |
| OTU18787 | + | NA | NA | NA |
| OTU16968 | NA | NA | - | + |
| OTU5033 | NA | NA | NA | NA |
| OTU3959 | NA | + | NA | NA |
| OTU677 | NA | NA | NA | NA |
| OTU6348 | NA | + | NA | NA |
| OTU17218 | NA | NA | NA | NA |
| OTU8046 | + | NA | NA | NA |
| OTU4786 | NA | NA | NA | NA |
| OTU3447 | + | NA | NA | NA |
| OTU14079 | NA | NA | NA | NA |
| OTU10361 | NA | NA | NA | NA |
| OTU1697 | NA | NA | + | NA |
| OTU15895 | NA | NA | NA | NA |
| OTU12802 | NA | NA | NA | NA |
| OTU15663 | + | NA | NA | NA |
| OTU12525 | NA | NA | NA | NA |
| OTU9393 | NA | NA | NA | NA |
| OTU9891 | NA | NA | NA | NA |
| OTU16624 | NA | NA | NA | NA |
| OTU219 | + | NA | NA | NA |
| OTU5184 | NA | NA | NA | NA |
| OTU1656 | NA | NA | NA | NA |
| OTU6099 | NA | NA | NA | NA |
| OTU10373 | NA | NA | NA | + |
| OTU9086 | NA | NA | NA | NA |
| OTU18023 | NA | NA | NA | NA |
| OTU15614 | + | NA | NA | NA |
| OTU3486 | NA | NA | NA | NA |
| OTU785 | + | NA | NA | NA |
| OTU10055 | NA | NA | + | NA |
| OTU7690 | NA | + | - | NA |
| OTU6683 | NA | NA | NA | NA |
| OTU4733 | NA | NA | NA | NA |
| OTU11861 | NA | NA | + | - |
| OTU1897 | NA | NA | NA | NA |
| OTU9230 | NA | NA | NA | NA |
| OTU2556 | + | NA | NA | NA |
| OTU4105 | NA | NA | NA | NA |
| OTU7180 | NA | NA | NA | NA |
| OTU10741 | NA | NA | NA | NA |
| OTU11254 | NA | NA | NA | NA |
| OTU332 | NA | NA | NA | NA |
| OTU16908 | + | NA | NA | NA |
| OTU8617 | + | NA | NA | NA |
| OTU18796 | + | NA | NA | NA |
| OTU14246 | NA | NA | NA | NA |
| OTU8495 | NA | NA | NA | NA |
| OTU17984 | NA | NA | NA | NA |
| OTU14343 | NA | NA | + | NA |
| OTU12377 | NA | NA | NA | NA |
| OTU18731 | NA | NA | NA | NA |
| OTU18622 | + | NA | NA | NA |
| OTU13307 | + | NA | NA | NA |
| OTU9250 | NA | NA | NA | NA |
| OTU6225 | NA | NA | NA | NA |
| OTU8648 | NA | NA | NA | NA |
| OTU7971 | NA | - | NA | NA |
| OTU1686 | NA | NA | NA | NA |
| OTU9817 | NA | NA | + | NA |
| OTU16474 | NA | NA | NA | NA |
| OTU3890 | NA | NA | NA | NA |
| OTU14805 | NA | NA | NA | NA |
| OTU4407 | NA | NA | NA | NA |
| OTU18228 | NA | NA | NA | NA |
| OTU18283 | NA | NA | NA | NA |
| OTU14278 | NA | NA | + | NA |
| OTU13945 | NA | NA | NA | NA |
| OTU18754 | NA | NA | NA | NA |
| OTU16674 | NA | NA | NA | NA |
| OTU10090 | + | NA | NA | NA |
| OTU11739 | + | NA | NA | NA |
| OTU6992 | NA | NA | NA | NA |
| OTU10702 | NA | - | NA | NA |
| OTU4294 | + | - | NA | NA |
| OTU15227 | + | NA | NA | NA |
| OTU15883 | NA | NA | NA | NA |
| OTU1048 | NA | NA | NA | NA |
| OTU16727 | NA | + | NA | NA |
| OTU13186 | + | NA | NA | NA |
| OTU3121 | NA | NA | NA | NA |
| OTU10959 | + | NA | NA | NA |
| OTU11316 | NA | NA | NA | NA |
| OTU9166 | + | NA | NA | NA |
| OTU2431 | NA | NA | NA | NA |
| OTU8602 | NA | NA | NA | NA |
| OTU16795 | NA | - | NA | NA |
| OTU11276 | NA | NA | NA | NA |
| OTU9941 | + | NA | NA | NA |
| OTU7067 | NA | NA | NA | NA |
| OTU3060 | NA | NA | NA | NA |
| OTU18900 | NA | NA | NA | NA |
| OTU4599 | NA | NA | NA | NA |
| OTU14539 | NA | NA | NA | NA |
| OTU12586 | NA | NA | NA | NA |
| OTU558 | + | NA | NA | NA |
| OTU17443 | NA | NA | NA | NA |
| OTU12422 | NA | NA | NA | NA |
| OTU5351 | NA | NA | NA | NA |
| OTU18741 | NA | NA | NA | NA |
| OTU12727 | NA | NA | NA | NA |
| OTU10560 | + | NA | NA | NA |
| OTU7905 | NA | NA | NA | NA |
| OTU9239 | + | NA | NA | NA |
| OTU10448 | NA | NA | NA | NA |
| OTU3889 | NA | NA | NA | NA |
| OTU7928 | NA | NA | NA | NA |
| OTU18788 | NA | NA | NA | NA |
| OTU5102 | NA | NA | NA | NA |
| OTU9419 | + | NA | NA | NA |
| OTU5974 | NA | NA | NA | NA |
| OTU9744 | NA | NA | NA | NA |
| OTU17072 | NA | NA | NA | NA |
| OTU3375 | + | NA | NA | NA |
| OTU5282 | NA | NA | NA | NA |
| OTU16599 | NA | NA | NA | NA |
| OTU8621 | NA | NA | NA | NA |
| OTU12655 | NA | NA | + | NA |
| OTU17307 | NA | NA | - | NA |
| OTU5835 | NA | NA | NA | NA |
| OTU16845 | NA | NA | NA | NA |
| OTU18916 | NA | NA | NA | NA |
| OTU1427 | + | NA | NA | NA |
| OTU17495 | NA | NA | NA | NA |
| OTU15050 | NA | NA | NA | NA |
| OTU11049 | NA | NA | NA | NA |
| OTU3879 | NA | NA | NA | NA |
| OTU18219 | NA | NA | NA | NA |
| OTU1343 | NA | NA | NA | NA |
| OTU2705 | + | NA | NA | NA |
| OTU15417 | NA | NA | NA | + |
| OTU15395 | NA | NA | NA | NA |
| OTU7730 | + | NA | NA | NA |
| OTU2702 | + | NA | NA | NA |
| OTU17063 | NA | NA | NA | + |
| OTU12431 | + | NA | NA | NA |
| OTU9434 | NA | NA | NA | NA |
| OTU316 | NA | NA | NA | NA |
| OTU10151 | NA | NA | + | NA |
| OTU2450 | NA | NA | NA | NA |
| OTU4034 | NA | NA | NA | NA |
| OTU8053 | + | NA | NA | NA |
| OTU4734 | NA | NA | NA | NA |
| OTU11097 | NA | NA | NA | NA |
| OTU10438 | + | NA | NA | NA |
| OTU9481 | + | NA | NA | NA |
| OTU5498 | NA | NA | NA | NA |
| OTU9460 | + | NA | NA | NA |
| OTU5174 | NA | NA | NA | NA |
| OTU1533 | + | NA | NA | NA |
| OTU909 | NA | NA | NA | NA |
| OTU4664 | NA | NA | NA | NA |
| OTU7563 | NA | + | NA | NA |
| OTU4369 | NA | NA | NA | NA |
| OTU10119 | NA | NA | NA | NA |
| OTU6197 | NA | NA | NA | NA |
| OTU18623 | NA | NA | NA | NA |
| OTU7413 | + | NA | NA | NA |
| OTU13732 | NA | NA | NA | NA |
| OTU16728 | NA | NA | + | NA |
| OTU15494 | NA | NA | NA | NA |
| OTU13377 | NA | NA | NA | NA |
| OTU7884 | NA | NA | NA | NA |
| OTU6358 | NA | NA | NA | - |
| OTU17051 | NA | NA | NA | NA |
| OTU13581 | NA | NA | + | NA |
| OTU2212 | NA | - | NA | NA |
| OTU6178 | NA | NA | NA | NA |
| OTU6125 | NA | NA | NA | NA |
| OTU9234 | + | NA | NA | NA |
| OTU6302 | NA | NA | NA | NA |
| OTU10943 | + | NA | NA | NA |
| OTU1003 | NA | NA | NA | NA |
| OTU6219 | NA | + | NA | NA |
| OTU658 | + | NA | NA | NA |
| OTU9418 | + | NA | NA | NA |
| OTU11816 | NA | NA | + | NA |
| OTU759 | NA | NA | NA | NA |
| OTU2571 | NA | NA | NA | NA |
| OTU8861 | NA | NA | NA | NA |
| OTU8350 | NA | NA | - | NA |
| OTU1142 | NA | NA | NA | NA |
| OTU10005 | NA | NA | + | NA |
| OTU3094 | + | NA | NA | NA |
| OTU4406 | NA | NA | NA | NA |
| OTU18195 | NA | NA | NA | + |
| OTU1077 | + | NA | NA | NA |
| OTU11526 | NA | NA | NA | NA |
| OTU4473 | + | NA | NA | NA |
| OTU11056 | + | NA | NA | NA |
| OTU17069 | NA | - | NA | + |
| OTU15373 | NA | NA | NA | NA |
| OTU3716 | + | NA | NA | NA |
| OTU12620 | NA | NA | NA | NA |
| OTU5862 | NA | NA | NA | NA |
| OTU1312 | NA | NA | NA | NA |
| OTU2029 | NA | NA | NA | NA |
| OTU4116 | NA | NA | NA | NA |
| OTU3156 | + | NA | NA | NA |
| OTU18798 | + | NA | NA | NA |
| OTU17882 | NA | NA | NA | NA |
| OTU10428 | NA | NA | NA | + |
| OTU7345 | NA | NA | NA | NA |
| OTU11471 | + | NA | NA | NA |
| OTU5667 | NA | NA | NA | NA |
| OTU14778 | NA | NA | NA | NA |
| OTU3431 | NA | NA | NA | NA |
| OTU16133 | NA | NA | NA | NA |
| OTU4073 | NA | NA | NA | NA |
| OTU448 | + | - | NA | NA |
| OTU6427 | NA | NA | NA | NA |
| OTU10040 | NA | NA | NA | - |
| OTU16559 | NA | NA | NA | NA |
| OTU9110 | NA | NA | NA | NA |
| OTU18596 | NA | NA | NA | NA |
| OTU1227 | NA | NA | NA | NA |
| OTU12967 | + | NA | NA | NA |
| OTU6216 | NA | NA | NA | NA |
| OTU141 | + | NA | NA | NA |
| OTU1674 | + | NA | NA | NA |
| OTU15785 | NA | NA | NA | + |
| OTU13573 | NA | NA | NA | NA |
| OTU4053 | NA | NA | NA | NA |
| OTU9572 | NA | NA | NA | NA |
| OTU18171 | + | NA | NA | NA |
| OTU8200 | + | NA | NA | NA |
| OTU6736 | + | NA | NA | NA |
| OTU13388 | NA | NA | NA | NA |
| OTU1183 | NA | NA | NA | NA |
| OTU8774 | NA | NA | NA | NA |
| OTU7993 | NA | NA | NA | NA |
| OTU15478 | NA | NA | NA | NA |
| OTU4735 | NA | NA | NA | + |
| OTU2382 | + | NA | NA | NA |
| OTU2908 | + | NA | NA | NA |
| OTU3853 | NA | NA | NA | NA |
| OTU14068 | + | NA | NA | NA |
| OTU2336 | + | - | NA | NA |
| OTU7228 | NA | NA | NA | NA |
| OTU11979 | NA | NA | NA | NA |
| OTU5622 | NA | NA | + | NA |
| OTU14787 | NA | NA | NA | NA |
| OTU10069 | NA | NA | NA | NA |
| OTU7901 | NA | NA | NA | NA |
| OTU6844 | NA | + | NA | NA |
| OTU2452 | + | NA | NA | NA |
| OTU4925 | NA | NA | NA | NA |
| OTU7146 | NA | - | NA | NA |
| OTU17722 | NA | NA | NA | NA |
| OTU14178 | NA | NA | NA | NA |
| OTU4850 | NA | NA | NA | NA |
| OTU9162 | NA | NA | NA | NA |
| OTU17923 | NA | NA | NA | NA |
| OTU2243 | + | NA | NA | NA |
| OTU17183 | NA | NA | NA | NA |
| OTU4678 | NA | + | NA | NA |
| OTU12407 | NA | NA | NA | NA |
| OTU6030 | + | NA | NA | NA |
| OTU5265 | NA | NA | NA | NA |
| OTU187 | NA | NA | NA | NA |
| OTU4587 | NA | NA | NA | NA |
| OTU515 | NA | NA | NA | NA |
| OTU8249 | NA | NA | NA | NA |
| OTU2630 | + | NA | NA | NA |
| OTU9135 | NA | NA | NA | NA |
| OTU7282 | NA | NA | NA | NA |
| OTU1487 | NA | NA | NA | NA |
| OTU9751 | NA | NA | NA | NA |
| OTU17475 | NA | NA | NA | NA |
| OTU12206 | NA | NA | NA | NA |
| OTU9093 | NA | NA | NA | NA |
| OTU10500 | NA | NA | NA | NA |
| OTU14247 | NA | NA | + | NA |
| OTU8277 | NA | NA | NA | NA |
| OTU3780 | + | - | NA | NA |
| OTU2046 | NA | NA | NA | NA |
| OTU4202 | NA | NA | NA | NA |
| OTU850 | NA | NA | NA | NA |
| OTU3370 | NA | NA | NA | NA |
| OTU4199 | NA | NA | NA | NA |
| OTU9381 | NA | NA | NA | NA |
| OTU17303 | NA | NA | NA | NA |
| OTU94 | + | NA | NA | NA |
| OTU14499 | NA | NA | NA | NA |
| OTU5279 | NA | + | NA | NA |
| OTU11703 | NA | NA | NA | NA |
| OTU11904 | NA | NA | + | NA |
| OTU14486 | NA | NA | + | NA |
| OTU11106 | NA | NA | NA | NA |
| OTU3952 | NA | NA | NA | NA |
| OTU9193 | NA | NA | NA | NA |
| OTU7878 | NA | NA | NA | NA |
| OTU251 | + | NA | NA | NA |
| OTU12143 | NA | NA | + | NA |
| OTU16606 | NA | NA | NA | NA |
| OTU10316 | NA | NA | NA | NA |
| OTU17451 | NA | NA | NA | NA |
| OTU17263 | NA | NA | NA | NA |
| OTU3279 | + | NA | NA | NA |
| OTU2187 | NA | NA | NA | NA |
| OTU4815 | NA | NA | NA | NA |
| OTU14876 | NA | NA | + | NA |
| OTU9928 | NA | - | + | NA |
| OTU4918 | NA | NA | NA | NA |
| OTU5770 | NA | NA | NA | NA |
| OTU10302 | NA | NA | NA | NA |
| OTU18415 | NA | NA | + | NA |
| OTU2572 | NA | NA | NA | NA |
| OTU17992 | NA | NA | NA | NA |
| OTU16152 | + | - | NA | NA |
| OTU15840 | NA | NA | NA | NA |
| OTU13528 | NA | NA | NA | NA |
| OTU10389 | NA | NA | NA | NA |
| OTU12684 | NA | NA | NA | NA |
| OTU7996 | NA | NA | NA | NA |
| OTU16005 | NA | NA | NA | NA |
| OTU12367 | NA | NA | NA | NA |
| OTU7545 | + | NA | NA | NA |
| OTU12426 | NA | NA | NA | NA |
| OTU10124 | NA | NA | NA | NA |
| OTU6975 | NA | NA | NA | NA |
| OTU16697 | NA | NA | NA | NA |
| OTU12730 | NA | NA | NA | NA |
| OTU5731 | NA | NA | NA | NA |
| OTU546 | + | NA | NA | NA |
| OTU10855 | NA | NA | NA | NA |
| OTU11457 | NA | NA | NA | NA |
| OTU6827 | NA | NA | NA | + |
| OTU13683 | NA | NA | NA | NA |
| OTU5918 | NA | NA | NA | NA |
| OTU132 | + | NA | NA | NA |
| OTU6060 | NA | NA | NA | NA |
| OTU2899 | + | NA | NA | NA |
| OTU7789 | NA | NA | NA | NA |
| OTU12559 | NA | NA | NA | NA |
| OTU9540 | + | NA | NA | NA |
| OTU14438 | NA | + | NA | NA |
| OTU15414 | NA | NA | NA | NA |
| OTU6581 | + | NA | NA | NA |
| OTU14568 | NA | NA | NA | NA |
| OTU12264 | NA | NA | NA | NA |
| OTU11426 | NA | NA | NA | NA |
| OTU10326 | NA | NA | NA | NA |
| OTU9182 | NA | NA | NA | NA |
| OTU4383 | NA | NA | NA | NA |
| OTU12161 | NA | NA | NA | NA |
| OTU8605 | NA | NA | NA | NA |
| OTU7987 | NA | NA | NA | NA |
| OTU1210 | + | NA | NA | NA |
| OTU1881 | NA | - | NA | NA |
| OTU16146 | NA | NA | NA | NA |
| OTU17113 | NA | NA | NA | NA |
| OTU8665 | NA | NA | NA | NA |
| OTU18508 | NA | NA | NA | + |
| OTU5413 | + | NA | NA | NA |
| OTU7711 | + | NA | NA | NA |
| OTU4495 | + | NA | NA | NA |
| OTU10465 | NA | NA | NA | NA |
| OTU4670 | NA | NA | NA | NA |
| OTU249 | NA | NA | NA | NA |
| OTU1539 | NA | NA | NA | NA |
| OTU11735 | NA | NA | NA | NA |
| OTU16406 | NA | - | NA | NA |
| OTU2493 | + | NA | NA | NA |
| OTU17112 | NA | NA | NA | NA |
| OTU1835 | NA | NA | NA | NA |
| OTU4330 | NA | NA | NA | NA |
| OTU9390 | NA | NA | NA | NA |
| OTU18679 | NA | NA | NA | NA |
| OTU16687 | NA | NA | NA | NA |
| OTU18390 | NA | NA | NA | NA |
| OTU16049 | NA | NA | NA | + |
| OTU11701 | NA | NA | + | NA |
| OTU3777 | NA | NA | NA | NA |
| OTU15107 | NA | NA | NA | NA |
| OTU376 | NA | NA | NA | NA |
| OTU11434 | + | NA | NA | NA |
| OTU2865 | NA | NA | NA | NA |
| OTU12596 | + | NA | NA | NA |
| OTU4325 | NA | NA | NA | NA |
| OTU1556 | NA | + | NA | NA |
| OTU17427 | NA | NA | NA | + |
| OTU11008 | + | NA | NA | NA |
| OTU3803 | NA | NA | NA | NA |
| OTU10430 | + | NA | + | NA |
| OTU8889 | + | NA | NA | NA |
| OTU17105 | NA | NA | NA | NA |
| OTU182 | + | NA | NA | NA |
| OTU18588 | NA | NA | NA | NA |
| OTU9254 | NA | NA | NA | NA |
| OTU11125 | NA | NA | + | NA |
| OTU12321 | NA | NA | NA | NA |
| OTU4997 | NA | NA | NA | NA |
| OTU8762 | NA | NA | NA | NA |
| OTU11806 | NA | NA | + | NA |
| OTU4645 | NA | NA | NA | NA |
| OTU18590 | NA | NA | - | + |
| OTU16543 | NA | NA | NA | NA |
| OTU1317 | NA | NA | NA | NA |
| OTU133 | + | NA | NA | NA |
| OTU12806 | NA | NA | NA | NA |
| OTU3142 | NA | NA | NA | NA |
| OTU1953 | NA | NA | NA | NA |
| OTU8373 | NA | NA | - | NA |
| OTU6387 | NA | NA | NA | NA |
| OTU6882 | NA | NA | NA | NA |
| OTU10443 | NA | NA | NA | NA |
| OTU6709 | NA | NA | NA | NA |
| OTU14774 | NA | NA | NA | NA |
| OTU4873 | NA | NA | NA | NA |
| OTU18572 | NA | + | NA | NA |
| OTU12567 | NA | NA | NA | NA |
| OTU17464 | NA | NA | NA | NA |
| OTU8758 | NA | NA | NA | NA |
| OTU8561 | NA | NA | NA | NA |
| OTU10255 | NA | NA | + | NA |
| OTU6239 | NA | NA | NA | NA |
| OTU1693 | NA | NA | NA | NA |
| OTU1243 | NA | NA | NA | NA |
| OTU17707 | NA | NA | NA | NA |
| OTU5686 | NA | NA | NA | NA |
| OTU1988 | + | NA | NA | NA |
| OTU16594 | NA | NA | NA | NA |
| OTU3347 | + | - | NA | NA |
| OTU4399 | NA | NA | + | NA |
| OTU8810 | + | NA | NA | NA |
| OTU9629 | NA | NA | NA | - |
| OTU15210 | NA | NA | NA | + |
| OTU2198 | NA | NA | NA | NA |
| OTU12619 | NA | NA | NA | NA |
| OTU3466 | + | NA | NA | NA |
| OTU11714 | + | NA | NA | NA |
| OTU13478 | NA | NA | NA | NA |
| OTU294 | NA | NA | NA | NA |
| OTU14577 | NA | + | NA | NA |
| OTU12096 | NA | NA | NA | NA |
| OTU16553 | NA | NA | NA | NA |
| OTU9107 | NA | NA | NA | NA |
| OTU11651 | NA | NA | NA | NA |
| OTU14274 | + | NA | NA | NA |
| OTU16798 | NA | NA | NA | NA |
| OTU13135 | NA | NA | NA | NA |
| OTU14153 | NA | NA | NA | NA |
| OTU7880 | + | NA | NA | NA |
| OTU10216 | NA | NA | NA | NA |
| OTU7948 | + | NA | NA | NA |
| OTU16359 | NA | NA | NA | NA |
| OTU16125 | NA | NA | NA | + |
| OTU12378 | NA | NA | + | NA |
| OTU18893 | NA | NA | NA | NA |
| OTU6702 | NA | NA | NA | NA |
| OTU192 | NA | NA | NA | NA |
| OTU203 | + | NA | NA | NA |
| OTU11260 | NA | + | NA | NA |
| OTU3141 | NA | NA | NA | NA |
| OTU17281 | NA | NA | NA | + |
| OTU6171 | NA | NA | NA | NA |
| OTU14919 | NA | NA | NA | NA |
| OTU6817 | + | NA | NA | NA |
| OTU700 | + | NA | NA | NA |
| OTU6162 | NA | NA | NA | NA |
| OTU807 | + | NA | NA | NA |
| OTU2093 | + | NA | NA | NA |
| OTU521 | NA | NA | NA | NA |
| OTU4040 | NA | NA | NA | NA |
| OTU16776 | NA | NA | NA | + |
| OTU5487 | + | NA | NA | NA |
| OTU4025 | NA | NA | NA | NA |
| OTU9005 | NA | + | - | NA |
| OTU3123 | NA | NA | NA | NA |
| OTU15215 | NA | NA | NA | NA |
| OTU10999 | NA | NA | NA | NA |
| OTU14810 | NA | NA | NA | NA |
| OTU10267 | NA | NA | NA | NA |
| OTU3867 | + | NA | NA | NA |
| OTU3990 | NA | NA | NA | NA |
| OTU18334 | NA | NA | + | NA |
| OTU1409 | NA | NA | NA | NA |
| OTU13526 | NA | NA | NA | NA |
| OTU18595 | NA | NA | NA | NA |
| OTU11863 | NA | NA | + | NA |
| OTU17417 | NA | NA | NA | NA |
| OTU18871 | NA | NA | NA | NA |
| OTU10517 | NA | NA | + | NA |
| OTU3809 | NA | NA | NA | NA |
| OTU11200 | NA | NA | NA | NA |
| OTU8348 | + | NA | NA | NA |
| OTU9151 | NA | NA | NA | - |
| OTU4499 | NA | NA | NA | NA |
| OTU19202 | NA | NA | NA | NA |
| OTU10972 | NA | NA | NA | NA |
| OTU9613 | NA | NA | NA | NA |
| OTU4085 | NA | NA | NA | NA |
| OTU2147 | NA | NA | NA | NA |
| OTU6667 | + | NA | NA | NA |
| OTU14920 | NA | NA | + | NA |
| OTU13887 | NA | NA | NA | NA |
| OTU4704 | NA | NA | NA | NA |
| OTU5283 | NA | NA | NA | NA |
| OTU15401 | + | NA | NA | NA |
| OTU5931 | NA | NA | NA | NA |
| OTU5035 | NA | NA | NA | NA |
| OTU12889 | NA | NA | + | NA |
| OTU8440 | NA | NA | NA | NA |
| OTU16270 | NA | NA | NA | NA |
| OTU6246 | NA | NA | NA | NA |
| OTU6245 | NA | NA | NA | NA |
| OTU16183 | NA | NA | NA | + |
| OTU3260 | NA | NA | NA | NA |
| OTU8119 | NA | NA | NA | NA |
| OTU7004 | NA | NA | NA | NA |
| OTU16839 | NA | NA | NA | NA |
| OTU15656 | + | NA | NA | NA |
| OTU13458 | NA | NA | NA | NA |
| OTU9213 | NA | NA | NA | NA |
| OTU11676 | NA | NA | NA | NA |
| OTU28 | NA | NA | NA | NA |
| OTU17953 | NA | NA | NA | NA |
| OTU2732 | + | NA | NA | NA |
| OTU6786 | NA | NA | NA | NA |
| OTU12020 | NA | NA | NA | NA |
| OTU5344 | NA | NA | NA | NA |
| OTU4544 | NA | + | NA | NA |
| OTU8908 | NA | NA | NA | NA |
| OTU17261 | NA | NA | NA | NA |
| OTU10563 | NA | NA | NA | NA |
| OTU18470 | NA | NA | NA | + |
| OTU6086 | NA | NA | NA | NA |
| OTU5976 | NA | NA | NA | NA |
| OTU5019 | NA | NA | NA | NA |
| OTU1023 | NA | NA | NA | + |
| OTU2523 | NA | NA | NA | NA |
| OTU12993 | NA | NA | NA | NA |
| OTU11632 | NA | NA | NA | NA |
| OTU4282 | + | NA | NA | NA |
| OTU8206 | NA | NA | NA | NA |
| OTU1847 | NA | NA | NA | NA |
| OTU8772 | + | NA | NA | NA |
| OTU5141 | NA | NA | NA | NA |
| OTU13697 | NA | NA | NA | NA |
| OTU3699 | NA | NA | NA | NA |
| OTU12033 | NA | NA | NA | NA |
| OTU6629 | NA | NA | NA | NA |
| OTU5844 | NA | NA | NA | NA |
| OTU10528 | NA | NA | NA | NA |
| OTU11689 | NA | NA | + | NA |
| OTU4748 | NA | NA | NA | NA |
| OTU8446 | NA | NA | NA | NA |
| OTU1648 | NA | NA | NA | NA |
| OTU2502 | NA | NA | NA | NA |
| OTU15801 | NA | NA | NA | NA |
| OTU7840 | NA | NA | NA | NA |
| OTU67 | NA | + | NA | NA |
| OTU16400 | NA | + | NA | NA |
| OTU6593 | NA | NA | NA | NA |
| OTU15448 | NA | NA | NA | NA |
| OTU160 | NA | NA | NA | NA |
| OTU3328 | + | NA | NA | NA |
| OTU18743 | NA | NA | NA | + |
| OTU16629 | NA | NA | NA | NA |
| OTU17133 | NA | NA | NA | NA |
| OTU3293 | NA | NA | NA | NA |
| OTU5356 | NA | NA | NA | NA |
| OTU1908 | NA | NA | NA | NA |
| OTU434 | NA | NA | NA | NA |
| OTU3669 | NA | NA | NA | NA |
| OTU13071 | + | NA | NA | NA |
| OTU12719 | NA | NA | NA | NA |
| OTU2640 | + | NA | NA | NA |
| OTU8407 | NA | NA | NA | NA |
| OTU7057 | NA | + | NA | NA |
| OTU2534 | NA | NA | NA | NA |
| OTU7391 | + | NA | NA | NA |
| OTU6971 | NA | NA | NA | NA |
| OTU13857 | NA | NA | NA | NA |
| OTU6653 | NA | NA | NA | NA |
| OTU13558 | NA | NA | NA | - |
| OTU15880 | NA | NA | + | NA |
| OTU12673 | NA | NA | NA | NA |
| OTU3819 | + | NA | NA | NA |
| OTU15693 | NA | NA | NA | NA |
| OTU7080 | NA | NA | NA | NA |
| OTU10692 | NA | NA | NA | NA |
| OTU391 | NA | NA | NA | NA |
| OTU11166 | NA | NA | NA | NA |
| OTU17182 | NA | NA | NA | NA |
| OTU5497 | NA | NA | NA | NA |
| OTU11038 | NA | NA | NA | NA |
| OTU4895 | NA | NA | NA | NA |
| OTU11154 | NA | NA | NA | NA |
| OTU5469 | NA | NA | NA | NA |
| OTU16716 | NA | NA | NA | NA |
| OTU5964 | + | NA | NA | NA |
| OTU11858 | NA | NA | NA | NA |
| OTU12801 | NA | NA | NA | NA |
| OTU5787 | NA | NA | NA | NA |
| OTU2439 | NA | NA | NA | NA |
| OTU17245 | NA | NA | NA | NA |
| OTU7940 | NA | NA | NA | NA |
| OTU18543 | NA | NA | NA | NA |
| OTU8091 | NA | NA | NA | NA |
| OTU4447 | + | NA | NA | NA |
| OTU1887 | NA | NA | NA | NA |
| OTU11560 | NA | NA | + | NA |
| OTU15544 | + | NA | NA | NA |
| OTU7128 | NA | NA | NA | NA |
| OTU14342 | NA | NA | NA | NA |
| OTU15907 | NA | NA | NA | NA |
| OTU15430 | NA | NA | NA | + |
| OTU8341 | + | NA | NA | NA |
| OTU3147 | + | NA | NA | NA |
| OTU268 | + | NA | NA | NA |
| OTU12212 | NA | NA | NA | NA |
| OTU4869 | NA | NA | NA | NA |
| OTU6065 | NA | NA | NA | NA |
| OTU1034 | + | NA | NA | NA |
| OTU5235 | NA | NA | NA | NA |
| OTU7200 | NA | NA | NA | NA |
| OTU12653 | NA | NA | NA | NA |
| OTU8593 | NA | NA | NA | NA |
| OTU10163 | NA | - | NA | NA |
| OTU12141 | NA | NA | NA | NA |
| OTU12721 | NA | NA | NA | NA |
| OTU16480 | NA | NA | NA | NA |
| OTU4790 | NA | NA | NA | NA |
| OTU469 | + | NA | NA | NA |
| OTU320 | NA | NA | NA | NA |
| OTU8102 | + | NA | NA | NA |
| OTU2361 | NA | NA | NA | NA |
| OTU10610 | NA | NA | NA | NA |
| OTU9785 | NA | NA | + | NA |
| OTU4931 | NA | NA | NA | NA |
| OTU17573 | NA | NA | NA | NA |
| OTU7930 | NA | NA | NA | NA |
| OTU13487 | NA | NA | + | NA |
| OTU18633 | NA | NA | NA | NA |
| OTU1356 | + | NA | NA | NA |
| OTU14044 | NA | NA | NA | NA |
| OTU16509 | NA | NA | NA | NA |
| OTU11139 | NA | NA | NA | NA |
| OTU14284 | NA | NA | NA | NA |
| OTU12515 | NA | NA | NA | NA |
| OTU11799 | NA | NA | NA | NA |
| OTU14032 | NA | NA | + | NA |
| OTU2210 | NA | NA | NA | NA |
| OTU4054 | NA | + | NA | NA |
| OTU3148 | + | NA | NA | NA |
| OTU8677 | NA | NA | NA | NA |
| OTU6802 | NA | NA | NA | NA |
| OTU17040 | NA | NA | NA | NA |
| OTU2394 | NA | NA | NA | NA |
| OTU17870 | NA | NA | NA | NA |
| OTU2518 | NA | NA | NA | NA |
| OTU16504 | NA | NA | NA | NA |
| OTU14295 | NA | NA | + | NA |
| OTU1919 | NA | - | NA | NA |
| OTU8230 | NA | NA | NA | NA |
| OTU6966 | NA | NA | NA | NA |
| OTU4879 | NA | NA | NA | NA |
| OTU16051 | NA | NA | NA | NA |
| OTU1372 | NA | NA | NA | NA |
| OTU10318 | NA | NA | NA | NA |
| OTU17142 | NA | NA | NA | NA |
| OTU10778 | NA | NA | NA | NA |
| OTU5657 | NA | NA | NA | NA |
| OTU15916 | NA | NA | NA | NA |
| OTU11753 | NA | NA | NA | NA |
| OTU18767 | NA | NA | NA | NA |
| OTU18836 | NA | NA | NA | NA |
| OTU2948 | NA | NA | NA | NA |
| OTU5965 | NA | NA | NA | NA |
| OTU3877 | NA | NA | NA | NA |
| OTU17588 | NA | NA | NA | NA |
| OTU11522 | NA | NA | NA | NA |
| OTU18823 | NA | NA | NA | NA |
| OTU18167 | NA | NA | NA | NA |
| OTU3518 | + | NA | NA | NA |
| OTU4069 | NA | + | NA | NA |
| OTU3134 | NA | NA | NA | NA |
| OTU10142 | NA | NA | NA | NA |
| OTU3981 | NA | NA | NA | NA |
| OTU16471 | NA | NA | NA | NA |
| OTU8588 | NA | NA | NA | NA |
| OTU11477 | NA | NA | NA | NA |
| OTU10022 | NA | NA | NA | NA |
| OTU13504 | NA | NA | + | NA |
| OTU18736 | NA | NA | + | NA |
| OTU5373 | + | NA | NA | - |
| OTU792 | NA | NA | NA | NA |
| OTU1558 | + | NA | NA | NA |
| OTU16878 | + | NA | NA | NA |
| OTU12830 | NA | NA | NA | NA |
| OTU6699 | NA | NA | NA | NA |
| OTU13552 | + | NA | NA | NA |
| OTU1591 | NA | NA | NA | NA |
| OTU10586 | NA | NA | NA | + |
| OTU16088 | NA | NA | NA | NA |
| OTU10593 | NA | NA | NA | NA |
| OTU7233 | NA | NA | NA | NA |
| OTU7504 | NA | NA | NA | NA |
| OTU11496 | + | NA | NA | NA |
| OTU3927 | NA | NA | NA | NA |
| OTU14418 | NA | NA | NA | NA |
| OTU13669 | NA | NA | NA | NA |
| OTU3978 | NA | NA | NA | NA |
| OTU6053 | NA | NA | NA | NA |
| OTU9324 | + | NA | NA | NA |
| OTU6362 | NA | + | NA | NA |
| OTU12558 | NA | NA | NA | NA |
| OTU9090 | NA | NA | NA | NA |
| OTU3844 | NA | NA | NA | NA |
| OTU14478 | NA | NA | NA | NA |
| OTU2876 | + | NA | NA | NA |
| OTU18987 | NA | NA | NA | NA |
| OTU6662 | NA | NA | NA | NA |
| OTU5959 | NA | NA | NA | NA |
| OTU10962 | NA | NA | NA | NA |
| OTU12612 | NA | NA | NA | NA |
| OTU8499 | NA | NA | NA | NA |
| OTU4402 | + | NA | NA | NA |
| OTU14533 | NA | NA | NA | NA |
| OTU1492 | NA | NA | NA | NA |
| OTU525 | NA | NA | NA | NA |
| OTU4097 | NA | NA | NA | - |
| OTU510 | NA | NA | NA | NA |
| OTU18814 | NA | NA | NA | + |
| OTU10653 | NA | NA | + | NA |
| OTU6174 | NA | NA | NA | NA |
| OTU3577 | NA | NA | NA | NA |
| OTU6886 | NA | + | NA | NA |
| OTU12369 | NA | NA | NA | NA |
| OTU1792 | NA | NA | NA | NA |
| OTU1576 | + | NA | NA | NA |
| OTU9686 | NA | NA | + | NA |
| OTU10682 | NA | NA | NA | NA |
| OTU4756 | NA | NA | NA | NA |
| OTU18832 | NA | NA | NA | NA |
| OTU3428 | NA | NA | + | NA |
| OTU5508 | NA | NA | NA | NA |
| OTU13927 | + | NA | NA | NA |
| OTU6682 | NA | NA | NA | NA |
| OTU16589 | NA | NA | NA | NA |
| OTU7106 | NA | NA | NA | NA |
| OTU8558 | NA | NA | NA | NA |
| OTU8078 | NA | NA | NA | NA |
| OTU14952 | NA | NA | NA | NA |
| OTU15241 | NA | NA | NA | NA |
| OTU9992 | NA | NA | NA | NA |
| OTU9786 | NA | NA | NA | NA |
| OTU8450 | NA | NA | NA | NA |
| OTU2504 | NA | NA | NA | NA |
| OTU1872 | NA | NA | NA | NA |
| OTU6627 | + | NA | NA | NA |
| OTU5854 | NA | NA | NA | NA |
| OTU6233 | NA | NA | NA | NA |
| OTU13423 | NA | NA | NA | NA |
| OTU16676 | NA | NA | NA | NA |
| OTU17204 | NA | NA | NA | NA |
| OTU2188 | + | NA | NA | NA |
| OTU13265 | NA | NA | NA | NA |
| OTU7140 | NA | NA | NA | NA |
| OTU17349 | NA | NA | NA | NA |
| OTU10486 | NA | NA | NA | NA |
| OTU7007 | NA | NA | NA | NA |
| OTU3078 | + | NA | NA | NA |
| OTU16062 | NA | NA | NA | NA |
| OTU13445 | NA | NA | NA | NA |
| OTU5212 | NA | NA | + | NA |
| OTU7506 | NA | NA | NA | NA |
| OTU6885 | NA | NA | NA | NA |
| OTU12419 | NA | NA | + | NA |
| OTU1605 | + | NA | NA | NA |
| OTU7019 | NA | NA | NA | NA |
| OTU9017 | NA | NA | NA | - |
| OTU6483 | NA | NA | NA | NA |
| OTU6040 | NA | NA | NA | NA |
| OTU9456 | NA | NA | NA | NA |
| OTU6681 | + | NA | NA | NA |
| OTU286 | NA | NA | NA | NA |
| OTU5005 | NA | NA | NA | NA |
| OTU16495 | NA | NA | NA | NA |
| OTU1892 | NA | NA | NA | NA |
| OTU11745 | NA | NA | NA | + |
| OTU542 | NA | NA | NA | NA |
| OTU5176 | NA | NA | NA | NA |
| OTU8490 | NA | NA | NA | NA |
| OTU9528 | + | NA | NA | NA |
| OTU8141 | NA | NA | NA | NA |
| OTU8290 | NA | NA | NA | NA |
| OTU16337 | NA | NA | NA | + |
| OTU5691 | NA | NA | NA | NA |
| OTU4725 | NA | NA | NA | NA |
| OTU2635 | + | NA | NA | NA |
| OTU18643 | + | NA | NA | NA |
| OTU3510 | NA | NA | NA | NA |
| OTU16080 | NA | NA | NA | + |
| OTU36 | + | NA | NA | NA |
| OTU2469 | NA | - | NA | NA |
| OTU4976 | NA | NA | NA | NA |
| OTU15942 | NA | NA | NA | NA |
| OTU12729 | NA | NA | NA | NA |
| OTU16159 | NA | NA | NA | NA |
| OTU12740 | NA | NA | + | NA |
| OTU16751 | NA | NA | NA | NA |
| OTU5885 | NA | NA | NA | NA |
| OTU8477 | NA | NA | NA | NA |
| OTU592 | + | NA | NA | NA |
| OTU8519 | NA | + | NA | NA |
| OTU1952 | NA | NA | NA | NA |
| OTU1787 | NA | NA | NA | + |
| OTU630 | NA | NA | NA | NA |
| OTU4648 | NA | NA | NA | NA |
| OTU18962 | NA | NA | NA | NA |
| OTU13418 | + | - | NA | NA |
| OTU13524 | NA | NA | NA | NA |
| OTU8247 | NA | NA | NA | NA |
| OTU9859 | NA | NA | NA | NA |
| OTU18502 | + | NA | NA | NA |
| OTU6511 | NA | NA | NA | NA |
| OTU1826 | + | NA | NA | NA |
| OTU15803 | NA | NA | NA | + |
| OTU5752 | NA | NA | NA | NA |
| OTU11055 | NA | NA | NA | NA |
| OTU9186 | + | NA | NA | NA |
| OTU16263 | NA | NA | NA | NA |
| OTU5100 | NA | NA | NA | NA |
| OTU12240 | NA | NA | NA | NA |
| OTU4905 | NA | NA | NA | NA |
| OTU14400 | NA | NA | + | NA |
| OTU15432 | NA | NA | NA | NA |
| OTU4446 | + | NA | NA | NA |
| OTU8509 | NA | NA | NA | NA |
| OTU16992 | NA | NA | NA | NA |
| OTU16983 | NA | NA | NA | NA |
| OTU175 | NA | NA | NA | NA |
| OTU17564 | NA | NA | NA | + |
| OTU15704 | NA | NA | NA | NA |
| OTU1114 | NA | NA | NA | NA |
| OTU5845 | NA | NA | NA | NA |
| OTU9943 | NA | NA | NA | NA |
| OTU8965 | NA | NA | NA | NA |
| OTU10587 | NA | NA | NA | NA |
| OTU9845 | NA | NA | NA | NA |
| OTU15224 | NA | NA | NA | + |
| OTU4471 | + | NA | NA | NA |
| OTU17023 | + | NA | NA | NA |
| OTU6665 | NA | NA | NA | NA |
| OTU5426 | NA | NA | NA | NA |
| OTU15983 | NA | NA | NA | NA |
| OTU16299 | NA | NA | NA | NA |
| OTU2211 | NA | NA | NA | NA |
| OTU18604 | NA | NA | NA | NA |
| OTU1429 | + | NA | NA | NA |
| OTU6557 | NA | NA | NA | NA |
| OTU11821 | NA | NA | NA | NA |
| OTU8932 | NA | NA | NA | NA |
| OTU5938 | NA | NA | NA | NA |
| OTU8142 | NA | NA | NA | NA |
| OTU15322 | + | NA | NA | NA |
| OTU18749 | NA | NA | NA | NA |
| OTU10427 | NA | NA | NA | NA |
| OTU54 | NA | + | NA | NA |
| OTU16110 | NA | NA | NA | NA |
| OTU15435 | NA | NA | NA | + |
| OTU16378 | NA | NA | NA | NA |
| OTU4799 | NA | NA | NA | NA |
| OTU5160 | NA | NA | NA | NA |
| OTU11518 | NA | NA | + | NA |
| OTU8725 | + | NA | NA | NA |
| OTU2991 | NA | NA | NA | NA |
| OTU11149 | NA | NA | NA | NA |
| OTU5812 | + | NA | NA | NA |
| OTU8985 | NA | NA | NA | NA |
| OTU15940 | NA | NA | NA | NA |
| OTU16305 | NA | NA | NA | NA |
| OTU417 | NA | NA | NA | NA |
| OTU8088 | NA | NA | NA | NA |
| OTU17731 | NA | NA | NA | NA |
| OTU17020 | NA | NA | NA | NA |
| OTU3847 | NA | NA | NA | NA |
| OTU12816 | NA | NA | NA | NA |
| OTU15400 | NA | NA | NA | NA |
| OTU18573 | NA | NA | NA | NA |
| OTU18578 | + | NA | NA | NA |
| OTU7561 | NA | NA | NA | NA |
| OTU4867 | NA | NA | NA | NA |
| OTU9672 | NA | NA | NA | NA |
| OTU18932 | NA | NA | NA | + |
| OTU8829 | NA | NA | NA | NA |
| OTU4441 | + | NA | NA | NA |
| OTU4445 | + | NA | NA | NA |
| OTU15226 | NA | NA | NA | + |
| OTU6851 | + | NA | NA | NA |
| OTU1677 | NA | NA | NA | NA |
| OTU8432 | NA | NA | NA | NA |
| OTU16939 | NA | NA | NA | NA |
| OTU7079 | NA | NA | NA | NA |
| OTU710 | NA | NA | NA | NA |
| OTU2339 | + | NA | NA | NA |
| OTU12571 | NA | NA | NA | NA |
| OTU16613 | NA | NA | + | NA |
| OTU16237 | + | NA | NA | NA |
| OTU5955 | NA | NA | NA | NA |
| OTU13598 | NA | NA | + | NA |
| OTU9563 | NA | NA | NA | NA |
| OTU6488 | NA | NA | NA | NA |
| OTU1485 | + | NA | NA | NA |
| OTU8840 | NA | NA | NA | NA |
| OTU3765 | + | NA | NA | NA |
| OTU15806 | NA | NA | NA | NA |
| OTU11974 | NA | NA | NA | NA |
| OTU6799 | NA | NA | NA | NA |
| OTU5552 | NA | NA | NA | NA |
| OTU6555 | NA | NA | NA | NA |
| OTU6259 | + | NA | NA | NA |
| OTU10803 | NA | NA | NA | NA |
| OTU18529 | NA | NA | NA | NA |
| OTU17666 | NA | NA | NA | NA |
| OTU6367 | NA | NA | NA | NA |
| OTU16218 | NA | NA | NA | NA |
| OTU18828 | NA | NA | NA | NA |
| OTU16240 | NA | NA | NA | NA |
| OTU16665 | NA | NA | NA | + |
| OTU12832 | NA | NA | NA | NA |
| OTU7043 | NA | NA | NA | NA |
| OTU19131 | NA | NA | NA | NA |
| OTU18842 | NA | NA | NA | NA |
| OTU15843 | NA | NA | + | NA |
| OTU11318 | NA | NA | + | NA |
| OTU15618 | NA | NA | NA | NA |
| OTU13559 | + | NA | NA | NA |
| OTU1313 | NA | NA | NA | NA |
| OTU16604 | NA | NA | NA | NA |
| OTU4288 | NA | NA | NA | NA |
| OTU1712 | NA | NA | NA | - |
| OTU2725 | NA | NA | NA | NA |
| OTU7778 | NA | + | NA | NA |
| OTU9469 | NA | NA | NA | NA |
| OTU16413 | NA | NA | NA | NA |
| OTU8500 | NA | NA | NA | NA |
| OTU1058 | NA | NA | NA | NA |
| OTU13485 | NA | NA | NA | NA |
| OTU11754 | + | NA | NA | NA |
| OTU4427 | NA | NA | NA | NA |
| OTU2239 | NA | NA | NA | NA |
| OTU3769 | NA | NA | NA | NA |
| OTU12176 | + | NA | NA | NA |
| OTU6241 | NA | NA | NA | NA |
| OTU6551 | NA | NA | NA | NA |
| OTU7365 | NA | NA | NA | NA |
| OTU960 | NA | NA | NA | NA |
| OTU2922 | + | NA | NA | NA |
| OTU4148 | NA | NA | NA | NA |
| OTU11853 | NA | NA | NA | NA |
| OTU7195 | NA | NA | NA | NA |
| OTU1469 | NA | NA | NA | NA |
| OTU12534 | NA | NA | NA | NA |
| OTU5695 | + | NA | NA | NA |
| OTU1310 | NA | NA | NA | + |
| OTU6930 | NA | NA | NA | NA |
| OTU17374 | NA | NA | NA | NA |
| OTU10601 | NA | NA | + | NA |
| OTU17191 | NA | NA | NA | NA |
| OTU8958 | NA | NA | NA | NA |
| OTU7790 | NA | NA | NA | NA |
| OTU12063 | NA | NA | NA | NA |
| OTU4172 | NA | NA | NA | NA |
| OTU10386 | NA | NA | NA | NA |
| OTU188 | NA | NA | NA | NA |
| OTU11743 | NA | NA | NA | NA |
| OTU379 | NA | NA | NA | NA |
| OTU12766 | NA | NA | NA | NA |
| OTU16609 | NA | NA | NA | NA |
| OTU11830 | NA | NA | NA | NA |
| OTU9488 | NA | NA | NA | NA |
| OTU4886 | NA | + | NA | NA |
| OTU12237 | NA | NA | NA | NA |
| OTU17339 | NA | NA | NA | NA |
| OTU16327 | NA | NA | NA | + |
| OTU3783 | NA | NA | NA | NA |
| OTU1134 | + | NA | NA | NA |
| OTU8989 | NA | NA | NA | NA |
| OTU18493 | NA | NA | + | NA |
| OTU7318 | NA | NA | NA | NA |
| OTU949 | NA | NA | NA | NA |
| OTU5796 | NA | NA | NA | NA |
| OTU4111 | NA | NA | NA | NA |
| OTU5418 | NA | + | NA | NA |
| OTU10678 | NA | NA | NA | NA |
| OTU15052 | NA | NA | NA | NA |
| OTU14491 | NA | NA | NA | NA |
| OTU4929 | NA | NA | NA | NA |
| OTU12073 | NA | NA | NA | NA |
| OTU1441 | NA | NA | NA | NA |
| OTU8567 | NA | NA | NA | NA |
| OTU10383 | NA | NA | + | NA |
| OTU11301 | NA | NA | NA | NA |
| OTU16701 | NA | NA | NA | NA |
| OTU16293 | NA | NA | NA | NA |
| OTU9246 | NA | NA | NA | NA |
| OTU213 | NA | NA | NA | NA |
| OTU11052 | NA | NA | NA | NA |
| OTU18763 | + | NA | NA | NA |
| OTU581 | NA | NA | + | NA |
| OTU6048 | NA | NA | NA | NA |
| OTU1587 | NA | NA | NA | NA |
| OTU13422 | NA | NA | + | NA |
| OTU18515 | NA | NA | NA | NA |
| OTU4273 | NA | NA | NA | - |
| OTU479 | NA | NA | NA | NA |
| OTU5117 | NA | NA | NA | NA |
| OTU6861 | NA | NA | NA | NA |
| OTU11616 | NA | NA | NA | NA |
| OTU9800 | NA | NA | NA | NA |
| OTU3168 | NA | NA | NA | NA |
| OTU9217 | NA | NA | NA | NA |
| OTU1466 | NA | NA | NA | NA |
| OTU7366 | + | NA | NA | NA |
| OTU10688 | NA | NA | NA | NA |
| OTU2937 | NA | NA | NA | NA |
| OTU4070 | NA | + | NA | NA |
| OTU16161 | NA | NA | + | NA |
| OTU1982 | NA | NA | NA | NA |
| OTU2665 | + | NA | NA | NA |
| OTU9719 | NA | NA | NA | NA |
| OTU366 | NA | NA | NA | NA |
| OTU2873 | NA | NA | + | NA |
| OTU5518 | NA | NA | NA | NA |
| OTU16018 | NA | NA | NA | + |
| OTU15423 | + | NA | NA | NA |
| OTU18950 | NA | NA | NA | NA |
| OTU11708 | NA | NA | NA | NA |
| OTU8165 | + | NA | NA | NA |
| OTU1128 | NA | NA | NA | + |
| OTU3322 | NA | NA | NA | NA |
| OTU5200 | NA | NA | NA | NA |
| OTU7342 | NA | NA | NA | NA |
| OTU8020 | NA | NA | NA | NA |
| OTU16244 | NA | NA | NA | NA |
| OTU18935 | NA | NA | NA | NA |
| OTU3330 | NA | NA | NA | NA |
| OTU4320 | + | NA | NA | NA |
| OTU6648 | NA | NA | NA | NA |
| OTU16639 | + | NA | NA | NA |
| OTU9517 | NA | NA | NA | NA |
| OTU6517 | NA | NA | NA | NA |
| OTU8583 | NA | + | NA | NA |
| OTU3638 | NA | NA | NA | NA |
| OTU9674 | NA | NA | NA | NA |
| OTU5056 | NA | NA | NA | + |
| OTU9552 | NA | NA | NA | NA |
| OTU11117 | NA | NA | NA | NA |
| OTU7237 | NA | NA | NA | NA |
| OTU2842 | + | NA | NA | NA |
| OTU6863 | NA | NA | NA | NA |
| OTU15445 | NA | NA | NA | + |
| OTU5782 | NA | NA | NA | NA |
| OTU16461 | NA | NA | NA | NA |
| OTU4159 | NA | NA | NA | NA |
| OTU1468 | NA | NA | NA | NA |
| OTU8944 | NA | NA | NA | NA |
| OTU18615 | NA | NA | NA | NA |
| OTU9998 | NA | NA | NA | NA |
| OTU18594 | NA | NA | NA | NA |
| OTU4531 | NA | NA | NA | NA |
| OTU7962 | NA | NA | NA | NA |
| OTU11846 | NA | NA | NA | NA |
| OTU12418 | + | NA | NA | NA |
| OTU9334 | NA | + | NA | NA |
| OTU15348 | NA | NA | NA | + |
| OTU18435 | NA | NA | NA | NA |
| OTU8653 | NA | NA | NA | NA |
| OTU10237 | NA | NA | NA | NA |
| OTU16652 | NA | NA | NA | + |
| OTU15558 | NA | NA | NA | NA |
| OTU5886 | NA | NA | NA | NA |
| OTU6713 | + | NA | NA | NA |
| OTU1138 | + | NA | NA | NA |
| OTU16131 | NA | NA | NA | NA |
| OTU2831 | + | NA | NA | NA |
| OTU3368 | + | NA | NA | NA |
| OTU10421 | NA | NA | NA | NA |
| OTU4416 | NA | NA | + | NA |
| OTU214 | + | NA | NA | NA |
| OTU3873 | NA | NA | NA | + |
| OTU13281 | NA | NA | + | NA |
| OTU10582 | NA | NA | NA | NA |
| OTU6338 | NA | NA | NA | NA |
| OTU17136 | NA | NA | NA | NA |
| OTU12579 | + | NA | NA | NA |
| OTU1040 | + | NA | NA | NA |
| OTU16523 | + | NA | NA | NA |
| OTU15914 | NA | NA | NA | + |
| OTU3365 | + | NA | NA | NA |
| OTU3462 | NA | NA | NA | NA |
| OTU8399 | + | NA | NA | NA |
| OTU9404 | + | NA | NA | NA |
| OTU12244 | + | - | NA | NA |
| OTU9754 | NA | NA | NA | NA |
| OTU1812 | NA | NA | NA | - |
| OTU9936 | NA | NA | NA | NA |
| OTU20 | NA | NA | NA | NA |
| OTU17031 | NA | NA | NA | NA |
| OTU11312 | NA | NA | NA | NA |
| OTU10594 | NA | NA | + | NA |
| OTU3571 | + | NA | NA | NA |
| OTU8120 | NA | NA | NA | NA |
| OTU2404 | + | NA | NA | NA |
| OTU7741 | + | NA | NA | NA |
| OTU18792 | NA | NA | NA | NA |
| OTU15510 | NA | NA | NA | + |
| OTU18556 | + | NA | NA | NA |
| OTU4467 | + | NA | NA | NA |
| OTU1103 | NA | NA | NA | NA |
| OTU10423 | NA | NA | + | NA |
| OTU537 | NA | NA | NA | + |
| OTU13020 | NA | NA | NA | NA |
| OTU1464 | NA | NA | NA | NA |
| OTU4831 | NA | + | NA | NA |
| OTU8311 | NA | NA | NA | NA |
| OTU18945 | NA | NA | NA | NA |
| OTU2615 | + | NA | NA | NA |
| OTU16332 | NA | NA | NA | NA |
| OTU4982 | NA | NA | + | NA |
| OTU14971 | NA | NA | + | NA |
| OTU4058 | NA | NA | NA | + |
| OTU10737 | NA | NA | NA | NA |
| OTU9752 | NA | NA | NA | NA |
| OTU9533 | NA | NA | NA | NA |
| OTU1161 | NA | NA | NA | NA |
| OTU16566 | NA | NA | NA | NA |
| OTU12941 | NA | NA | NA | - |
| OTU12366 | NA | NA | NA | + |
| OTU12741 | NA | NA | + | NA |
| OTU483 | + | NA | NA | NA |
| OTU14023 | NA | NA | NA | NA |
| OTU12201 | NA | NA | NA | NA |
| OTU6313 | NA | NA | NA | NA |
| OTU12198 | NA | NA | + | NA |
| OTU12017 | NA | NA | NA | NA |
| OTU80 | + | NA | NA | NA |
| OTU9083 | NA | NA | NA | NA |
| OTU10514 | NA | NA | NA | NA |
| OTU3376 | NA | NA | NA | NA |
| OTU6129 | NA | NA | NA | NA |
| OTU12210 | + | NA | NA | NA |
| OTU4482 | + | NA | NA | NA |
| OTU2308 | NA | NA | NA | NA |
| OTU12636 | NA | - | NA | NA |
| OTU4050 | NA | NA | NA | NA |
| OTU4488 | NA | NA | NA | NA |
| OTU5186 | NA | NA | NA | + |
| OTU2182 | + | NA | NA | NA |
| OTU1874 | NA | NA | NA | NA |
| OTU5585 | NA | NA | + | NA |
| OTU13750 | NA | NA | NA | NA |
| OTU15197 | NA | NA | NA | NA |
| OTU16078 | NA | NA | NA | NA |
| OTU4020 | NA | + | NA | NA |
| OTU4731 | NA | NA | + | NA |
| OTU5099 | NA | NA | NA | NA |
| OTU8345 | NA | NA | NA | NA |
| OTU5150 | NA | NA | NA | NA |
| OTU9522 | + | NA | NA | NA |
| OTU123 | NA | NA | NA | NA |
| OTU6558 | + | NA | NA | NA |
| OTU8788 | + | NA | NA | NA |
| OTU13457 | NA | NA | NA | NA |
| OTU1029 | + | NA | NA | NA |
| OTU12095 | + | NA | NA | - |
| OTU18488 | NA | NA | NA | NA |
| OTU10731 | NA | NA | NA | NA |
| OTU10039 | NA | NA | NA | NA |
| OTU7734 | NA | NA | NA | NA |
| OTU7263 | NA | NA | NA | NA |
| OTU8083 | NA | NA | NA | NA |
| OTU7053 | NA | NA | NA | NA |
| OTU3112 | NA | NA | NA | NA |
| OTU18982 | NA | NA | NA | NA |
| OTU1475 | NA | NA | NA | NA |
| OTU11233 | NA | NA | NA | NA |
| OTU4760 | NA | NA | NA | NA |
| OTU8806 | + | NA | NA | NA |
| OTU19070 | NA | NA | NA | NA |
| OTU3097 | NA | NA | NA | NA |
| OTU1051 | NA | NA | NA | NA |
| OTU6664 | NA | NA | NA | NA |
| OTU2546 | + | NA | NA | NA |
| OTU11879 | NA | NA | NA | NA |
| OTU12969 | NA | + | NA | NA |
| OTU18614 | NA | NA | NA | NA |
| OTU8732 | NA | NA | NA | NA |
| OTU4806 | NA | NA | NA | NA |
| OTU18524 | NA | NA | NA | NA |
| OTU9509 | + | NA | NA | NA |
| OTU13506 | NA | NA | NA | NA |
| OTU16969 | NA | NA | NA | NA |
| OTU15882 | NA | NA | NA | NA |
| OTU667 | + | NA | NA | NA |
| OTU3118 | + | NA | NA | NA |
| OTU7912 | + | NA | NA | NA |
| OTU15169 | NA | NA | NA | + |
| OTU6249 | NA | NA | NA | NA |
| OTU8355 | NA | NA | NA | NA |
| OTU10 | NA | NA | NA | NA |
| OTU1016 | + | NA | NA | NA |
| OTU2849 | NA | NA | NA | NA |
| OTU7775 | NA | NA | NA | NA |
| OTU11531 | + | NA | NA | NA |
| OTU12402 | NA | NA | NA | NA |
| OTU10492 | NA | NA | NA | NA |
| OTU17474 | NA | NA | NA | + |
| OTU12144 | NA | NA | NA | NA |
| OTU6541 | NA | + | NA | NA |
| OTU16587 | NA | NA | NA | NA |
| OTU12820 | NA | NA | NA | NA |
| OTU11190 | NA | NA | NA | NA |
| OTU4959 | NA | NA | NA | - |
| OTU13037 | + | NA | + | NA |
| OTU16472 | NA | NA | NA | NA |
| OTU16787 | NA | NA | NA | + |
| OTU7194 | + | NA | NA | NA |
| OTU8250 | + | NA | NA | NA |
| OTU12809 | NA | NA | + | NA |
| OTU16132 | NA | NA | NA | NA |
| OTU4190 | NA | NA | NA | NA |
| OTU5331 | NA | NA | NA | NA |
| OTU17353 | NA | NA | NA | + |
| OTU18662 | + | NA | NA | NA |
| OTU2786 | NA | NA | NA | NA |
| OTU1442 | NA | NA | NA | NA |
| OTU8756 | NA | NA | NA | NA |
| OTU5343 | NA | NA | NA | NA |
| OTU2886 | + | NA | NA | NA |
| OTU17178 | NA | NA | NA | NA |
| OTU11130 | NA | NA | NA | NA |
| OTU5134 | NA | NA | NA | NA |
| OTU4941 | NA | NA | NA | NA |
| OTU9088 | + | NA | NA | NA |
| OTU18886 | + | NA | NA | NA |
| OTU19001 | NA | NA | NA | NA |
| OTU8523 | + | NA | NA | NA |
| OTU3420 | NA | NA | NA | NA |
| OTU6904 | NA | + | NA | NA |
| OTU11213 | NA | NA | NA | NA |
| OTU11629 | NA | NA | NA | NA |
| OTU14201 | NA | NA | NA | NA |
| OTU8234 | NA | NA | NA | NA |
| OTU12713 | NA | NA | NA | NA |
| OTU139 | + | NA | NA | NA |
| OTU8611 | NA | NA | NA | NA |
| OTU10885 | NA | NA | NA | NA |
| OTU10919 | NA | NA | NA | NA |
| OTU15542 | NA | NA | NA | NA |
| OTU17053 | NA | NA | NA | NA |
| OTU2326 | NA | NA | NA | NA |
| OTU16092 | NA | NA | NA | NA |
| OTU10472 | NA | NA | NA | NA |
| OTU7338 | + | NA | NA | NA |
| OTU18800 | NA | NA | NA | NA |
| OTU10703 | NA | NA | NA | NA |
| OTU4077 | + | NA | NA | NA |
| OTU9755 | NA | NA | + | NA |
| OTU14361 | NA | NA | NA | NA |
| OTU8907 | + | NA | NA | NA |
| OTU15856 | NA | NA | NA | NA |
| OTU16140 | NA | NA | NA | NA |
| OTU9338 | NA | NA | NA | NA |
| OTU14259 | NA | NA | NA | NA |
| OTU4127 | NA | NA | NA | NA |
| OTU5495 | NA | NA | NA | NA |
| OTU16913 | NA | NA | NA | NA |
| OTU10340 | NA | NA | NA | NA |
| OTU9705 | NA | NA | NA | NA |
| OTU18571 | NA | NA | NA | NA |
| OTU6829 | NA | + | NA | NA |
| OTU11705 | NA | NA | NA | NA |
| OTU10494 | NA | NA | NA | NA |
| OTU12818 | NA | NA | NA | NA |
| OTU3792 | NA | NA | NA | NA |
| OTU16610 | NA | NA | NA | NA |
| OTU4013 | + | NA | NA | NA |
| OTU9443 | + | NA | NA | NA |
| OTU19135 | NA | NA | NA | NA |
| OTU7303 | NA | NA | NA | NA |
| OTU8885 | NA | + | NA | NA |
| OTU11364 | + | NA | NA | NA |
| OTU12417 | NA | NA | NA | NA |
| OTU18061 | NA | NA | NA | NA |
| OTU155 | + | NA | NA | NA |
| OTU12564 | NA | NA | NA | NA |
| OTU7478 | NA | NA | NA | NA |
| OTU12049 | NA | NA | NA | NA |
| OTU18931 | NA | NA | NA | NA |
| OTU5828 | NA | NA | NA | NA |
| OTU12931 | NA | NA | NA | NA |
| OTU11510 | NA | NA | NA | NA |
| OTU12553 | NA | NA | NA | NA |
| OTU16925 | NA | NA | + | NA |
| OTU18246 | NA | NA | NA | NA |
| OTU16114 | NA | NA | NA | NA |
| OTU2761 | + | NA | NA | NA |
| OTU3061 | + | NA | NA | NA |
| OTU5259 | NA | NA | NA | NA |
| OTU4156 | NA | NA | NA | NA |
| OTU1698 | + | NA | NA | NA |
| OTU4434 | NA | NA | NA | + |
| OTU16623 | NA | NA | NA | + |
| OTU4041 | NA | NA | NA | NA |
| OTU15038 | NA | NA | NA | NA |
| OTU7915 | NA | NA | NA | NA |
| OTU478 | + | NA | NA | NA |
| OTU11515 | + | NA | NA | NA |
| OTU4465 | + | NA | NA | NA |
| OTU3587 | NA | NA | NA | NA |
| OTU6182 | NA | NA | NA | NA |
| OTU3554 | NA | NA | NA | NA |
| OTU4762 | NA | NA | NA | NA |
| OTU4145 | NA | NA | NA | NA |
| OTU304 | + | NA | NA | NA |
| OTU8306 | NA | NA | NA | NA |
| OTU9129 | NA | NA | NA | NA |
| OTU17266 | NA | NA | NA | NA |
| OTU12645 | NA | NA | NA | NA |
| OTU14709 | + | NA | NA | NA |
| OTU3306 | NA | NA | NA | NA |
| OTU10310 | NA | - | NA | NA |
| OTU17037 | NA | NA | NA | NA |
| OTU16273 | NA | NA | NA | NA |
| OTU11568 | NA | NA | NA | + |
| OTU12912 | NA | NA | NA | NA |
| OTU9466 | + | NA | NA | NA |
| OTU7165 | NA | NA | NA | NA |
| OTU4940 | NA | NA | NA | NA |
| OTU12454 | NA | NA | NA | NA |
| OTU6613 | + | NA | NA | NA |
| OTU7435 | NA | NA | NA | NA |
| OTU3465 | + | NA | NA | NA |
| OTU1086 | NA | NA | NA | NA |
| OTU9136 | NA | NA | NA | NA |
| OTU1909 | NA | NA | NA | NA |
| OTU9352 | NA | + | NA | NA |
| OTU3312 | NA | NA | NA | NA |
| OTU19201 | + | NA | NA | NA |
| OTU3786 | NA | NA | NA | NA |
| OTU16664 | NA | NA | NA | NA |
| OTU13259 | + | NA | NA | NA |
| OTU17043 | NA | NA | NA | NA |
| OTU1024 | NA | NA | NA | NA |
| OTU5396 | NA | NA | NA | NA |
| OTU709 | + | NA | NA | NA |
| OTU6341 | NA | NA | NA | NA |
| OTU841 | NA | NA | NA | NA |
| OTU5163 | NA | NA | NA | NA |
| OTU11078 | NA | NA | NA | NA |
| OTU3263 | + | NA | NA | NA |
| OTU7169 | NA | NA | NA | NA |
| OTU10993 | NA | NA | NA | NA |
| OTU10725 | NA | NA | NA | NA |
| OTU9929 | NA | NA | NA | NA |
| OTU7325 | NA | NA | NA | NA |
| OTU10004 | NA | NA | NA | NA |
| OTU1095 | NA | NA | NA | NA |
| OTU10576 | NA | NA | + | NA |
| OTU16963 | NA | NA | NA | NA |
| OTU8409 | NA | NA | NA | NA |
| OTU13242 | NA | NA | NA | NA |
| OTU2824 | NA | NA | NA | NA |
| OTU8754 | + | NA | NA | NA |
| OTU12815 | NA | NA | NA | NA |
| OTU4951 | NA | NA | NA | NA |
| OTU504 | NA | NA | NA | NA |
| OTU8475 | NA | NA | NA | NA |
| OTU10898 | NA | NA | NA | NA |
| OTU11451 | NA | NA | NA | NA |
| OTU446 | NA | NA | NA | NA |
| OTU6526 | NA | NA | NA | NA |
| OTU9838 | NA | NA | NA | NA |
| OTU9769 | NA | NA | NA | NA |
| OTU2249 | NA | NA | NA | NA |
| OTU6804 | NA | NA | NA | NA |
| OTU17283 | NA | NA | NA | NA |
| OTU18952 | NA | NA | NA | NA |
| OTU15537 | NA | NA | NA | NA |
| OTU1431 | + | NA | NA | NA |
| OTU10821 | NA | NA | NA | NA |
| OTU8766 | NA | NA | NA | NA |
| OTU819 | + | NA | NA | NA |
| OTU15163 | NA | NA | NA | NA |
| OTU16508 | NA | NA | NA | NA |
| OTU11112 | + | NA | NA | NA |
| OTU5967 | NA | NA | NA | NA |
| OTU10223 | NA | NA | NA | NA |
| OTU15805 | NA | NA | NA | + |
| OTU2009 | NA | NA | NA | NA |
| OTU14019 | NA | NA | NA | NA |
| OTU1571 | NA | NA | NA | NA |
| OTU12583 | NA | NA | NA | NA |
| OTU255 | NA | NA | NA | NA |
| OTU6179 | NA | + | NA | NA |
| OTU7718 | NA | NA | NA | NA |
| OTU17252 | NA | NA | NA | NA |
| OTU10252 | NA | NA | NA | NA |
| OTU16791 | NA | NA | NA | NA |
| OTU3181 | NA | NA | NA | NA |
| OTU7933 | NA | NA | NA | NA |
| OTU13006 | NA | NA | NA | NA |
| OTU11996 | NA | NA | + | - |
| OTU13093 | NA | NA | NA | NA |
| OTU2475 | NA | NA | NA | NA |
| OTU15714 | NA | NA | NA | NA |
| OTU5990 | NA | NA | NA | NA |
| OTU6781 | NA | NA | NA | NA |
| OTU7 | + | NA | NA | NA |
| OTU8824 | NA | NA | NA | NA |
| OTU11983 | NA | NA | + | NA |
| OTU1165 | NA | NA | NA | NA |
| OTU4497 | NA | NA | NA | NA |
| OTU2604 | NA | NA | NA | NA |
| OTU9454 | + | NA | NA | NA |
| OTU18447 | NA | NA | NA | NA |
| OTU1395 | NA | NA | NA | NA |
| OTU11455 | + | NA | NA | NA |
| OTU686 | NA | NA | NA | NA |
| OTU6961 | NA | NA | + | NA |
| OTU4161 | NA | NA | NA | NA |
| OTU15730 | NA | NA | NA | NA |
| OTU16497 | NA | NA | NA | NA |
| OTU16071 | NA | NA | NA | NA |
| OTU6121 | NA | NA | NA | NA |
| OTU13503 | NA | NA | NA | NA |
| OTU17030 | + | NA | NA | NA |
| OTU1248 | NA | NA | NA | NA |
| OTU10454 | NA | NA | NA | NA |
| OTU5303 | NA | NA | NA | NA |
| OTU10597 | NA | NA | NA | NA |
| OTU18707 | NA | NA | NA | NA |
| OTU16401 | NA | NA | NA | NA |
| OTU4278 | + | NA | NA | NA |
| OTU16136 | NA | NA | NA | NA |
| OTU11649 | NA | NA | NA | NA |
| OTU16334 | NA | NA | NA | NA |
| OTU11876 | NA | NA | NA | NA |
| OTU4813 | NA | NA | NA | NA |
| OTU18739 | NA | NA | NA | NA |
| OTU10328 | NA | NA | NA | NA |
| OTU11715 | NA | NA | NA | NA |
| OTU12938 | NA | NA | NA | NA |
| OTU12019 | NA | NA | NA | NA |
| OTU16207 | NA | NA | NA | NA |
| OTU8525 | NA | + | NA | NA |
| OTU9512 | NA | NA | NA | NA |
| OTU12960 | + | NA | NA | NA |
| OTU4926 | NA | NA | NA | NA |
| OTU3270 | + | NA | NA | NA |
| OTU11849 | NA | NA | NA | NA |
| OTU7614 | NA | NA | NA | NA |
| OTU2644 | + | NA | NA | NA |
| OTU1262 | + | NA | NA | NA |
| OTU3784 | NA | - | NA | + |
| OTU18475 | NA | NA | NA | NA |
| OTU9276 | NA | NA | NA | NA |
| OTU9288 | NA | NA | NA | NA |
| OTU12014 | NA | NA | NA | NA |
| OTU13404 | NA | NA | NA | NA |
| OTU9599 | NA | NA | NA | NA |
| OTU8752 | NA | NA | + | NA |
| OTU16649 | NA | + | NA | NA |
| OTU18417 | NA | NA | NA | + |
| OTU130 | NA | NA | NA | NA |
| OTU12959 | + | NA | NA | NA |
| OTU2356 | + | NA | NA | NA |
| OTU11837 | NA | NA | NA | NA |
| OTU16822 | NA | NA | NA | NA |
| OTU10794 | NA | NA | NA | NA |
| OTU13695 | NA | NA | NA | NA |
| OTU6791 | + | NA | NA | NA |
| OTU11747 | NA | NA | NA | NA |
| OTU3862 | NA | NA | NA | NA |
| OTU11982 | NA | NA | NA | NA |
| OTU1283 | + | NA | NA | NA |
| OTU15950 | NA | NA | NA | NA |
| OTU15245 | NA | NA | NA | NA |
| OTU5736 | NA | NA | NA | NA |
| OTU7215 | NA | NA | NA | NA |
| OTU551 | NA | NA | NA | + |
| OTU8191 | NA | NA | NA | NA |
| OTU7925 | NA | NA | NA | NA |
| OTU11891 | NA | NA | NA | NA |
| OTU2668 | + | NA | NA | NA |
| OTU4681 | NA | NA | NA | NA |
| OTU4440 | NA | NA | NA | NA |
| OTU15371 | NA | NA | NA | NA |
| OTU3562 | NA | NA | NA | NA |
| OTU3602 | NA | NA | NA | NA |
| OTU298 | + | NA | NA | NA |
| OTU8222 | NA | NA | NA | NA |
| OTU15872 | NA | NA | NA | NA |
| OTU13955 | + | NA | NA | NA |
| OTU10533 | NA | NA | + | NA |
| OTU14928 | NA | NA | NA | NA |
| OTU5533 | NA | NA | NA | NA |
| OTU12697 | NA | NA | NA | NA |
| OTU4721 | NA | NA | NA | NA |
| OTU14421 | NA | NA | NA | NA |
| OTU14036 | NA | NA | NA | NA |
| OTU15590 | + | NA | NA | NA |
| OTU17377 | NA | NA | NA | + |
| OTU6839 | NA | NA | NA | NA |
| OTU10108 | NA | NA | NA | NA |
| OTU11037 | + | NA | NA | NA |
| OTU4096 | NA | NA | NA | NA |
| OTU16106 | + | NA | NA | NA |
| OTU9526 | + | NA | NA | NA |
| OTU5969 | NA | NA | NA | NA |
| OTU16536 | NA | NA | NA | NA |
| OTU9862 | + | NA | NA | NA |
| OTU7668 | NA | NA | NA | NA |
| OTU16827 | NA | NA | NA | NA |
| OTU4603 | NA | NA | NA | NA |
| OTU5907 | NA | NA | NA | NA |
| OTU10816 | NA | NA | NA | NA |
| OTU16432 | NA | NA | NA | + |
| OTU10581 | NA | NA | NA | NA |
| OTU9761 | NA | NA | NA | + |
| OTU2260 | + | NA | NA | NA |
| OTU9062 | NA | NA | NA | NA |
| OTU2923 | NA | NA | NA | NA |
| OTU3149 | NA | NA | NA | NA |
| OTU16600 | NA | NA | NA | NA |
| OTU2150 | NA | NA | NA | NA |
| OTU1380 | NA | NA | NA | NA |
| OTU5477 | NA | NA | NA | NA |
| OTU7225 | + | NA | NA | NA |
| OTU9069 | NA | NA | NA | NA |
| OTU15740 | NA | NA | NA | NA |
| OTU8708 | NA | NA | NA | NA |
| OTU10409 | NA | NA | NA | NA |
| OTU10002 | + | NA | NA | NA |
| OTU9433 | NA | NA | NA | NA |
| OTU6915 | NA | NA | NA | + |
| OTU6641 | NA | NA | NA | NA |
| OTU16194 | NA | NA | NA | NA |
| OTU6976 | NA | NA | NA | NA |
| OTU284 | + | NA | NA | NA |
| OTU4343 | + | NA | NA | NA |
| OTU4023 | NA | NA | NA | NA |
| OTU1741 | NA | NA | NA | NA |
| OTU13142 | NA | NA | NA | NA |
| OTU11266 | NA | NA | NA | NA |
| OTU15508 | NA | NA | NA | NA |
| OTU4067 | NA | NA | NA | NA |
| OTU15535 | NA | NA | NA | NA |
| OTU17246 | NA | NA | NA | NA |
| OTU2926 | NA | NA | NA | NA |
| OTU3849 | NA | NA | NA | NA |
| OTU4822 | NA | NA | NA | NA |
| OTU3649 | + | NA | NA | NA |
| OTU16103 | NA | NA | NA | NA |
| OTU3568 | NA | NA | NA | NA |
| OTU10442 | NA | NA | NA | NA |
| OTU8481 | NA | + | NA | NA |
| OTU2514 | + | NA | NA | NA |
| OTU13699 | NA | NA | NA | NA |
| OTU1089 | + | NA | NA | NA |
| OTU4821 | NA | NA | NA | NA |
| OTU3377 | + | NA | NA | NA |
| OTU9531 | + | NA | NA | NA |
| OTU9474 | + | NA | NA | NA |
| OTU5961 | NA | NA | NA | NA |
| OTU7292 | NA | + | NA | NA |
| OTU2216 | NA | NA | NA | NA |
| OTU5874 | NA | NA | NA | NA |
| OTU2433 | + | NA | NA | NA |
| OTU5225 | NA | NA | NA | NA |
| OTU13453 | NA | NA | NA | NA |
| OTU5385 | + | NA | NA | NA |
| OTU15349 | NA | NA | NA | + |
| OTU18659 | NA | NA | NA | NA |
| OTU18564 | NA | NA | NA | NA |
| OTU4152 | NA | NA | NA | NA |
| OTU9819 | + | NA | NA | NA |
| OTU9640 | NA | + | NA | NA |
| OTU12151 | NA | NA | + | NA |
| OTU15577 | NA | NA | NA | NA |
| OTU10502 | NA | - | NA | NA |
| OTU6888 | NA | NA | NA | NA |
| OTU6602 | NA | NA | NA | NA |
| OTU2277 | + | NA | NA | NA |
| OTU16422 | NA | NA | NA | NA |
| OTU10707 | NA | NA | NA | NA |
| OTU8486 | NA | NA | NA | + |
| OTU15658 | + | NA | NA | NA |
| OTU13608 | NA | NA | NA | NA |
| OTU3596 | NA | NA | NA | NA |
| OTU694 | NA | NA | NA | NA |
| OTU12914 | NA | NA | + | NA |
| OTU605 | + | NA | NA | NA |
| OTU4019 | NA | NA | NA | NA |
| OTU14043 | NA | NA | NA | NA |
| OTU5445 | NA | NA | NA | NA |
| OTU10766 | NA | NA | NA | NA |
| OTU8422 | + | NA | NA | NA |
| OTU13385 | NA | NA | NA | NA |
| OTU512 | + | NA | NA | NA |
| OTU9253 | NA | NA | NA | NA |
| OTU10191 | NA | NA | NA | NA |
| OTU2209 | NA | NA | NA | NA |
| OTU9921 | NA | + | NA | NA |
| OTU11396 | NA | NA | NA | NA |
| OTU6098 | NA | + | NA | NA |
| OTU16533 | NA | NA | NA | NA |
| OTU6946 | NA | NA | NA | NA |
| OTU10213 | NA | NA | + | NA |
| OTU3852 | NA | NA | NA | NA |
| OTU5665 | NA | NA | NA | NA |
| OTU5615 | NA | NA | NA | NA |
| OTU12114 | NA | NA | NA | + |
| OTU6750 | NA | NA | NA | NA |
| OTU13323 | NA | NA | NA | NA |
| OTU17162 | NA | NA | NA | NA |
| OTU257 | + | NA | NA | NA |
| OTU12573 | NA | NA | + | NA |
| OTU11330 | NA | NA | NA | NA |
| OTU12981 | NA | NA | NA | NA |
| OTU4751 | NA | NA | NA | NA |
| OTU11760 | NA | NA | NA | NA |
| OTU16386 | NA | NA | NA | NA |
| OTU11888 | NA | NA | NA | NA |
| OTU9046 | NA | NA | NA | NA |
| OTU8627 | NA | + | NA | NA |
| OTU5272 | NA | + | NA | NA |
| OTU12018 | NA | NA | NA | NA |
| OTU6519 | NA | NA | NA | NA |
| OTU9904 | NA | NA | NA | NA |
| OTU4872 | NA | NA | NA | NA |
| OTU4832 | NA | NA | NA | NA |
| OTU13236 | NA | NA | NA | NA |
| OTU11192 | NA | NA | NA | NA |
| OTU8335 | NA | NA | NA | NA |
| OTU1583 | NA | NA | NA | NA |
| OTU2954 | NA | NA | NA | NA |
| OTU18804 | NA | NA | NA | + |
| OTU3652 | + | NA | NA | NA |
| OTU3366 | NA | NA | NA | NA |
| OTU5555 | NA | NA | NA | NA |
| OTU10046 | NA | NA | NA | NA |
| OTU4417 | + | NA | NA | NA |
| OTU2268 | NA | NA | NA | NA |
| OTU7420 | NA | NA | NA | NA |
| OTU8871 | NA | NA | NA | NA |
| OTU11481 | NA | NA | NA | NA |
| OTU10478 | NA | NA | + | NA |
| OTU18559 | + | NA | NA | NA |
| OTU9274 | NA | NA | NA | NA |
| OTU8482 | NA | + | NA | NA |
| OTU6905 | NA | + | NA | NA |
| OTU10349 | + | NA | NA | NA |
| OTU8728 | + | NA | NA | NA |
| OTU14893 | NA | NA | NA | NA |
| OTU1353 | + | NA | NA | NA |
| OTU7238 | NA | NA | NA | NA |
| OTU2767 | NA | NA | NA | NA |
| OTU6133 | NA | NA | NA | NA |
| OTU3817 | NA | NA | + | NA |
| OTU14570 | NA | NA | NA | NA |
| OTU358 | NA | NA | NA | NA |
| OTU16812 | NA | NA | NA | NA |
| OTU4300 | + | NA | NA | NA |
| OTU635 | + | NA | NA | NA |
| OTU3027 | + | NA | NA | NA |
| OTU8881 | NA | + | NA | NA |
| OTU4777 | NA | NA | NA | NA |
| OTU11015 | NA | NA | + | - |
| OTU2511 | NA | NA | NA | NA |
| OTU8638 | NA | NA | NA | NA |
| OTU190 | NA | NA | NA | NA |
| OTU7078 | NA | NA | NA | NA |
| OTU2031 | NA | + | NA | NA |
| OTU790 | NA | NA | NA | NA |
| OTU16031 | NA | NA | NA | + |
| OTU5710 | NA | NA | NA | NA |
| OTU2825 | NA | NA | NA | NA |
| OTU8380 | NA | NA | NA | NA |
| OTU17090 | NA | NA | NA | NA |
| OTU2301 | NA | NA | NA | NA |
| OTU2409 | + | NA | NA | NA |
| OTU5410 | NA | NA | NA | NA |
| OTU1181 | + | NA | NA | NA |
| OTU11773 | NA | NA | NA | NA |
| OTU12251 | NA | NA | NA | NA |
| OTU11224 | NA | NA | NA | NA |
| OTU2217 | + | NA | NA | NA |
| OTU7158 | NA | + | NA | NA |
| OTU13151 | NA | NA | + | NA |
| OTU10717 | NA | NA | NA | NA |
| OTU4110 | NA | NA | + | NA |
| OTU19091 | + | NA | NA | NA |
| OTU2473 | NA | NA | NA | NA |
| OTU11216 | NA | NA | NA | NA |
| OTU4919 | NA | NA | NA | NA |
| OTU3163 | NA | NA | NA | NA |
| OTU8459 | NA | NA | NA | NA |
| OTU16243 | NA | NA | NA | NA |
| OTU1959 | NA | NA | NA | NA |
| OTU15870 | NA | NA | NA | NA |
| OTU18376 | NA | NA | NA | NA |
| OTU8039 | NA | NA | NA | NA |
| OTU6792 | + | NA | NA | NA |
| OTU9699 | NA | NA | NA | NA |
| OTU16411 | NA | NA | NA | NA |
| OTU13657 | NA | NA | NA | NA |
| OTU18863 | NA | NA | NA | NA |
| OTU10781 | NA | NA | NA | NA |
| OTU10714 | NA | NA | NA | NA |
| OTU16494 | NA | NA | NA | NA |
| OTU17095 | NA | NA | NA | NA |
| OTU11027 | NA | NA | + | NA |
| OTU413 | NA | NA | NA | NA |
| OTU14026 | NA | NA | NA | NA |
| OTU17295 | NA | NA | NA | NA |
| OTU978 | NA | NA | NA | NA |
| OTU12764 | NA | + | NA | NA |
| OTU10756 | NA | NA | NA | NA |
| OTU8028 | NA | NA | NA | NA |
| OTU170 | NA | NA | NA | NA |
| OTU3022 | + | NA | NA | NA |
| OTU5617 | NA | + | NA | NA |
| OTU6978 | NA | NA | NA | NA |
| OTU6663 | NA | NA | + | NA |
| OTU15881 | NA | NA | NA | NA |
| OTU18812 | NA | NA | NA | NA |
| OTU6454 | NA | NA | + | NA |
| OTU2213 | + | NA | NA | NA |
| OTU4035 | NA | NA | NA | NA |
| OTU10507 | NA | NA | + | NA |
| OTU18843 | NA | NA | NA | NA |
| OTU12737 | NA | NA | NA | NA |
| OTU15765 | NA | NA | NA | NA |
| OTU11520 | NA | NA | + | NA |
| OTU16042 | NA | NA | NA | + |
| OTU13400 | NA | NA | NA | NA |
| OTU2957 | NA | NA | NA | NA |
| OTU10513 | NA | NA | NA | NA |
| OTU7886 | + | NA | NA | NA |
| OTU12207 | NA | NA | NA | NA |
| OTU10967 | NA | NA | + | NA |
| OTU2479 | + | NA | NA | NA |
| OTU9495 | + | NA | NA | NA |
| OTU2927 | NA | NA | NA | NA |
| OTU11648 | NA | NA | + | NA |
| OTU786 | + | NA | NA | NA |
[truncated: 234,032 more chars]
