# Supplementary material for: Changes in the Distribution Preference of Soil Microbial Communities During Secondary Succession in a Temperate Mountain Forest
Source: Front Microbiol. 2022 Jun 17;13:923346. doi: 10.3389/fmicb.2022.923346 (PMC9247583; doi:10.3389/fmicb.2022.923346)
Supplement: Supplementary file 2 [file Data_Sheet_2.docx]

1.Venn

data(data1)

library('VennDiagram')

A<-A1[,1]

B<-B2[,1]

C<-C3[,1]

D<-D4[,1]

venn.plot <- venn.diagram(

x = list(

A=A,

D=D,

B=B,

C=C

),

filename = NULL,

col = "black",

lty = "dotted",

lwd = 4,

fill = c("cornflowerblue", "green", "yellow", "darkorchid1"),

alpha = 0.50,

label.col = c("orange", "white", "darkorchid4", "white", "white", "white",

"white", "white", "darkblue", "white",

"white", "white", "white", "darkgreen", "white"),

cex = 2.0,

fontfamily = "serif",

fontface = "bold",

cat.col = c("darkblue", "darkgreen", "orange", "darkorchid4"),

cat.cex = 2.5,

cat.fontfamily = "serif"

);

plot.new ()

grid.draw(venn.plot)

2. Kruskal–Wallis

library(multcomp)

library(ggpubr)

data(data1)

my_comparisons <- list(c("A", "B"),c("A", "C"), c("A", "D"), c("B","C"),c("B","D"),c("C","D"))

plot.new()

data$Succession.type=factor(data$Succession.type,c("A","B","C","D"))

ggboxplot(data,x="Succession.type",y="Species.richness",color="Succession.type",palette="jco",legend="none",add="jitter")+

stat_compare_means(comparisons=my_comparisons)+

stat_compare_means(label.y =29,cex=5.5)

3. rarefaction curve

library(picante)

library(ggplot2)

library(doBy)

data(otu)

alpha_index <- function(x, method = 'richness', tree = NULL, base = exp(1)) {

if (method == 'richness') result <- rowSums(x > 0)

else if (method == 'chao1') result <- estimateR(x)[2, ]

else if (method == 'ace') result <- estimateR(x)[4, ]

else if (method == 'shannon') result <- diversity(x, index = 'shannon', base = base)

else if (method == 'simpson') result <- diversity(x, index = 'simpson') else if (method == 'pielou') result <- diversity(x, index = 'shannon', base = base) / log(estimateR(x)[1, ], base)

else if (method == 'gc') result <- 1 - rowSums(x == 1) / rowSums(x) #goods_coverage

else if (method == 'pd' & !is.null(tree)) { #PD_whole_tree

pd <- pd(x, tree, include.root = FALSE)

result <- pd[ ,1]

names(result) <- rownames(pd)

}

result

}

alpha_curves <- function(x, step, method = 'richness', rare = NULL, tree = NULL, base = exp(1)) {

x_nrow <- nrow(x)

if (is.null(rare)) rare <- rowSums(x) else rare <- rep(rare, x_nrow)

alpha_rare <- list()

for (i in 1:x_nrow) {

step_num <- seq(0, rare[i], step)

if (max(step_num) < rare[i]) step_num <- c(step_num, rare[i])

alpha_rare_i <- NULL

for (step_num_n in step_num) alpha_rare_i <- c(alpha_rare_i, alpha_index(x = rrarefy(x[i, ], step_num_n), method = method, tree = tree, base = base))

names(alpha_rare_i) <- step_num

alpha_rare <- c(alpha_rare, list(alpha_rare_i))

}

names(alpha_rare) <- rownames(x)

alpha_rare

}

rarecurve(otu, step = 2000, col = c('red', 'green', 'blue', 'orange', 'purple', 'black'))

richness_curves <- alpha_curves(otu, step = 2000, method = 'richness')

plot_richness <- data.frame()

for (i in names(richness_curves)) {

richness_curves_i <- (richness_curves[[i]])

richness_curves_i <- data.frame(rare = names(richness_curves_i), alpha = richness_curves_i, sample = i, stringsAsFactors = FALSE)

plot_richness <- rbind(plot_richness, richness_curves_i)

}

rownames(plot_richness) <- NULL

plot_richness$rare <- as.numeric(plot_richness$rare)

plot_richness$alpha <- as.numeric(plot_richness$alpha)

ggplot(plot_richness, aes(rare, alpha, color = sample)) +

geom_line() +

labs(x = 'Number of sequences', y = 'Richness', color = NULL) +

theme(panel.grid = element_blank(), panel.background = element_rect(fill = 'transparent', color = 'black'), legend.key = element_rect(fill = 'transparent')) +

scale_x_continuous(breaks = seq(0, 6000000, 260000), labels = as.character(seq(0, 6000000, 260000)))

4. heat map

library(gstat)

library(sp)

data(data1)

x=rep(seq(10,90,by=2.5),each=40)

y=rep(seq(10,90,by=2.5),40)

lin.grid=data.frame(x,y)

coordinates(lin.grid)=~x+y

gridded(lin.grid)=TRUE

AN<-data

coordinates(AN)=~x+y

AN.kriged=krige(多样性~1,AN,lin.grid)

plot.new()

spplot(AN.kriged["var1.pred"],col.regions=terrain.colors(16),xlab ="West-→East(m)", ylab = "South-→North(m)")

baiyunshan=matrix(data1$z,nrow=6,ncol=6)

x<-c(0,20,40,60,80,100)

y<-c(0,20,40,60,80,100)

plot.new ()

layout(matrix(c(1:4),2,2,byrow=T))

contour(x,y,baiyunshan,nlevels=20,xlab="A（m）",labcex=1.2,cex.lab=1.5)

5. species accumulation diagrams

library('ggplot2')

SLE=read.csv('细菌.csv')

SLE$Habitat=c(1:100)

mydata_SLE <- melt(SLE,

id.vars="Habitat",variable.name="Genus",value.name="Percent")

colourCount =30

getPalette = colorRampPalette(brewer.pal(12, "Spectral")) ggplot(mydata_SLE,aes(Habitat,Percent,fill=Genus))+ geom_bar(stat="identity",position="fill",width=0.7)+

ggtitle("Bacteria")+

theme_minimal()+

scale_fill_manual(values = getPalette(colourCount)) +

theme(legend.position = "right") +

guides(fill=guide_legend(ncol=1))+

coord_flip()

6.RDA

library(vegan)

library(plyr)

library(gglayer)

library(ggplot2)

library(ggrepel)

fc<-read.csv("环境")

sp<-read.csv("物种")

spp=decostand(sp,method = "hellinger")uu=rda(spp~.,fc)#RDA分析

ii=summary(uu)

anova(uu, by = "term", permutations=999)

sp=as.data.frame(ii$species[,1:2])/2st=as.data.frame(ii$sites[,1:2])

yz=as.data.frame(ii$biplot[,1:2])

grp=as.data.frame(c(rep("a",25),rep("b",25), rep("c",25),rep("d",25)))

colnames(grp)="group"

ggplot() +

geom_point(data = st,aes(RDA1,RDA2,shape=grp$group,fill=grp$group),size=6)+

scale_shape_manual(values = c(20:25))+

geom_ord_ellipse(aes(st$RDA1,st$RDA2,color=grp$group,group=grp$group), ellipse_pro = 0.68,linetype=3,size=1)+

geom_segment(data = sp,aes(x = 0, y = 0, xend = RDA1, yend = RDA2),

arrow = arrow(angle=22.5,length = unit(0.35,"cm"),

type = "closed"),linetype=1, size=0.6,colour = "white")+ geom_segment(data = yz,aes(x = 0, y = 0, xend = RDA1, yend = RDA2),

arrow = arrow(angle=22.5,length = unit(0.35,"cm"),

type = "closed"),linetype=1, size=0.6,colour = "tomato3")+ geom_text_repel(data = yz,aes(RDA1,RDA2,label=row.names(yz)))+

labs(x=paste("RDA 1 (", format(100 *ii$cont[[1]][2,1], digits=6), "%)", sep=""),

y=paste("RDA 2 (", format(100 *ii$cont[[1]][2,2], digits=6), "%)", sep=""))+

geom_hline(yintercept=0,linetype=3,size=1) +

geom_vline(xintercept=0,linetype=3,size=1)+

guides(shape=guide_legend(title=NULL),color=guide_legend(title=NULL),

fill=guide_legend(title=NULL))+

theme_bw()+theme(panel.grid=element_blank())

library(rdacca.hp)

data(mite)

data(mite.env)

mite.hel <- decostand(mite, method = 'hellinger')

mite.rda <- rda(mite.hel~., mite.env, scale = FALSE)

exp_adj <- RsquareAdj(mite.rda)$adj.r.squared * mite.rda$CCA$eig/sum(mite.rda$CCA$eig)

rda1_exp <- paste('RDA1:', round(exp_adj[1]*100, 2), '%')

rda2_exp <- paste('RDA2:', round(exp_adj[2]*100, 2), '%')

plot(mite.rda, display = c('wa', 'cn'), type = 'n', xlab = rda1_exp, ylab = rda2_exp)

text(mite.rda, display = 'cn', col = 'blue', cex = 0.8)

points(mite.rda, display = 'wa', pch = 19, cex = 1)

mite.rda.hp <- rdacca.hp(mite.hel, mite.env, method = 'RDA', type = 'adjR2', scale = FALSE)

mite.rda.hp$Hier.part

7.Torus

data(data1)

yun=read.csv("data1")

plotdata<-as.matrix(yun)

toursonesp=function(plotdata,spcol,gx,gy,gridsize)

{

plotdimqx=gx/gridsize # Calculates no. of x-axis quadrats of plot.

plotdimqy=gy/gridsize # Calculates no. of y-axis quadrats of plot.

totmat=matrix(plotdata[,3],plotdimqy,plotdimqx)# creates a total abundance matrix

habmat=matrix(plotdata[,2],plotdimqy,plotdimqx) #creates the habitat matrix

spmat=matrix(plotdata[,spcol],plotdimqy,plotdimqx)#creates the focus species matrix

a<-plotdata[,2]

num.habs=length(unique(a))

realden=numeric()

GrLsEq=matrix(0,1,num.habs*4)

spstcnthab=numeric()

totstcnthab=numeric()

for(i in 1:num.habs){

spstcnthab[i]=sum(spmat[habmat==i])

totstcnthab[i]=sum(totmat[habmat==i])

realden[i]=spstcnthab[i]/totstcnthab[i]}

for(x in 0:(plotdimqx-1))

{

for(y in 0:(plotdimqy-1))

{

newhab=matrix(0,plotdimqy,plotdimqx)

# The following "if" statements create the x,y torus-translation of the habitat map.

if(y==0 & x==0)

newhab=habmat

if(y==0 & x>0)

newhab=habmat[c(1:plotdimqy),c((plotdimqx-x+1):plotdimqx,1:(plotdimqx-x))] #

if(x==0 & y>0)

newhab=habmat[c((plotdimqy-y+1):plotdimqy,1:(plotdimqy-y)),c(1:plotdimqx)] # \ue068\ue01c

if(x>0 & y>0)

newhab=habmat[c((plotdimqy-y+1):plotdimqy,1:(plotdimqy-y)),c((plotdimqx-x+1):plotdimqx,1:(plotdimqx-x))] #

Torspstcnthab=numeric() # Creates empty vector for stem counts per sp. per habitat in torus-based maps.

Tortotstcnthab=numeric() # Creates empty vector for tot. stem counts per habitat in torus-based maps.

for(j in 1:num.habs)

{

Tortotstcnthab[j]=sum(totmat[newhab==j]) # Determines tot. no. stems per habitat of the focal torus-based map.

Torspstcnthab[j]=sum(spmat[newhab==j]) # Determines tot. no. stems for focal sp. per habitat of the focal torus-based map.

}

Torspprophab=Torspstcnthab/Tortotstcnthab # Calculates relative stem density of focal sp. per habitat of the focal torus-based map.

for(i in 1:num.habs)

{

if(realden[i]>Torspprophab[i]) # If rel. dens. of focal sp. in focal habitat of true map is greater than rel. dens. of focal sp. in focal habitat of torus-based map, then add one to "greater than (Gr.Hab)" count.

GrLsEq[1,(4*i)-2]=GrLsEq[1,(4*i)-2]+1

if(realden[i]<Torspprophab[i]) # If rel. dens. of focal sp. in focal habitat of true map is less than rel. dens. of focal sp. in focal habitat of torus-based map, then add one to "less than (Ls.Hab)" count.

GrLsEq[1,(4*i)-1]=GrLsEq[1,(4*i)-1]+1

if(realden[i]==Torspprophab[i]) # If rel. dens. of focal sp. in focal habitat of true map is equal to rel. dens. of focal sp. in focal habitat of torus-based map, then add one to "equal to (Eq.Hab)" count.

GrLsEq[1,4*i]=GrLsEq[1,4*i]+1

}

} # Closes "for loop" through all 20-m translations along x-axis.

} # Closes "for loop" through all 20-m translations along y-axis.

for(i in 1:num.habs)

{

GrLsEq[1,(4*i)-3]=realden[i]

}

p_value=vector()

for(n in 1:num.habs){

p_value[n]=(GrLsEq[1,n*4-2]+GrLsEq[1,n*4])/(GrLsEq[1,n*4-2]+GrLsEq[1,n*4-1]+GrLsEq[1,n*4])

}

habt=unique(plotdata[,2])

species=rep(names(plotdata)[spcol],length(habt))

determination=vector()

for(i in 1:length(habt))

{if (p_value[i]<0.05)

determination[i]=c("significent negative corralation")

else if (p_value[i]>0.95)

determination[i]=c("significent positive corralation")

else if(p_value[i]<=0.95&p_value[i]>=0.05)

determination[i]=c("stochastic distribution")

}

return(data.frame(spcol,habt,p_value,determination))

}

toursonesp(plotdata,4,100,400,20)
